# Signatures of selection and environmental adaptation across the goat genome post-domestication

**DOI:** 10.1186/s12711-018-0421-y

**Published:** 2018-11-19

**Authors:** Francesca Bertolini, Bertrand Servin, Andrea Talenti, Estelle Rochat, Eui Soo Kim, Claire Oget, Isabelle Palhière, Alessandra Crisà, Gennaro Catillo, Roberto Steri, Marcel Amills, Licia Colli, Gabriele Marras, Marco Milanesi, Ezequiel Nicolazzi, Benjamin D. Rosen, Curtis P. Van Tassell, Bernt Guldbrandtsen, Tad S. Sonstegard, Gwenola Tosser-Klopp, Alessandra Stella, Max F. Rothschild, Stéphane Joost, Paola Crepaldi, the AdaptMap consortium

**Affiliations:** 1 Department of Animal Science, Iowa State University, Ames, IA 50011 USA; 2 National Institute of Aquatic Resources, Technical University of Denmark (DTU), 2800 Lyngby, Denmark; 3 GenPhySE, INRA, Université de Toulouse, INPT, ENVT, 31326 Castanet Tolosan, France; 4 Dipartimento di Medicina Veterinaria, Università degli Studi di Milano, 20133 Milan, Italy; 5 Laboratory of Geographic Information Systems (LASIG), School of Architecture, Civil and Environmental Engineering (ENAC), Ecole Polytechnique Fédérale de Lausanne (EPFL), 1015 Lausanne, Switzerland; 6 Recombinetics Inc, St Paul, 55104 MN USA; 7 Consiglio per la ricerca in agricoltura e l’analisi dell’economia agraria (CREA) - Research Centre for Animal Production and Acquaculture, 00015 Monterotondo, Roma, Italy; 8 Centre for Research in Agricultural Genomics (CRAG), CSIC-IRTA-UAB-UB, Campus Universitat Autonoma de Barcelona, Bellaterra, 08193 Barcelona, Spain; 9 DIANA Dipartimento di Scienze Animali, della Nutrizione e degli Alimenti, Università Cattolica del S. Cuore, 29100 Piacenza, Italy; 10 BioDNA Centro di Ricerca sulla Biodiversità e sul DNA Antico, Università Cattolica del S. Cuore, 29100 Piacenza, Italy; 11 Fondazione Parco Tecnologico Padano (PTP), 26900 Lodi, Italy; 12 Department of Support, Production and Animal Health, School of Veterinary Medicine, São Paulo State University (UNESP), Araçatuba, Brazil; 13 Animal Genomics and Improvement Laboratory, ARS USDA, Beltsville, MD 20705 USA; 14 Center for Quantitative Genetics and Genomics, Aarhus University, 8830 Tjele, Denmark

## Abstract

**Background:**

Since goat was domesticated 10,000 years ago, many factors have contributed to the differentiation of goat breeds and these are classified mainly into two types: (i) adaptation to different breeding systems and/or purposes and (ii) adaptation to different environments. As a result, approximately 600 goat breeds have developed worldwide; they differ considerably from one another in terms of phenotypic characteristics and are adapted to a wide range of climatic conditions. In this work, we analyzed the AdaptMap goat dataset, which is composed of data from more than 3000 animals collected worldwide and genotyped with the CaprineSNP50 BeadChip. These animals were partitioned into groups based on geographical area, production uses, available records on solid coat color and environmental variables including the sampling geographical coordinates, to investigate the role of natural and/or artificial selection in shaping the genome of goat breeds.

**Results:**

Several signatures of selection on different chromosomal regions were detected across the different breeds, sub-geographical clusters, phenotypic and climatic groups. These regions contain genes that are involved in important biological processes, such as milk-, meat- or fiber-related production, coat color, glucose pathway, oxidative stress response, size, and circadian clock differences. Our results confirm previous findings in other species on adaptation to extreme environments and human purposes and provide new genes that could explain some of the differences between goat breeds according to their geographical distribution and adaptation to different environments.

**Conclusions:**

These analyses of signatures of selection provide a comprehensive first picture of the global domestication process and adaptation of goat breeds and highlight possible genes that may have contributed to the differentiation of this species worldwide.

**Electronic supplementary material:**

The online version of this article (10.1186/s12711-018-0421-y) contains supplementary material, which is available to authorized users.

## Background

The goat (*Capra hircus*) is considered one of the earliest domesticated livestock species. The domestication process started around 10,000 years ago in the Fertile Crescent area from a unique wild and still living ancestor, the bezoar or *Capra aegagrus* [1]. At present, there are more than one billion of goats that inhabit all types of ecological areas across the globe [2]. Compared with the other major livestock species such as pigs, cattle and sheep, goats have undergone the largest increase (+34%) in population since 2000, i.e. larger than pigs (+15%), cattle (+14%) and sheep (+14%) (http://www.fao.org/faostat/en/). Today, over 90% of the goats are distributed across Asia and Africa, followed by the Americas, Europe, and Oceania [3]. In the most rural areas of the world, goats are often considered the poor person’s cow. In fact, goats can be used for milk, meat, fiber, and leather production [4], as well as transport. Moreover, they are easy to house and manage; goats can be raised by small families, women, and children and provide a fundamental source of food for millions of people. Several factors have contributed to the differentiation of goat breeds, which are classified mainly into two types: (i) adaptation to different breeding systems and/or purposes, i.e. in some countries, breeds have been selected for specific production traits such as milk (e.g. Saanen and Alpine), meat (e.g. Landrace and Boer) and fiber (e.g. Angora and Cashmere); and (ii) adaptation to different environments, i.e. goats have adapted to various agro-climatic conditions. In addition, goat breeds have undergone differentiation through founder effects, and the processes of admixture and genetic drift. Thus, about 600 breeds have been developed and are distributed worldwide [5]. They differ from one another in terms of many phenotypic characteristics such as size, color, horn shape and dimension, reproductive and productive traits and are adapted to a wide range of bioclimatic conditions. Directional natural and artificial selection events have left footprints across the genome, which are known as signatures of selection. Signatures of selection are defined as the reduction, elimination or change of genetic variation in genomic regions that are adjacent to causative variants in response to natural or artificial selective pressure. Such variants usually affect several traits and contribute to shaping a breed [6]. The process by which the frequency of a selectively favored variant increases in a population is termed a selective sweep. The recent development of species-specific genomic tools (such as single nucleotide polymorphism (SNP) arrays) have allowed researchers to extend whole-genome analyses to livestock species, which cover many aspects of genetic diversity, including signatures left by selection processes [7].

In spite of the major economic importance of goats, high-throughput genomic resources for this species have become available only recently. In 2011, the International Goat Genome Consortium developed and released the first high-throughput SNP chip with more than 50,000 SNPs (Illumina CaprineSNP50 BeadChip), which was built using 10 biologically and geographically different breeds [8]. The first complete assembly of the goat genome was released in 2013 by Dong et al. [9] and a second version of the reference genome that exploits single-molecule long read sequencing (PacBio) has just been released (ARS1; [10]). With this new version, gene annotation has improved considerably and the position of the SNPs on the CaprineSNP50 BeadChip has been updated. With the availability of genomic and high-throughput SNP tools, there is an increased interest in identifying and exploring signatures of selection and the genomic diversity resulting from adaptation to environment and human selective pressure. These genomic tools were used to identify signatures of selection in circumscribed datasets. The CaprineSNP50 BeadChip was used to investigate and compare several Swiss goat breeds and genomic signatures of selection were detected in regions that affect variation in coat color, growth, and milk composition [11]. The combination of analyses of runs of homozygosity (ROH), *F*_ST_ (fixation index), XP-EHH (cross population extended haplotype homozygosity), and the use of Bayesian methods allowed the detection of signatures of selection in a region that contains genes related to the immune system in another mountain breed raised in the North of Italy, the Valdostana Italian goat breed [12]. Other analyses of signatures of selection were performed in a reduced number of breeds, thus detecting regions that are linked to production and reproduction traits [13] and in the Barki Egyptian goat breed, which is raised and adapted to hot/dry environments [14]. In the latter study, analyses of iHS (integrated haplotype score*)* and pairwise *F*_ST_ that identified selective sweeps led to the identification of genes related to thermotolerance, body size, energy metabolism and nervous and auto-immune response [14]. Furthermore, signatures of selection that were linked to dry and hot conditions and to metabolic traits were identified by using whole-genome sequence (WGS) data from Moroccan goat breeds through XP-CLR analysis [15]. WGS information was also successfully used to detect regions that are under different selection pressures in Chinese and Mongolian goats and are related to breeding or reproductive traits [16, 17].

Genomic changes that result from climate changes and are linked to adaptation to different environments can be analyzed with a landscape genomic approach, which was successfully applied in other livestock species, for example in Ugandan cattle [18]. To date in goats, this approach was applied only on a reduced number of SNPs or amplified fragment length polymorphism (AFLP) markers [19, 20]. However, it would be interesting to apply it at the genomic level since it provides useful information on the environmental factors that shaped the genome.

In this work, we used different approaches to identify regions under artificial and environmental selection across the AdaptMap goat dataset, which is composed of data from more than 3000 animals that were collected worldwide and genotyped with the CaprineSNP50 BeadChip. For this purpose, we considered several groups of animals that were partitioned based on geographical area, as done in Colli et al. [21], production uses, available records on solid coat color and environmental variables in relation to the geographical coordinates of sampling. For the first time, we applied FLK and hapFLK analyses on goat data, which have been successfully used in analyses of high-throughput data from sheep [22, 23]. These methods increase the power of detection for signatures of selection, and they enable detection of soft or incomplete selective sweeps. Finally, we also applied landscape genomic approaches to this large goat dataset, to investigate the role of natural selection in shaping the goat genome.

## Methods

Prior to the application of methods for detecting signatures of selection, we applied several filtering steps to the AdaptMap goat dataset, which originally contained 4563 animals from 144 breeds that were collected worldwide and genotyped with the CaprineSNP50 BeadChip. This dataset was first edited by removing mixed breeds, related animals, SNPs that were monomorphic, unmapped, mapped to sex chromosomes or with low call rate, which resulted in a working dataset of 46,654 SNPs and 3197 animals [21].

Additional filtering steps were applied depending on the analysis performed, as described below. The main analyses are summarized as follows: (1) detection of signatures of selection based on the genetic diversity of subcontinental populations or breeds, and (2) detection of signatures of selection associated with specific phenotypes, traits or external variables (e.g. annual mean temperature based on global positioning system (GPS) coordinates). In general, each investigation was conducted using two complementary approaches. For each analysis, genes within selected regions or nearby detected SNPs (± 100 kb) were identified using the Bedtools software [24] and the most recent version of the goat genome assembly (ARS1; [10]).

### Signatures of selection based on genetic diversity of subcontinental populations or breeds

#### Signatures between and within subcontinental groups

To detect signatures of major differentiation between populations, we used the FLK [25] and hapFLK [26] approaches. Briefly, these methods account for population structure and differences in effective population size by modelling the genetic divergence between populations as derived from drift and population division. Because these methods are not completely robust to strong bottlenecks and large admixture events, subsets of populations were selected starting from the working dataset, removing admixed animals and strongly inbred populations. This was done first by identifying genetic sub-structure in the initial diversity analysis which defined sub-continental groups [21]. Then, an admixture analysis was carried out to identify and remove admixed populations within each sub-continental group, which generated sub-continental filtered groups. This last step was performed using the Treemix software [27], allowing for up to three migration events within each group. Graphical representations of the results of all Treemix analyses are in Additional file 1. The number of animals included in each sub-continental group is in Table 1. The final dataset comprised 2481 individuals that are grouped into 62 (61 *Capra hircus* + one *Capra aegagrus*) populations.

**Table 1:** Number of animals and breeds that composed the sub-continental groups after filtering steps

Groups	Breed code	Breed name (Country)	Number
Alpines	ALP_FR	*Alpine (France)*	50
	BIO	*Bionda dell’Adamello*	24
	FSS	*Fosses*	24
	ORO	*Orobica*	22
	PTV	*Poitevine*	27
	SAA_FR	*Saanen (France)*	50
	VAL	*Valdostana*	24
	VSS	*Valpassiria*	24
Angoras	ANG_AR	*Angora (Argentina)*	50
	ANG_FR	*Angora (France)*	26
	ANG_ZA	*Angora (South Africa)*	48
	ANK	*Ankara*	18
	IRA	*Iranian goat (unknown)*	9
	KIL	*Kil*	23
Boers	BOE_AU	*Boer (Australia)*	32
	BOE_CH	*Boer (Switzerland)*	50
	BOE_US	*Boer (United States)*	29
	BOE_ZW	*Boer (Zimbawe)*	17
Central Asia	THA	*Thari*	16
	TED	*Teddi*	47
	PAH	*Pahari*	19
	KAC	*Kachan*	19
	DDP	*DDP*	20
East Africa	ABR	*Abergelle*	49
	GAL	*Galla*	23
	GUM	*Gumez*	39
	KAR	*Karamonja*	19
	KEF	*Keffa*	44
	MAA	*Maasai*	18
	PRW	*Pare White*	19
	SEA	*Small East African*	50
	SEB	*Sebei*	21
	SNJ	*Sonjo*	20
	WYG	*Woyito Guji*	39
Egypt	BRK	*Barki*	50
	NBN_EG	*Nubian (Egypt)*	50
	OSS	*Oasis*	50
	SID	*Saidi*	50
Northern Europe	LNR_DK	*Landrace (Denmark)*	50
	LNR_FI	*Landrace (Finland)*	20
	NRW	*Norwegian*	17
North west Africa	CAM	*Cameroon goat*	37
	GUE	*Malagueña*	16
	PEU	*Peulh*	22
	RSK	*Red Sokoto*	19
	SAH	*Sahel*	15
	TAR	*Targui*	19
	WAD	*West African Dwarf*	50
South Africa	DZD	*Dedza*	15
	LND	*Landin*	29
	MSH	*Mashona*	22
South eastern Europe	ARG	*Argentata*	24
	CCG	*Ciociara Grigia*	16
	DIT	*Di Teramo*	19
	GAR	*Garganica*	15
	GGT	*Girgentana*	24
South western Europe	BEY	*Bermeya*	23
	MAL	*Mallorquina*	18
	MLG	*Malaguena*	40
	MUG	*Murciano*-*Granadina*	20
	PYR	*Pyrenean*	26
	RAS	*Blanca de Rasquera*	20

Country is indicated when necessary for the analysis

Following filtering of the data, FLK and hapFLK analyses were carried out on each of the sub-continental filtered groups, using the wild ancestor of domestic goat (Bezoar, *Capra aegagrus*) as an outgroup to root population trees. For hapFLK, the cross-validation procedure was performed with the fastPHASE software [28], which determined that 30 haplotype clusters were needed to capture haplotype diversity. For both hapFLK and FLK analyses, p-values were computed as explained in the hapFLK software documentation. False discovery rates (FDR) were estimated using the qvalue R package [29] and SNPs corresponding to an FDR of 0.15 or less were considered significant.

A genome scan for signatures of selection between sub-continental groups was also performed using FLK analysis as described by Fariello et al. [22]. Briefly, the frequency of the ancestral allele of each group was estimated from the within-group analysis. These ancestral alleles were then used to perform a new genome scan using FLK analysis. Frequencies of Bezoar alleles were used to root the population tree. Only the groups that corresponded to clear geographical clustering of goat populations were considered for this ancestral analysis, i.e. the Southwestern, Southeastern, Northern and Alpine European groups, the Central Asian group and the Northwestern, Easter and Southern African groups.

#### Signatures of selection within breeds

The above-mentioned filtered dataset that was used for FLK and hapFLK analyses was also used to detect signatures of selection within the breeds using: (i) ROH and (ii) iHS statistic [30]. ROH analyses were performed using the Zanardi software [31] by considering a minimum of 15 SNPs per ROH, a minimum ROH length of 1 Mb and allowing for one heterozygous SNP within an ROH to account for the possibility of genotyping errors. For each SNP, the proportion of animals that displayed a homozygous region at that SNP was calculated. Then, this measure was transformed by its empirical quantile for all SNPs across the genome (*i.e.* the proportion of all SNPs with a higher or equal proportion of homozygous animals in the breed considered). The iHS analyses were performed in populations with at least 10 genotyped individuals. Within each population, phasing of individuals was performed using SHAPEIT2 [32]. For SNPs with a minor allele frequency higher than 0.05, the ancestral alleles were randomly assigned. The iHS statistics were calculated using the rehh v2.0 R package [33]. Standardized iHS values were computed in allele frequency bins of 0.05 and then further corrected using robust estimations of their mean and variance using the rlm function from the MASS R package [34]. Because assessing significance of ROH and iHS values is less robust, ROH and iHS signals were reported only for significant FLK or hapFLK signatures of selection.

### Signatures of selection associated with specific traits or external covariates

#### External phenotypes and production traits

Reduced subsets of the dataset were investigated to detect genomic regions associated with specific phenotypes. Reductions were performed according to the availability of the information for each animal or breed included in the dataset. Therefore, two reduced panels were created corresponding to differences related to production purposes and phenotypes (solid coat colors).

##### Panel 1: production purposes

A questionnaire that contained four possible production assignments (milk, meat, fiber, and leather) was circulated to all AdaptMap members who provided the samples, to obtain information on the main purpose of each breed. Pre-filtering was performed by considering only breeds with known and prevalent purposes. Since none of the breeds was described as being solely raised for leather production, this purpose was excluded from subsequent analyses.

Then, another filtering step was performed on the working dataset to remove breeds that are raised for more than one purpose by using an in-house script that discarded animals belonging to different purpose groups that shared the same coordinates of the first component ± 1.5 of the overall quantile distribution (ee Additional file 2: Figure S1). The final filtered dataset included 192 animals (three breeds) for fiber, 241 animals (12 breeds) for meat and 818 animals (23 breeds) for milk. Details on breeds and sample sizes are in Table 2a. Three types of analyses were carried out on the final filtered dataset: ROH, *F*_ST_ and XP-EHH. ROH analyses were performed for each separate purpose group using the Zanardi software [31] by considering a minimum of 15 SNPs per ROH, a minimum ROH length of 1 Mb and allowing for one heterozygous SNP within an ROH to account for the possibility of genotyping errors, as previously mentioned. The *F*_ST_ and XP-EHH analyses were carried out by comparing each group against all the others and using the script described by Talenti et al. [12] in which 1-Mb windows with an overlap of 500 kb were considered for window-based *F*_ST_ and the Selscan software [35] for XP-EHH. The results were normalized with the norm normalization tool included in the software suite.

**Table 2:** List of filtered animals and breeds for comparisons of breeds with different production purposes^a^ and coat colors^b^

Purpose^a^	Breed code	Breed name	Number	Coat color^b^	Breed code	Breed name	Number
Milk	ALP	*Alpine (Camosciata delle Alpi)*	150	White	ANG	*Angora*	131
	ARG	*Argentata*	24		ANK	*Ankara*	18
	ASP	*Aspromontana*	23		CAS	*Nicastrese*	44
	BIO	*Bionda dell’Adamello*	24		CRP	*Carpathian*	14
	CCG	*Ciociara Grigia*	16		GAL	*Galla*	23
	CRS	*Corse*	29		SAA	*Saanen*	145
	DIT	*Di Teramo*	19	Black	DDP	*DDP*	20
	GAR	*Garganica*	15		KIL	*Kil*	23
	LNR	*Landrace*	85		KLS	*Kilis*	36
	MLG	*Malagueña*	40	Red	BEY	*Bermeya*	23
	MLS	*Maltese Sarda*	12		RME	*Rossa Mediterranea*	30
	MLT	*Maltese*	16		MLG	*Malaguena*	40
	MUG	*Murciano*-*Granadina*	20				
	NIC	*Nicastrese*	20				
	NRW	*Norwegian*	17				
	ORO	*Moroccan*	22				
	PTV	*Poitevine*	27				
	PVC	*Provencale*	17				
	RME	*Rossa Mediterranea*	30				
	SAA	*Saanen*	142				
	SAR	*Sarda*	27				
	TOG	*Toggenburg*	19				
	VSS	*Valpassiria*	24				
Meat	BOE	*Boer*	138				
	BRI	*Bari*	25				
	LOP	*Local_Pothohari*	13				
	RAN	*Murciano*-*Granadina*	2				
	TED	*Teddi*	47				
	THA	*Thari*	16				
Fiber	ANG	*Angora*	131				
	ANK	*Ankara*	18				
	CAS	*Cashmere*	43				

For all three analyses (ROH, window-based *F*_ST_ and XP-EHH), the top 0.5% of SNPs based on marker or window distribution was retained as relevant. The Bedtools software [24] was used to find consensus regions between two or all three approaches. Only regions detected by at least two approaches were considered for further analyses.

##### Panel 2: solid coat colors

Pictures representative of the breeds for each animal of the working dataset were provided by the AdaptMap members and were inspected to find common and unique patterns across each breed. Considering the high variability of patterns and the lack of availability of high-quality pictures for some breeds, only those with confident solid coat colors were considered. Three groups were created: a Black group (79 animals and three breeds), a White group (375 animals and six breeds), and a Red group (93 animals and three breeds). Breeds and samples sizes are in Table 2b. For each group, a *F*_ST_ analysis was carried out by considering the following pairwise comparisons: White vs. Black and Red, Black vs. White and Red vs. White. These comparisons were performed using the script described by Talenti et al. [12] for which 500-kb windows with overlaps of 250 kb were considered. For each comparison, the top four windows that contained at least four SNPs, corresponding to the 0.9996 percentile of the overall distribution were considered. The genes within these windows were screened using the coat color gene website of the International Federation of Pigment Cell Society (http://www.espcr.org/micemut) to investigate their direct or indirect associations with coat colors in mice and shades of skin color in humans.

SNPs that were located on either side of candidate genes for both purpose and coat color panels (± 5 Mb = short and ± 10 Mb = long) were used as sets of variables with the same animals for canonical discriminant analysis (CDA), which was performed with the CANDISC procedure implemented in SAS-stat software (SAS Institute, Inc., vers.9.4). These analyses allowed the identification of the SNPs that contributed most to the discrimination between groups. These contributions were summarized as canonical functions (CAN), which were linear combinations of the original variables (discriminant SNPs). Visual inspection of the CAN1 vs. CAN2 scatter plot of the CAN1 and CAN2 values for each SNP was used to pinpoint the precise regions associated with the separation between groups.

#### Signatures of selection associated with bioclimatic environmental variables

To study the influence of the environment on the distribution of adaptive genetic variation, a landscape genomic approach using the Samβada software [36] and *F*_ST_ analyses were applied to identify genotypes that are significantly associated with environmental variables. For this purpose, we used a reduced subset of samples with known geographic coordinates of the sampling point. This reduced dataset contained 2661 animals from 28 countries (Table 3). Finally, an additional filtering step was applied before performing the *F*_ST_ analyses (see below).

**Table 3:** List of breeds (name, code and number of animals per breed) with available GPS coordinates used for landscape genomic analysis

Breed code	Breed name	Number	Breed code	Breed name	Number	Breed code	Breed name	Number
ABR	*Abergelle*	49	GHA	*Ghazalia*	4	NSJ	*Nsanje*	6
ALB	*Alpine x Boer*	5	GOG	*Gogo*	12	OIG	*Old Irish Goat*	11
ALP	*Alpine (Camosciata delle Alpi)*	146	GUE	*Guera*	16	ORO	*Orobica*	22
AND	*Nganda*	6	GUM	*Gumez*	39	OSS	*Oasis*	50
ANG	*Angora*	80	IRA	*Iranian goat*	9	PAF	*Pafuri*	4
ARG	*Argentata*	24	JAT	*Jattan*	15	PAH	*Pahari*	19
ARR	*Traditional Arran*	8	JON	*Jonica*	11	PAL	*Palmera*	14
ASP	*Aspromontana*	23	KAC	*Kachan*	19	PAT	*Pateri*	27
BAB	*Barbari*	16	KAM	*Kamori*	38	PEU	*Peulh*	22
BAR	*Barcha*	4	KAR	*Karamonja*	19	PRW	*Pare White*	19
BAW	*Balaka*-*Ulongwe*	12	KEF	*Keffa*	44	PTV	*Poitevine*	27
BEY	*Bermeya*	23	KES	*Koh*-*e*-*sulmani*	13	PVC	*Provençale*	15
BEZ	*Bezoar*	7	KIG	*Kigezi*	4	PYR	*Pyrenean*	26
BIO	*Bionda dell’Adamello*	24	LGW	*Lilongwe*	3	RAN	*Rangeland*	50
BLB	*Bilberry*	10	LND	*Landin*	29	RAS	*Blanca de Rasquera*	20
BOE	*Boer*	108	LNR	*Landrace*	50	RME	*Rossa Mediterranea*	30
BRI	*Bari*	25	LOH	*Lohri*	17	RSK	*Red Sokoto*	19
BRK	*Barki*	50	LOP	*Local Pothohari*	13	SAA	*Saanen*	106
BUT	*Bugituri*	31	MAA	*Maasai*	18	SAH	*Sahel*	15
CAM	*Cameroon goat*	37	MAL	*Mallorquina*	18	SDN	*Soudanaise*	22
CAN	*Caninde*	23	MAN	*LaMancha*	3	SEA	*Small East African*	50
CAS	*Cashmere*	44	MAU	*Maure*	13	SEB	*Sebei*	21
CCG	*Ciociara Grigia*	1	MEN	*San Clemente*	19	SHL	*Sahel*	19
CHA	*Chappar*	9	MLG	*Malagueña*	24	SID	*Saidi*	50
CRE	*Creole*	49	MLY	*Malya*	11	SNJ	*Sonjo*	20
CRO	*Local Cross*	5	MOR	*Moroccan goat*	10	SOF	*Sofia*	22
CRP	*Carpathian*	14	MOX	*Moxoto’*	23	SOU	*SudOuest*	8
CRS	*Corse*	29	MSH	*Mashona*	22	TAP	*Tapri*	22
DDP	*DDP*	20	MTB	*Matebele*	22	TAR	*Targui*	19
DIA	*Diana*	14	MUB	*Mubende*	18	TED	*Teddi*	47
DJA	*Djallonke*	10	MUG	*Murciano*-*Granadina*	20	THA	*Thari*	16
DRA	*Draa*	4	NAI	*Naine*	14	THY	*Thyolo*	9
DZD	*Dedza*	15	NBN	*Nubian*	63	TOG	*Toggenburg*	20
FSS	*Fosses*	24	NDA	*Noire de l’Atlas*	4	TUN	*Tunisian*	21
GAL	*Galla*	23	NGD	*Nganda*	11	VAL	*Valdostana*	24
GAR	*Garganica*	15	NOR	*Nord*	4	WAD	*West African Dwarf*	49
GAZ	*Gaza*	4	NRW	*Norwegian*	17	WYG	*Woyito Guji*	39

##### Landscape genomic analyses

The environmental conditions were characterized using the bioclimatic variables from the WorldClim database (http://www.worldclim.org/) (see Additional file 3: Table S1). These data were available worldwide at a resolution of one arc-second and represented an average of the conditions from years 1950 to 2000. The values of these variables were extracted for the coordinates of each sampling point using the QGIS 2.14.7 software and were centered-reduced. A buffer area around each sampling point was generated to integrate, into the model, the environmental variability of the area that could influence an individual. Here, a radius of 5 km was selected which assumed that the goat could move in a circular area of 10 km in diameter, centered on the sampling point. Within that area, in order to consider only the area corresponding to the potential home range of the goats, a land cover discrimination based on the Global Land Cover 2000 classification was applied [37]. On this basis, the areas corresponding to artificial surfaces (urban land cover) and water bodies were removed. For the remaining areas, the bioclimatic variables were retrieved and for each one, seven statistical measures (minimum, maximum, mean, standard deviation, range, median and mode) were computed.

The Samβada software [36] was used to compute the parallel processing of multiple univariate logistic regressions between each genotype versus each environmental variable. All univariate models were computed and filtered considering a significance threshold of 0.05 before Bonferroni correction. Then, a second step of filtering was applied to retrieve only the SNPs for which at least two genotypes were significantly associated with an environmental variable and showed simultaneously: (1) a very strong effect of the environment on the genotype (absolute value of the β1 regression coefficient higher than the 0.99 percentile of all absolute values of β1 of the significant models), (2) a high statistical significance of the association (G and Wald scores higher than the 0.95 percentile of all G and Wald scores of the significant models) and (3) a strong goodness of fit of the models, with an Akaike’s information criterion (AIC) lower than the 0.10 percentile of all AIC of the significant models. The Enrichr database [38, 39] was used for the genes detected with the Samβada analyses to identify the major biological processes that involved the identified genes (GO biological process) by considering significant clusters with a P value lower than 0.05.

##### *F*_ST_*analyses*

Each animal of the working dataset with a known GPS geographic location was assigned to a Köppen climate group (Tropical, Dry, Temperate, and Cold; [40]) through the website climate-data.org. Then, we applied the following filters: (i) for each climate group, only breeds with at least 10 animals allocated were considered, and (ii) if the same breed was in two or more groups only the animals that belonged to the groups of known breed origin were considered and the others were discarded. The assignment of each individual to a Köppen group and the subsequent filtering of animals provided a dataset of 1689 animals (141 for the Tropical group, 796 for the Dry group, 632 for the Temperate group and 120 for the Continental group), with no redundant breeds across the groups; assignments are summarized in Table 4. MDS (multidimensional scaling) of the filtered animals was performed using the Plink 1.9 software [41]. Single SNP *F*_ST_ was performed by comparing each group to the remaining groups merged together. The top 20 SNPs of each comparison, corresponding to the 0.9995 percentile of the distribution were considered and compared with the previous results of the landscape genomics analysis that had been filtered based on a significance threshold of 0.05 before Bonferroni correction. Only the selected *F*_ST_ SNPs with a G score or Wald score and β1 regression coefficient higher than 0.99 quantile were retained for further analysis. Allele frequencies of the SNPs that were shared by different groups were calculated and compared using the Plink 1.9 software [41].

**Table 4:** Breed composition and number of animals according to the Köppen group classification

Köppen group	Breed code	Breed name	Number
Tropical	CAM	*Cameroon goat*	11
	NAI	*Naine*	14
	WAD	*West African Dwarf*	15
	SEA	*Small East African*	16
	CAN	*Caninde*	23
	MOX	*Moxoto’*	23
	GUM	*Gumez*	39
Dry	CHA	*Chappar*	9
	KES	*Koh*-*e*-*sulmani*	13
	LOP	*Local Pothohari*	13
	MAU	*Maure*	13
	JAT	*Jattan*	15
	SAH	*Sahel*	15
	BAB	*Barbari*	16
	GUE	*Guera*	16
	THA	*Thari*	16
	LOH	*Lohri*	17
	MUG	*Murciano*-*Granadina*	17
	KAC	*Kachan*	19
	PAH	*Pahari*	19
	TAR	*Targui*	19
	DDP	*DDP*	20
	MTB	*Matebele*	22
	PEU	*Peulh*	22
	SDN	*Soudanaise*	22
	TAP	*Tapri*	22
	BRI	*Bari*	25
	PAT	*Pateri*	27
	BUT	*Bugituri*	31
	KAM	*Kamori*	38
	TED	*Teddi*	47
	ABR	*Abergelle*	49
	BRK	*Barki*	50
	OSS	*Oasis*	50
	RAN	*Rangeland*	50
	SID	*Saidi*	50
	NBN	*Nubian*	54
Temperate	BLB	*Bilberry*	10
	TOG	*Toggenburg*	10
	JON	*Jonica*	11
	MAL	*Mallorquina*	12
	MLS	*Maltese sarda*	12
	NRW	*Norwegian*	12
	PAL	*Palmera (Canaria breed)*	12
	LNR	*Landrace*	13
	OIG	*Old Irish Goat*	13
	MLT	*Maltese*	14
	BEY	*Bermeya*	15
	GAR	*Garganica*	15
	CCG	*Ciociara Grigia*	16
	MSH	*Mashona*	16
	PVC	*Provençale*	16
	ASP	*Aspromontana*	17
	NIC	*Nicastrese*	17
	DIT	*Di Teramo*	19
	TUN	*Tunisian*	21
	VAL	*Valdostana*	21
	ORO	*Orobica*	22
	VSS	*Valpassiria*	22
	GGT	*Girgentana*	23
	ARG	*Argentata*	24
	FSS	*Fosses*	24
	ANG	*Angora*	25
	PYR	*Pyrenean*	26
	PTV	*Poitevine*	27
	SAR	*Sarda*	27
	CRS	*Corse*	28
	MLG	*Malagueña*	29
	RME	*Rossa Mediterranea*	30
	BOE	*Boer*	33
Continental	CRP	*Carpathian*	10
	BIO	*Bionda dell’Adamello*	17
	SAA	*Saanen*	46
	ALP	*Alpine (Camosciata delle Alpi)*	47

## Results

### Selection of goat populations for the analysis of signatures of selection based on worldwide genetic diversity

**Fig. 1:**
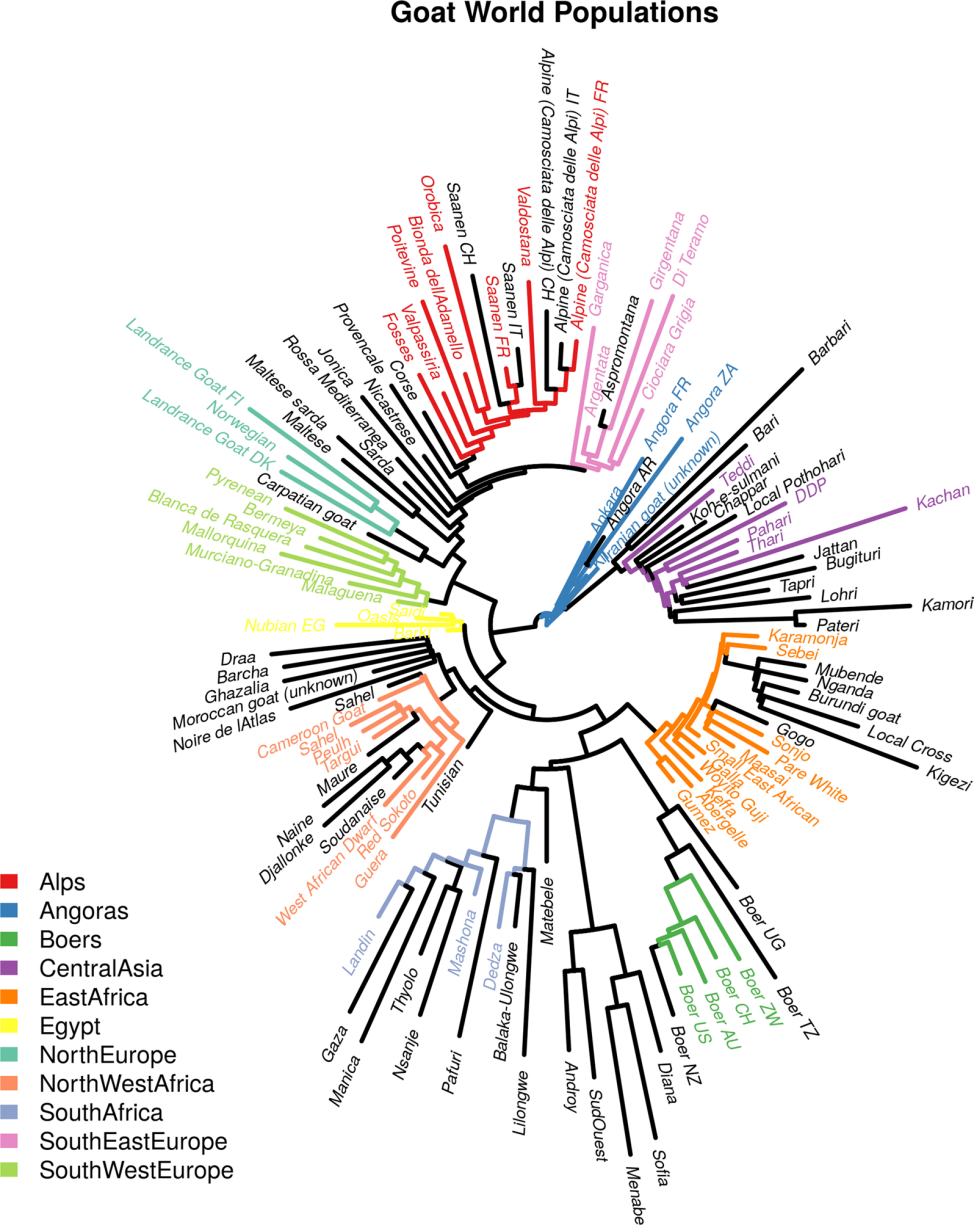
Populations used to detect signatures of selection. Populations are color-coded according to their identified geographical groups. Populations in black were not considered in the analyses signatures of selection (see details in the text)

A dataset of goat breeds ensuring robust modelling was developed prior to FLK and hapFLK analyses. This dataset included 61 breeds, which overall represented all the genetic diversity present in the complete dataset (Fig. 1). The genetic diversity in this set of breeds mirrored their geographical origin and was consistent with a radiation from the domestication center. Thus, each population was assigned to one of the 11 sub-continental groups, and each sub-continental group was analyzed independently. In Fig. 1, the root of the population tree, which is located at the center, corresponded to the ancestral population of all goat breeds, *i.e.* the ancestral domesticated population. Extant populations radiated from this ancestral population with populations that were geographically closer to the domestication center also usually genetically closer. The populations that were closest to the ancestor belonged to the “Angora” group, which originated from Turkey. A little further, in their own sub-group, there was a set of breeds from Pakistan (“Central Asia” group). The other two large sub-trees consisted of populations from Africa and Europe, which were split further into sub-continental groups.

#### Signatures of selection participating in the adaptive differentiation of goat populations

The FLK and hapFLK analyses detected 67 signatures of selection among the 10 population groups. Figures S2 and S3 (see Additional file 2: Figures S2 and S3) show the Manhattan plots for the FLK and hapFLK analyses and Figure S4 (see Additional file 2: Figure S4) shows the overview of the genomic distribution of all signatures of selection. The complete list of signatures of selection is in Table S2 (see Additional file 3: Table S2). Graphical representations of FLK, hapFLK, ROH and iHS signals for all signatures of selection are in Additional file 4. The northern European group was the only group for which no significant signatures of selection were detected. The populations that constitute this group display long terminal branches in the population tree (Fig. 1), which indicates that they have all experienced a strong reduction in population size. Such a reduction in size creates extensive genetic drift, which alone can explain the genetic differentiation of these populations. Hence, such extensive genetic drift makes the detection of signatures of selection difficult, which can explain the lack of power observed for the northern European group.

Most of the signatures detected were specific to one population. A total of 13 genomic regions were shared between at least two groups (see Additional file 3: Table S2). These regions were annotated by (i) listing the genes that were located ± 10 0 kb near the highest FLK or hapFLK signal and (ii) identifying the populations that showed an elevated ROH and/or iHS signal within the region.

A signature of selection on chromosome 5 between 30 and 40 Mb encompasses the *ADAMTS20* (*ADAM metallopeptidase with thrombospondin type 1 motif 20*) gene (see Additional file 2: Figure S5 and Additional file 4). This genomic region matched with a ROH signal in the Pyrenean goat population of the southwestern European group and with an iHS signal in the Argentata dell’Etna breed of the southeastern European group. It also matched with ROH and iHS signals in the Sahel, Peulh and Thari breeds of the northwestern Africa group. These breeds were genetically quite homogeneous and clustered within the same population in the genetic diversity study, thus, they were combined for the ROH analysis. An iHS signal was present in the Murciano-Granadina population, but did not match with the position of the *ADAMTS20* gene, thus it may represent another selection target.

A second signature selection on chromosome 6 harbors the *KIT* (*KIT proto*-*oncogene receptor tyrosine kinase*) gene in three population groups (see Additional file 2: Figure S6 and Additional file 4). A ROH signal was detected in the Kacchan breed of the central European group, the Abergelle breed of the east European group and the Argentata dell’Etna breed of the southeastern European group.

A third selection signature on chromosome 13 matched with the position of the *ASIP* (*Agouti signaling protein*) gene (see Additional file 2: Figure S7 and Additional file 4). In Pakistani breeds, a clear ROH signal, corresponding to a large fixed haplotype was detected in the Kacchan population sample from Pakistan and in several breeds of the Alpine group (Alpine, Poitevine and Valdostana). The most significant FLK signal in the Alpine group corresponded to the SNP that was closest to *ASIP*, however, the highest FLK signal in central Asian breeds did not correspond to the same SNP and the region spanned several genes.

Another relevant signal was detected on chromosome 1 between 110 and 130 Mb in the Alpine and southwestern European groups (see Additional file 2: Figure S8 and Additional file 4). This region seemed to match with an FLK signal in the Alpine Valdostana breed, which presents a high proportion of homozygous individuals for this region. An iHS signal also on chromosome 1, at ~ 108 Mb was found in the Ciociara Grigia Italian breed of the southeastern European group (see Additional file 2: Figure S26). Another signal was detected on chromosome 6 close to the cluster of casein genes *CSN1S1*, *CSN1S2* and *CSN2*, in the groups of Alpine breeds and Eastern African populations (see Additional file 2: Figure S9 and Additional file 4). The signature of selection detected for the Alpine populations clearly points to the casein genes while for the Eastern African populations, the FLK signals seem to point to a different cluster of genes that encode glucuronosyltransferase enzymes.

A complex signature of selection on chromosome 6 was identified between 25 and 50 Mb (see Additional file 2: Figure S10 and Additional file 4) and probably results from multiple signals in different genes and different groups. In the Egyptian group, the gene closest to the highest hapFLK signal was *LCORL* (*ligand dependent nuclear receptor corepressor like*). This region clearly matched with an extended region of shared ROH in the Nubian goat population from Egypt (see Additional file 4). Among the southwestern European populations, the Bermeya population presented an iHS signal at the same locus. This region in Egyptian populations overlaps with other signals detected in the southwestern and southeastern European groups. It contains the *ABCG2* (*ATP binding cassette subfamily G member 2*) gene and matched with an iHS signal in the Murciano-Granadina breed of the southwestern European group and in the Argentata dell’Etna breed of the southeastern European group. In both population groups, the hapFLK signal is much stronger than the FLK signal and no clear ROH signal was observed in the breeds of these two groups. A possible explanation for these two observations is that the signature of selection is due to a soft selective sweep rather than a hard sweep.

Another signature of selection was found on chromosome 12 in the southwestern European group and the central Asian group (see Additional file 2: Figure S11 and Additional file 4). This region contained the *RXFP2* (*relaxin/insulin like family peptide receptor 2*) gene. The two populations that showed a clear signature of selection in this region were the Thari goat from Central Asia (iHS signal) and the Blanca de Rasquera Spanish breed (ROH signal).

**Fig. 2:**
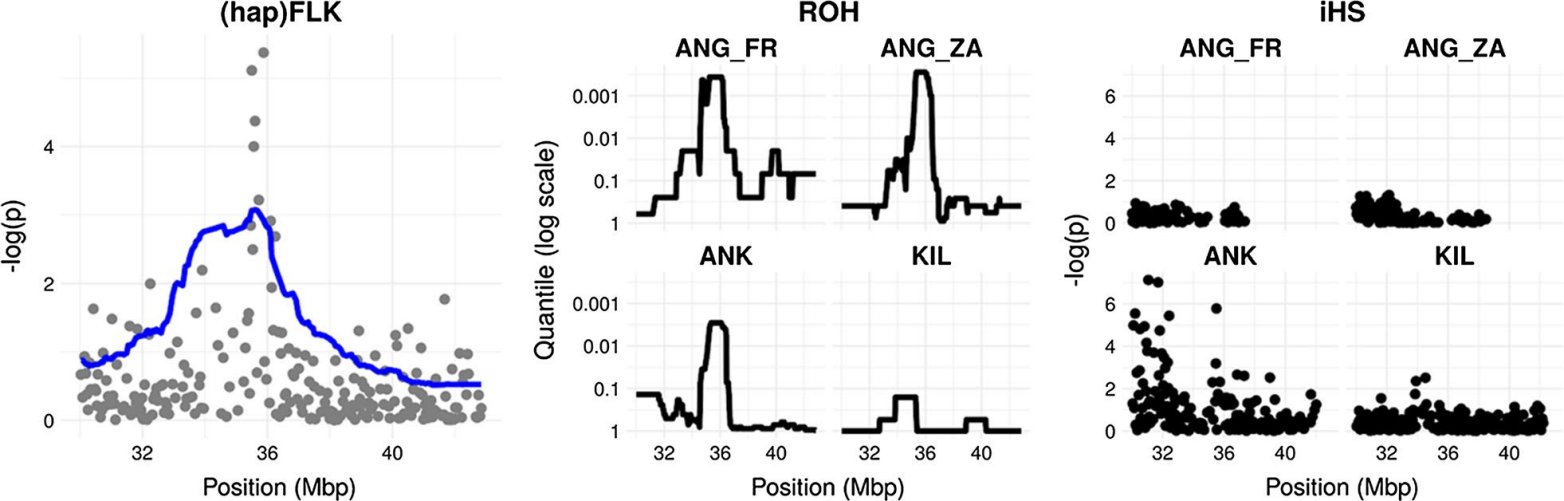
Signatures of selection on chromosome 25 in the Angora group. Left panel: FLK (points) and hapFLK (line) signals. Middle panel: ROH signals. Right panel: iHS signals

Finally, a region on chromosome 25 (35.50-35.88 Mb) was detected in the FLK analysis in the Angora group and confirmed by ROH and iHS analyses (Fig. 2).

#### Signatures of early adaptation

**Fig. 3:**
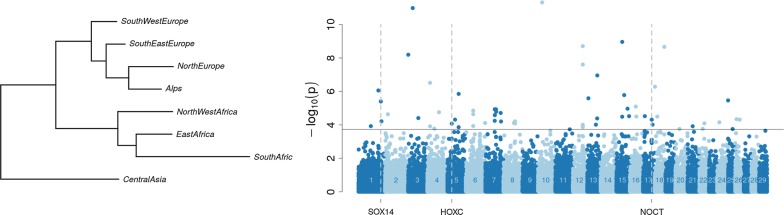
Genome scans for early adaptation based on differentiation between geographical groups. Left: Population tree built from the estimates of ancestral allele frequency in each continental group. Right: Manhattan plot of FLK p-values computed from the estimates of ancestral allele frequency and accounting for the ancestral tree structure

FLK analysis on the filtered geographical dataset based on the estimated ancestral allele frequency of geographical groups detected 62 SNPs with evidence for outlying differentiation among groups (Fig. 3) and (see Additional file 3: Table S3). The phylogenetic tree seemed to confirm the relationship between sub-continental blocks. Among the 62 SNPs, two signatures of selection were found on chromosome 1 close to the *SOX14* (*SRY*-*box 14*) gene and on chromosome 16 close to the *NOCT* (*nocturnin*) gene. Another signature of selection on chromosome 5 is located within the *HOXC* (*homeobox C*) gene cluster (see Additional file 3: Table S3).

#### Signatures of selection associated with external phenotypes and production traits

##### Production purposes

**Fig. 4:**
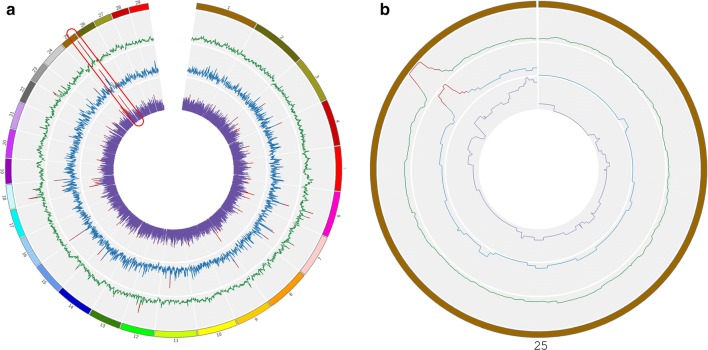
ROH, *F*_ST_ and XP-EHH for fiber (a), and detail of the ROH analyses on chromosome 25 for the breeds that compose the group of “fiber-producing” goat breeds: *Angora, Ankara* and *Cashmere* (b). (a) The three analyses are shown with different plot colors, within the most external squared-based circle, where each color represent a chromosome (chromosome number outside the squares): green (external) = ROH; blue (middle) = *F*_ST_; violet (internal): XP-EHH. For the three analyses, the regions above the threshold are marked in red. The region of high homozygosity (chromosome 25: 35,240,726-36,394,939 bp) is highlighted in red. (b) The three different breeds are labelled with different colors: green (external) = Angora; blue (middle) = Ankara; violet (internal): Cashmere. For the three breeds, the part corresponding to the 35-36 Mb region is marked in red when above the threshold

The analyses performed on the group of “fiber-producing” goat breeds detected 18 regions on 11 chromosomes with ROH, 88 regions and/or SNPs on 27 chromosomes with XP-EHH and 24 regions on 11 chromosomes with *F*_ST_ (Fig. 4). Among these, six regions on chromosomes 6, 18 and 25, including 34 genes, were detected by all three methods (see Additional file 3: Table S4). The region on chromosome 25 (34.69-36.43 Mb) showed the highest values in all the three analyses (Fig. 4a). Among the three breeds that compose the “fiber-producing” goat breed group, only Angora and Ankara showed a signature of selection in this region with none being detectable in Cashmere (Fig. 4b). This signature of selection overlapped with the regions detected by FLK/hapFLK, ROH and iHS analyses within the Angora group (Fig. 2) and contained 24 genes, including the *CUX1* (*cut like homeobox 1*) and the *PLOD3* (*procollagen*-*lysine,2*-*oxoglutarate 5*-*dioxygenase 3*) genes. The window-based *F*_ST_ analysis between Cashmere and Angora breeds pointed out several regions on chromosomes 2, 5, 6, 8, 9, 12, 13, 14, 17, 20, 25, 28 (data not shown). The ROH analyses on the Cashmere individuals highlighted several major regions on chromosomes 2, 10, 22 (47-48 Mb), 3, 5 and 8 (data not shown).

The analyses performed on the group of “meat-producing” goat breeds detected 25 regions on eight chromosomes with ROH, 121 regions and/or SNPs on 27 chromosomes with XP-EHH and 24 regions on 16 chromosomes with *F*_ST_ (see Additional file 2: Figure S12]. Among these regions, two regions on chromosomes 3 and 18 were detected by at least two of the three methods and contained 18 genes [see Additional file 3: Table S5), including *TSHB* (*thyroid stimulating hormone beta*), *NRAS* (*neuroblastoma RAS viral oncogene homolog*) and *AMPD1* (*adenosine monophosphate deaminase 1*), all on chromosome 3.

The analyses performed for the group of “milk-producing” goat breeds detected 11 regions on eight chromosomes with ROH, 286 regions and/or SNPs in all autosomes with XP-EHH and 24 regions on 15 chromosomes with *F*_ST_ (see Additional file 2: Figure S13). Among these regions, four were on chromosomes 11, 13 and 14, which included 20 genes that were detected by at least two of the three methods (see Additional file 3: Table S6), among which the *EFEMP1* (*EGF containing fibulin like extracellular matrix protein 1*) gene on chromosome 11. The ROH analysis revealed a region on chromosome 6 (75-120 Mb) that contains the cluster of casein genes, although it was not confirmed by the *F*_ST_ and XP-EHH analyses (data not shown).

The CDA, which was performed on the chromosomal regions surrounding the genes previously identified for the three groups, confirmed the results found in previous analyses. The region on chromosome 25 (34.69-36.43 Mb) that contained the *CUX1* and *PLOD3* genes could separate individuals in the group of “fiber-producing” goat breeds from the other two groups on the CAN1 variable, and the SNPs that best separate the three groups were in the middle of these regions (see Additional file 2: Figure S14). The region on chromosome 3 that included the *TSHB, NRAS* and *AMPD1* genes could separate the group of “meat-producing” goat breeds from the remaining two groups on the CAN2 variable when considering the highest peak in the middle of the region (see Additional file 2: Figure S15). Finally, the region on chromosome 6 that contains the casein cluster could separate the “milk-producing” breeds from the other two groups on the CAN1 variable, with a high peak near to the *CSN1S1* (*alpha S1 casein*) gene (see Additional file 2: Figure S16).

##### Coat color

The distribution on the MDS plot of the single breeds using the medium-density SNP chip overlapped partially between the three groups of coat color. This probably reflects geography rather than coat color, with the Middle Eastern, Asian (Pakistan) and African goats being separated from all European goats on the first component (see Additional file 2: Figure S17). The Angora and Ankara (White) breeds and the Kil and Kilis (Black) breeds, which all originate from Turkey formed two clusters. For the “coat color” groups, we detected regions on chromosomes 5 (70.0–70.5 Mb), 13 (53.0–53.5 Mb), and 18 (15.50–16.25 Mb) in the Black vs. White comparison, regions on chromosomes 5 (36.25–36.75 Mb), 9 (11.5–12 Mb), and 13 (53–53.5 Mb and 62.75–63.25 Mb) in the White vs. Black and Red comparison, and regions on chromosomes 8 (27.0–27.5 Mb), 22 (2.25–3.0 Mb) and 29 (39.25–39.75 Mb) in the Red vs. White comparison. The list of genes within these regions is in Table S7 (see Additional file 3: Table S7) and includes *ADAMTS20* and *TIMP3* (*TIMP metallopeptidase inhibitor 3*) on chromosome 5, *SOX18* (*SRY*-*box 18*) and *ASIP* on chromosome 13, and *MC1R* (*melanocortin 1 receptor*) on chromosome 18. CDA on the *MC1R* gene showed that SNPs present on either side of this gene could distinguish the solid Red goats from the solid Black and White animals that were close on the CAN1 (see Additional file 2: Figure S18), which disagrees with the above result that indicated that *MC1R* could separate black and white individuals. Similarly, SNPs located near the *ASIP* gene seemed to be able to distinguish the solid Red from the solid black and white groups (see Additional file 2: Figure S19). In the CDA, SNPs in the region surrounding the *ADAMTS20* gene could distinguish and separate all three groups on the CAN1 variable, and particularly the white group (see Additional file 2: Figures S20).

### Adaptation to environment

#### Landscape genomics analysis

**Fig. 5:**
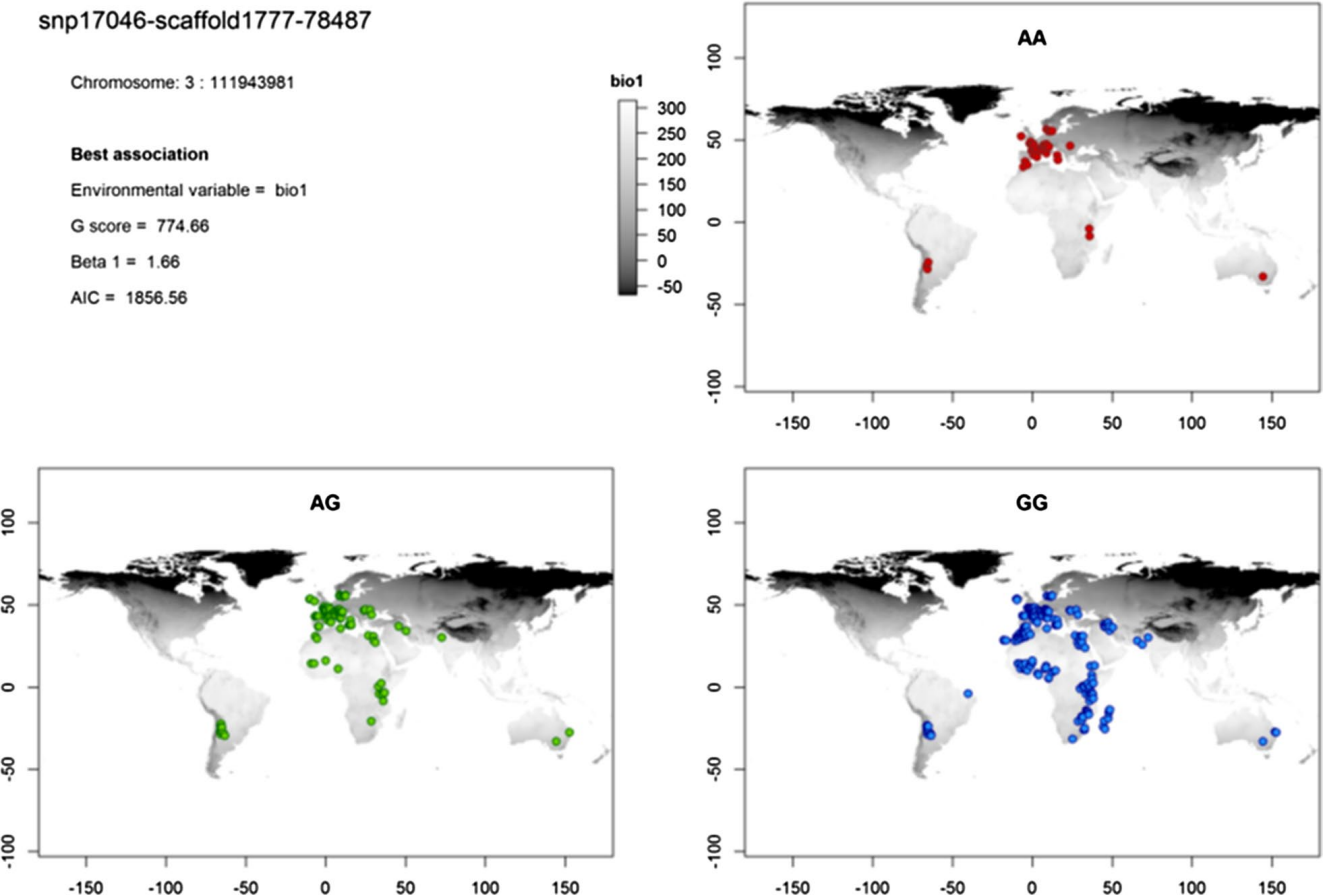
Map of the worldwide distribution of genotypes for the snp24965-scaffold2564-131990 (3:1091508)

The Samβada results showed that more than 80% of the SNPs appear in at least one significant association between a genotype and a bioclimatic variable. In addition, 57 SNPs were involved in associations that respected the second series of filtering criteria (values for the β1 coefficient, G score, Wald score > 0.99 quantile, AIC criterion < 0.1 quantile, and at least two genotypes associated with at least one environmental variable) (see Additional file 3: Table S8). The most significant associations (highest G score) obtained with these SNPs involved the environmental variable related to annual mean temperature (bio1) for 49 SNPs, mean temperature of the coldest quarter (bio11) for three SNPs, mean diurnal range (bio2) for three SNPs, precipitation of the driest month (bio14) for one SNP and isothermality (bio3) for one SNP. The results obtained with the sampling point variables were very similar to those obtained with mode, maximum, mean and median values computed within the buffer area, whereas the associations with range, minimum and standard deviation were less significant. One of the strongest associations was observed between the *CC* genotype of SNP snp24965-scaffold2564-131990 located on chromosome 3:1091508 and the mean annual temperature (bio1). This association had the highest G and Wald scores of the models filtered using the criteria above and the highest Efron score of all significant models (using a threshold 0.05 before Bonferroni correction). The spatial distribution of the genotypes for this SNP showed that the *AA* genotype is only present in Europe or in southern regions of high altitude. The *AC* genotype showed a similar trend, even if it is slightly more frequent in the southern regions, while the *CC* genotype was observed in the whole area of study (Fig. 5). This SNP is located close to the *pre*-*B cell leukemia homeobox 1* (*PBX1*) gene. The list of genes that are located near (± 100 kb) each of the 57 SNPs is in Additional file 3: Table S9 and the geographical distribution of the genotypes for the remaining 56 SNPs is in the Additional file 5. Analysis of the biological processes of the genes located in the vicinity of these SNPs (see Additional file 3: Table S10) highlighted genes that are linked to several pathways, such as the insulin and glucose signaling pathway and metabolism [(*IGF2* (*insulin*-*like growth factor 2*)], glycogen metabolism, lipid biosynthetic processes, oxidative stress [*GPR37L1* (*prosaposin receptor GPR37L1*) and *INS* (*insulin*) genes] and regulation of vasodilation. Two genes are involved in circadian rhythms regulation: *RAI1* (*retinoic acid induced 1*) and *TH* (*tyrosine hydroxylase*).

#### *F*_ST_*and landscape genomic analyses of climatic associations*

**Fig. 6:**
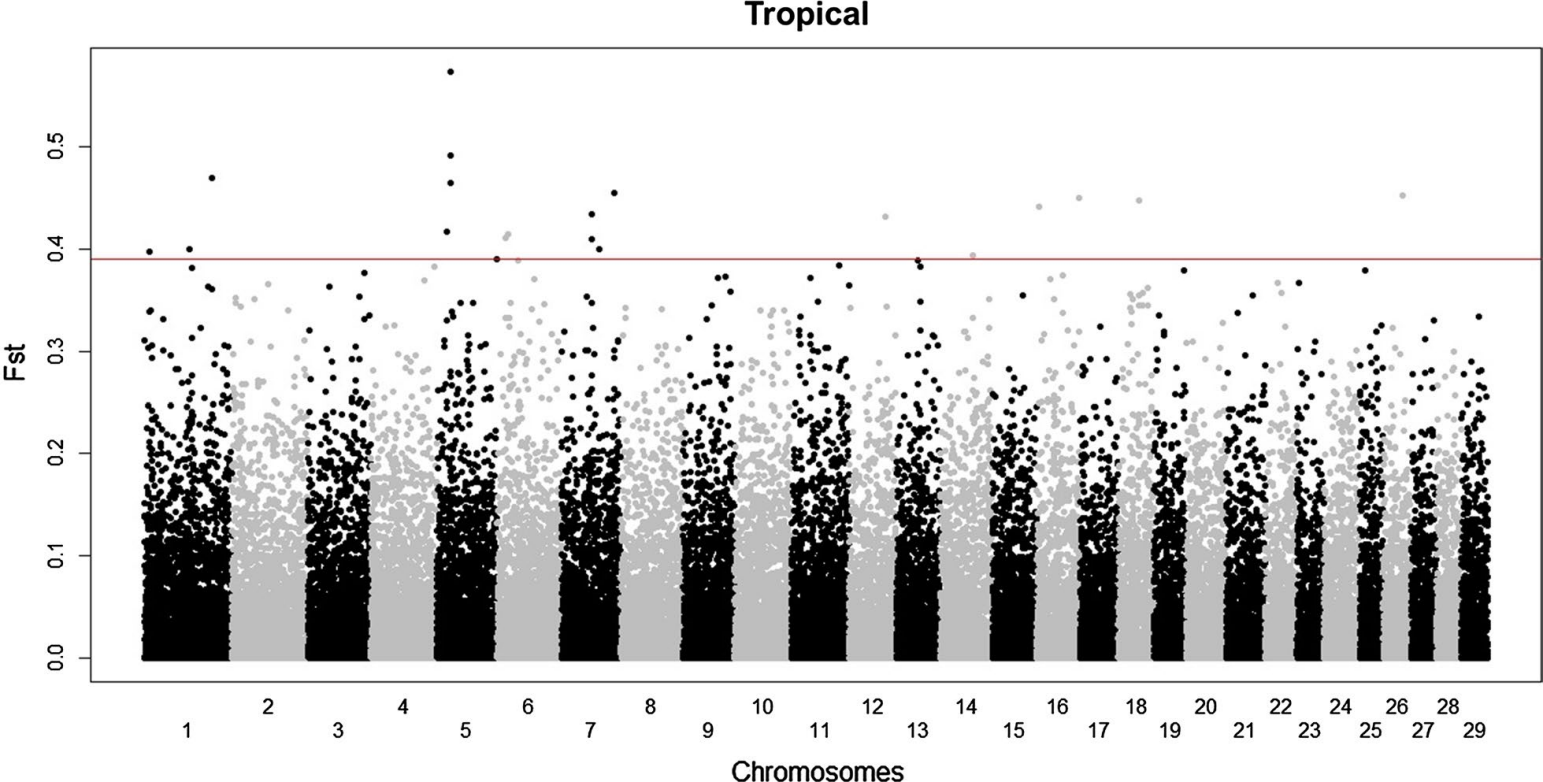
*F*_ST_ plot of the comparison of the Tropical group vs. the other groups. The threshold line in red represents the 0.995 of the percentile distribution (*F*_ST_ = 0.391)

The MDS analysis (see Additional file 2: Figure S21) showed an overlap between the Köppen climate groups, with Tropical and Continental being the smallest and most clustered groups. The *F*_ST_ plots are in Fig. 6 and Figures S22 to S24 (see Additional file 2: Figures S22, S23 and S24) and indicate for each analysis the number of SNPs above the selected threshold. Twenty SNPs for each comparison met the threshold of the quantile > 0.9995, among which, 13 are shared between the Dry and Temperate/Continental groups with opposite major alleles between these groups (see Additional file 3: Table S11). Interestingly, the Tropical group did not have any common SNPs with the other groups, which reduced the list from 80 to 65 SNPs. When these 65 SNPs were compared with the Samβada results, they all showed either high G scores, Wald scores or β1 regression coefficients (quantile values considering only those obtained from the significant models > 0.99 of the empirical distribution) for at least one genotype in several environmental variables. These SNPs are summarized in Table S12 (see Additional file 3: Table S12). In the landscape genomic analysis, nine of these SNPs showed G scores, Wald scores and β1 regression coefficients that were all higher than the 0.999 quantile. In total, 197 genes were detected in the regions (± 100 kb) close to these 65 SNPs (see Additional file 3: Table S13). For the Tropical group, a cluster of *HOXC* genes (*HOXC4, HOXC5, HOXC6, HOXC8, HOXC9, HOXC10, HOXC11, HOXC12* and *HOXC13*) that are located near the three SNPs on chromosome 5 was detected. Analysis of the genes for the four Köppen climate groups revealed several genes such as *SOX17* (*transcription factor SOX*-*17*) for the Dry and Temperate groups, *CLYBL* (*citrate lyase subunit beta*-*like protein*) for the Dry and Continental groups, *LYPLA1* (*lysophospholipase 1*), *ATP6V1H* (*vacuolar ATPase*) and *RGS20* (*regulator of G*-*protein signaling 20*) for the Dry and Temperate groups and, with a different SNP, also in the Tropical group, *CAPN10* (*calpain 10*) and *RNPEPL1* (*arginyl aminopeptidase* (*aminopeptidase B)*-*like 1*) were found for the Continental group only but also detected in the landscape genomic analyses and in the FLK analyses for signature of early adaptation.

## Discussion

Detection of signatures of selection from genotype data alone is possible either by searching for genomic regions that show high levels of differentiation between populations or by looking for regions of low genetic diversity within a population. In this work, both approaches were used because they detect different kinds of selection events. Selection events that lead to the rapid fixation of an initially rare variant, i.e. hard sweeps, should lead to signals that can be detected by both approaches. However, in this case, within-population methods are more powerful than differentiation-based methods, but differentiation-based approaches can detect a greater diversity of selection signals, such as selection on standing variation (soft sweeps) or diversifying selection.

Our analyses were not limited to searching for signals of selection within or among populations. We also exploited other available information, such as differences in production purposes, phenotypes, and geographical/bioclimatic coordinates to elucidate the mechanisms of selection. The detection of adaptive loci in the genome is an essential part of studies on environmental adaptation, since it can help understanding which regions of a genome and therefore which genes have been or are being shaped by natural selection. Spatial analysis with geographical information systems (GIS) and environmental variables, along with molecular data, were included in the landscape genomic analysis to uncover the genomic regions under selection and identify candidate environmental factors that cause this selection. Multiple univariate logistic regressions were carried out to test for association between allele frequencies at marker loci and environmental variables. The analyses considered variables that can be summarized by differences in temperature and precipitation, which could be indirectly correlated to water availability. The Samβada analyses showed that more significant associations and more selected SNPs were associated with differences in temperature than with differences in precipitation. Therefore, temperature appears to have played a bigger role in the adaptation processes of goat breeds.

In addition, our analyses examined the signals that were detected in the genome after a detailed modeling of genetic divergence and population structure. Complex admixture and strong population bottlenecks can mimic the effect of selection by reducing local variability considerably. Therefore, to detect these signals, we applied the FLK and hapFLK approaches to individuals with a unique genetic background, without recent admixture or population bottlenecks. Finally, signatures of selection left by recent human selection on goat populations that are linked to production purposes or phenotypic standardization were analyzed.

Taken together, these analyses contributed to provide a genome-wide picture of the genes and genomic regions that have been subject to selection and led to signatures of selection that concern different biological pathways or group of genes, which were detected by one or more types of analyses.

### Genes related to milk production

Among all the genes that can contribute to milk yield, quality and processing, the casein genes are one of the most important gene families in ruminants, since they have a major role in the production of cheese products from milk. In the goat genome, this gene cluster is located on chromosome 6 between 85.95 and 86.25 Mb. Analysis of the ROH scores of the breeds selected for milking purposes showed that the longest region with loss of variability coincides with the casein region on chromosome 6 between 85.9 and 86.2 Mb. The goat breeds used for milk and cheese production belong mainly to the European breeds, particularly from Italy and France, which suggests that the signals identified are related to a common direction of selection within these breeds, i.e. that they are used mainly to produce whole milk (20% of production) and cheeses (80% of production) [42]. Many dairy goat breeds of Europe have been developed through breeding with the two most specialized dairy breeds, i.e. Saanen and Alpine from Switzerland [21]. Due to its popularity as a dairy breed, Saanen goats are distributed across more than 80 countries worldwide [5]. The genomic region that contains the cluster of casein genes was also detected by FLK analysis, which identified a region containing this gene cluster in the group of Alpine breeds and a region close to this cluster in the group of Eastern African populations. The different analyses and particularly the FLK and hapFLK analyses also detected other regions related to milk production. Chromosome 6 carries several genes that are related to milk production and found in several ruminant species. Another region on chromosome 6 that contains the *ABCG2* gene was detected in the Egyptian, southwestern and southeastern European groups, which was further confirmed by the iHS signal detected for the Murciano-Granadina and Argentata dell’Etna breeds. The *ABCG2* gene matched with a previously identified signature of selection in sheep [22] and polymorphisms in this gene have been associated with milk yield and composition in cattle and sheep [43, 44]. The ROH/Fst/XP_EHH analyses detected another consensus region on chromosome 11 that contains the *EFEMP1* gene, which is associated with conjugated linoleic lipid contents in the meat of Wagyu Angus cattle breed [45]. A region on chromosome 1 between 110 and 130 Mb that was detected in the Alpine and southwestern European groups is homologous to a genomic region in dairy cattle for which selective pressure was previously reported [46]. A separate signature of selection approximately 5 Mb away from this region was also found in the Italian local breed Ciociara Grigia, which is used for milk production. Brito et al. [13] recently identified other genomic regions that may be associated with milk production, using a partially overlapping subset of breeds [13]. This difference may be due to a different breed composition of the dataset used and underlines the fact that there may be some breed-specific regions related to milk composition. Therefore, more tailored experiments are needed to decipher the genetics behind milk production in goat.

### Genes related to fiber production

For the group of “fiber-producing” goat breeds, the strongest signature of selection in all three types of analyses (ROH, XP-EHH and *F*_ST_) was detected in the genomic regions containing the *CUX1* and *PLOD3* genes. The detected regions in Angora and Ankara were confirmed by the FLK and hapFLK analyses. These genes are associated with hair development: *CUX1* is associated with wavy hairs and curly whiskers in mouse [47] and *PLOD3* may play a role in the formation of hairs or in their texture [48]. The roles of these genes are consistent with the presence of a signature of selection only in the two Angora breeds (Angora and Ankara), which have curly hairs, and not in the Cashmere breed, which has long and wavy hairs. For the Cashmere breed, Brito et al. [13] proposed several alternative genomic regions. The *F*_ST_ analyses comparing the Cashmere vs. the Angora and Ankara breeds that revealed a signature of selection on chromosome 25 did not show any overlapping regions with those reported in [13], probably because the approach used in [13] highlighted other genomic regions. The ROH analyses carried out on the data for the Cashmere breed revealed a region on chromosome 22 that overlapped with one of the regions reported by Brito et al. [13] and was identified by a *F*_ST_ analysis of Cashmere against several other breeds. Thus, this may be an interesting region to investigate further for fiber production in the Cashmere breed. In addition, it suggests that different genomic regions are involved with fiber production in Cashmere and Angora/Ankara breeds.

### Genes related to meat production

In the analyses comparing breeds for meat production, some of the detected regions contain genes associated with muscle formation. On chromosome 3, we identified a region that includes the *AMPD1* and *NRAS* genes, in agreement with previous findings in cattle and mice. *AMPD1* is involved in the deamination of AMP in skeletal muscles [49] and its disruption was reported to influence the expression of neighboring genes, such as *NRAS*, in mice [50]. In addition, allelic variants of the *AMPD1* gene are associated with traits such as heart girth and body weight in Chinese beef cattle [51]. Another gene within the same region, *TSHB* may also be associated with muscular functionality. *TSHB* encodes a subunit of the thyroid stimulating hormone (TSH), which plays a pivotal role in regulating thyroid activity, stimulating colloid reabsorption and in the release of the T_4_ hormone [52]. The role of thyroid function in growth is known, and different levels of TSH are associated with hyperthyroidism or hypothyroidism [53]. In fact, high levels of TSH are commonly found in obese human children and adolescents [54]. In goat, this gene has been characterized in relation with reproductive seasonality [55].

The main breed of the “meat-producing” group is the Boer breed, which is selected for meat production and for which several breeding programs have been developed. In spite of this, no evidence of strong selection in the Boer breed for genes related to meat production was detected in our analyses. A possibility is that variation in meat production traits in this population may have a highly polygenic basis (many alleles of small effects determine the trait). Because of the size of the samples and the availability of phenotype records, we used approaches that are not suited to detect such selection events. Other targeted studies on the Boer breed are needed to evaluate the possible genomic impact of breeding programs in this population.

### Genes related to coat color

ROH analysis of the data for the three groups of solid coat colors detected signatures of selection near at least five genes that are known to be involved in the color and pattern definition of the coat: *ADAMTS20, MC1R*, *ASIP*, *SOX18* and *TIMP3*. Two of these genes (*MC1R* and *SOX18*) were specifically detected in the comparison between solid Black and solid White individuals, whereas the remaining three were identified in both solid Black vs. solid White and solid Black and Red vs. solid White groups.

The well-known *MC1R* gene is involved in the genetic determinism of color [56] and located on chromosome 18 in goats. It plays a major role in controlling the switch from eumelanin (black and brown) to pheomelanin (yellow to red; [57]) produced by melanocytes. This gene has already been studied in several species [58], such as cat [59], horse [60], dog [61], pig [62], cattle [63], sheep [64] and even in goat [65–68]. Although described in other species, no direct association between *MC1R* and red coat color is known in goat.

*ASIP* is a competitive antagonist of *MSH* (*melanocyte stimulating hormone*) for binding MC1R, enabling the switch from eumelanin to pheomelanin [69]. Similarly to what we observed for SNPs within the region surrounding *MC1R*, SNPs within the region around *ASIP* seemed to be able to distinguish the solid Red from the solid Black and White groups. The presence of an association with the Red/Black group in the region surrounding this gene seems to confirm its role also in goats, as hypothesized in several studies [11, 70]. Moreover, the signal on this gene was also found with the differentiation tests (FLK, hapFLK) in Alpine, Poitevine and Valdostana goats with black-and-tan pigmentation pattern. This further confirms the potential role of this gene in modulating the melanocyte activity in goats, in particular its involvement in the switch from black to red pigments, whereas the white color may to be caused by other gene(s).

The third candidate gene identified is *SOX18*, which encodes a transcription factor that plays an important role in hair follicles and blood vessel development during embryogenesis in disheveled mice, a semi-dominant mutation characterized by coat sparseness [71, 72]. In addition, alleles of this gene seem to be responsible for a dark phenotype in mice [57, 73].

*ADAMTS20* located on chromosome 5 encodes a highly conserved metalloprotease. This gene is required for melanoblast survival and mediating Kit signaling in skin colonization such as the belted white locus in inbred mouse colonies [74–76]. It is also important in multiple biological functions, such as delayed palate closure [77] and soft tissue syndactyly [78]. Another gene detected with these approaches, *TIMP3*, is known to inhibit the activity of metalloproteinases, and shows high specificity and selectivity for the ADAM and ADAMTS families [79] that are involved in melanoma cancerous cell development [80]. A signature of selection that included this gene was also found in the Southwestern European goat group and in particular in the Pyrenean goat population, which is characterized by a piebald black and white pattern, in the Argentata dell’Etna breed, which is characterized by a silver coat colour, and in the group of breeds from Northwestern Africa where Sahel, Peulh and Thari breeds are characterized by white or white spotted coat colours. However, for the last three breeds, no reliable pictures were available to evaluate the coat color pattern.

The signatures of selection near the *KIT* gene observed in the Kacchan and Abergelle breeds could be related to their spotted phenotypes (in this case, this phenotype is localized mainly on the anterior parts of the animals) as already observed in other species. It should be noted that the Argentata dell’Etna breed has a silver phenotype and that, in the fox, an analogous phenotype is caused by a mutation on an autosomal copy of the *KIT* gene [81].

### Genes related to other traits

In this work, a strong association was found between the results of the landscape genomics analyses and those obtained using independent analyses based on other types of more categorical classifications, such as the Köppen classification. Several signals were associated with environmental parameters and these loci behave atypically in comparison with the theoretical distribution for neutral loci. Among the 13 common SNPs that differentiated the Dry and Temperate/Continental groups, i.e. between hot-dry and temperate-cold areas, all showed a different major allele, and this may be the sign of natural selection that is driving alleles in opposite directions for the adaptation of the breeds to different environments. These SNPs were also confirmed by landscape genomics analyses, i.e. although they are not included in the top 57 SNPs, they displayed a high significance for one or more bioclimatic variables. Several of the genes around these 13 SNPs are associated with production traits in cattle. *LYPLA1* encodes a hormone that acts as a ghrelin inhibitor and is, therefore, involved in the regulation of the appetite, as shown in rats [82]. This gene is also associated with feed intake and weight gain in cattle [83]. Other genes, such as *RGS20* and *SOX17* may play a role in pubertal development, as shown in Brahman cattle [84]. Regarding *CLYBL*, it was shown to be differentially expressed in cows with different milk citrate contents [85]. A region detected by FLK and landscape genomics and which is unique in the Continental group through *F*_ST_ analysis contains the *CAPN10* and *RNPEPL1* genes. Calpains are calcium-regulated proteases involved in cellular functions that include muscle proteolysis both ante- and postmortem. In livestock, they play important roles in muscle growth and development, myoblast fusion, and differentiation [86]. The early post-mortem cleavage of these proteins leads to the tenderization of meat and, thus, *calpain* genes have been associated with meat tenderness [87, 88]. The *RNPEPL1* gene is differentially expressed in pigs with different muscularity traits [89]. FLK analyses detected the *RXFP2* gene in the Southwestern European group and the central Asian group, particularly in the Thari and Blanca de Rasquera breeds. This well-known gene is responsible for the polled condition in most domestic sheep populations and associated with horn phenotypes in the Soay breed, a feral archaic sheep from Scottish islands, but it has not been shown to be involved in the development of horns in goats [22]. This signature of selection suggests that the genetic determinism of horns in these two goat populations may be specific compared to other goat breeds.

The *LCORL* gene was present in a signature of selection detected in the Egyptian group, particularly in the Nubian breed. It has been associated with signatures of selection linked to animal size in cattle [90, 91] and in European commercial and local pig breeds [92].

### Insulin, glucose pathway and oxidative stress response genes

Temperature variation detected by the Samβada analyses can operate as a primary environmental stressor. A major cellular effect caused by multiple environmental stressors is the generation of reactive oxygen species (ROS) that leads to oxidative stress. This was reported in livestock species such as cattle and chickens. In goats, it could be supported by genes that are involved in pathways linked to temperature changes, such as insulin or glucose pathways and oxidative stress response. The *GPR37L1* gene is primarily expressed in neuronal cells and has a role in protecting primary astrocytes against oxidative stress [93]. Another gene related to oxidative stress is the *INS* gene, its level in pancreatic cells changing in case of oxidative stress [94], which stimulates the *HSP* (*heat shock protein*) gene in cardiac tissue [95]. The *INS* gene is also the target of other important pathways related to insulin production, response of the organism to insulin and glucose metabolism. These biological processes are the most predominant in the landscape analyses. Heat stress can also affect the response of an organism to insulin stimulus, which in turn affects lipid and carbohydrate pathways [96, 97]. Several of the genes involved in pathways related to temperature changes were close to SNPs identified in our analyses and are associated with production traits in several species. For example, *IGF2* that is present in pathways related to insulin, glucose, glycogen, lipid, and carbohydrate processes is one of the most important genes for meat production and heat stress response in several species [98, 99].

### *HOXC* genes family and related genes

Three SNPs located on chromosome 5 were detected by *F*_ST_ analyses in the Tropical group. SNPs with the highest *F*_ST_ value were included in the *HOXC* cluster, and in the landscape genomics analysis they were associated with the bioclimatic variables defined as “isothermality” and “temperature seasonality”. A genome scan between geographical groups to detect signals of early adaptation identified the same region. The *HOX* gene family (*HOXA*, *HOXB*, *HOXC* and *HOXD* clusters) controls the body plan of the embryo along the craniocaudal (head–tail) axis. The *HOX* gene family was recently associated with mating/sexual behavior in Drosophila, *C. elegans* and mice [100, 101]. Selective pressure on some *HOX* genes was also detected in bats and aquatic mammals [102, 103]. Recently, a signature of selection near the *HOXA* and *HOXC* gene clusters was also reported in sheep [22]. One of the strongest associations in the Samβada analysis was detected close to the *PBX1* gene, which is involved in the morphological development of several parts of the body and tissues. *PBX1* interacts with *HOX* gene clusters, which includes the *HOXC* gene [104, 105]. It is interesting to note that most of the breeds assigned to the Tropical group were characterized by a smaller size (e.g. Cameroon goat, Naine, Small east Africa, and African Dwarf). It has been reported that that the small body size of Zebu cattle in tropical areas can contribute to heat tolerance, since small animals have a higher surface to body mass ratio, together with the fact that small size correlates also with low nutritional requirements [106]. These observations may apply also to goats and such genes can determine an adaptation towards a smaller size and therefore better heat management in areas where hot temperatures are constant.

### Genes linked to circadian clock

The genome scan for signatures of selection between geographical groups and the landscape genomics analysis detected several genes that are linked to circadian clock rhythm suggesting a possible association with adaptation at different latitudes. These included *SOX14*, *NOCT*, *RAI1* and *TH*. *SOX14* is required to drive the development of a functional network supporting light-circadian behaviors [107]. *NOCT* encodes a circadian deadenylase expressed at high levels during the night in several mammalian tissues and was recently implicated in circadian control of metabolism [108]. *RAI1* has an important function in the maintenance of circadian rhythmicity. In humans, it is crucial for normal regulation of circadian rhythms, lipid metabolism and neurotransmitter function [109]. This gene disrupts the transcription of the *CLOCK* gene, a key component of the mammalian circadian oscillator that regulates many other critical genes involved in metabolism, physiology, and behavior. A chromosomal deletion of *RAI1* in mice is associated with a short circadian period, whereas in humans it is associated with the Smith-Magenis syndrome, a pathology that is characterized by an inverted melatonin rhythm, sleep disturbances, abnormal feeding, and cognitive disturbance [110, 111]. Finally, *TH* is involved in the production of tyrosine hydroxylase, which is important for the nervous system to function normally. Tyrosine hydroxylase is the rate-limiting enzyme in the synthesis of catecholamine, and hence plays a key role in the physiology of adrenergic neurons and receptors. It is involved in the production of neurotransmitters that control physical movements and involuntary body processes, such as the regulation of blood pressure and body temperature. Moreover, in the response to photoperiod, it is well known that tyrosine hydroxylase neurons have estrogen receptors that are involved in the physiological cyclic activity induced by photoperiod [112].

Another gene that could be linked to the reproductive seasonality is *TSHB*, which has been reported to be associated with meat production and reproductive seasonality in goats [55].

## Conclusions

The goat is one of the most adaptable livestock species distributed worldwide across a large variety of climatic and geographical areas and used by humans for different purposes. Natural and artificial pressures have led to different genomic signatures of selection across the genomes of many goat breeds. By applying different kinds of analyses, we were able to detect (1) different allelic distributions worldwide that are associated with bioclimatic variables and groups, (2) signatures of selection that differentiate breeds raised for different economical purposes, and (3) population structure clustering that represent sub-continental groups We were also able to identify several genomic regions that contain genes related to processes such as milk-, meat- or fiber-related production, coat color, glucose pathway, oxidative stress response, size and the internal circadian clock. These results provide a first comprehensive picture into the global domestication process and adaptation of the goat and highlights several genes that have contributed to the differentiation of this species worldwide.

## Additional files

**Additional file 1.**Treemix plots. Each page shows the output of a continental/sub-continental group

**Additional file 2: Figure S1.** MDS plot of the breeds, grouped by production purpose: milk, meat and fiber groups. Group colors: milk = green, meat = red, fiber = blue; MDS and box plots of the first two components pre-filtering (upper) and MDS plots after filtering. **Figure S2.** Manhattan plot of the FLK results for the sub-continental group after filtering steps. Sub-geographical group names are given on top of each plot. Chromosomes are alternately red and black. **Figure S3.** Manhattan plot of the hapFLK results for the sub-continental group after filtering steps. Sub-geographical group names are given on top of each plot. Chromosomes are alternately red and black. **Figure S4.** Genomic distribution of FLK and hapFLK signals across population groups. For each chromosome and each sub-continental group, the chromosomal position detected with at least one of the two approaches is indicated. **Figure S5.** Signatures of selection on chromosome 5 for the North western Africa, South eastern Africa and South western Africa groups. North western Africa (NWA): red; South eastern Europe (SEE): green; South western Europe (SWE): blue. The table (bottom-right) reports the genes within the region in which a signature was detected. **Figure S6.** Signatures of selection on chromosome 6 for the Central Asia, East Africa and South east Europe groups. Central Asia (CA): red; East Africa (EA): green; South east Europe (SEE): blue. The table (upper-right) reports the genes within the region in which a signature was detected. **Figure S7**. Signatures of selection on chromosome 13 for the Alpines and Central Asia groups. Alpines (Alps): red; Central Asia (CA): blue. The table (right) reports the genes within the region in which a signature was detected. **Figure S8.** Signatures of selection on chromosome 1 for the Alpines and South Western Europe groups. Alpines (Alps); red; South Western Europe (SWE): blue. The table (bottom-right) reports the genes within the region in which a signature was detected. **Figure S9.** FLK signals on chromosome 6 around the *casein* gene cluster. The cluster of unannotated genes between *YTHDC1* and *SULT1B1* consists of genes coding for glucuronosyltransferase enzymes. Alps; red; East Arica (EA): blue. The table (right) reports the genes within the casein cluster region. **Figure S10.** Signatures of selection on chromosome 6 for the Egypt, South Eastern Europe and South Western Europe groups. Egypt (Egypt): red; South Eastern Europe (SEE): green; South Western Europe (SWE): blue. The table (bottom-right) reports the genes within the region in which a signature was detected. **Figure S11.** Signatures of selection on chromosome 12 for the Central Asia and South Western Europe groups. Central Asia (CA); red; South Western Europe (SWE): blue. The table (right) reports the genes within the region in which a signature was detected. **Figure S12.** ROH, *F*_ST_ and XP-EHH results for the group of “meat-producing” goat breeds. Types of analysis are indicated with different plot colors, within the most external squared-based circle, where each color represent a chromosome (chromosome number outside the squares): green (external) = ROH; blue (middle) = *F*st; violet (internal): XP-EHH. For the three analyses, the regions above the threshold are marked in red. **Figure S13.** ROH, *F*_ST_ and XP-EHH results for the group of “milk-producing” goat breeds. Types of analysis are indicated with different plot colors, within the most external squared-based circle, where each color represent a chromosome (chromosome number outside the squares): green (external) = ROH; blue (middle) = *F*st; violet (internal): XP-EHH. For the three analyses, the regions above the threshold are marked in red. **Figure S14.** CDA for the region on chromosome 25 detected for the group of “fiber-producing” goat breeds. (a): LONG and (b) SHORT: (b). Left: CDA plot. Right: Correlation value of the SNPs used for the analyses for CAN1 and CAN2. **Figure S15.** CDA for the region on chromosome 25 detected for the group of “meat-producing” goat breeds. (a): LONG and (b) SHORT: (b). Left: CDA plot. Right: Correlation value of the SNPs used for the analyses for CAN1 and CAN2. **Figure S16.** CDA for the region on chromosome 25 detected for the group of “milk-producing” goat breeds. (a): LONG and (b) SHORT: (b). Left: CDA plot. Right: Correlation value of the SNPs used for the analyses for CAN1 and CAN2. **Figure S17.** MDS plot of breeds considered for the panel of coat colors. Breed codes and subdivisions based on the coat color pattern are indicated in the right part of the plot. **Figure S18.** CDA for the region on chromosome 18 near the *MC1R* gene detected for the group of coat color breeds. (a): LONG and (b) SHORT: (b). Left: CDA plot. Right: Correlation value of the SNPs used for the analyses for CAN1 and CAN2. **Figure S19.** CDA for the region on chromosome 13 near the *ASIP* gene detected for the group of coat color breeds. (a): LONG and (b) SHORT: (b). Left: CDA plot. Right: Correlation value of the SNPs used for the analyses for CAN1 and CAN2. **Figure S20.** CDA for the region on chromosome 5 near the *ADAMTS20* gene detected for the group of coat color breeds. (a): LONG and (b) SHORT: (b). Left: CDA plot. Right: Correlation value of the SNPs used for the analyses for CAN1 and CAN2. **Figure S21.** MDS plot of the filtered dataset considering components 1 and 2. Animals are color-coded based on the Köppen classification of groups: Tropical (green), Dry (red), Temperate (orange), Continental (blue). **Figure S22.** *F*_ST_ plot of the comparison of the Dry group vs. the other groups. The threshold line in red represents the 0.995 of the percentile distribution (*F*_ST_ = 0.398). **Figure S23.** *F*_ST_ plot of the comparison of the Temperate group vs. the other groups. The threshold line in red represents the 0.995 of the percentile distribution (*F*_ST_ = 0.320). **Figure S24.** *F*_ST_ plot of the comparison of the Continental group vs. the other groups. The threshold line in red represents the 0.995 of the percentile distribution (*F*_ST_ = 0.507).

**Additional file 3: Table S1.** Environmental variables considered for the landscape genomic analysis. **Table S2.** FLK and hapFLK windows for the sub-continental groups after filtering steps. Overlapping or partially overlapping regions across the different geographical subdivisions are reported with the same letter as used for the population group (from ^a^ to ^m^). **Table S3.** Selective sweeps of early adaptation. The bold values indicate overlap or partial overlap between comparisons. **Table S4** Common regions between ROH and XP-EHH and/or *F*_ST_ analyses for the group of “fiber-producing” goat breeds and genes within these regions. **Table S5.** Common regions between ROH and XP-EHH and/or *F*_ST_ analyses for the group of “meat-producing” goat breeds and genes within these regions. **Table S6.** Common regions between ROH and XP-EHH and/or *F*_ST_ analyses for the group of “milk-producing” goat breeds and genes within these regions. **Table S7.** Top four regions detected for the coat color groups (Black, White and Red), indicated comparisons between the groups and genes located nearby these regions. **Table S8.** Results of the most significant associations involving the 57 filtered SNPs. For each SNP: SNP probe, genotype, genomic coordinate, associated environmental variable (“Env”) and scores and statistical test output values (“Gscore”, “WaldScore”, “Efron”, “AIC”, “Beta_0” and “Beta_1”) are reported. **Table S9.** List of genes located nearby (± 100 kb) the 57 filtered SNPs obtained by the landscape genomics analysis. **Table S10.** GO term biological processes. **Table S11.** MAF (minor allele frequency) and major allele of the 13 SNPs shared by Dry and Temperate/Continental groups. **Table S12.** Samβada significant results for the SNPs of the *F*_ST_ analyses. For each SNP probe: chromosome and position are reported, as well as Environment, G score, Wald Score, AIC, Abs_Beta1 and the Köppen group in which the SNP was detected by the *F*_ST_ analyses. **Table S13.** List of genes located nearby (± 100 kb) of the 65 SNPs selected with the *F*_ST_ approach and confirmed by Samβada. The * symbol indicates the SNPs with the highest value (> 0.999) also in landscape genomic analysis

**Additional file 4.** Graphical representation of ROH and iHS results at the breed level of the signals FLK and hapFLK in the sub continental groups. Left panel: FLK (points) and hapFLK (line) signatures, with as header the chromosomal region investigated. Middle panels: ROH signatures. Right panels: iHS signatures [see Additional file 2]

**Additional file 5.** Genotype distribution of the SNPs detected in the landscape genomics analysis based on the GPS coordinates. Each page represent a SNP, with its coordinates reported on the upper right part of each sheet, with environmental variable associated and statistics (G score, Beta 1 and AIC). The three possible genotypes are labelled with three different colors: *AA* = red; *AB* = green; *BB* = blue
