# Supplementary material for: Signatures of selection and environmental adaptation across the goat genome post-domestication
Source: Genet Sel Evol. 2018 Nov 19;50:57. doi: 10.1186/s12711-018-0421-y (PMC6240954; doi:10.1186/s12711-018-0421-y)
Supplement: Supplementary file 1 — Additional file 1.Treemix plots. Each page shows the output of a continental/sub-continental group [file 12711_2018_421_MOESM1_ESM.pdf]

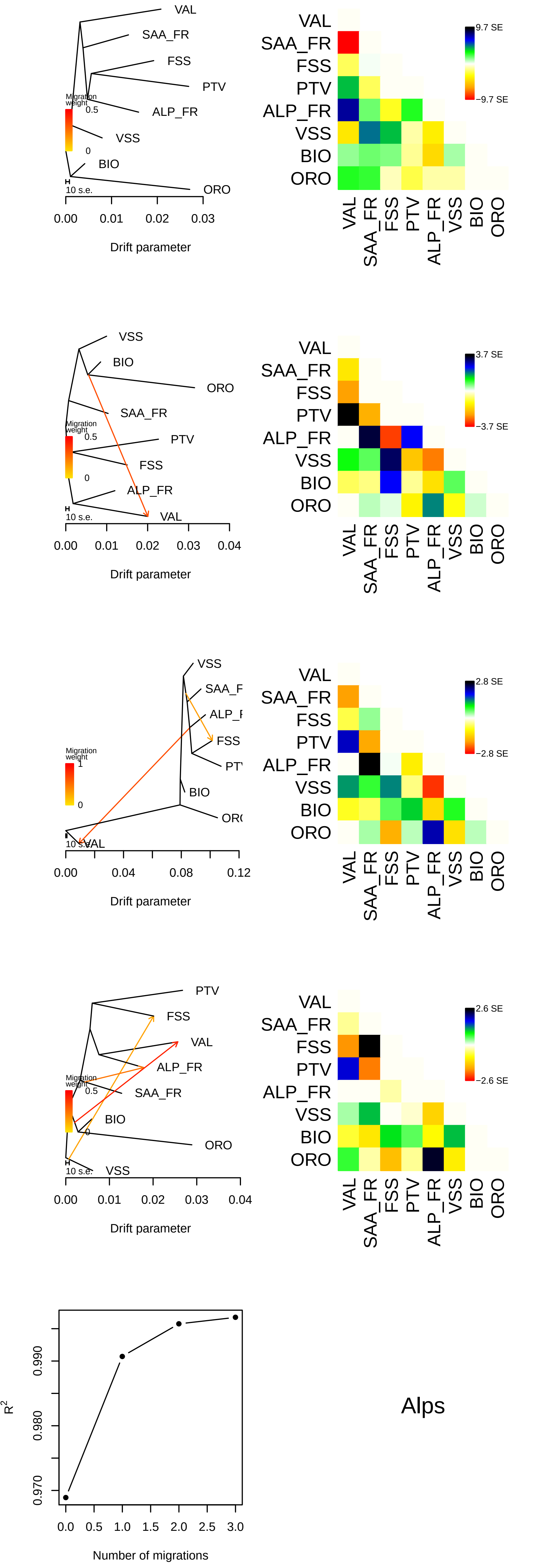
VAL
SAA_FR
FSS
PTV
ALP_FR
VSS
BIO
ORO
Migration weight
0.5
0
10 s.e.
0.00 0.01 0.02 0.03
Drift parameter
9.7 SE
−9.7 SE
VSS
BIO
ORO
SAA_FR
PTV
FSS
ALP_FR
VAL
0.00 0.01 0.02 0.03 0.04
Drift parameter
3.7 SE
−3.7 SE
VSS
SAA_F
ALP_F
FSS
PT
BIO
OR
VAL
Migration weight
1
0
0.00 0.04 0.08 0.12
Drift parameter
2.8 SE
−2.8 SE
PTV
FSS
VAL
ALP_FR
SAA_FR
BIO
ORO
VSS
0.00 0.01 0.02 0.03 0.04
Drift parameter
2.6 SE
−2.6 SE
R^2
0.970
0.980
0.990
0.0 0.5 1.0 1.5 2.0 2.5 3.0
Number of migrations
Alps

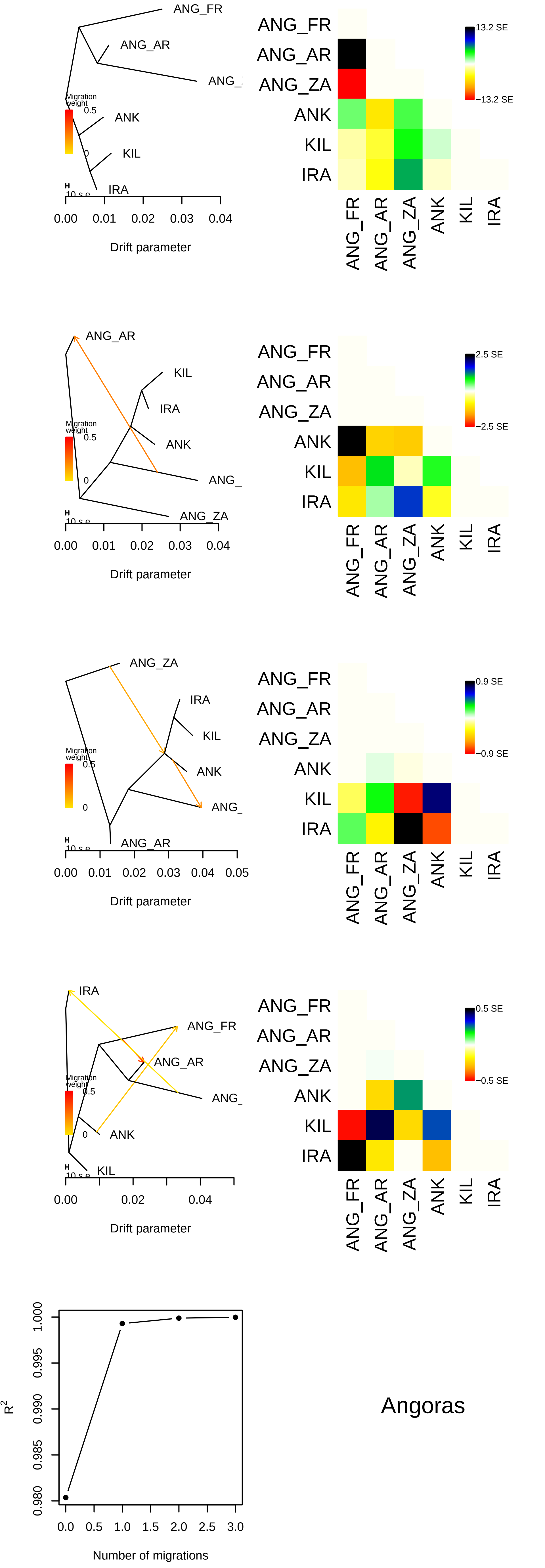
ANG_FR
ANG_AR
ANG_ZA
ANK
KIL
IRA
Migration weight
0.5
0
10 s.e.
0.00
0.01
0.02
0.03
0.04
0.05
Drift parameter
13.2 SE
−13.2 SE
2.5 SE
−2.5 SE
0.9 SE
−0.9 SE
0.5 SE
−0.5 SE
R^2
0.980
0.985
0.990
0.995
1.000
0.0
0.5
1.0
1.5
2.0
2.5
3.0
Number of migrations
Angoras

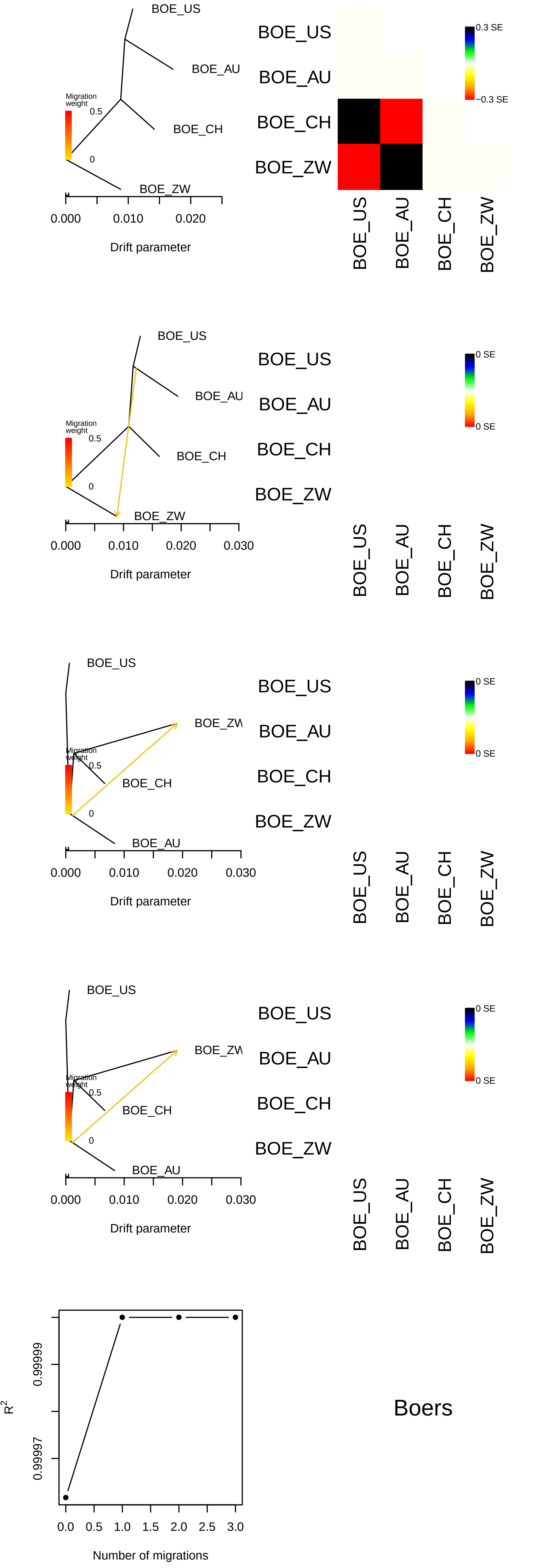

BOE_US
BOE_AU
BOE_CH
BOE_ZW
Migration weight
0.5
0
0.000
0.010
0.020
0.030
Drift parameter
0.3 SE
−0.3 SE
0 SE
R^2
0.99999
0.99997
0.0
0.5
1.0
1.5
2.0
2.5
3.0
Number of migrations
Boers

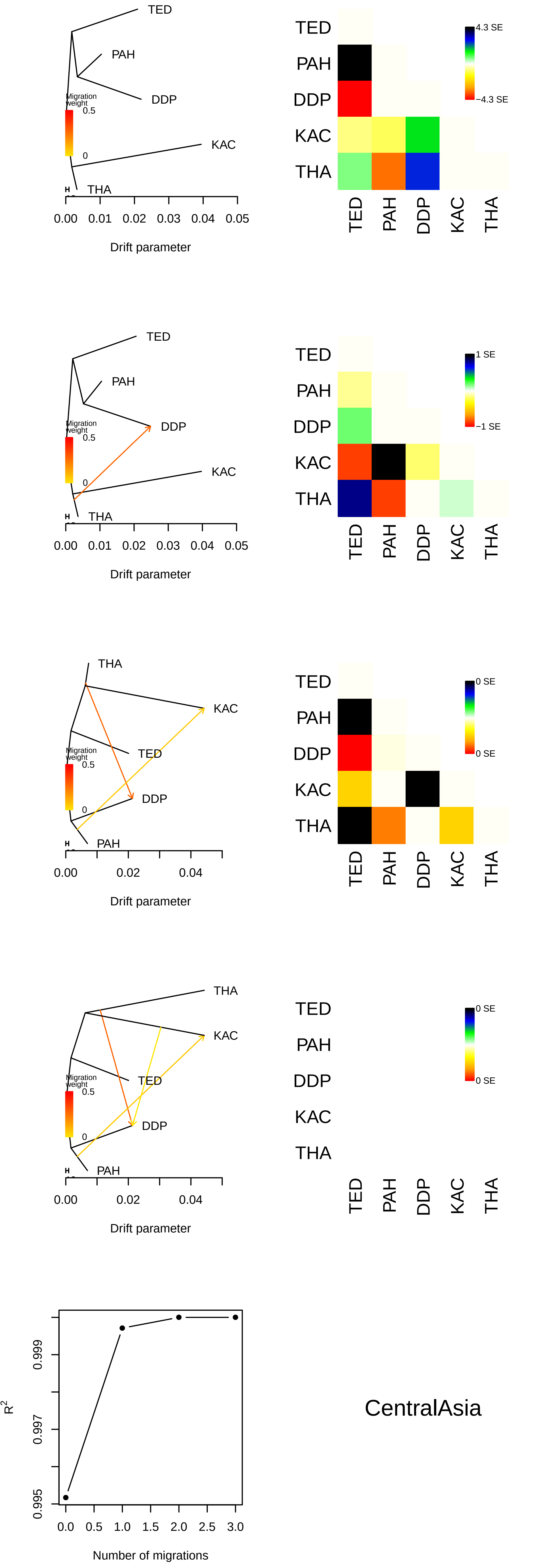
TED
PAH
DDP
KAC
THA
Migration weight
0.5
0
0.00 0.01 0.02 0.03 0.04 0.05
Drift parameter
4.3 SE
−4.3 SE
1 SE
−1 SE
0 SE
0.00 0.02 0.04
0.995 0.997 0.999
R^2
0.0 0.5 1.0 1.5 2.0 2.5 3.0
Number of migrations
CentralAsia

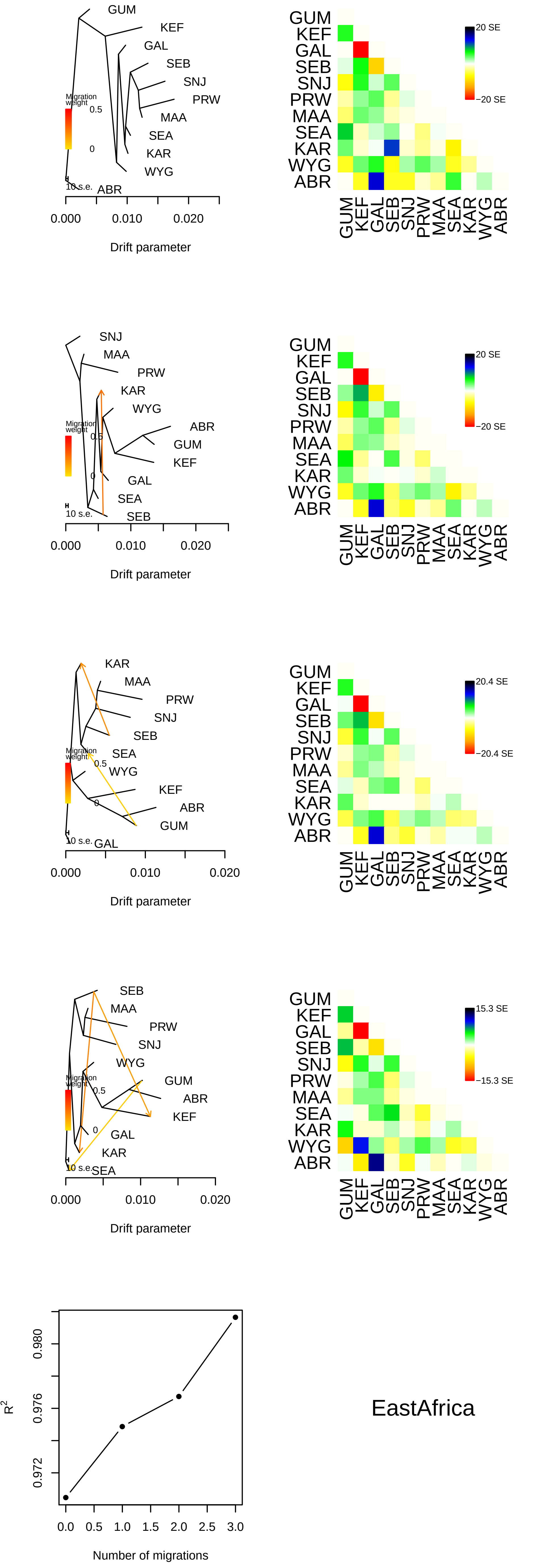

Migration weight
10 s.e.
Drift parameter
0.000
0.010
0.020
GUM
KEF
GAL
SEB
SNJ
PRW
MAA
SEA
KAR
WYG
ABR
20 SE
−20 SE
20.4 SE
−20.4 SE
15.3 SE
−15.3 SE
R^2
Number of migrations
EastAfrica

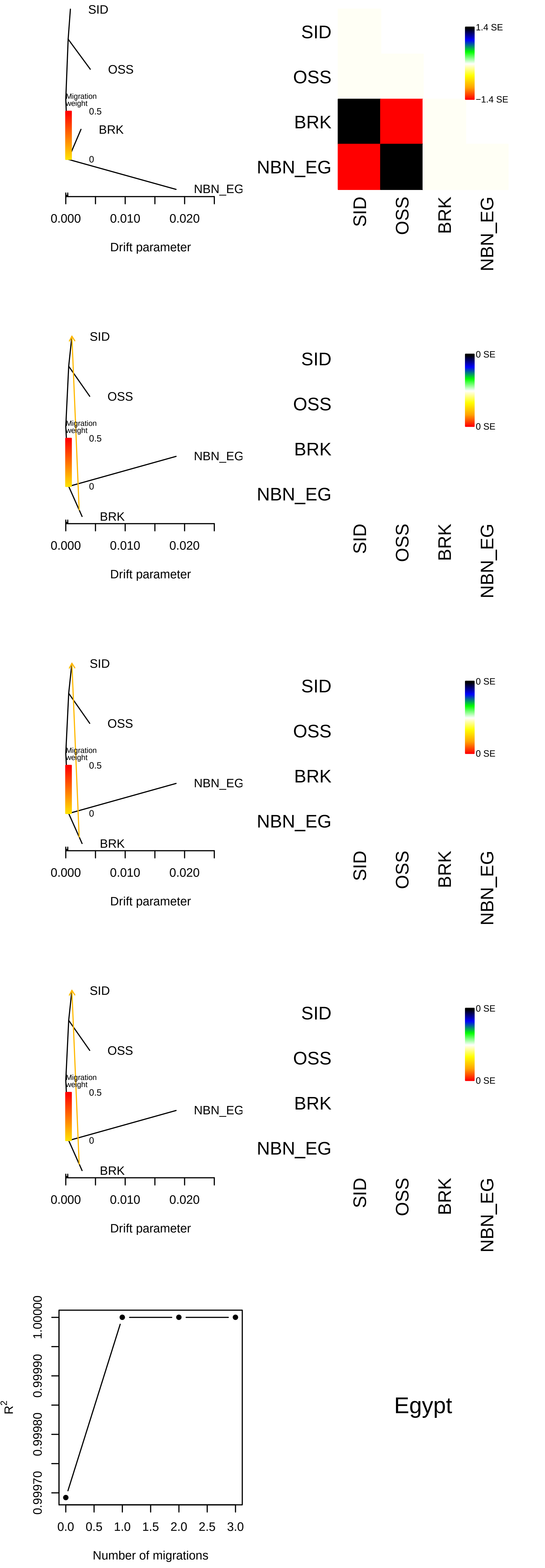
SID
OSS
BRK
NBN_EG
Migration weight
0.5
0
0.000
0.010
0.020
Drift parameter
1.4 SE
−1.4 SE
0 SE
0 SE
R^2
0.99970
0.99980
0.99990
1.00000
0.0
0.5
1.0
1.5
2.0
2.5
3.0
Number of migrations
Egypt

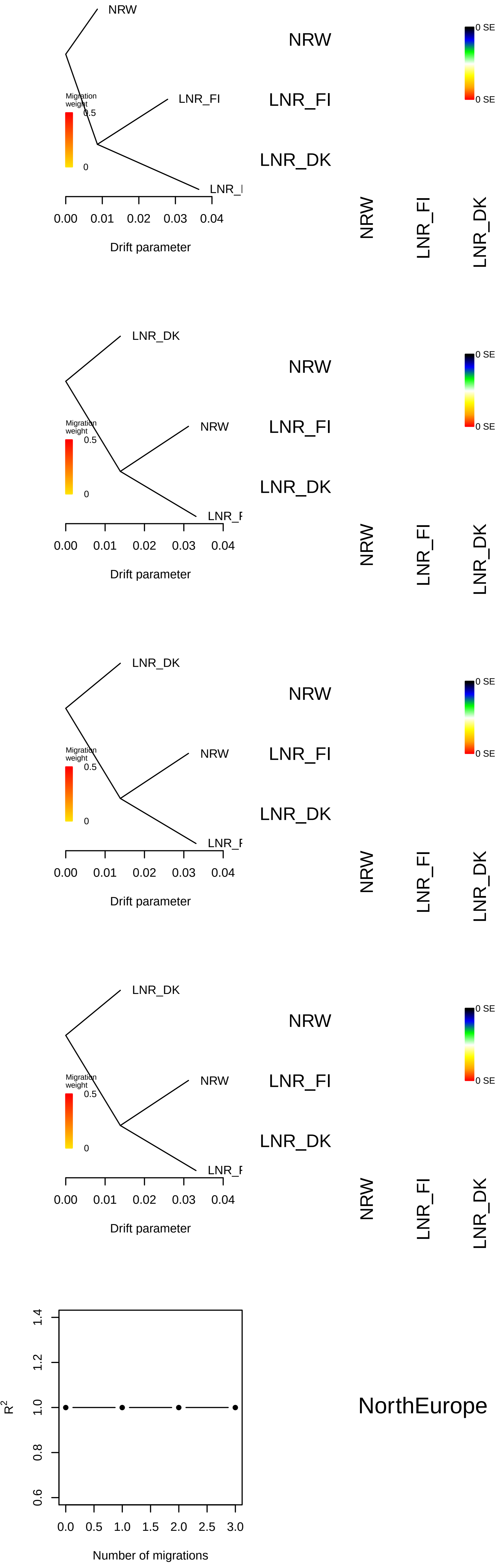
NRW
LNR_FI
LNR_DK
Migration weight
0.5
0
Drift parameter
0.00 0.01 0.02 0.03 0.04
0 SE
Number of migrations
R^2
NorthEurope

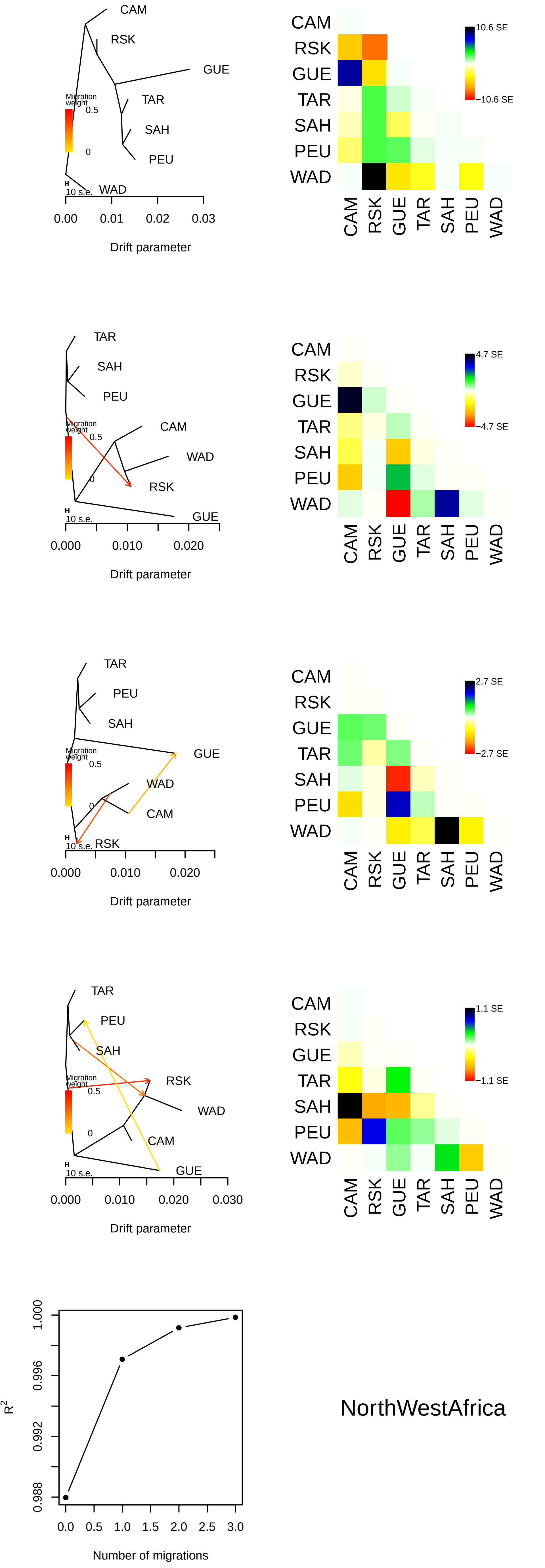
CAM
RSK
GUE
TAR
SAH
PEU
WAD
Migration weight
0.5
0
10 s.e.
Drift parameter
10.6 SE
−10.6 SE
4.7 SE
−4.7 SE
2.7 SE
−2.7 SE
1.1 SE
−1.1 SE
0.00 0.01 0.02 0.03
0.000 0.010 0.020
0.000 0.010 0.020 0.030
R^2
0.988 0.992 0.996 1.000
0.0 0.5 1.0 1.5 2.0 2.5 3.0
Number of migrations
NorthWestAfrica

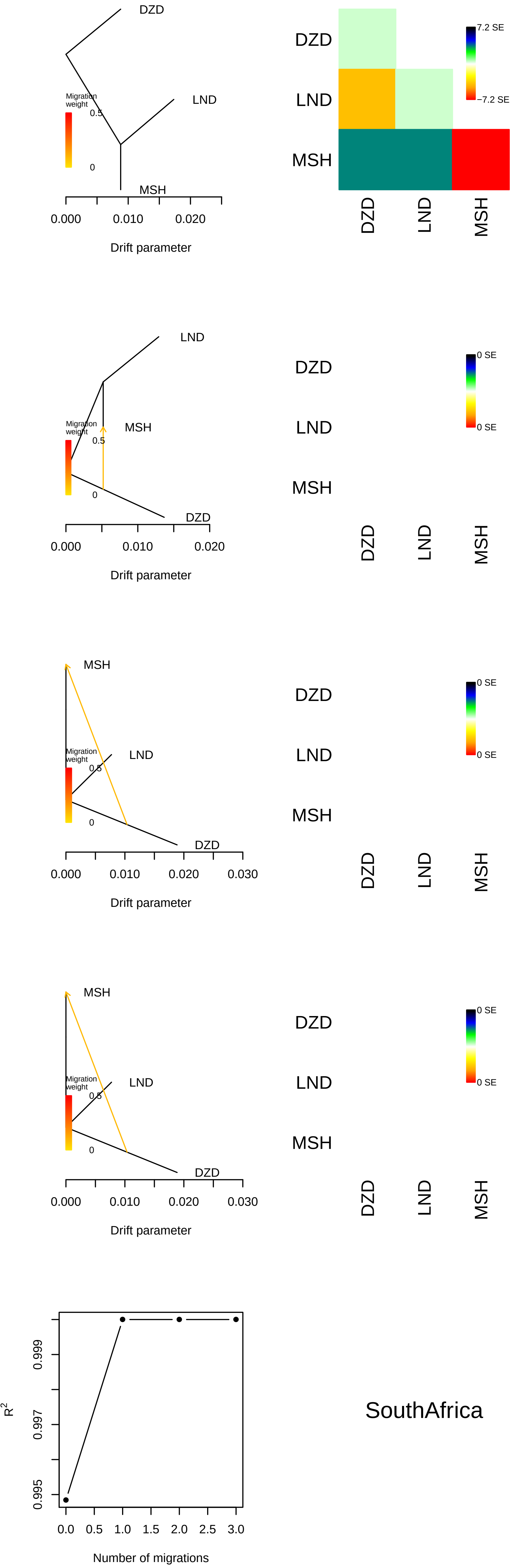
DZD
LND
MSH
Migration weight
0.5
0
0.000
0.010
0.020
Drift parameter
7.2 SE
−7.2 SE
DZD
LND
MSH
LND
MSH
DZD
Migration weight
0.5
0
0.000
0.010
0.020
Drift parameter
0 SE
0 SE
DZD
LND
MSH
MSH
LND
DZD
Migration weight
0.5
0
0.000
0.010
0.020
0.030
Drift parameter
0 SE
0 SE
DZD
LND
MSH
MSH
LND
DZD
Migration weight
0.5
0
0.000
0.010
0.020
0.030
Drift parameter
0 SE
0 SE
DZD
LND
MSH
R2
0.995
0.997
0.999
0.0
0.5
1.0
1.5
2.0
2.5
3.0
Number of migrations
SouthAfrica

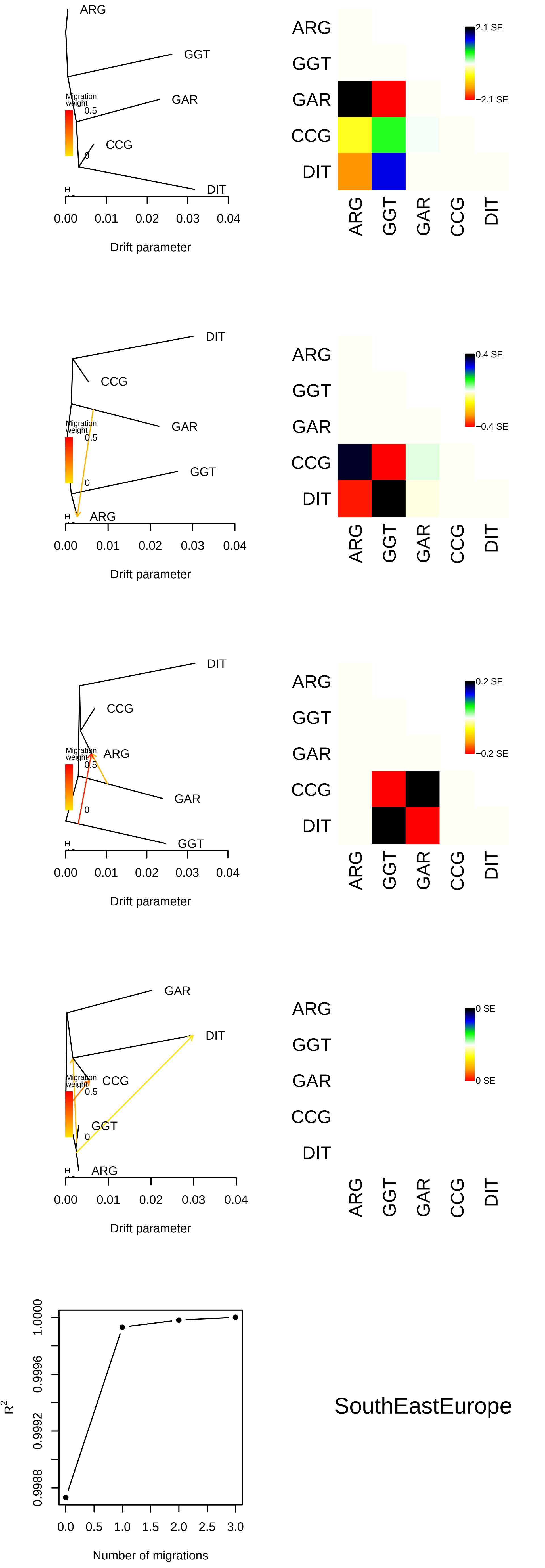

ARG
GGT
GAR
CCG
DIT
Migration weight
0.5
0
0.00 0.01 0.02 0.03 0.04
Drift parameter
2.1 SE
−2.1 SE
0.4 SE
−0.4 SE
0.2 SE
−0.2 SE
0 SE
0 SE
R^2
1.0000
0.9996
0.9992
0.9988
0.0 0.5 1.0 1.5 2.0 2.5 3.0
Number of migrations
SouthEastEurope

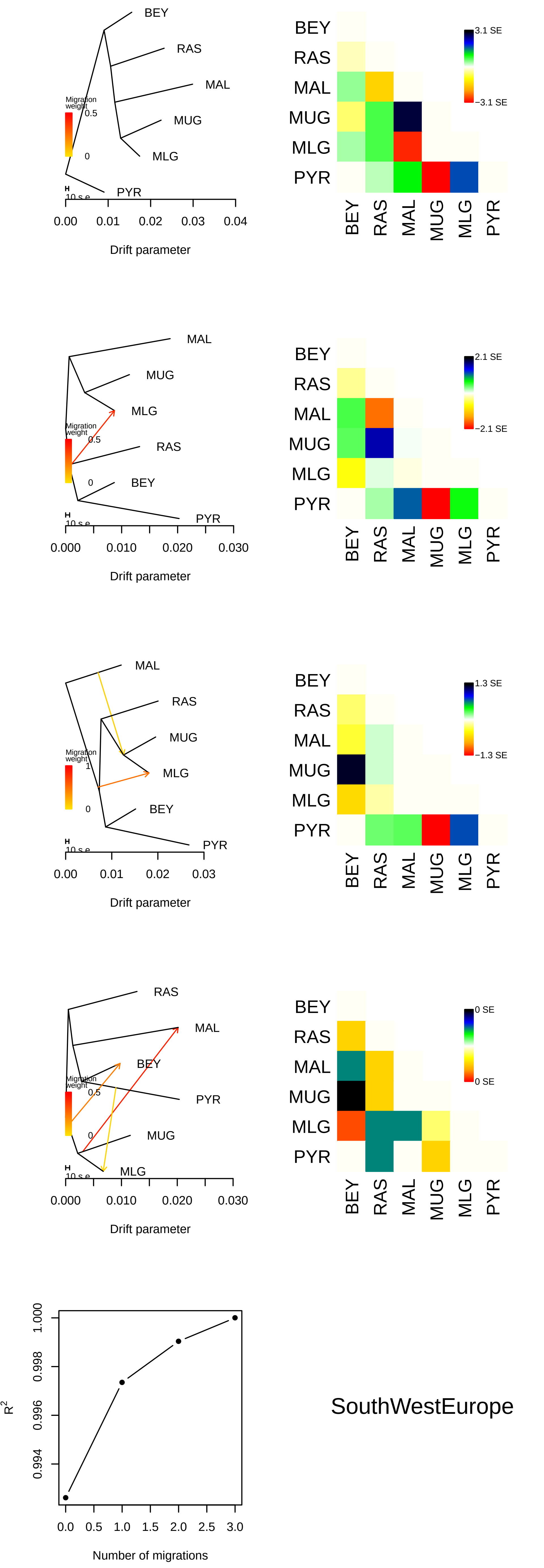
BEY
RAS
MAL
MUG
MLG
PYR
Migration weight
Drift parameter
3.1 SE
−3.1 SE
2.1 SE
−2.1 SE
1.3 SE
−1.3 SE
0 SE
10 s.e.
Number of migrations
R^2
SouthWestEurope
