# Supplementary material for: Signatures of selection and environmental adaptation across the goat genome post-domestication
Source: Genet Sel Evol. 2018 Nov 19;50:57. doi: 10.1186/s12711-018-0421-y (PMC6240954; doi:10.1186/s12711-018-0421-y)

**Additional File 2 for:**

**Signatures of selection and environmental adaptation across the goat genome post domestication.**

Francesca Bertolini, Bertrand Servin, Andrea Talenti, Estelle Rochat, Eui Soo Kim, Claire Oget, Isabelle Palhière, Alessandra Crisà, Gennaro Catillo, Roberto Steri, Marcel Amills, Licia Colli, Gabriele Marras, Marco Milanesi, Ezequiel Nicolazzi, Benjamin D Rosen, Curtis P Van Tassell, Bernt Guldbrandtsen, Tad S Sonstegard, Gwenola Tosser-Klopp, Alessandra Stella, Max F Rothschild, Stéphane Joost, Paola Crepaldi and the ADAPTmap consortium

**Figure S1**

Title: MDS plot of the breeds, grouped by production purpose: milk, meat and fiber groups

Description: group colors: milk = green, meat = red, fiber = blue; MDS and box plots of the first two components pre-filtering (upper) and MDS plots after filtering.


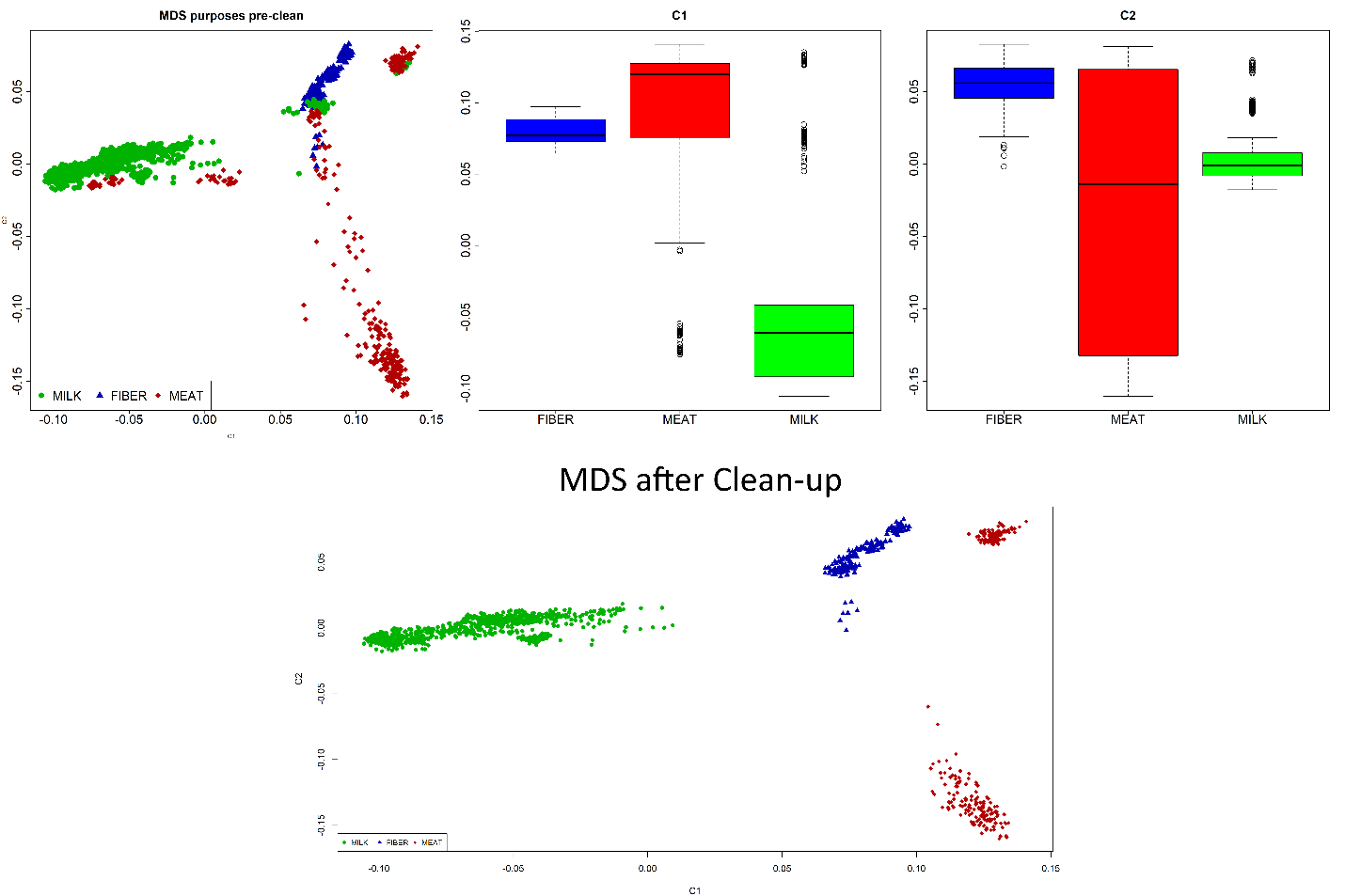


**Figure S2**

Title: Manhattan plot of the FLK results for the sub-continental group after filtering steps.

Description: Sub-geographical group names are reported on top of each plot. Chromosomes are alternates red and black.


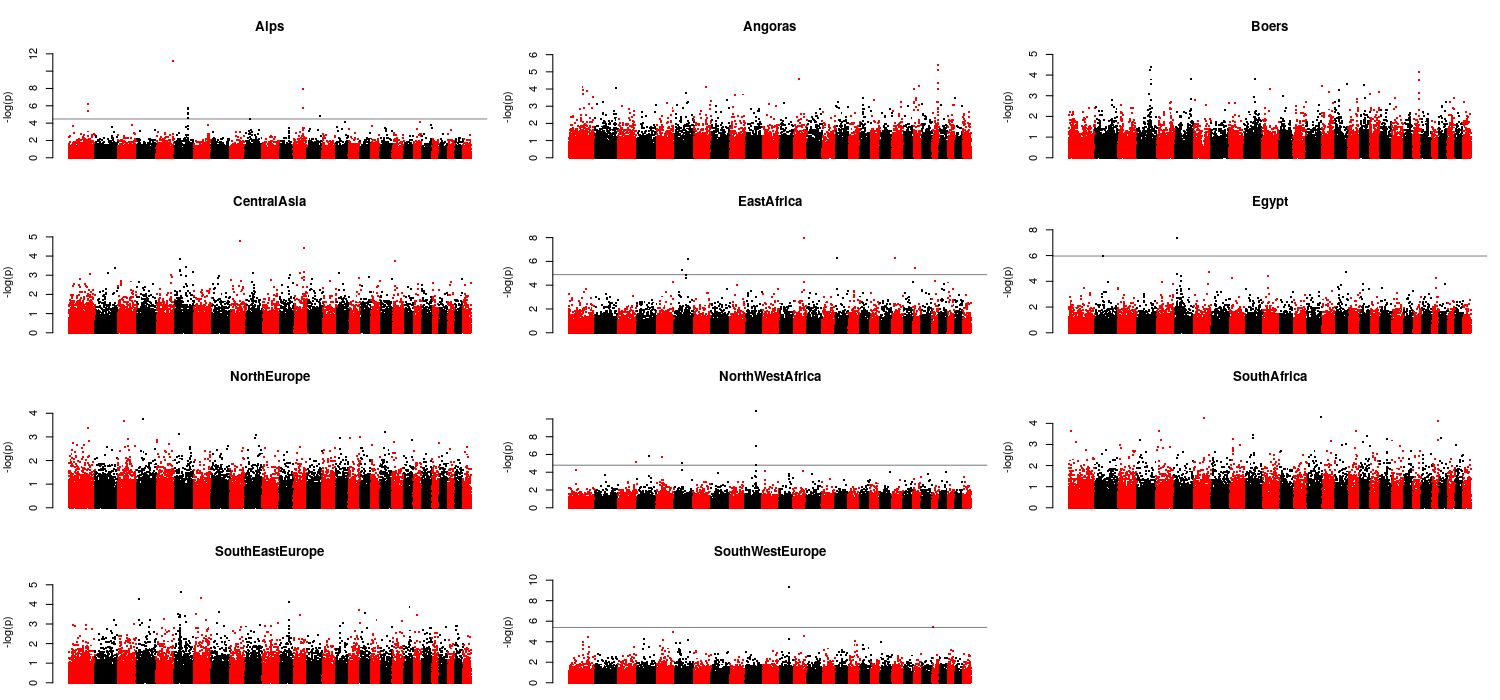


Figure S3

Title: Manhattan plot of the hapFLK results for the sub-continental group after filtering steps

Description: Sub-geographical group names are reported on top of each plot. Chromosomes are alternates red and black.


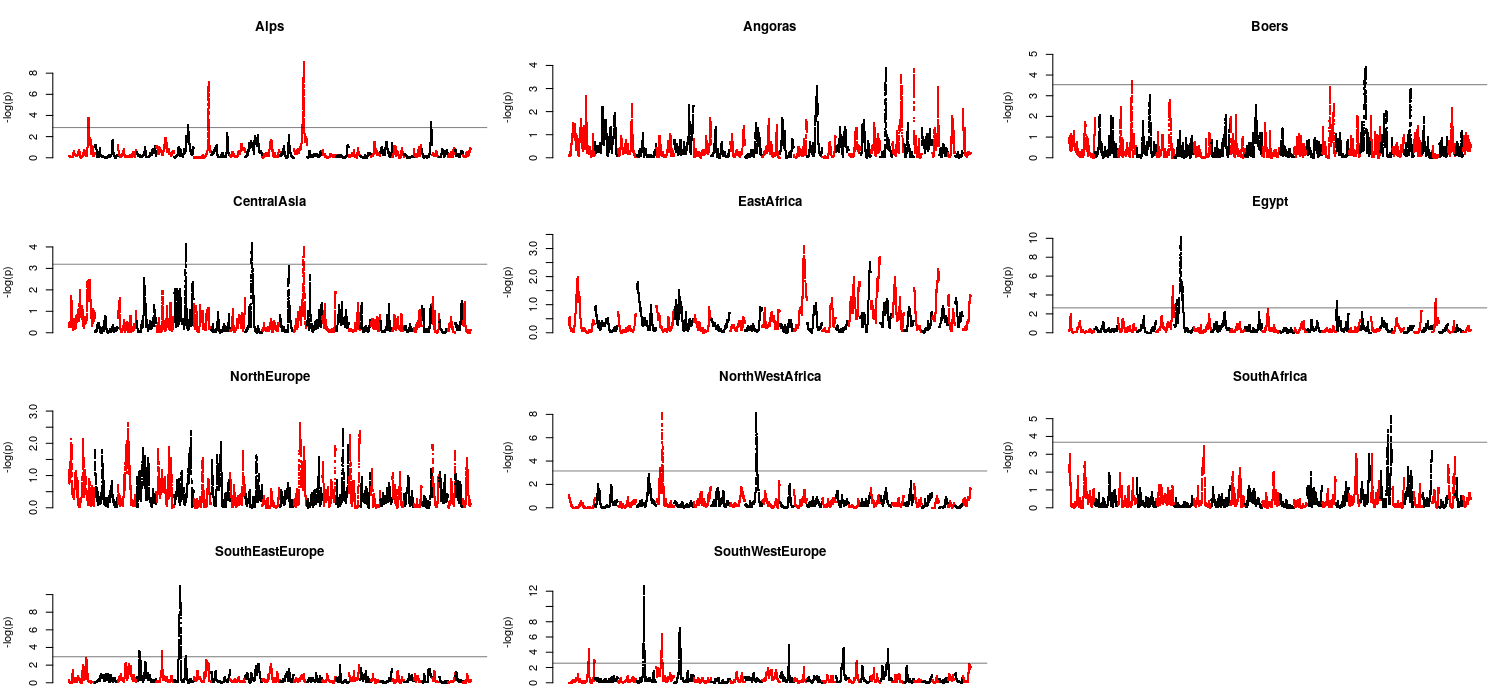


Figure S4

Title: Genomic distribution of FLK and hapFLK signals across population groups

Description: For each chromosome and each sub-continental group, the chromosomal position detected with at least one of the two approaches are detected.


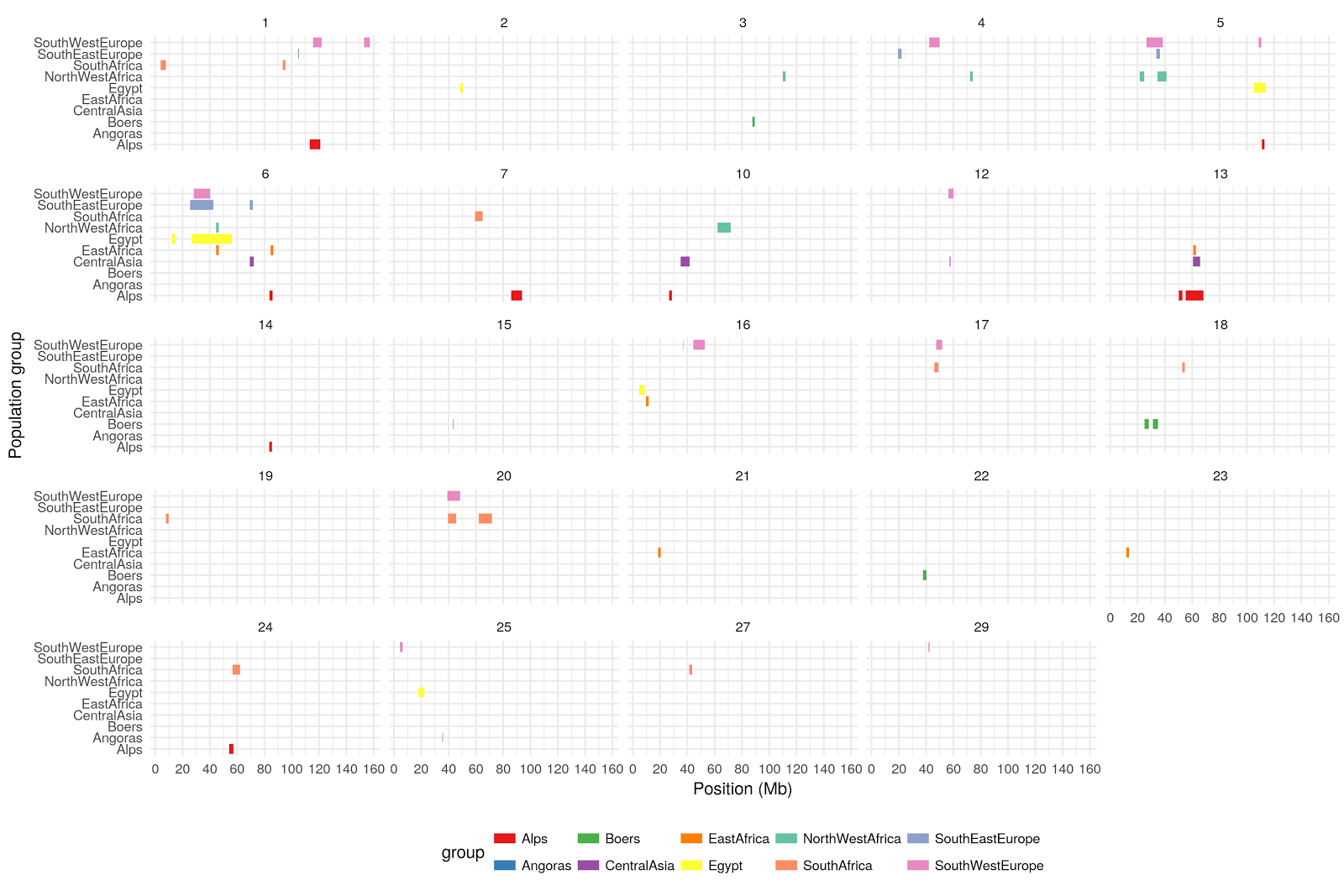


Figure S5

Title: Signatures of selection on chromosome 5 for the North western Africa, South eastern Africa and South western Africa groups.

Description: North western Africa (NWA): red; South eastern Europe (SEE): green; South western Europe (SWE): blue. The table (bottom-right) reported the genes within the region in which a signature was detected.


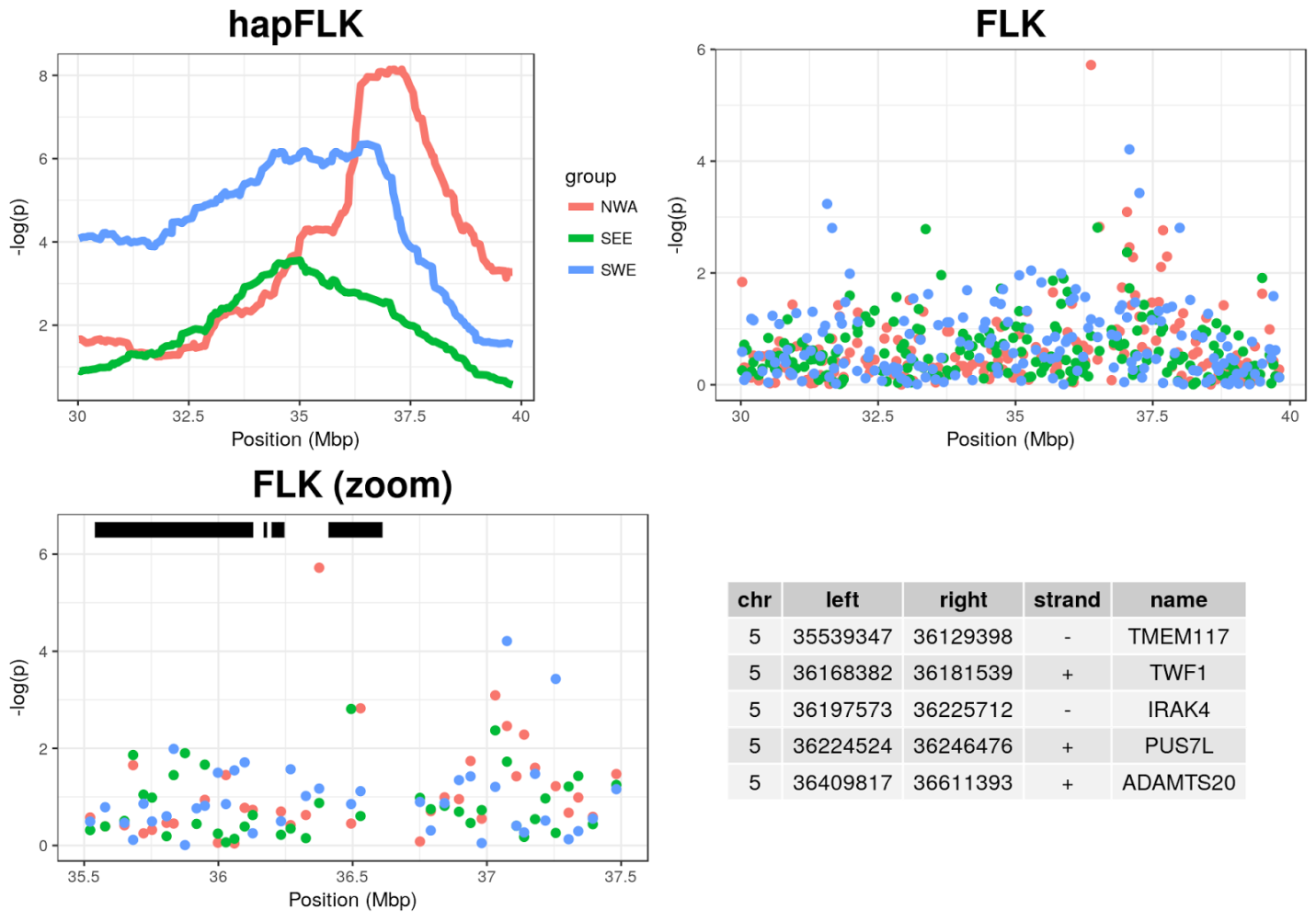


Figure S6

Title: Signatures of selection on chromosome 6 for the Central Asia, East Africa and South east Europe groups.

Description: Central Asia (CA): red; East Africa (EA): green; South east Europe (SEE): blue. The table (upper-right) reported the genes within the region in which a signature was detected.


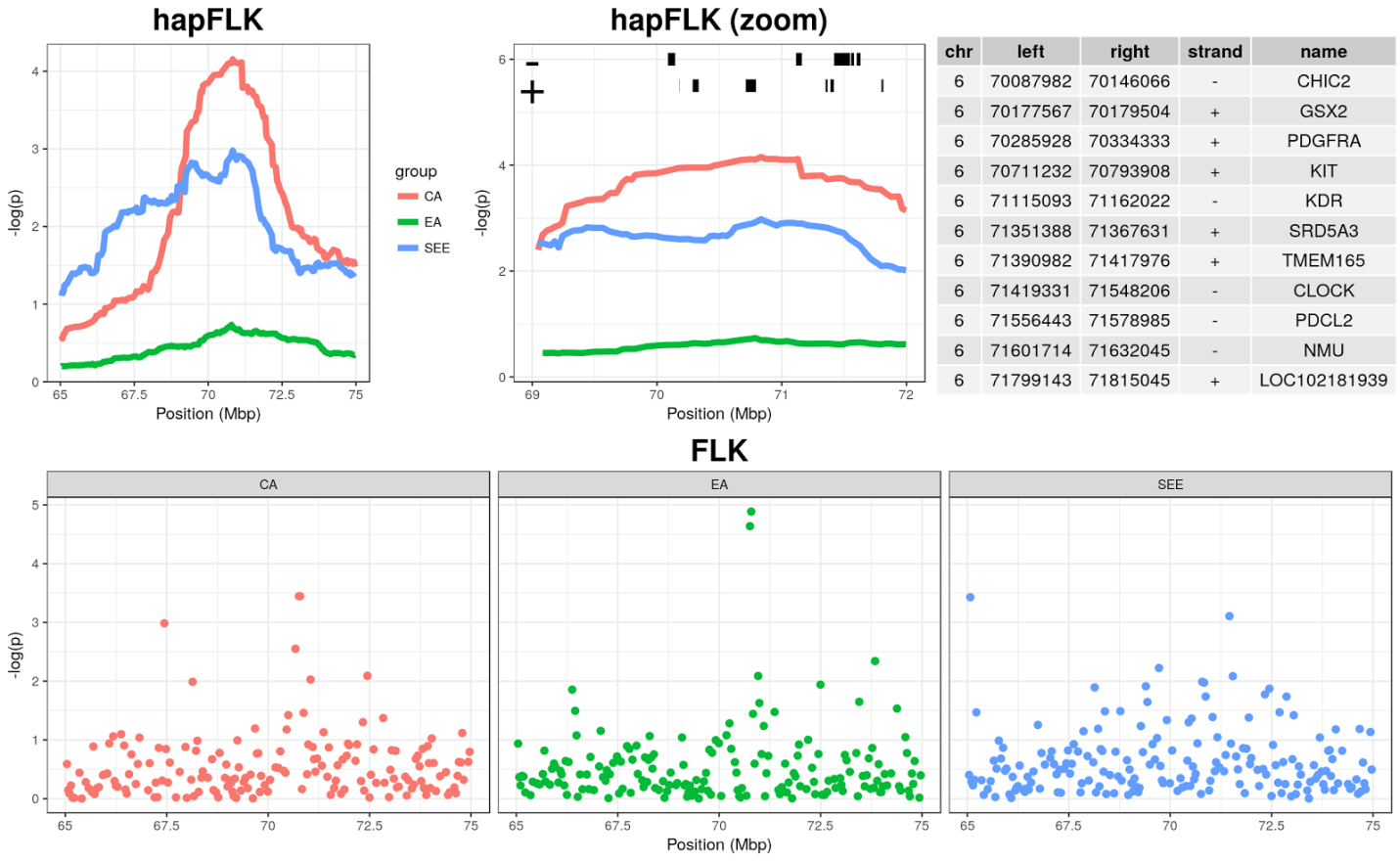


Figure S7

Title: Signatures of selection on chromosome 13 for the Alpines and Central Asia groups.

Description: Alpines (Alps): red; Central Asia (CA): blue. The table (right) reported the genes within the region in which a signature was detected.


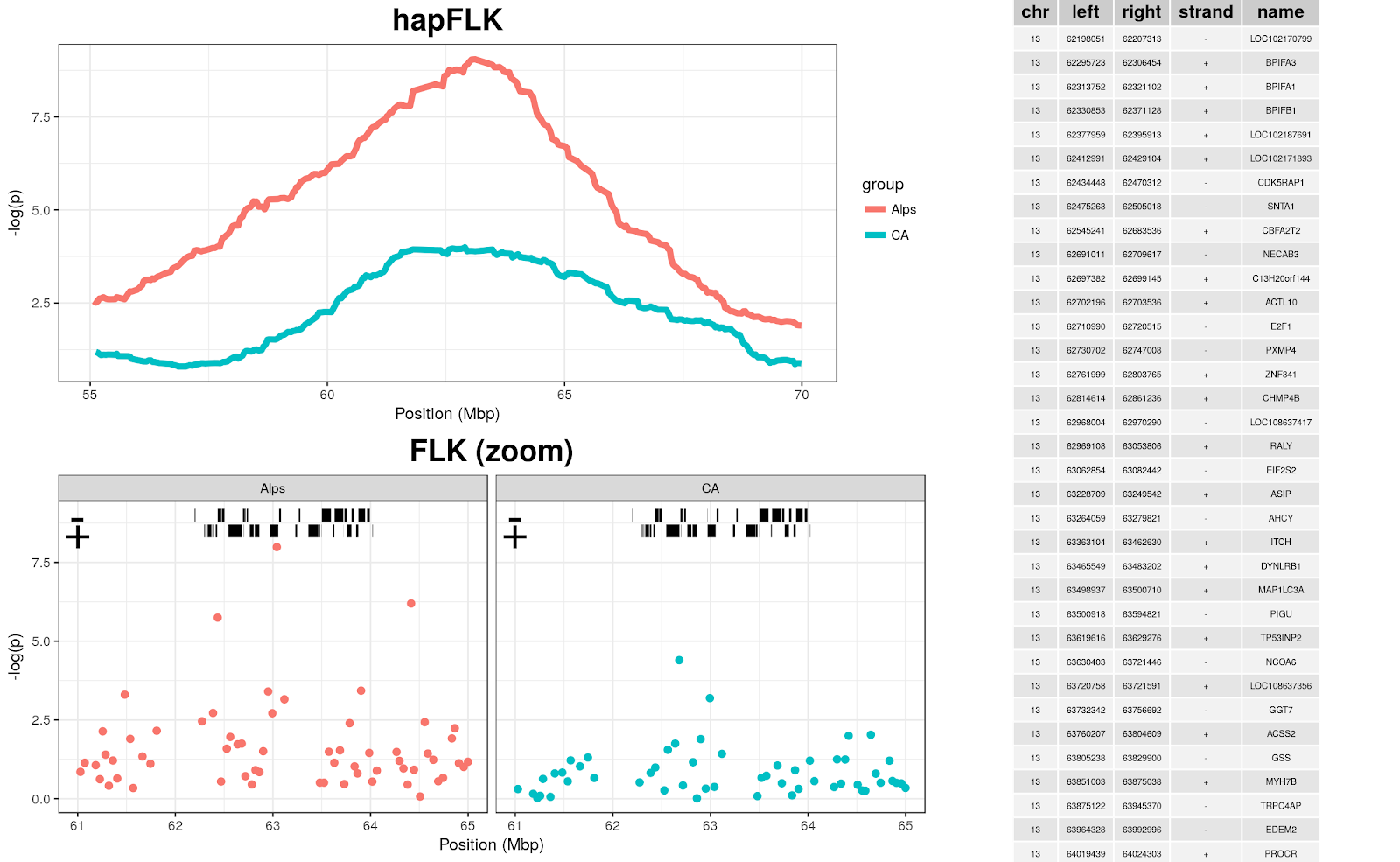


Figure S8

Title: Signatures of selection on chromosome 1 for the Alpines and South western Europe

Description: Alpines (Alps); red; South western Europe (SWE): blue. The table (bottom-right) reported the genes within the region in which a signature was detected.


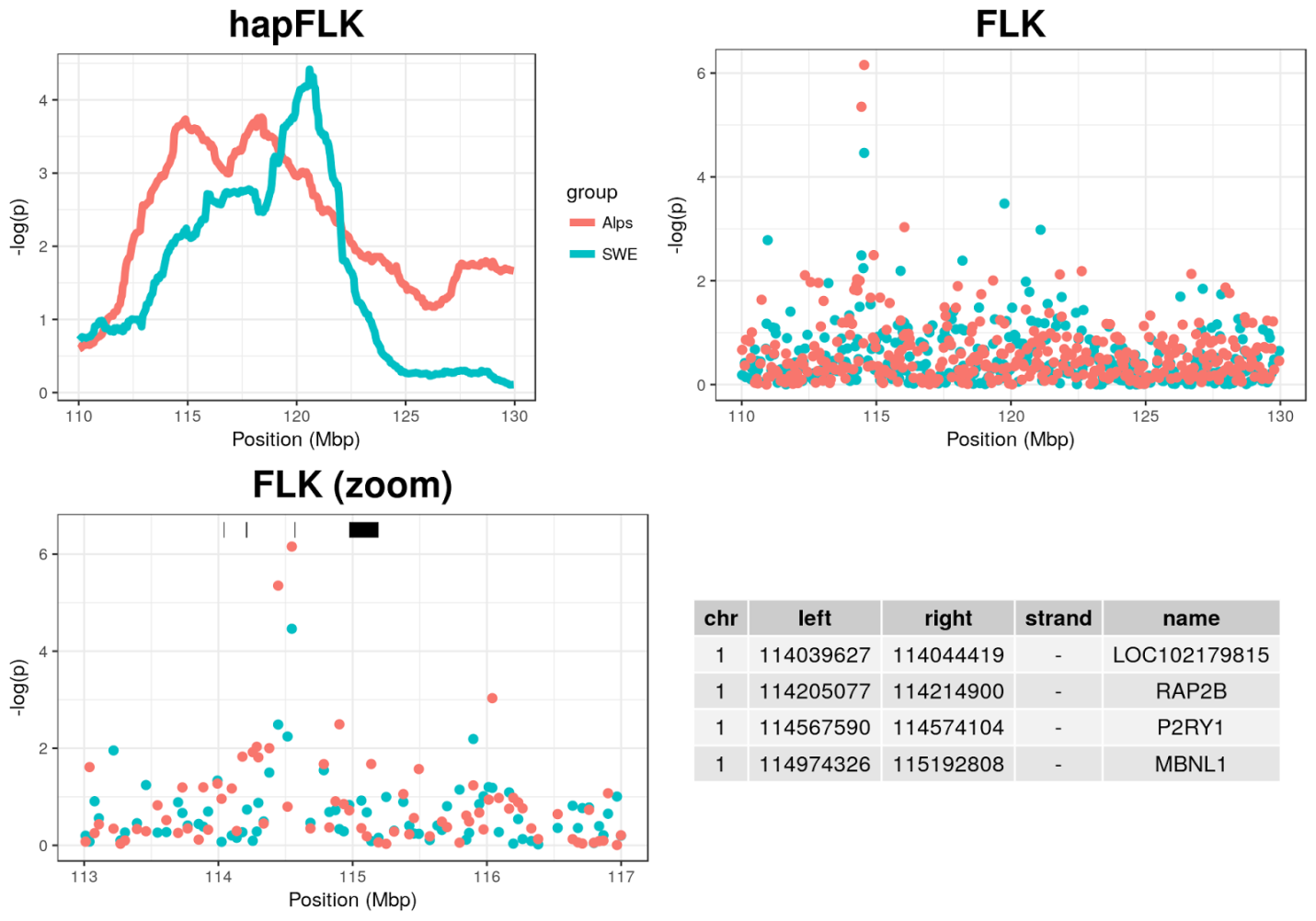


Figure S9

Title: FLK signals on CHI6 around the casein gene cluster. The cluster of unannotated genes between *YTHDC1* and *SULT1B1* consists of genes coding for glucuronosyltransferase enzymes.

Description: Alps; red; East Arica (EA): blue. The table (right) reported the genes within the casein cluster region.


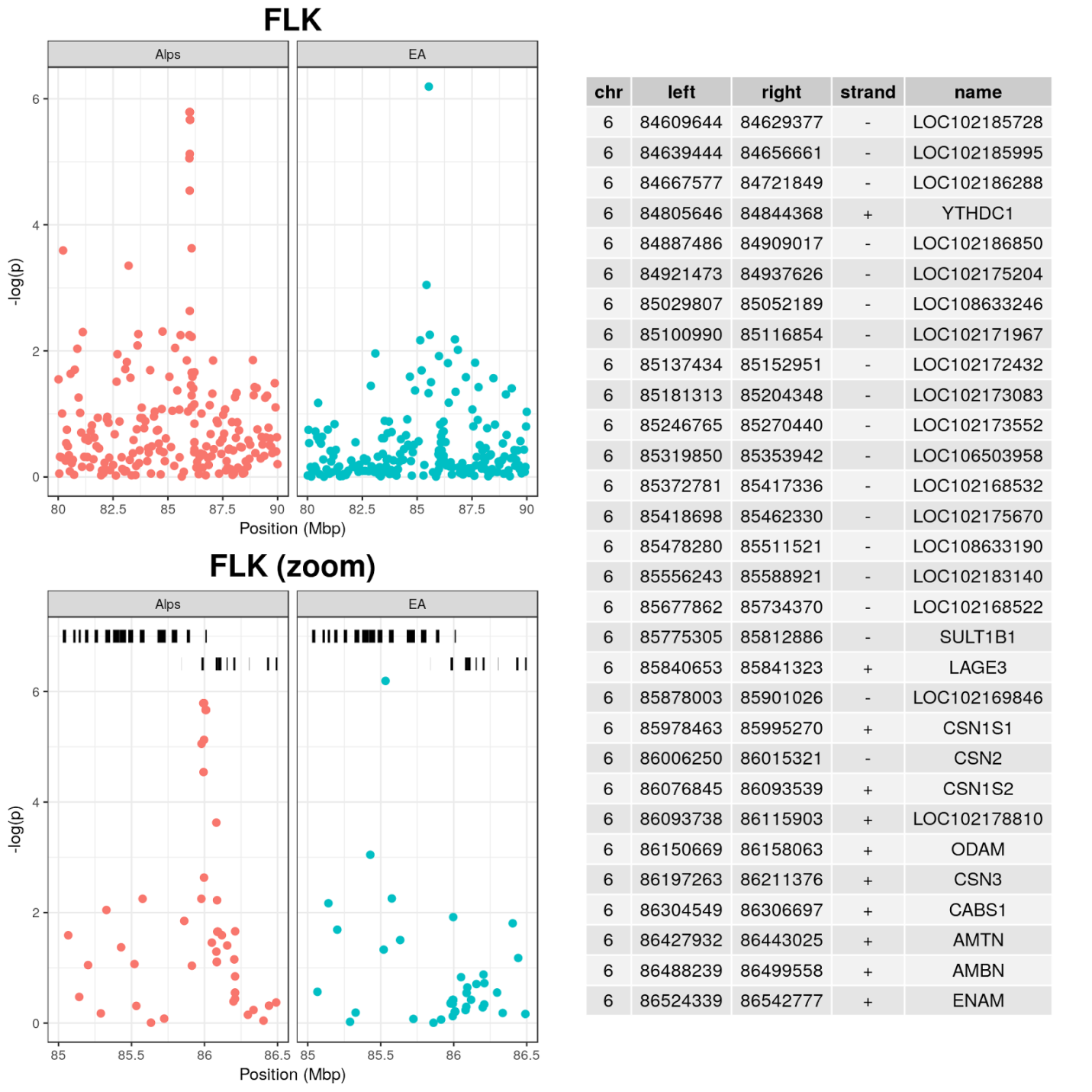


Figure S9. Selection signature, on CHI12 in the CA and SWE groups.


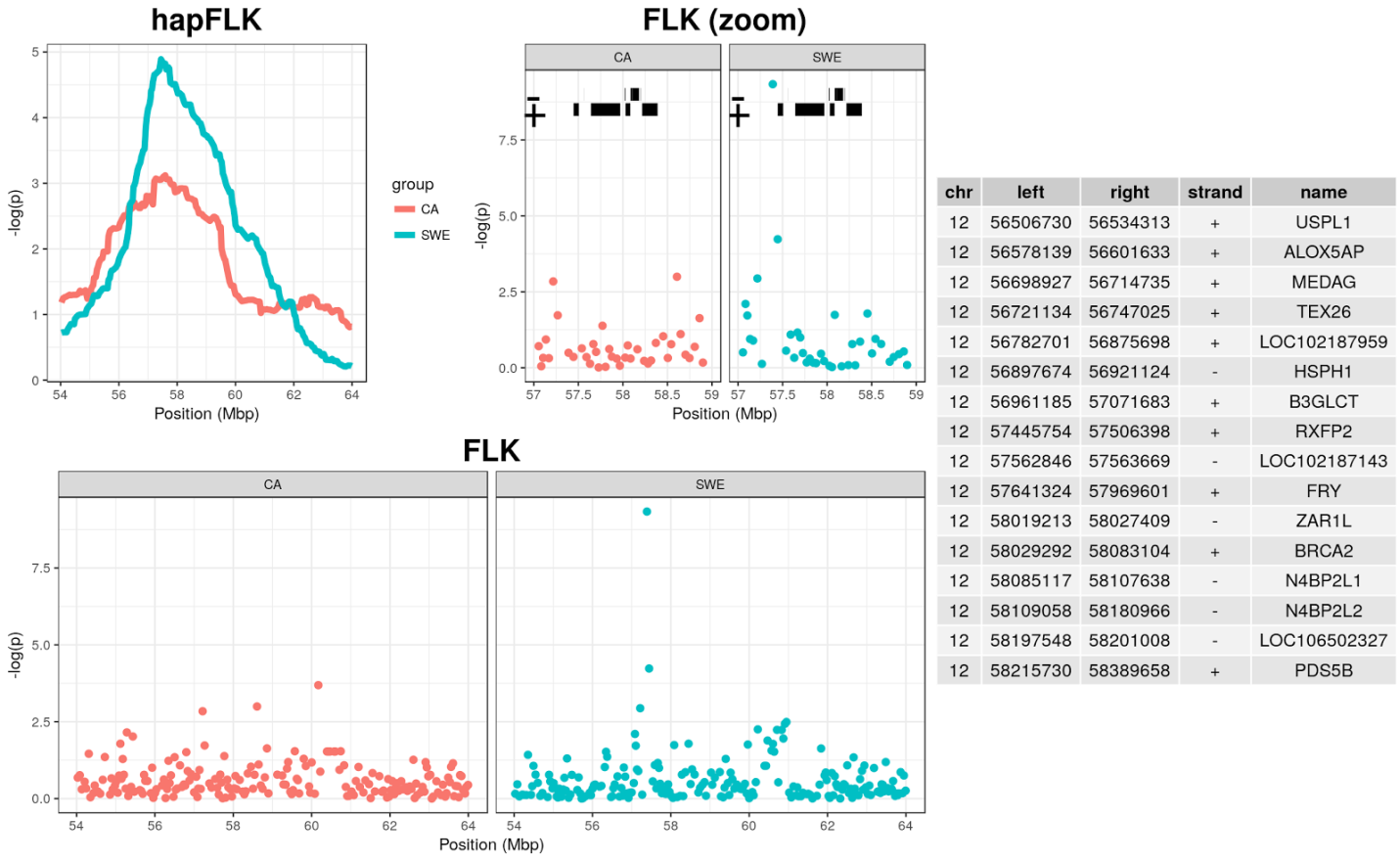


Figure S10

Title: Signatures of selection on chromosome 6 for the Egypt, South eastern Europe and South western Europe groups.

Description: Egypt (Egypt): red; South eastern Europe (SEE): green; South western Europe (SWE): blue. The table (bottom-right) reported the genes within the region in which a signature was detected.


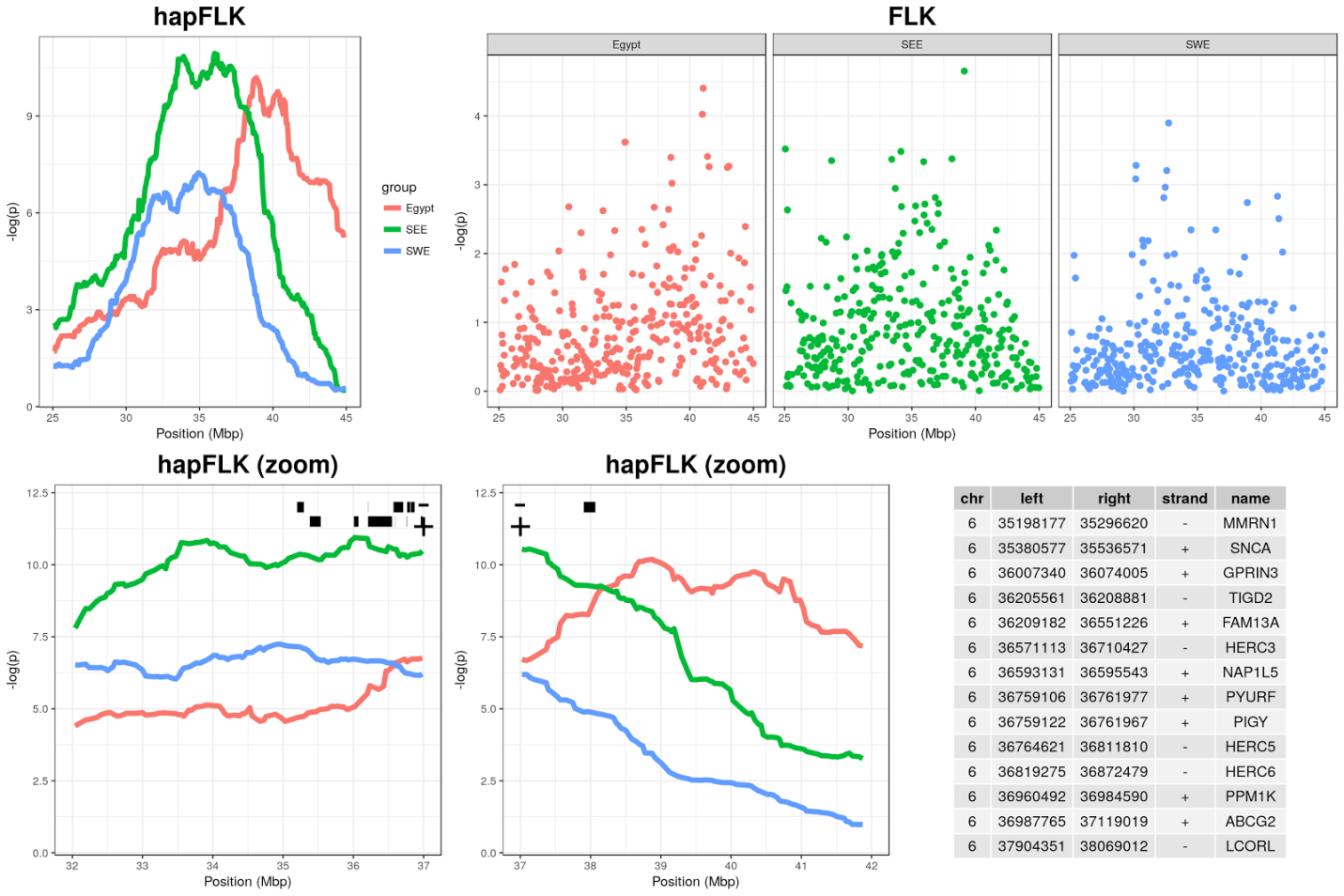


Figure S11

Title: Signatures of selection on chromosome 12 for the Central Asia and South western Europe


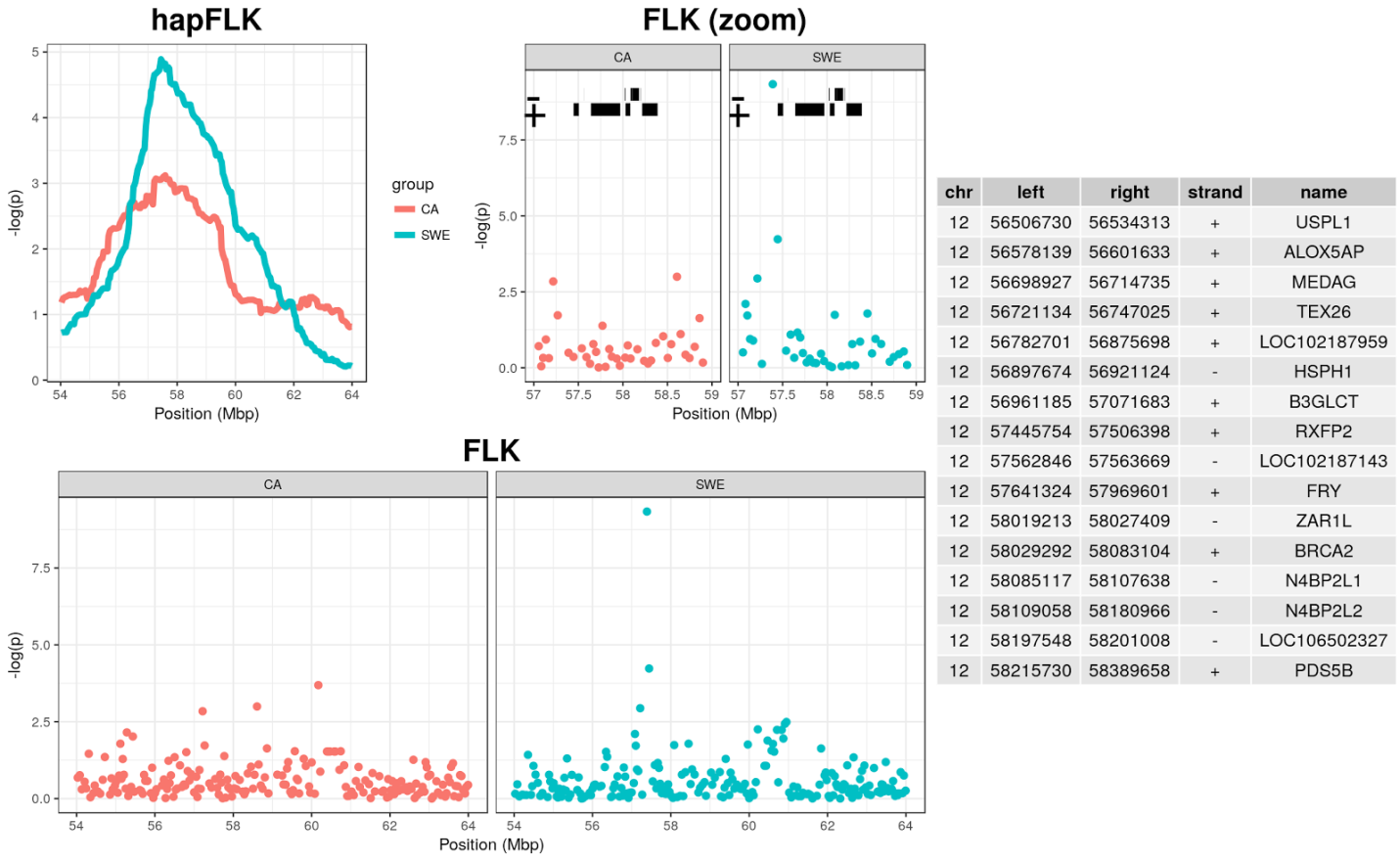
Description: Central Asia (CA); red; South western Europe (SWE): blue. The table (right) reported the genes within the region in which a signature was detected.

Figure S12

Title: ROH, *F*_ST_ and XP-EHH results for the group of “meat-producing” goat breeds

Description: Analysis types showed with different plot colors, within the most external squared-based circle, where each color represent a chromosome (chromosome number outside the squares): green (external) = ROH; blue (middle) = Fst; violet (internal): XP-EHH. For the three analyses, the regions above the threshold are marked in red.


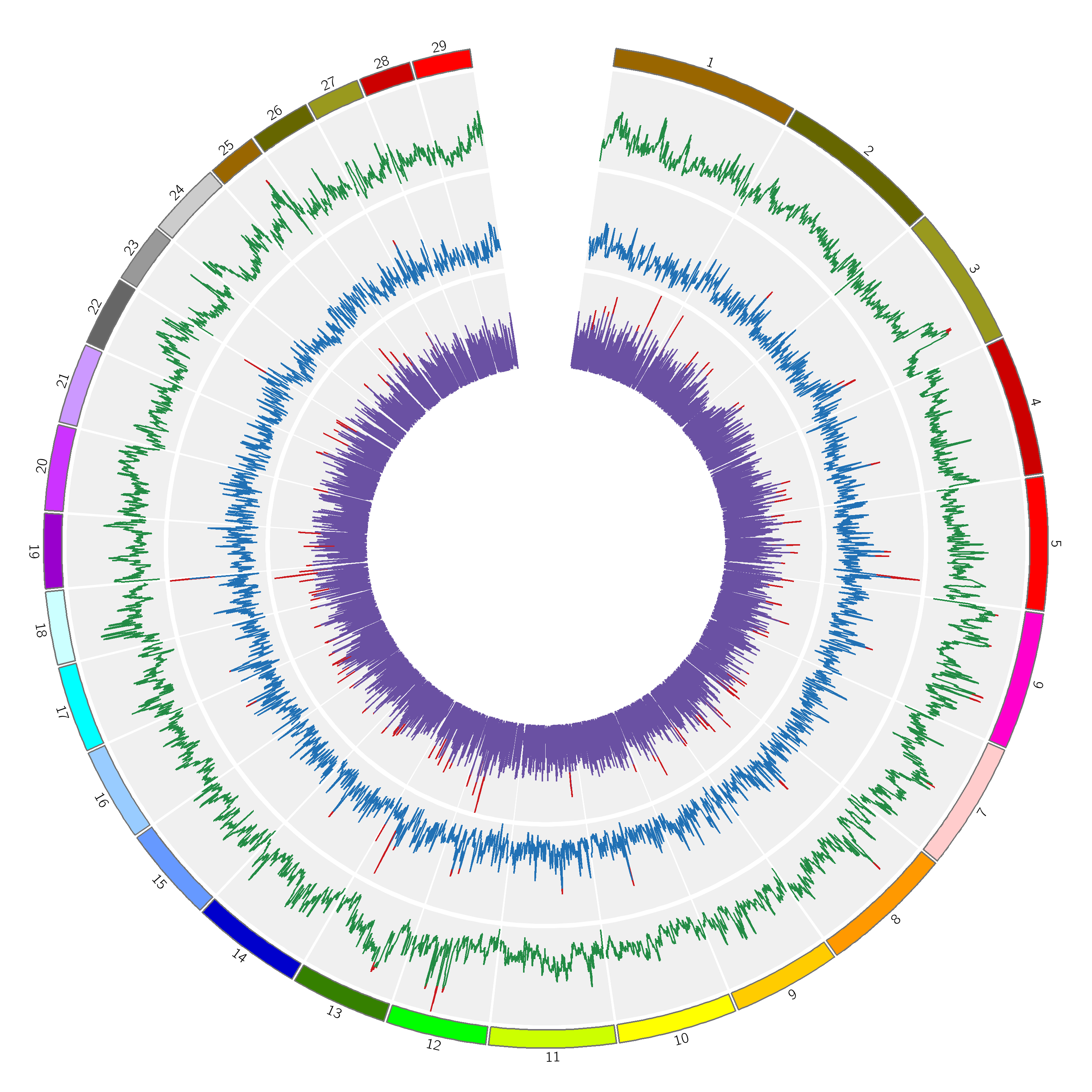


Figure S13

Title: ROH, *F*_ST_ and XP-EHH results for the group of “milk-producing” goat breeds

Description: Analysis types showed with different plot colors, within the most external squared-based circle, where each color represent a chromosome (chromosome number outside the squares): green (external) = ROH; blue (middle) = Fst; violet (internal): XP-EHH. For the three analyses, the regions above the threshold are marked in red.


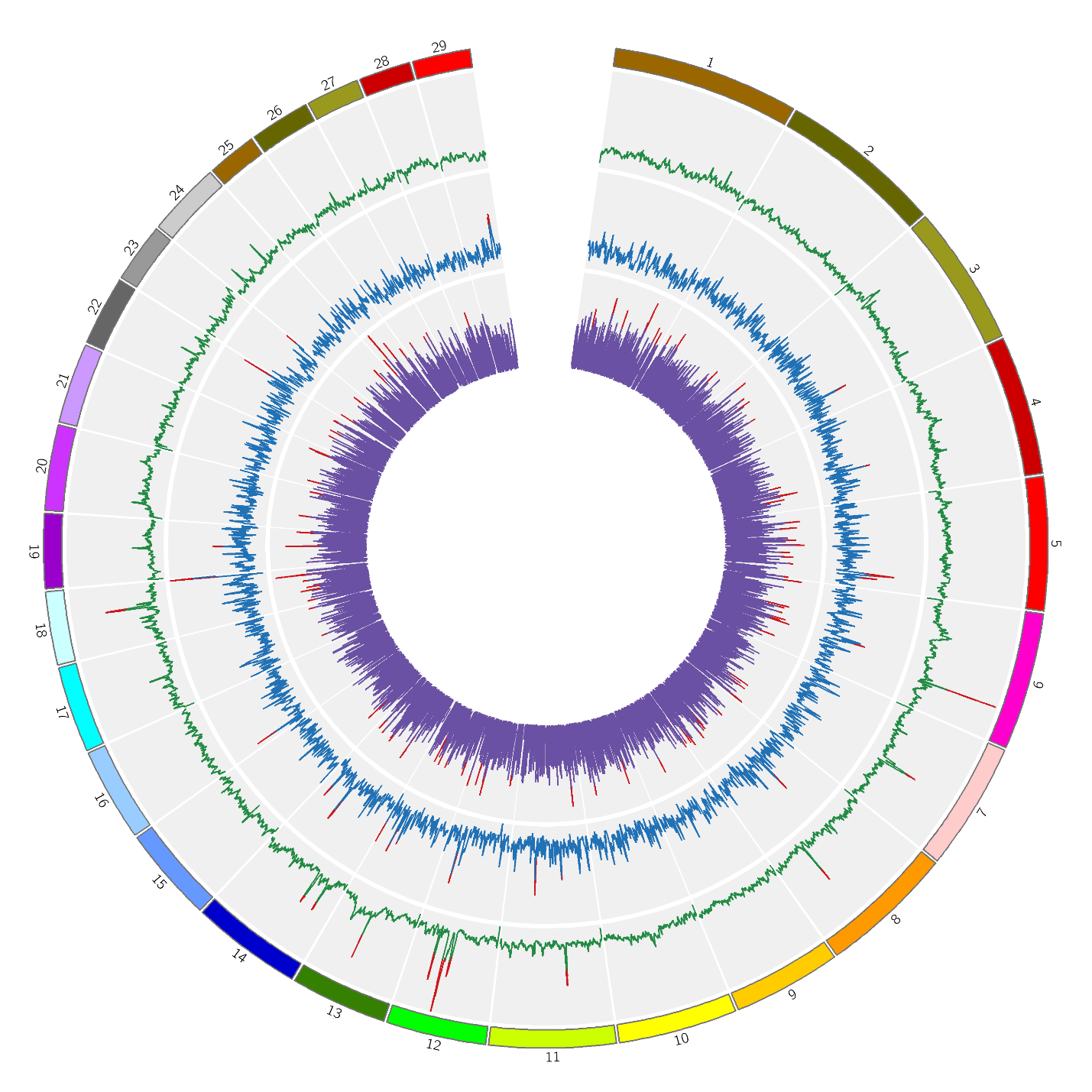


Figure S14

Title: CDA for the region on chromosome 25 detected for the group of “meat-producing” goat breeds.

Description: (a): LONG and (b) SHORT: (b). Left: CDA plot. Right: Correlation value of the SNPs used for the analyses for CAN1 and CAN2.


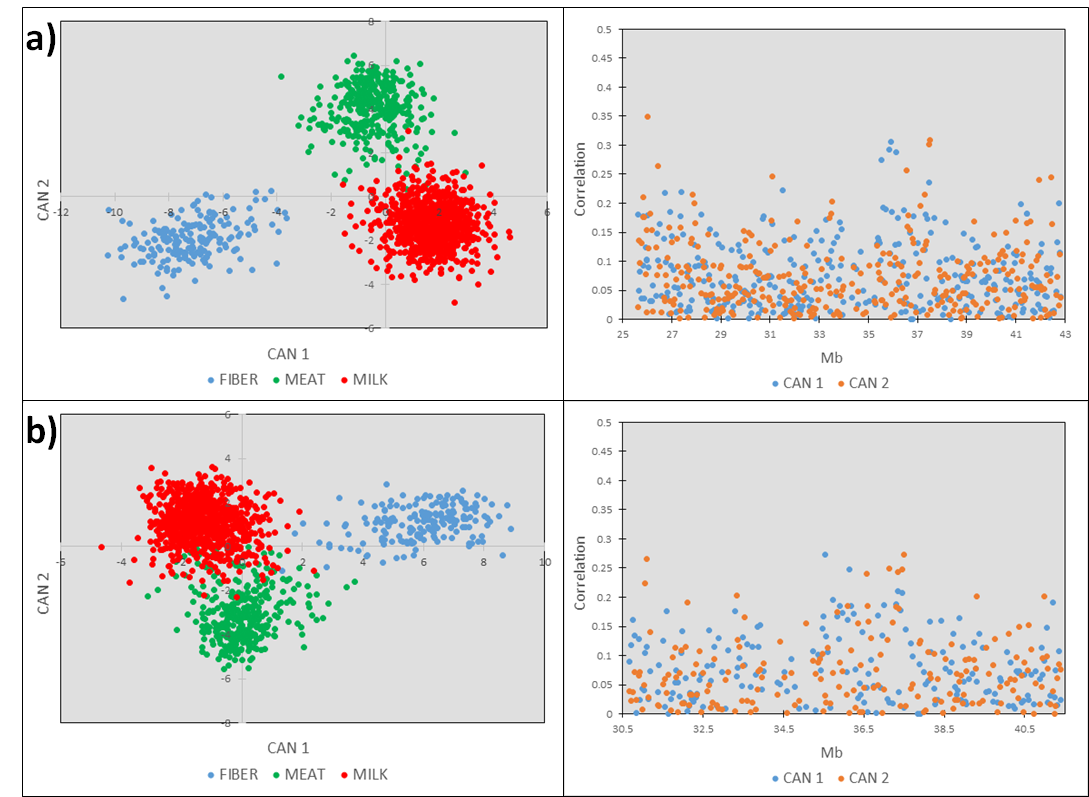


Figure S15

Title: CDA for the region on chromosome 25 detected for the group of “meat-producing” goat breeds.

Description: (a): LONG and (b) SHORT: (b). Left: CDA plot. Right: Correlation value of the SNPs used for the analyses for CAN1 and CAN2.


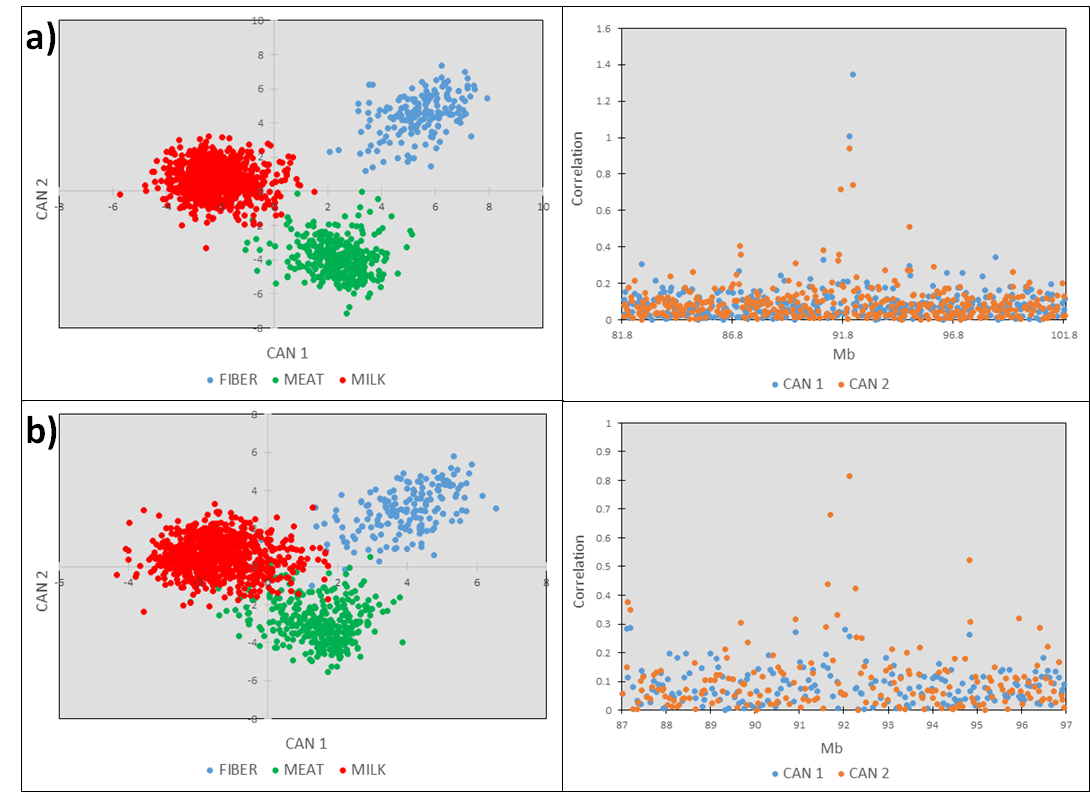


Figure S16

Title: CDA for the region on chromosome 25 detected for the group of “milk-producing” goat breeds.

Description: (a): LONG and (b) SHORT: (b). Left: CDA plot. Right: Correlation value of the SNPs used for the analyses for CAN1 and CAN2.


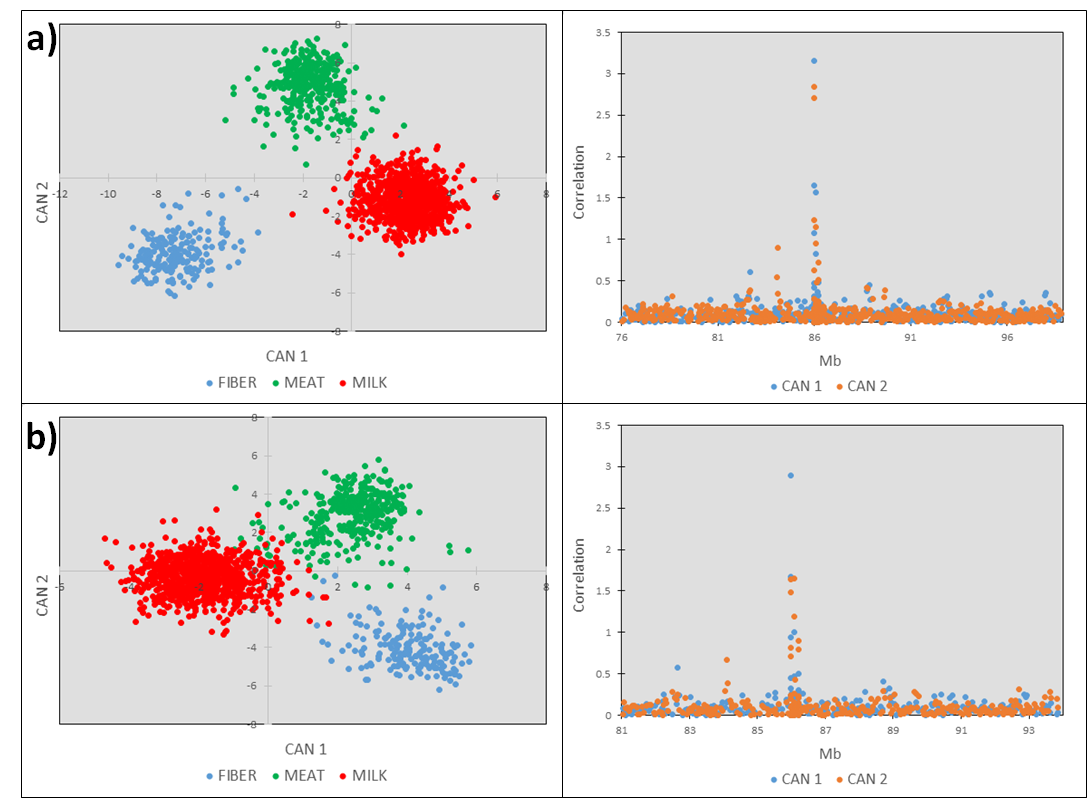


Figure S17

Title: MDS plot of breeds considered for the panel of coat colors

Description: Breed codes and subdivision based on the coat color pattern are indicated in the right part of the plot.


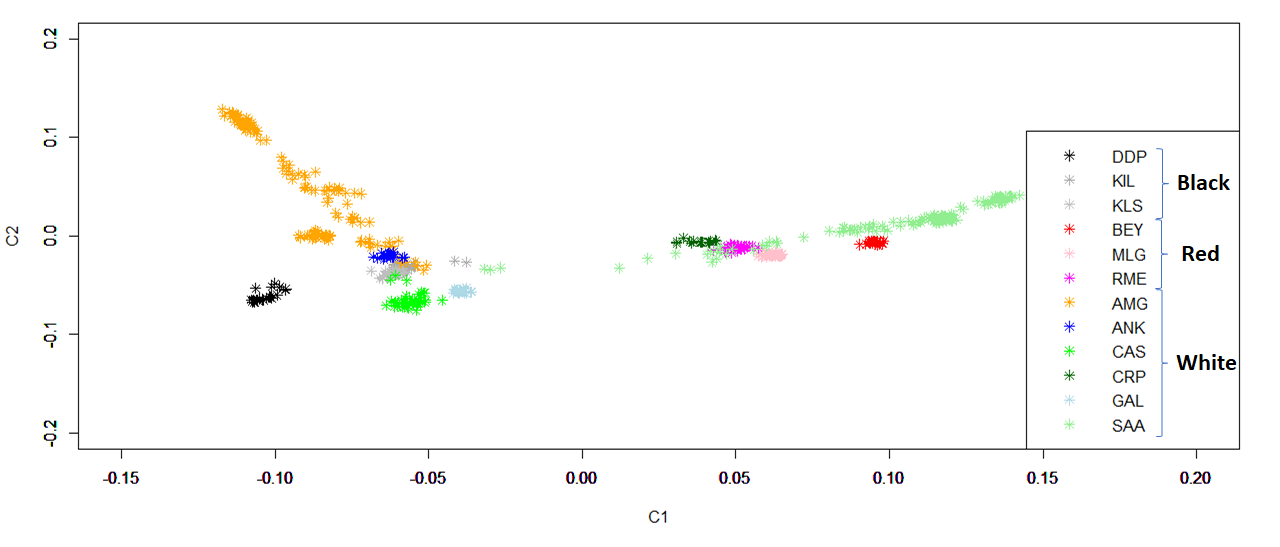


Figure S18

Title: CDA for the region on chromosome 18 near the *MC1R* gene detected for the group of coat color breeds.

Description: (a): LONG and (b) SHORT: (b). Left: CDA plot. Right: Correlation value of the SNPs used for the analyses for CAN1 and CAN2.


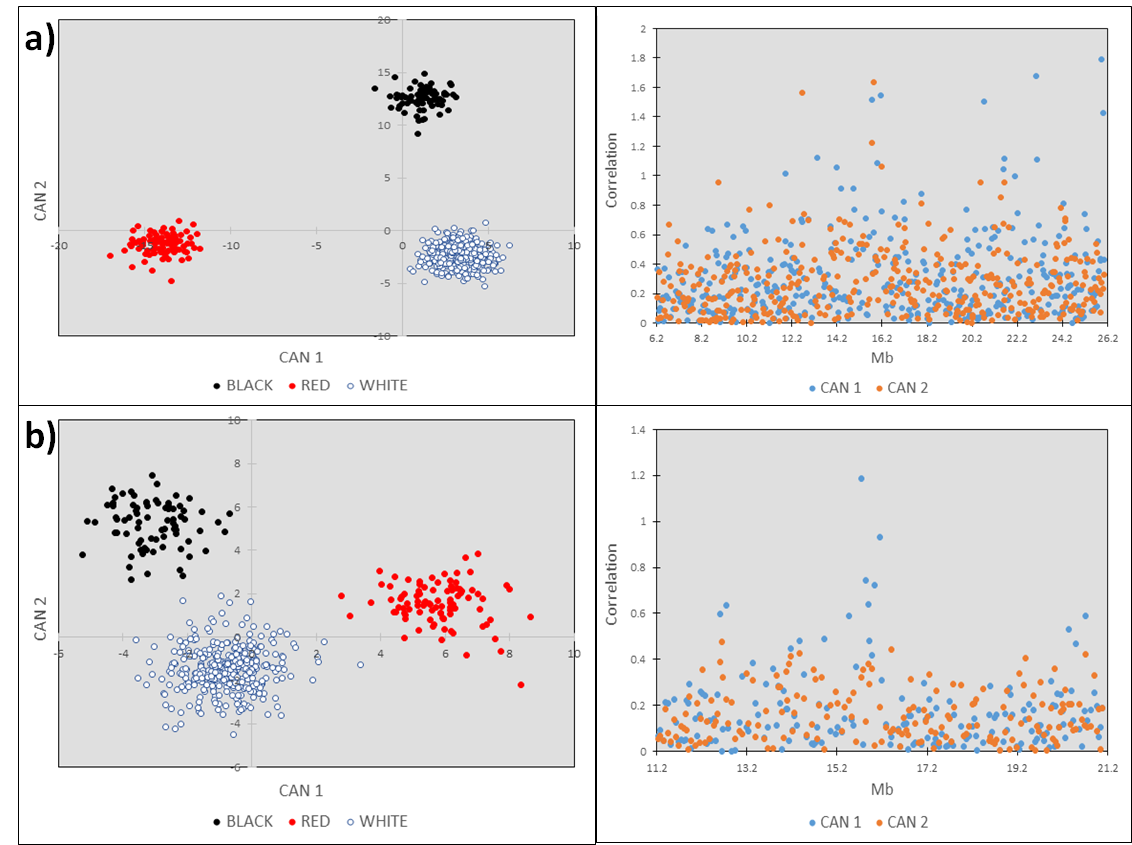


Figure S19

Title: CDA for the region on chromosome 13 near the *ASIP* gene detected for the group of coat color breeds.

Description: (a): LONG and (b) SHORT: (b). Left: CDA plot. Right: Correlation value of the SNPs used for the analyses for CAN1 and CAN2.


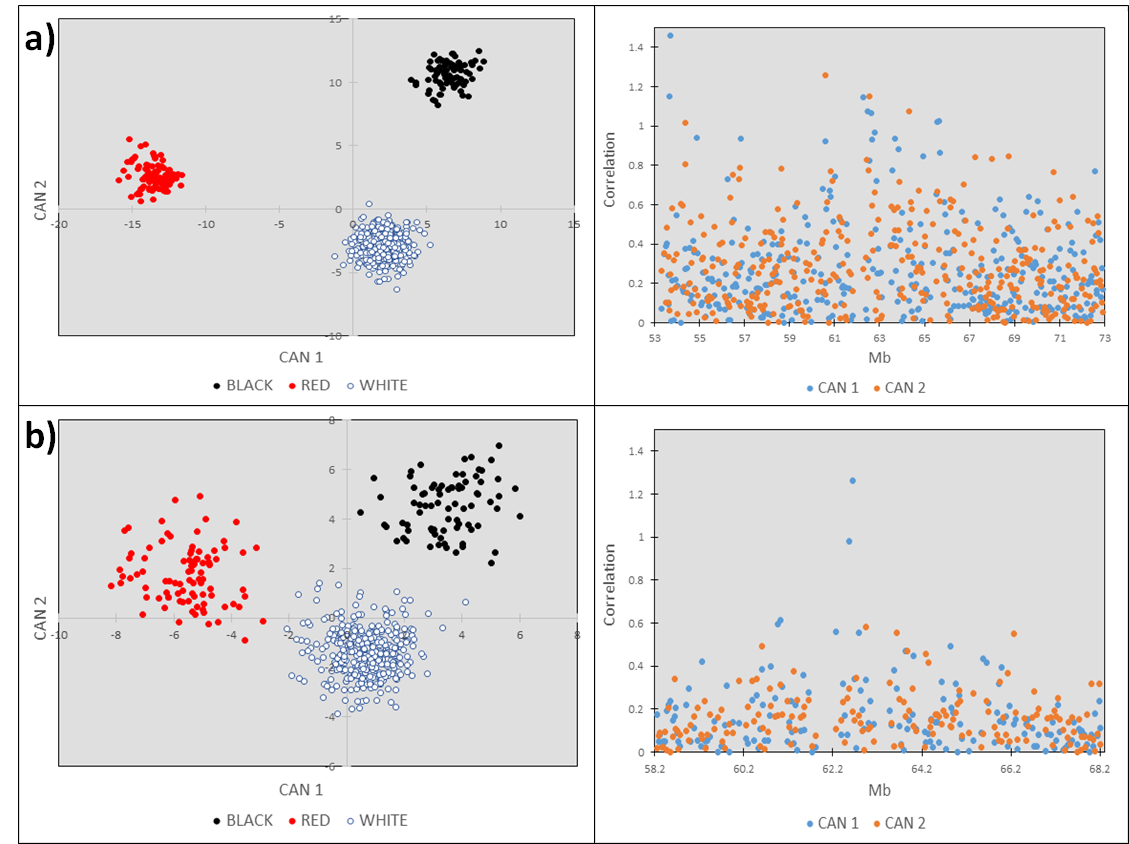


Figure S20

Title: CDA for the region on chromosome 5 near the *ADAMTS20* gene detected for the group of coat color breeds.

Description: (a): LONG and (b) SHORT: (b). Left: CDA plot. Right: Correlation value of the SNPs used for the analyses for CAN1 and CAN2.


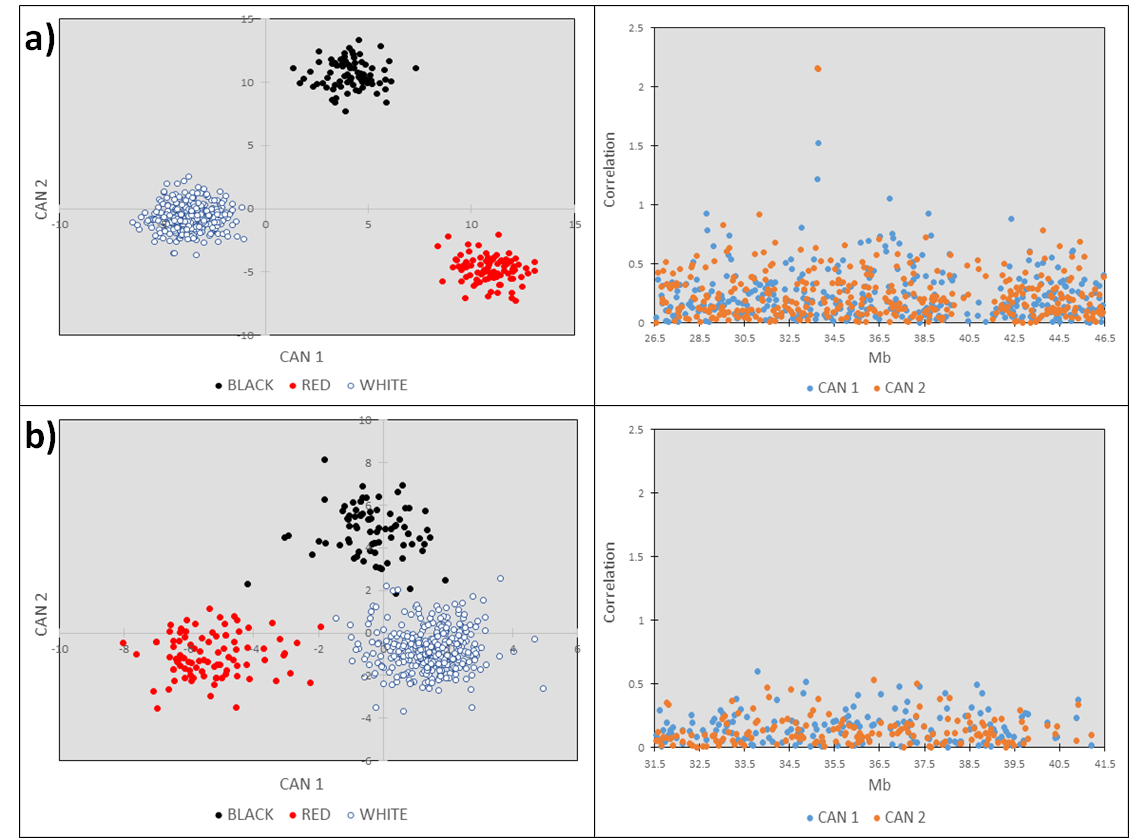


Figure S21

Title: MDS plot of the filtered dataset considering components 1 and 2. Animals are colored based on the Köppen group: Tropical (green), Dry (red), Temperate (orange), Continental (blue)

Description: Animals are color-coded based on the Köppen classification of groups: Tropical (green), Dry (red), Temperate (orange), Continental (blue).


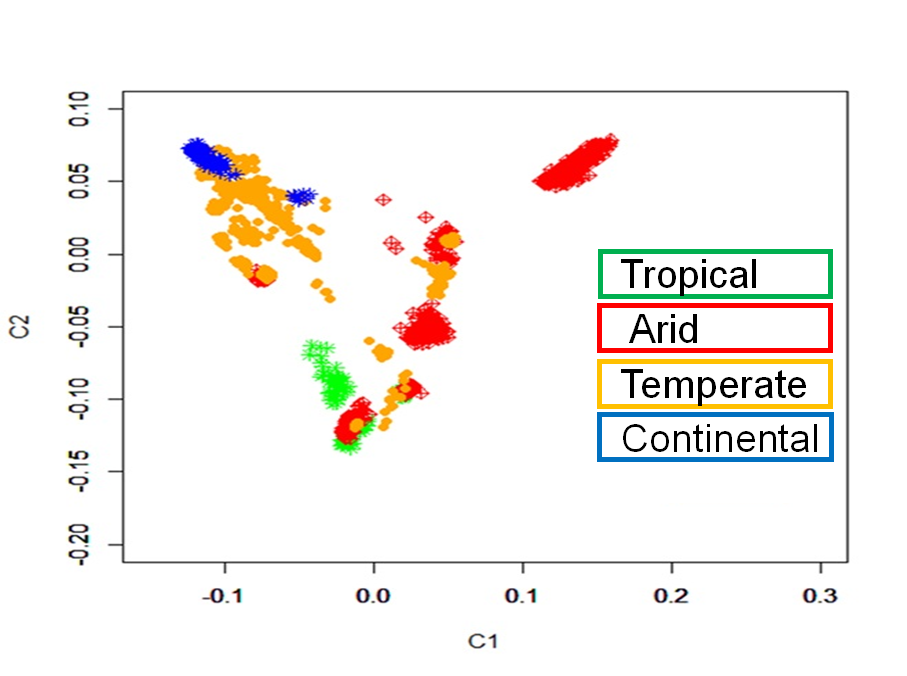


Figure S22

Title: *F*_ST_ plot of the comparison of the Dry group vs. the other groups.

Description: The threshold line in red represents the 0.995 of the percentile distribution (*F*_ST_ = 0.398).


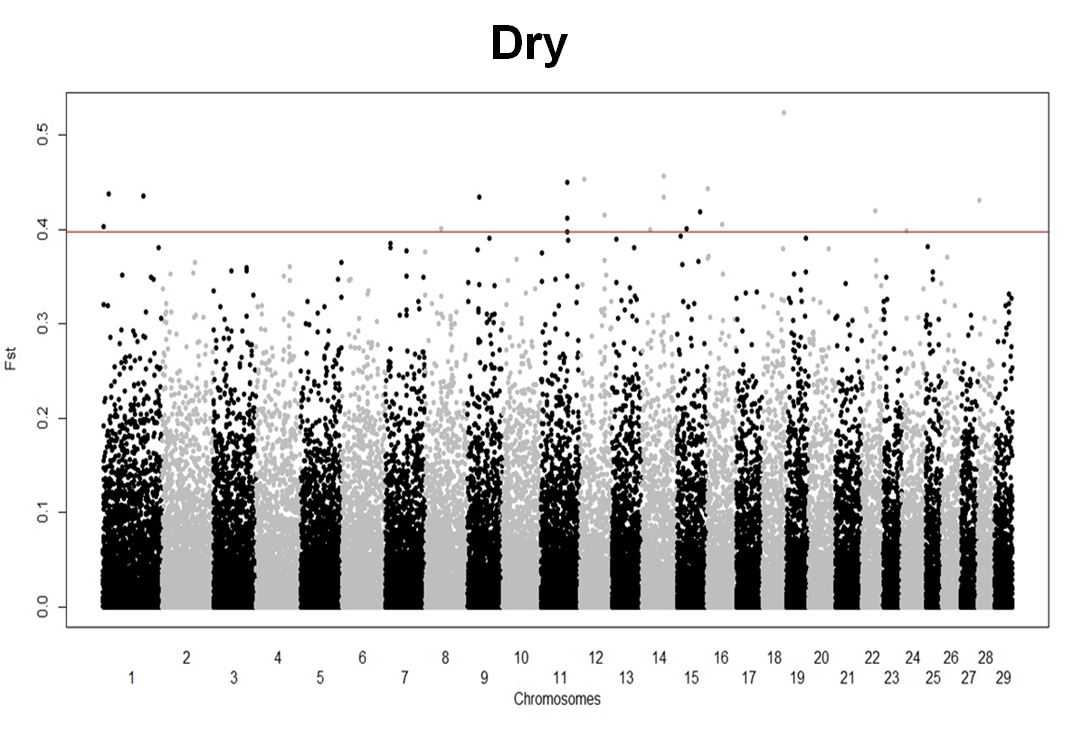


Figure S23

Title: *F*_ST_ plot of the comparison of the Temperate group vs. the other groups.

Description: The threshold line in red represents the 0.995 of the percentile distribution (*F*_ST_ = 0.320).


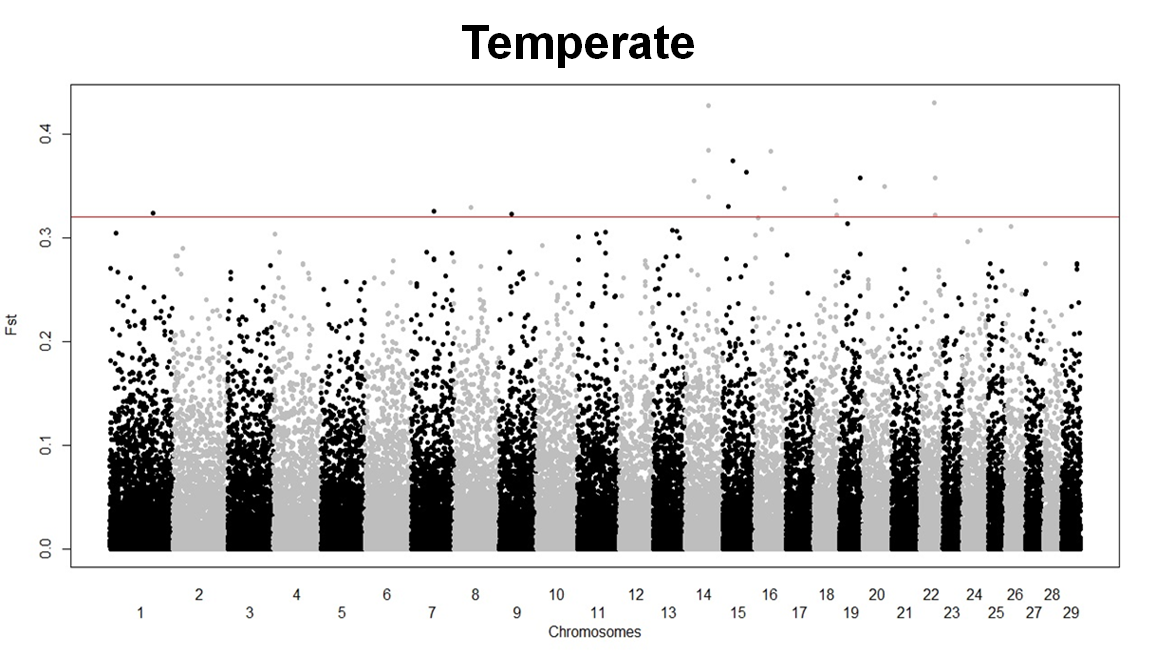


Figure S24

Title: *F*_ST_ plot of the comparison of the Continental group vs. the other groups.

Description: The threshold line in red represents the 0.995 of the percentile distribution (*F*_ST_ = 0.507).


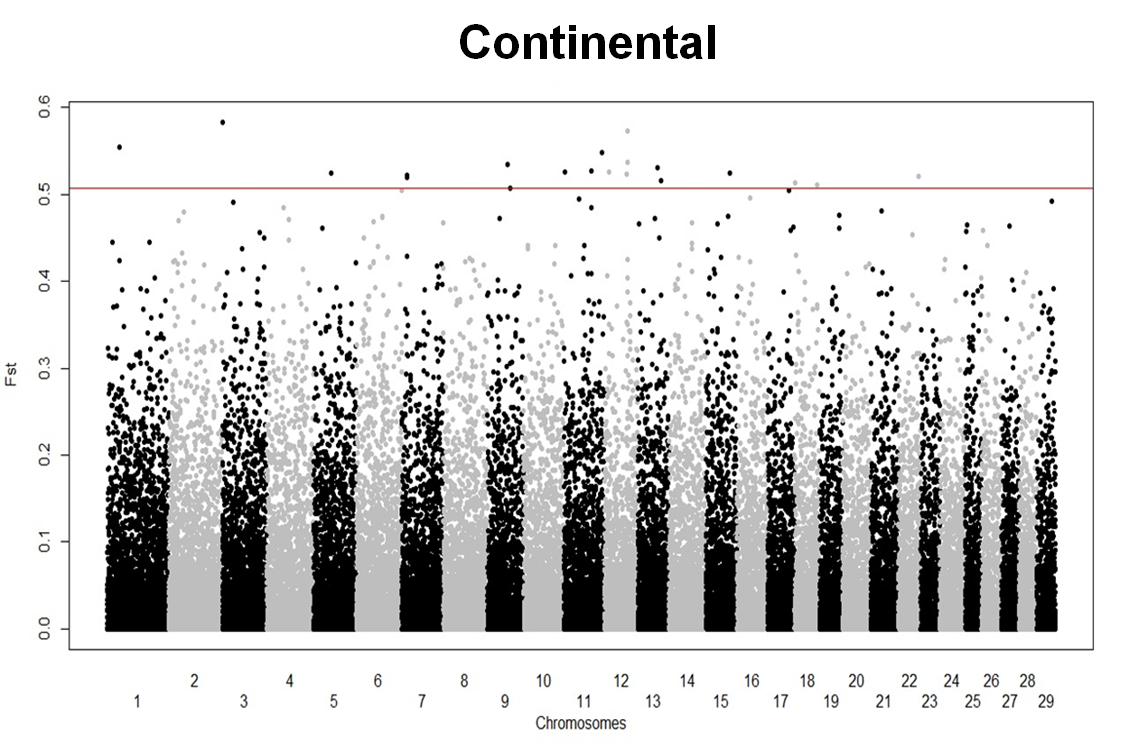

Supplement: Supplementary file 2 — Additional file 2: Figure S1. MDS plot of the breeds, grouped by production purpose: milk, meat and fiber groups. Group colors: milk = green, meat = red, fiber = blue; MDS and box plots of the first two components pre-filtering (upper) and MDS plots after filtering. Figure S2. Manhattan plot of the FLK results for the sub-continental group after filtering steps. Sub-geographical group names are given on top of each plot. Chromosomes are alternately red and black. Figure S3. Manhattan plot of the hapFLK results for the sub-continental group after filtering steps. Sub-geographical group names are given on top of each plot. Chromosomes are alternately red and black. Figure S4. Genomic distribution of FLK and hapFLK signals across population groups. For each chromosome and each sub-continental group, the chromosomal position detected with at least one of the two approaches is indicated. Figure S5. Signatures of selection on chromosome 5 for the North western Africa, South eastern Africa and South western Africa groups. North western Africa (NWA): red; South eastern Europe (SEE): green; South western Europe (SWE): blue. The table (bottom-right) reports the genes within the region in which a signature was detected. Figure S6. Signatures of selection on chromosome 6 for the Central Asia, East Africa and South east Europe groups. Central Asia (CA): red; East Africa (EA): green; South east Europe (SEE): blue. The table (upper-right) reports the genes within the region in which a signature was detected. Figure S7. Signatures of selection on chromosome 13 for the Alpines and Central Asia groups. Alpines (Alps): red; Central Asia (CA): blue. The table (right) reports the genes within the region in which a signature was detected. Figure S8. Signatures of selection on chromosome 1 for the Alpines and South Western Europe groups. Alpines (Alps); red; South Western Europe (SWE): blue. The table (bottom-right) reports the genes within the region in which a signature was detected. Fi [file 12711_2018_421_MOESM2_ESM.docx]
