# Supplementary material for: Signatures of selection and environmental adaptation across the goat genome post-domestication
Source: Genet Sel Evol. 2018 Nov 19;50:57. doi: 10.1186/s12711-018-0421-y (PMC6240954; doi:10.1186/s12711-018-0421-y)
Supplement: Supplementary file 3 — Additional file 3: Table S1. Environmental variables considered for the landscape genomic analysis. Table S2. FLK and hapFLK windows for the sub-continental groups after filtering steps. Overlapping or partially overlapping regions across the different geographical subdivisions are reported with the same letter as used for the population group (from a to m). Table S3. Selective sweeps of early adaptation. The bold values indicate overlap or partial overlap between comparisons. Table S4 Common regions between ROH and XP-EHH and/or FST analyses for the group of “fiber-producing” goat breeds and genes within these regions. Table S5. Common regions between ROH and XP-EHH and/or FST analyses for the group of “meat-producing” goat breeds and genes within these regions. Table S6. Common regions between ROH and XP-EHH and/or FST analyses for the group of “milk-producing” goat breeds and genes within these regions. Table S7. Top four regions detected for the coat color groups (Black, White and Red), indicated comparisons between the groups and genes located nearby these regions. Table S8. Results of the most significant associations involving the 57 filtered SNPs. For each SNP: SNP probe, genotype, genomic coordinate, associated environmental variable (“Env”) and scores and statistical test output values (“Gscore”, “WaldScore”, “Efron”, “AIC”, “Beta_0” and “Beta_1”) are reported. Table S9. List of genes located nearby (± 100 kb) the 57 filtered SNPs obtained by the landscape genomics analysis. Table S10. GO term biological processes. Table S11. MAF (minor allele frequency) and major allele of the 13 SNPs shared by Dry and Temperate/Continental groups. Table S12. Samβada significant results for the SNPs of the FST analyses. For each SNP probe: chromosome and position are reported, as well as Environment, G score, Wald Score, AIC, Abs_Beta1 and the Köppen group in which the SNP was detected by the FST analyses. Table S13. List of genes located nearby (± 100 kb) of the 65 SNPs [file 12711_2018_421_MOESM3_ESM.docx]

**Additional file 3**

**Supporting tables**

**Signatures of selection and environmental adaptation across the goat genome post domestication.**

Francesca Bertolini, Bertrand Servin, Andrea Talenti, Estelle Rochat, Eui Soo Kim, Claire Oget, Isabelle Palhière, Alessandra Crisà, Gennaro Catillo, Roberto Steri, Marcel Amills, Licia Colli, Gabriele Marras, Marco Milanesi, Ezequiel Nicolazzi, Benjamin D Rosen, Curtis P Van Tassell, Bernt Guldbrandtsen, Tad S Sonstegard, Gwenola Tosser-Klopp, Alessandra Stella, Max F Rothschild, Stéphane Joost, Paola Crepaldi and the ADAPTmap consortium

Table S1

Title: Environmental variables considered for the landscape genomic analysis

| **CODE** | **Environmental variable** |
| --- | --- |
| bio1 | Annual Mean Temperature |
| bio2 | Mean Diurnal Range (Mean of monthly (max temp - min temp) |
| bio3 | Isothermality (BIO2/BIO7) (* 100) |
| bio4 | Temperature Seasonality (standard deviation *100) |
| bio5 | Max Temperature of Warmest Month |
| bio6 | Min Temperature of Coldest Month |
| bio7 | Temperature Annual Range (BIO5-BIO6) |
| bio8 | Mean Temperature of Wettest Quarter |
| bio9 | Mean Temperature of Driest Quarter |
| bio10 | Mean Temperature of Warmest Quarter |
| bio11 | Mean Temperature of Coldest Quarter |
| bio12 | Annual Precipitation |
| bio13 | Precipitation of Wettest Month |
| bio14 | Precipitation of Driest Month |
| bio15 | Precipitation Seasonality (Coefficient of Variation) |
| bio16 | Precipitation of Wettest Quarter |
| bio17 | Precipitation of Driest Quarter |
| bio18 | Precipitation of Warmest Quarter |
| bio19 | Precipitation of Coldest Quarter |

Table S2

Title: FLK and hapFLK windows for the sub-continental groups after filtering steps

Description: . Overlapping or partially overlapping regions across the different geographical subdivisions are reported with the same letter as used for the population group (from ^a^ to ^m^).

| Chromosome | Begin | End | Max | Population group | Significant test |
| --- | --- | --- | --- | --- | --- |
| 1 | 113218602 | 120984378 | 118392825 | Alps^a^ | hapFLK-FLK |
| 5 | 110987414 | 112987414 | 111987414 | Alps^b^ | FLK |
| 6 | 83714029 | 86010332 | 85633238 | Alps^c^ | hapFLK-FLK |
| 7 | 85816424 | 93832043 | 90554967 | Alps | hapFLK |
| 10 | 26650280 | 28650280 | 27650280 | Alps | FLK |
| 13 | 50164018 | 52871150 | 51862548 | Alps | hapFLK |
| 13 | 55195097 | 68264381 | 63117523 | Alps^d^ | hapFLK-FLK |
| 14 | 83528638 | 85528638 | 84528638 | Alps | FLK |
| 24 | 54147826 | 57304785 | 55516749 | Alps^e^ | hapFLK |
| 25 | 35499729 | 35875779 | 35875779 | Angoras | FLK |
| 3 | 87639747 | 89364906 | 88858645 | Boers | hapFLK |
| 15 | 43352658 | 43792940 | 43694682 | Boers | hapFLK |
| 18 | 25071336 | 28245263 | 27362382 | Boers | hapFLK |
| 18 | 31198730 | 35000522 | 32396235 | Boers | hapFLK |
| 22 | 37656735 | 40272340 | 39826427 | Boers | hapFLK |
| 6 | 69276841 | 72152335 | 70834068 | CentralAsia^f^ | hapFLK |
| 10 | 35064301 | 41683768 | 37422911 | CentralAsia | hapFLK |
| 12 | 57217560 | 57876029 | 57590836 | CentralAsia^g^ | hapFLK |
| 13 | 60601288 | 65827525 | 62900048 | CentralAsia^d^ | hapFLK |
| 6 | 44625340 | 46625340 | 45625340 | EastAfrica^h,i^ | FLK |
| 6 | 70758117 | 70788808 | 70788808 | EastAfrica^f^ | FLK |
| 6 | 84532845 | 86532845 | 85532845 | EastAfrica^c^ | FLK |
| 13 | 60744929 | 62744929 | 61744929 | EastAfrica^d^ | FLK |
| 16 | 9665432 | 11665432 | 10665432 | EastAfrica | FLK |
| 21 | 18565570 | 20565570 | 19565570 | EastAfrica | FLK |
| 23 | 11699427 | 13699427 | 12699427 | EastAfrica | FLK |
| 2 | 48414819 | 50414819 | 49414819 | Egypt | FLK |
| 5 | 105348578 | 114048603 | 108343813 | Egypt^b,l^ | hapFLK |
| 6 | 12273179 | 14699830 | 13901525 | Egypt | hapFLK-FLK |
| 6 | 26839183 | 56407325 | 38874120 | Egypt^i,j^ | hapFLK |
| 16 | 4722376 | 8699610 | 6275371 | Egypt | hapFLK |
| 25 | 17956639 | 22233001 | 19270749 | Egypt | hapFLK |
| 3 | 109932435 | 111932435 | 110932435 | NorthWestAfrica | FLK |
| 4 | 72141822 | 74141822 | 73141822 | NorthWestAfrica | FLK |
| 5 | 21547109 | 24855824 | 22691808 | NorthWestAfrica | hapFLK |
| 5 | 34522975 | 41204720 | 37304425 | NorthWestAfrica^k^ | hapFLK-FLK |
| 6 | 44596401 | 46596401 | 45596401 | NorthWestAfrica^h^ | FLK |
| 10 | 62054442 | 71807740 | 64695254 | NorthWestAfrica | hapFLK-FLK |
| 1 | 3966539 | 7779682 | 5460566 | SouthAfrica | hapFLK |
| 1 | 93356538 | 95356538 | 94356538 | SouthAfrica | hapFLK |
| 7 | 59341407 | 64948071 | 61128942 | SouthAfrica | hapFLK |
| 17 | 46054408 | 49062316 | 47225499 | SouthAfrica^m^ | hapFLK |
| 18 | 52729206 | 54627639 | 54339025 | SouthAfrica | hapFLK |
| 19 | 7826339 | 9843633 | 9193588 | SouthAfrica | hapFLK |
| 20 | 39575171 | 45511327 | 42361183 | SouthAfrica^n^ | hapFLK |
| 20 | 62097412 | 71781295 | 64056638 | SouthAfrica | hapFLK |
| 24 | 56647513 | 62183185 | 59103668 | SouthAfrica^e^ | hapFLK |
| 27 | 41378342 | 43498682 | 42236849 | SouthAfrica | hapFLK |
| 1 | 104689233 | 105366890 | 104816054 | SouthEastEurope | hapFLK |
| 4 | 19451901 | 22034923 | 20069558 | SouthEastEurope | hapFLK |
| 4 | 24581276 | 24677932 | 24677932 | SouthEastEurope | hapFLK |
| 5 | 33693610 | 36375339 | 35013574 | SouthEastEurope^k^ | hapFLK |
| 6 | 25577939 | 42622601 | 36018329 | SouthEastEurope^j^ | hapFLK |
| 6 | 69276841 | 71495823 | 70834068 | SouthEastEurope^f^ | hapFLK |
| 1 | 115631053 | 121977086 | 120588995 | SouthWestEurope^a^ | hapFLK |
| 1 | 152997608 | 157299677 | 153934330 | SouthWestEurope | hapFLK |
| 4 | 42274548 | 49845833 | 45000136 | SouthWestEurope | hapFLK |
| 5 | 26482234 | 38473200 | 36529333 | SouthWestEurope^k^ | hapFLK |
| 5 | 108677642 | 110677642 | 109677642 | SouthWestEurope^l^ | FLK |
| 6 | 28226788 | 40254762 | 34924411 | SouthWestEurope^j^ | hapFLK |
| 12 | 56346759 | 60001028 | 57445240 | SouthWestEurope^g^ | hapFLK-FLK |
| 16 | 36920184 | 37286479 | 37035994 | SouthWestEurope | hapFLK |
| 16 | 44337810 | 52775081 | 50852666 | SouthWestEurope | hapFLK |
| 17 | 47363443 | 51843758 | 48096733 | SouthWestEurope^m^ | hapFLK |
| 20 | 39090403 | 48445048 | 45922443 | SouthWestEurope^n^ | hapFLK |
| 25 | 4395270 | 6395270 | 5395270 | SouthWestEurope | FLK |
| 29 | 41725738 | 42566976 | 41786801 | SouthWestEurope | hapFLK |

Table S3

Title: Selective sweeps of early adaptation.

Description: The bold values indicate overlap or partial overlap between comparisons.

| chr | Pos | Pzero | FLK | Pvalue | Closest Gene | Genes within 100Kb |
| --- | --- | --- | --- | --- | --- | --- |
| 1 | 116330987 | 0.20243992411019 | 40.8163195173072 | 8.78053472576271e-07 | P2RY12 | IGSF10-MED12L-P2RY12-P2RY13-GPR87-P2RY14 |
| **1** | **131136001** | **0.825954204012401** | **37.3745352200742** | **3.98327722502836e-06** | **SOX14** |  |
| 1 | 135095840 | 0.9480349899512 | 31.0260528190341 | 6.14897559313364e-05 | SRPRB | RAB6B-SRPRB |
| 1 | 72849309 | 0.836406794333853 | 29.4618435038589 | 0.000119150624894026 | GP5 | ATP13A3-GP5-LRRC15-CPN2 |
| 10 | 23158052 | 0.758176242300796 | 67.4869546101362 | 4.74986680400392e-12 | RAD51B |  |
| **12** | **60710546** | **0.403418534473993** | **29.1685056900373** | **0.000134799100187092** | **MAB21L1** | **MAB21L1** |
| **12** | **60829727** | **0.410938258870259** | **29.9066794311595** | **9.87761009457367e-05** | **MAB21L1** |  |
| **12** | **60910543** | **0.250171598886531** | **48.8170086145011** | **2.46415749617372e-08** | **NBEA** |  |
| **12** | **60950668** | **0.22129699297368** | **54.3986866079607** | **1.96124219969914e-09** | **NBEA** | **DCLK1** |
| 13 | 53305564 | 0.21635708984519 | 29.9366429026218 | 9.75345292842498e-05 | ZBTB46 | UCKL1-DNAJC5-TPD52L2-ABHD16B-ZBTB46-SLC2A4RG-LIME1-ZGPAT-ARFRP1-TNFRSF6B-RTEL1-STMN3 |
| 13 | 61318983 | 0.118888456194633 | 31.9621401750269 | 4.12775638502332e-05 | NOL4L |  |
| 13 | 62468886 | 0.922388557910142 | 45.4712359283248 | 1.10773146364916e-07 | CDK5RAP1 | CDK5RAP1-SNTA1-CBFA2T2 |
| 13 | 7110576 | 0.828874278165878 | 38.3727058416131 | 2.57375375665053e-06 | ESF1 | ESF1-NDUFAF5 |
| **15** | **34039958** | **0.236940665137848** | **32.5131882744329** | **3.26166377594399e-05** | **HBBC** | **HBBC** |
| **15** | **34072318** | **0.842733102354674** | **55.6829750770018** | **1.09157210615564e-09** | **HBBC** | **HBBC** |
| 15 | 45808623 | 0.912682069720123 | 39.3696800787486 | 1.66136702922796e-06 | SOX6 | SOX6 |
| 15 | 65810847 | 0.865935980602249 | 35.0816566270844 | 1.07960576355235e-05 | GUCY1A2 |  |
| 15 | 75286045 | 0.948634813402488 | 32.6773109476782 | 3.04035897555708e-05 | TRPC6 |  |
| 16 | 36083298 | 0.169434182359299 | 35.7465001083516 | 8.09293012905215e-06 | SCYL3 | METTL18-C16H1orf112-SCYL3-KIFAP3 |
| 16 | 38963940 | 0.750657739637909 | 32.5732683221447 | 3.17886194042229e-05 | TNFSF18 |  |
| 17 | 10667314 | 0.23083644923108 | 32.6926540534449 | 3.02044537868434e-05 | RBM19 |  |
| **17** | **52433462** | **0.265108315209863** | **31.565369089668** | **4.88856453728757e-05** | **NOCT** | **NOCT-ELF2** |
| 17 | 56950210 | 0.938496579106259 | 29.9498713021296 | 9.69913057420269e-05 | FREM3 |  |
| 18 | 15219184 | 0.145150837124586 | 32.5616593259926 | 3.19469769780718e-05 | ZC3H18 | ZFPM1-ZC3H18-IL17C-MVD-SNAI3 |
| 18 | 1621854 | 0.0704575637923225 | 41.9914222691518 | 5.21977716647097e-07 | PRP6 | PRP6 |
| 18 | 56302975 | 0.095428188257938 | 54.177676176644 | 2.1690430809205e-09 | DBP | SULT2B1-FAM83E-SPACA4-RPL18-SPHK2-DBP-CA11-NTN5-MAMSTR-IZUMO1-FUT1 |
| 2 | 16174178 | 0.10557424640612 | 33.2980926711941 | 2.32965688840321e-05 | NPPC | NPPC-COPS7B-PDE6D |
| 20 | 23584452 | 0.922192949736416 | 28.5214981465137 | 0.000176829406217032 | SLC38A9 | SLC38A9-PLPP1 |
| 21 | 31425517 | 0.867282797188101 | 29.4230220332924 | 0.000121113924579565 | SCAPER |  |
| 22 | 20172811 | 0.482341525913204 | 28.5814472098886 | 0.000172446018969647 | GRM7 |  |
| 22 | 30130952 | 0.173230687337749 | 30.3637734481789 | 8.14230372870018e-05 | FOXP1 |  |
| 24 | 12176434 | 0.914990733825944 | 30.707041079739 | 7.04038767251272e-05 | SYT4 |  |
| 25 | 184627 | 0.113181715135158 | 37.7001547909789 | 3.4549363539503e-06 | LUC7L | MPG-NPRL3-HBM-LUC7L-FAM234A-RGS11-ARHGDIG-PDIA2-AXIN1 |
| 25 | 27019108 | 0.667527521434049 | 28.5098063808392 | 0.000177696991915736 | STX1B | FBXL19-ORAI3-SETD1A-HSD3B7-STX1B-STX4-ZNF668-ZNF646-PRSS53-VKORC1-BCKDK-KAT8-PRSS8-PRSS36 |
| 26 | 26946545 | 0.87935965400185 | 31.5724351606504 | 4.87387373003843e-05 | CFAP43 | ITPRIP-GSTO2-CFAP43 |
| 26 | 7797585 | 0.634960134248341 | 31.7297794323717 | 4.55781362592875e-05 | FAM53B | LHPP |
| 3 | 1091508 | 0.134509399632877 | 51.7992891698456 | 6.39521220174461e-09 | GPR35 | GPR35-CAPN10-RNPEPL1-DUSP28-ANKMY1-GPC1 |
| 3 | 27914663 | 0.0620312913529452 | 65.8048831197879 | 1.03601766598853e-11 | LRP8 | LRP8 |
| 3 | 62157548 | 0.905327332563561 | 32.0785785425982 | 3.92759015668755e-05 | WDR63 | MCOLN3-WDR63 |
| **4** | **18647771** | **0.265636749755495** | **43.1996375615041** | **3.05223773909421e-07** | **AKR1D1** | **AKR1D1-CREB3L2** |
| **4** | **18709407** | **0.55451993061206** | **29.4052737326993** | **0.000122022092524285** | **CREB3L2** | **CREB3L2** |
| 4 | 42973708 | 0.941438193323142 | 28.6406535772951 | 0.000168222040648331 | POLM | POLM-AEBP1-POLD2-MYL7-GCK-YKT6 |
| 4 | 69423893 | 0.825780938119433 | 33.9594126365631 | 1.75284807326854e-05 | CFTR | CFTR |
| **5** | **25748303** | **0.683057957183698** | **30.2872235818634** | **8.41034581069805e-05** | **HOXC6** | **HOXC4-HOXC5-HOXC6-HOXC8-HOXC9-HOXC10-HOXC11-HOXC12-HOXC13** |
| 5 | 47020710 | 0.186972110629511 | 31.5771161851265 | 4.86416561193079e-05 | LLPH | TMBIM4-LLPH |
| **5** | **68349535** | **0.715044448471026** | **29.1184504714566** | **0.000137664619958412** | **TCP11L2** | **CKAP4-TCP11L2-POLR3B** |
| **5** | **68382872** | **0.81418827832934** | **39.7466726813259** | **1.40739850205248e-06** | **TCP11L2** | **CKAP4-TCP11L2-POLR3B** |
| 6 | 115615071 | 0.929027233489983 | 30.4929352558233 | 7.70902458987969e-05 | RGS12 | LRPAP1-RGS12-HGFAC-DOK7 |
| ***6*** | ***45547830*** | ***0.0983336706181021*** | ***34.4681975309035*** | ***1.40749245902413e-05*** | ***SLC34A2*** | ***SLC34A2*** |
| ***6*** | ***45596401*** | ***0.118607209373144*** | ***29.5002113592852*** | ***0.000117241107506423*** | ***SLC34A2*** | ***SLC34A2-SEL1L3*** |
| ***6*** | ***45625340*** | ***0.238224878064338*** | ***33.3332916699173*** | ***2.29469743923607e-05*** | ***SLC34A2*** | ***SLC34A2-SEL1L3*** |
| **7** | **55549326** | **0.633085084309106** | **31.1954644342056** | **5.7218916880933e-05** | **YIPF5** |  |
| **7** | **55625453** | **0.603850388261615** | **34.8941621099636** | **1.17084673430808e-05** | **YIPF5** |  |
| 7 | 56083938 | 0.824180488847163 | 33.0279574149204 | 2.61608199648199e-05 | NR3C1 | NR3C1 |
| **7** | **63109676** | **0.574815232123456** | **34.875217572934** | **1.18048007668832e-05** | **LECT2** | **TGFBI-LECT2-FBXL21-IL9** |
| **7** | **63967869** | **0.787655381527292** | **34.1216566473954** | **1.63447419617206e-05** | **PITX1** | **PITX1** |
| 7 | 91112082 | 0.178437817673233 | 33.7288859393026 | 1.93576669069113e-05 | DPP9 | TNFAIP8L1-MYDGF-DPP9-FEM1A-TICAM1 |
| 7 | 93142001 | 0.908239245736905 | 30.9762655663571 | 6.28036894400152e-05 | ACTL9 | ACTL9 |
| 8 | 62425582 | 0.888879734825751 | 30.5032694591647 | 7.67535797857111e-05 | TMOD1 | TMOD1-TSTD2-NCBP1 |
| **8** | **68876819** | **0.281037420860708** | **30.3139383628731** | **8.31582825701016e-05** | **DOK2** | **DOK2-XPO7** |
| **8** | **68964508** | **0.182663662520953** | **31.0111469421021** | **6.18802632065678e-05** | **XPO7** | **DOK2-XPO7-NPM2-FGF17-DMTN** |

Table S4

Title: Common regions between ROH and XP-EHH and/or *F*_ST_ analyses for the group of “fiber-producing” goat breeds and genes within these regions.

| chr | Bp initial Window | Bp final Window | N. SNPs | gene start | gene end | Gene Symbol |
| --- | --- | --- | --- | --- | --- | --- |
| 6 | 70525829 | 71130414 | 13 | 71115093 | 71162022 | *KDR* |
|  |  |  |  | 70711232 | 70793908 | *KIT* |
| 18 | 14181623 | 14301292 | 3 | 14298744 | 14310906 | *C18H16orf95* |
| 18 | 14523113 | 14862060 | 6 | 14620550 | 14652253 | *KLHDC4* |
|  |  |  |  | 14698040 | 14726715 | *SLC7A5* |
|  |  |  |  | 14745215 | 14771479 | *CA5A* |
|  |  |  |  | 14541671 | 14621256 | *JPH3* |
|  |  |  |  | 14784395 | 14855786 | *BANP* |
| 18 | 22296173 | 22296174 | 1 | 22271152 | 22300390 | *VPS35* |
| 18 | 36966108 | 36966109 | 1 | 36944059 | 36982672 | *PRMT7* |
| 25 | 34691092 | 36429922 | 28 | 35137095 | 35142502 | *TMEM120A* |
|  |  |  |  | 35264989 | 35266715 | *HSPB1* |
|  |  |  |  | 35361750 | 35369513 | *ZP3* |
|  |  |  |  | 35488128 | 35492594 | *POLR2J* |
|  |  |  |  | 35492896 | 35499258 | *LRWD1* |
|  |  |  |  | 36324666 | 36329398 | *IFT22* |
|  |  |  |  | 36373629 | 36380450 | *CLDN15* |
|  |  |  |  | 36391764 | 36400126 | *PLOD3* |
|  |  |  |  | 35144486 | 35175362 | *STYXL1* |
|  |  |  |  | 35174573 | 35186838 | *MDH2* |
|  |  |  |  | 35204037 | 35257583 | *SRRM3* |
|  |  |  |  | 35287266 | 35311757 | *YWHAG* |
|  |  |  |  | 35333602 | 35347549 | *SSC4D* |
|  |  |  |  | 35499313 | 35506796 | *ALKBH4* |
|  |  |  |  | 35539698 | 35565667 | *PRKRIP1* |
|  |  |  |  | 35583306 | 35606399 | *SH2B2* |
|  |  |  |  | 36090367 | 36101773 | *MYL10* |
|  |  |  |  | 36369683 | 36373330 | *FIS1* |
|  |  |  |  | 36385061 | 36391660 | *ZNHIT1* |
|  |  |  |  | 35380089 | 35414612 | *DTX2* |
|  |  |  |  | 35508694 | 35523124 | *ORAI2* |
|  |  |  |  | 36129628 | 36297264 | *COL26A1* |
|  |  |  |  | 36422175 | 36444567 | *NAT16* |
|  |  |  |  | 35608593 | 35969965 | *CUX1* |

Table S5

Title: Common regions between ROH and XP-EHH and/or *F*_ST_ analyses for the group of “meat-producing” goat breeds and genes within these regions.

| chr | Bp initial Window | Bp final Window | N. SNPs | gene start | gene end | Gene Symbol |
| --- | --- | --- | --- | --- | --- | --- |
| 3 | 91030577 | 92500000 | 26 | 91114071 | 91122343 | *DCLRE1B* |
|  |  |  |  | 91200646 | 91203714 | *OLFML3* |
|  |  |  |  | 91980400 | 91990841 | *NRAS* |
|  |  |  |  | 91017438 | 91076182 | *PTPN22* |
|  |  |  |  | 91103643 | 91113968 | *AP4B1* |
|  |  |  |  | 91142964 | 91199543 | *HIPK1* |
|  |  |  |  | 91839388 | 91852778 | *BCAS2* |
|  |  |  |  | 91946176 | 91968861 | *AMPD1* |
|  |  |  |  | 92042544 | 92048250 | *SIKE1* |
|  |  |  |  | 92290631 | 92330433 | *TSPAN2* |
|  |  |  |  | 91090626 | 91097324 | *BCL2L15* |
|  |  |  |  | 91304621 | 91371433 | *SYT6* |
|  |  |  |  | 91626609 | 91773066 | *TRIM33* |
|  |  |  |  | 91853738 | 91943286 | *DENND2C* |
|  |  |  |  | 91990382 | 92025685 | *CSDE1* |
|  |  |  |  | 92104658 | 92251092 | *SYCP1* |
|  |  |  |  | 92272539 | 92277722 | *TSHB* |
| 18 | 12933010 | 12933011 | 1 | 12653900 | 13048675 | *GSE1* |

Table S6

Title: Common regions between ROH and XP-EHH and/or *F*_ST_ analyses for the group of “milk-producing” goat breeds and genes within these regions.

| chr | Bp initial Window | Bp final Window | N. SNPs | gene start | gene end | Gene Symbol |
| --- | --- | --- | --- | --- | --- | --- |
| 11 | 37591747 | 37591748 | 1 | 37573117 | 37597163 | *MTIF2* |
| 11 | 38000000 | 38610640 | 9 | 38225954 | 38226073 | *MIR217* |
|  |  |  |  | 38251608 | 38251693 | *MIR216B* |
|  |  |  |  | 38093732 | 38165206 | *EFEMP1* |
|  |  |  |  | 38443506 | 38665182 | *CCDC85A* |
| 13 | 63000000 | 63836533 | 9 | 63498937 | 63500710 | *MAP1LC3A* |
|  |  |  |  | 63619616 | 63629276 | *TP53INP2* |
|  |  |  |  | 63720758 | 63721591 | *LOC108637356* |
|  |  |  |  | 63062854 | 63082442 | *EIF2S2* |
|  |  |  |  | 63228709 | 63249542 | *ASIP* |
|  |  |  |  | 63264059 | 63279821 | *AHCY* |
|  |  |  |  | 63465549 | 63483202 | *DYNLRB1* |
|  |  |  |  | 63732342 | 63756692 | *GGT7* |
|  |  |  |  | 63760207 | 63804609 | *ACSS2* |
|  |  |  |  | 63805238 | 63829900 | *GSS* |
|  |  |  |  | 62969108 | 63053806 | *RALY* |
|  |  |  |  | 63363104 | 63462630 | *ITCH* |
|  |  |  |  | 63500918 | 63594821 | *PIGU* |
|  |  |  |  | 63630403 | 63721446 | *NCOA6* |
| 14 | 30115953 | 30216259 | 2 | 30144423 | 30240225 | *LOC108637476* |

Table S7

Title: Top four regions detected for the coat color groups (Black, White and Red), indicated comparisons between the groups and genes located nearby these regions.

| Comparison | Chr | win_start | win_end | gene_start | gene_end | gene_symbol |
| --- | --- | --- | --- | --- | --- | --- |
| *Black VS White* | 5 | 70000000 | 70500000 | 70075729 | 70134562 | *TIMP3* |
|  |  |  |  | 70481063 | 71091017 | *LARGE1* |
|  |  |  |  | 69807334 | 70282973 | *SYN3* |
|  | 13 | 53000000 | 53500000 | 53094654 | 53095711 | *NPBWR2* |
|  |  |  |  | 53113890 | 53114891 | *LKAAEAR1* |
|  |  |  |  | 53118290 | 53124458 | *RGS19* |
|  |  |  |  | 53125287 | 53132799 | *TCEA2* |
|  |  |  |  | 53143569 | 53145450 | *SOX18* |
|  |  |  |  | 53193878 | 53198630 | *SAMD10* |
|  |  |  |  | 53200864 | 53211439 | *ZNF512B* |
|  |  |  |  | 53352356 | 53355918 | *SLC2A4RG* |
|  |  |  |  | 53356593 | 53359342 | *LIME1* |
|  |  |  |  | 53369440 | 53376107 | *ARFRP1* |
|  |  |  |  | 53376787 | 53378322 | *TNFRSF6B* |
|  |  |  |  | 53401707 | 53411582 | *STMN3* |
|  |  |  |  | 53439490 | 53440850 | *FNDC11* |
|  |  |  |  | 53451694 | 53459922 | *PTK6* |
|  |  |  |  | 53000927 | 53064388 | *MYT1* |
|  |  |  |  | 53099266 | 53108772 | *OPRL1* |
|  |  |  |  | 53156897 | 53192582 | *PRPF6* |
|  |  |  |  | 53228440 | 53257795 | *DNAJC5* |
|  |  |  |  | 53261672 | 53277835 | *TPD52L2* |
|  |  |  |  | 53279463 | 53281144 | *ABHD16B* |
|  |  |  |  | 53359431 | 53369262 | *ZGPAT* |
|  |  |  |  | 53378620 | 53399261 | *RTEL1* |
|  |  |  |  | 53417414 | 53436555 | *GMEB2* |
|  |  |  |  | 53443100 | 53451287 | *SRMS* |
|  |  |  |  | 53459839 | 53461498 | *PPDPF* |
|  |  |  |  | 53495221 | 53543630 | *KCNQ2* |
|  |  |  |  | 53211523 | 53222894 | *UCKL1* |
|  |  |  |  | 53300718 | 53352160 | *ZBTB46* |
|  |  |  |  | 53475687 | 53483660 | *EEF1A2* |
|  | 18 | 15500000 | 16250000 | 16104704 | 16106439 | *MC1R** |
|  |  |  |  | 15451405 | 15529169 | *CBFA2T3* |
|  |  |  |  | 15607271 | 15651676 | *ACSF3* |
|  |  |  |  | 15663350 | 15682385 | *CDH15* |
|  |  |  |  | 15682552 | 15687167 | *SLC22A31* |
|  |  |  |  | 15730126 | 15847970 | *ANKRD11* |
|  |  |  |  | 15864475 | 15885588 | *SPG7* |
|  |  |  |  | 15891288 | 15894637 | *RPL13* |
|  |  |  |  | 15902244 | 15914398 | *CPNE7* |
|  |  |  |  | 15930964 | 15942253 | *DPEP1* |
|  |  |  |  | 15950502 | 15957489 | *CHMP1A* |
|  |  |  |  | 15957526 | 15975383 | *CDK10* |
|  |  |  |  | 15975384 | 15979076 | *SPATA2L* |
|  |  |  |  | 15979128 | 16007182 | *ZNF276* |
|  |  |  |  | 15982481 | 15992129 | *VPS9D1* |
|  |  |  |  | 16007155 | 16043913 | *FANCA* |
|  |  |  |  | 16045124 | 16078337 | *SPIRE2* |
|  |  |  |  | 16080645 | 16102527 | *TCF25* |
|  |  |  |  | 16121580 | 16138058 | *DEF8* |
|  |  |  |  | 16137777 | 16141183 | *CENPBD1* |
|  |  |  |  | 16162963 | 16170801 | *DBNDD1* |
| *White VS Black+Red* | 13 | 62750000 | 63250000 | 62814614 | 62861236 | *CHMP4B* |
|  |  |  |  | 63062854 | 63082442 | *EIF2S2* |
|  |  |  |  | 63228709 | 63249542 | *ASIP ** |
|  |  |  |  | 62761999 | 62803765 | *ZNF341* |
|  |  |  |  | 62969108 | 63053806 | *RALY* |
|  | 5 | 36250000 | 36750000 | 36409817 | 36611393 | *ADAMTS20** |
|  | 13 | 53000000 | 53500000 | 53094654 | 53095711 | *NPBWR2* |
|  |  |  |  | 53113890 | 53114891 | *LKAAEAR1* |
|  |  |  |  | 53118290 | 53124458 | *RGS19* |
|  |  |  |  | 53125287 | 53132799 | *TCEA2* |
|  |  |  |  | 53143569 | 53145450 | *SOX18 ** |
|  |  |  |  | 53193878 | 53198630 | *SAMD10* |
|  |  |  |  | 53200864 | 53211439 | *ZNF512B* |
|  |  |  |  | 53352356 | 53355918 | *SLC2A4RG* |
|  |  |  |  | 53356593 | 53359342 | *LIME1* |
|  |  |  |  | 53369440 | 53376107 | *ARFRP1* |
|  |  |  |  | 53376787 | 53378322 | *TNFRSF6B* |
|  |  |  |  | 53401707 | 53411582 | *STMN3* |
|  |  |  |  | 53439490 | 53440850 | *FNDC11* |
|  |  |  |  | 53451694 | 53459922 | *PTK6* |
|  |  |  |  | 53000927 | 53064388 | *MYT1* |
|  |  |  |  | 53099266 | 53108772 | *OPRL1* |
|  |  |  |  | 53156897 | 53192582 | *PRPF6* |
|  |  |  |  | 53228440 | 53257795 | *DNAJC5* |
|  |  |  |  | 53261672 | 53277835 | *TPD52L2* |
|  |  |  |  | 53279463 | 53281144 | *ABHD16B* |
|  |  |  |  | 53359431 | 53369262 | *ZGPAT* |
|  |  |  |  | 53378620 | 53399261 | *RTEL1* |
|  |  |  |  | 53417414 | 53436555 | *GMEB2* |
|  |  |  |  | 53443100 | 53451287 | *SRMS* |
|  |  |  |  | 53459839 | 53461498 | *PPDPF* |
|  |  |  |  | 53495221 | 53543630 | *KCNQ2* |
|  |  |  |  | 53211523 | 53222894 | *UCKL1* |
|  |  |  |  | 53300718 | 53352160 | *ZBTB46* |
|  |  |  |  | 53475687 | 53483660 | *EEF1A2* |
| *Red VS White* | 8 | 27000000 | 27500000 | 26770649 | 27126378 | *CNTLN* |
|  |  |  |  | 27347049 | 27833546 | *BNC2* |
|  | 22 | 2250000 | 3000000 | 2582220 | 2664523 | *CMC1* |
|  |  |  |  | 2667955 | 2704435 | *AZI2* |
|  |  |  |  | 2704097 | 2853095 | *ZCWPW2* |
|  | 29 | 39250000 | 39750000 | 39621921 | 39669514 | *APOOL* |

Table S8

Title: Results of the most significant associations involving the 57 filtered SNPs.

Description: For each SNP: SNP probe, genotype, genomic coordinate, associated environmental variable (“Env”) and scores and statistical test output values (“Gscore”, “WaldScore”, “Efron”, “AIC”, “Beta_0” and “Beta_1”) are reported.

| **Probe name** | **Gen.** | **Chr.** | **Position** | **Env** | **Gscore** | **WaldScore** | **Efron** | **AIC** | **Beta_0** | **Beta_1** |
| --- | --- | --- | --- | --- | --- | --- | --- | --- | --- | --- |
| snp12120-scaffold1448-789150 | AA | 17 | 8947492 | bio1 | 870.62 | 480.47 | 0.33 | 1577.00 | 2.63 | 1.98 |
| snp12120-scaffold1448-789150 | GG | 17 | 8947492 | bio14 | 174.39 | 161.06 | 0.05 | 873.90 | -3.51 | 1.01 |
| snp13612-scaffold1526-472688 | GG | 12 | 10476413 | bio1 | 738.50 | 482.96 | 0.29 | 1913.28 | 2.05 | 1.59 |
| snp14167-scaffold1566-1187146 | AA | 4 | 90982814 | bio1 | 556.41 | 372.35 | 0.30 | 1656.38 | 2.47 | 1.53 |
| snp15124-scaffold1614-514416 | GG | 9 | 20632 | bio1 | 645.14 | 410.22 | 0.24 | 1645.86 | 2.51 | 1.65 |
| snp1630-scaffold1046-1400739 | GG | 19 | 14819207 | bio1 | 605.17 | 406.42 | 0.22 | 1761.91 | 2.33 | 1.52 |
| snp1640-scaffold10469-208327 | GG | 13 | 53160922 | bio1 | 784.03 | 481.22 | 0.30 | 1762.86 | 2.28 | 1.73 |
| snp17046-scaffold1777-78487 | GG | 3 | 111943981 | bio1 | 774.66 | 490.95 | 0.29 | 1856.56 | 2.13 | 1.66 |
| snp18275-scaffold1855-155165 | GG | 4 | 6347244 | bio1 | 518.43 | 369.02 | 0.20 | 1799.52 | 2.27 | 1.39 |
| snp18290-scaffold1857-340926 | AA | 18 | 56302975 | bio1 | 806.68 | 496.97 | 0.32 | 1807.50 | 2.20 | 1.72 |
| snp18571-scaffold1878-249261 | CC | 26 | 14182177 | bio1 | 511.00 | 348.28 | 0.23 | 1619.58 | 2.55 | 1.49 |
| snp1858-scaffold1052-1225291 | AA | 14 | 24622151 | bio1 | 584.16 | 394.71 | 0.24 | 1734.12 | 2.36 | 1.51 |
| snp19202-scaffold1931-360047 | CC | 7 | 95367449 | bio1 | 429.09 | 293.54 | 0.23 | 1453.42 | 2.80 | 1.47 |
| snp19629-scaffold1983-289157 | GG | 14 | 91122307 | bio1 | 541.49 | 361.89 | 0.19 | 1619.09 | 2.57 | 1.53 |
| snp21944-scaffold2156-537302 | GG | 4 | 26776780 | bio1 | 695.59 | 455.60 | 0.25 | 1836.71 | 2.19 | 1.58 |
| snp22598-scaffold2239-125488 | GG | 5 | 98061330 | bio1 | 676.53 | 434.78 | 0.25 | 1733.27 | 2.36 | 1.63 |
| snp23118-scaffold2304-126585 | GG | 14 | 81291766 | bio11 | 552.08 | 362.93 | 0.21 | 1510.94 | 2.75 | 1.62 |
| snp23119-scaffold2304-159526 | AA | 14 | 81267067 | bio1 | 732.64 | 446.11 | 0.28 | 1658.95 | 2.48 | 1.75 |
| snp23120-scaffold2304-195841 | CC | 14 | 81230482 | bio1 | 724.18 | 448.14 | 0.27 | 1695.91 | 2.42 | 1.71 |
| snp24965-scaffold2564-131990 | CC | 3 | 1091508 | bio1 | 1177.55 | 618.80 | 0.44 | 1793.66 | 1.97 | 2.09 |
| snp24975-scaffold257-240573 | GG | 3 | 55922240 | bio1 | 812.98 | 501.34 | 0.31 | 1813.88 | 2.17 | 1.72 |
| snp26537-scaffold2765-185379 | GG | 29 | 50448626 | bio1 | 881.00 | 534.72 | 0.32 | 1867.44 | 2.06 | 1.76 |
| snp26988-scaffold287-681145 | GG | 2 | 17797516 | bio1 | 716.07 | 418.54 | 0.28 | 1529.79 | 2.75 | 1.84 |
| snp26989-scaffold287-727057 | AA | 2 | 17843753 | bio1 | 597.63 | 350.07 | 0.23 | 1373.25 | 3.06 | 1.82 |
| snp27730-scaffold296-191224 | AA | 24 | 12176434 | bio1 | 665.36 | 447.27 | 0.26 | 1882.67 | 2.13 | 1.52 |
| snp27993-scaffold30-2392105 | CC | 6 | 81955680 | bio1 | 936.50 | 536.54 | 0.36 | 1752.56 | 2.22 | 1.89 |
| snp28462-scaffold303-4402023 | GG | 19 | 34412900 | bio1 | 905.26 | 554.06 | 0.36 | 1935.29 | 1.91 | 1.74 |
| snp29393-scaffold318-794069 | AA | 9 | 53453711 | bio1 | 490.82 | 353.54 | 0.18 | 1782.37 | 2.30 | 1.36 |
| snp34929-scaffold416-2711266 | GG | 14 | 36927531 | bio1 | 793.05 | 486.38 | 0.32 | 1771.89 | 2.27 | 1.73 |
| snp3602-scaffold1111-963076 | GG | 11 | 49353940 | bio1 | 640.32 | 417.84 | 0.25 | 1727.12 | 2.39 | 1.59 |
| snp3630-scaffold1113-532595 | GG | 2 | 16174178 | bio1 | 938.04 | 532.71 | 0.35 | 1733.19 | 2.28 | 1.92 |
| snp38099-scaffold475-377998 | GG | 10 | 38273854 | bio1 | 613.87 | 402.04 | 0.24 | 1689.98 | 2.45 | 1.58 |
| snp38502-scaffold4864-10739 | GG | 25 | 41460436 | bio1 | 697.45 | 436.47 | 0.26 | 1689.17 | 2.44 | 1.69 |
| snp39015-scaffold494-5605944 | AA | 4 | 42973708 | bio1 | 707.71 | 432.80 | 0.28 | 1636.66 | 2.54 | 1.74 |
| snp39795-scaffold509-2468879 | GG | 28 | 13109332 | bio1 | 635.45 | 431.84 | 0.24 | 1866.54 | 2.15 | 1.50 |
| snp40187-scaffold512-1292428 | GG | 18 | 20337955 | bio1 | 493.44 | 329.63 | 0.23 | 1520.00 | 2.73 | 1.53 |
| snp40808-scaffold524-376781 | GG | 9 | 25627287 | bio1 | 536.66 | 363.43 | 0.20 | 1652.74 | 2.51 | 1.50 |
| snp41855-scaffold5454-26183 | AA | 16 | 78915310 | bio2 | 596.13 | 318.97 | 0.25 | 1415.97 | 3.08 | 1.92 |
| snp43454-scaffold579-4091420 | AA | 9 | 62239938 | bio1 | 684.63 | 448.84 | 0.25 | 1820.53 | 2.22 | 1.58 |
| snp43455-scaffold579-4131867 | AA | 9 | 62199365 | bio1 | 540.32 | 363.19 | 0.20 | 1632.57 | 2.53 | 1.52 |
| snp43684-scaffold585-2375977 | AA | 8 | 78374753 | bio1 | 1146.11 | 567.08 | 0.42 | 1586.95 | 2.45 | 2.25 |
| snp44803-scaffold609-3176060 | GG | 17 | 17982610 | bio1 | 582.43 | 395.88 | 0.22 | 1760.89 | 2.33 | 1.50 |
| snp46383-scaffold640-488411 | AA | 1 | 24332845 | bio2 | 498.17 | 328.19 | 0.19 | 1733.72 | 2.43 | 1.47 |
| snp46696-scaffold65-2591416 | GG | 1 | 106720093 | bio1 | 501.71 | 350.59 | 0.17 | 1687.69 | 2.45 | 1.43 |
| snp4913-scaffold1164-1343187 | AA | 18 | 1357647 | bio1 | 637.12 | 435.88 | 0.24 | 1897.77 | 2.11 | 1.48 |
| snp49766-scaffold710-2154639 | AA | 6 | 19118259 | bio1 | 532.02 | 363.88 | 0.20 | 1674.57 | 2.47 | 1.48 |
| snp51272-scaffold75-5110850 | GG | 14 | 56659218 | bio3 | 449.00 | 240.24 | 0.17 | 1513.94 | 3.06 | 2.01 |
| snp55752-scaffold864-3177522 | CC | 27 | 19963783 | bio1 | 578.70 | 372.23 | 0.21 | 1570.08 | 2.66 | 1.62 |
| snp56061-scaffold875-397187 | AA | 13 | 15504116 | bio1 | 470.88 | 329.01 | 0.19 | 1615.90 | 2.56 | 1.43 |
| snp57572-scaffold920-314791 | AA | 5 | 42374355 | bio1 | 801.05 | 502.58 | 0.31 | 1861.87 | 2.11 | 1.68 |
| snp59380-scaffold979-422092 | GG | 1 | 1361457 | bio2 | 566.47 | 333.22 | 0.23 | 1568.57 | 2.74 | 1.71 |
| snp6037-scaffold1213-90216 | GG | 5 | 5722249 | bio1 | 870.56 | 490.16 | 0.31 | 1626.24 | 2.52 | 1.93 |
| snp6177-scaffold1218-403823 | AA | 14 | 63784925 | bio11 | 524.30 | 357.22 | 0.18 | 1569.68 | 2.64 | 1.54 |
| snp6389-scaffold1226-48340 | CC | 26 | 134674 | bio11 | 521.62 | 344.87 | 0.24 | 1471.55 | 2.81 | 1.61 |
| snp6505-scaffold1230-1135091 | AA | 21 | 32270925 | bio1 | 622.79 | 421.37 | 0.23 | 1824.08 | 2.23 | 1.51 |
| snp7527-scaffold127-6291318 | AA | 2 | 22057270 | bio1 | 746.41 | 467.41 | 0.30 | 1768.64 | 2.29 | 1.69 |
| snp88-scaffold100-417512 | AA | 15 | 75951851 | bio1 | 495.54 | 351.39 | 0.17 | 1729.33 | 2.38 | 1.40 |

Table S9

Title: List of genes located nearby (± 100 kb) the 57 filtered SNPs obtained by the landscape genomics analysis

| Chr | pos | SNP | gene start | gene end | gene symbol |
| --- | --- | --- | --- | --- | --- |
| 1 | 1361457 | snp59380-scaffold979-422092 | 1240437 | 1273105 | *PAXBP1* |
|  |  |  | 1384166 | 1397045 | *C1H21orf59* |
|  |  |  | 1282061 | 1376560 | *SYNJ1* |
|  |  |  | 1439122 | 1545430 | *EVA1C* |
| 1 | 24332845 | snp46383-scaffold640-488411 | - | - | *-* |
| 1 | 106720093 | snp46696-scaffold65-2591416 | 106759308 | 106760256 | *C1H3orf80* |
|  |  |  | 106612851 | 106740367 | *IFT80* |
| 2 | 16174178 | snp3630-scaffold1113-532595 | 16144029 | 16148670 | *NPPC* |
|  |  |  | 16210787 | 16236131 | *COPS7B* |
|  |  |  | 15749236 | 16107599 | *DIS3L2* |
|  |  |  | 16236483 | 16290580 | *PDE6D* |
| 2 | 17797516 | snp26988-scaffold287-681145 | 17763215 | 17916591 | *TRIP12* |
| 2 | 17843753 | snp26989-scaffold287-727057 | 17763215 | 17916591 | *TRIP12* |
| 2 | 22057270 | snp7527-scaffold127-6291318 | 21958513 | 22214193 | *NYAP2* |
| 3 | 1091508 | snp24965-scaffold2564-131990 | 1099278 | 1110046 | *CAPN10* |
|  |  |  | 1116268 | 1124813 | *RNPEPL1* |
|  |  |  | 990417 | 1045907 | *KIF1A* |
|  |  |  | 1073344 | 1096435 | *GPR35* |
|  |  |  | 1127184 | 1131136 | *DUSP28* |
|  |  |  | 1190860 | 1218517 | *GPC1* |
|  |  |  | 1136915 | 1190488 | *ANKMY1* |
| 3 | 55922240 | snp24975-scaffold257-240573 | - | - | *-* |
| 3 | 111943981 | snp17046-scaffold1777-78487 | 111841452 | 111845962 | *TOMM40L* |
|  |  |  | 111845097 | 111850245 | *NR1I3* |
|  |  |  | 111905321 | 111910332 | *MPZ* |
|  |  |  | 111850326 | 111889608 | *PCP4L1* |
|  |  |  | 111946023 | 111974585 | *CFAP126* |
|  |  |  | 111913690 | 111944243 | *SDHC* |
| 4 | 6347244 | snp18275-scaffold1855-155165 | 6277276 | 6281636 | *CDK5* |
|  |  |  | 6282739 | 6286933 | *ASIC3* |
|  |  |  | 6263854 | 6275539 | *SLC4A2* |
|  |  |  | 6307622 | 6316498 | *ATG9B* |
|  |  |  | 6315627 | 6334075 | *NOS3* |
|  |  |  | 6349296 | 6386108 | *KCNH2* |
|  |  |  | 6288518 | 6305424 | *ABCB8* |
| 4 | 26776780 | snp21944-scaffold2156-537302 | 26751962 | 26799129 | *STRIP2* |
|  |  |  | 26559138 | 26686430 | *NRF1* |
|  |  |  | 26736264 | 26743259 | *SMKR1* |
|  |  |  | 26803940 | 26993967 | *AHCYL2* |
|  |  |  | 42961990 | 42970832 | *POLM* |
|  |  |  | 42997455 | 43005591 | *POLD2* |
|  |  |  | 43018388 | 43020852 | *MYL7* |
|  |  |  | 43023328 | 43059614 | *GCK* |
|  |  |  | 43069084 | 43078402 | *YKT6* |
|  |  |  | 42860328 | 42913183 | *BLVRA* |
|  |  |  | 42988005 | 42997363 | *AEBP1* |
| 4 | 90982814 | snp14167-scaffold1566-1187146 | 91031114 | 91035660 | *SP8* |
|  |  |  | 91056779 | 91172420 | *ABCB5* |
| 5 | 5722249 | snp6037-scaffold1213-90216 | 5624977 | 5791534 | *OSBPL8* |
| 5 | 42374355 | snp57572-scaffold920-314791 | 42098735 | 42378642 | *PTPRR* |
|  |  |  | 42379253 | 42500726 | *PTPRB* |
| 5 | 98061330 | snp22598-scaffold2239-125488 | - | - | *-* |
| 6 | 19118259 | snp49766-scaffold710-2154639 | 19037849 | 19038478 | *LIN28A* |
|  |  |  | 19178643 | 19194790 | *GIMD1* |
|  |  |  | 19199791 | 19227268 | *AIMP1* |
| 6 | 81955680 | snp27993-scaffold30-2392105 | -1 | -1 |  |
| 7 | 95367449 | snp19202-scaffold1931-360047 | 95439060 | 95445151 | *SPC24* |
|  |  |  | 95455530 | 95482690 | *KANK2* |
|  |  |  | 95284072 | 95373858 | *SMARCA4* |
|  |  |  | 95396295 | 95430738 | *LDLR* |
| 9 | 20632 | snp15124-scaffold1614-514416 | 104207 | 129767 | *MB21D1* |
| 9 | 25627287 | snp40808-scaffold524-376781 | 25667120 | 25676649 | *FAM229B* |
|  |  |  | 25699274 | 25714487 | *WISP3* |
|  |  |  | 25512853 | 25661234 | *LAMA4* |
|  |  |  | 25676711 | 25698552 | *TUBE1* |
| 9 | 53453711 | snp29393-scaffold318-794069 | 53315015 | 53933095 | *PTPRK* |
| 9 | 62199365 | snp43455-scaffold579-4131867 | 62080820 | 62115387 | *IL20RA* |
|  |  |  | 62170459 | 62188060 | *IL22RA2* |
|  |  |  | 62196587 | 62220421 | *IFNGR1* |
| 9 | 62239938 | snp43454-scaffold579-4091420 | 62170459 | 62188060 | *IL22RA2* |
|  |  |  | 62196587 | 62220421 | *IFNGR1* |
| 10 | 38273854 | snp38099-scaffold475-377998 | 38182899 | 38213975 | *SLC30A4* |
|  |  |  | 38295222 | 38348608 | *SQRDL* |
| 11 | 49353940 | snp3602-scaffold1111-963076 | 49450012 | 49528012 | *KCMF1* |
|  |  |  | 49197164 | 49389243 | *TCF7L1* |
| 12 | 10476413 | snp13612-scaffold1526-472688 | 10300844 | 10589442 | *DOCK9* |
| 13 | 15504116 | snp56061-scaffold875-397187 | 14934979 | 15489576 | *CELF2* |
|  |  |  | 15587282 | 15785634 | *USP6NL* |
| 13 | 49539282 | snp13033-scaffold150-2378517 | -1 | -1 |  |
| 13 | 53160922 | snp1640-scaffold10469-208327 | 53094654 | 53095711 | *NPBWR2* |
|  |  |  | 53113890 | 53114891 | *LKAAEAR1* |
|  |  |  | 53118290 | 53124458 | *RGS19* |
|  |  |  | 53125287 | 53132799 | *TCEA2* |
|  |  |  | 53143569 | 53145450 | *SOX18* |
|  |  |  | 53193878 | 53198630 | *SAMD10* |
|  |  |  | 53200864 | 53211439 | *ZNF512B* |
|  |  |  | 53224886 | 53224977 | *MIR1388* |
|  |  |  | 53000927 | 53064388 | *MYT1* |
|  |  |  | 53099266 | 53108772 | *OPRL1* |
|  |  |  | 53156897 | 53192582 | *PRPF6* |
|  |  |  | 53228440 | 53257795 | *DNAJC5* |
|  |  |  | 53211523 | 53222894 | *UCKL1* |
| 14 | 36927531 | snp34929-scaffold416-2711266 | 36889325 | 36966427 | *COLEC10* |
|  |  |  | -1 | -1 |  |
| 14 | 81230482 | snp23120-scaffold2304-195841 | 81166501 | 81168897 | *SMPD5* |
|  |  |  | 81186394 | 81188312 | *EXOSC4* |
|  |  |  | 81189485 | 81192839 | *GPAA1* |
|  |  |  | 81205652 | 81209620 | *SHARPIN* |
|  |  |  | 81209801 | 81212830 | *MAF1* |
|  |  |  | 81226123 | 81229696 | *HGH1* |
|  |  |  | 81250250 | 81250321 | *MIR1839* |
|  |  |  | 81136748 | 81165697 | *SPATC1* |
|  |  |  | 81168936 | 81186366 | *OPLAH* |
|  |  |  | 81212927 | 81221609 | *WDR97* |
|  |  |  | 81291158 | 81310006 | *BOP1* |
|  |  |  | 81295505 | 81299508 | *SCX* |
|  |  |  | 81310095 | 81328764 | *HSF1* |
|  |  |  | 81329989 | 81338811 | *DGAT1* |
|  |  |  | 81239632 | 81291078 | *MROH1* |
| 14 | 81267067 | snp23119-scaffold2304-159526 | 81166501 | 81168897 | *SMPD5* |
|  |  |  | 81186394 | 81188312 | *EXOSC4* |
|  |  |  | 81189485 | 81192839 | *GPAA1* |
|  |  |  | 81205652 | 81209620 | *SHARPIN* |
|  |  |  | 81209801 | 81212830 | *MAF1* |
|  |  |  | 81226123 | 81229696 | *HGH1* |
|  |  |  | 81250250 | 81250321 | *MIR1839* |
|  |  |  | 81356433 | 81358207 | *TMEM249* |
|  |  |  | 81359261 | 81362291 | *FBXL6* |
|  |  |  | 81168936 | 81186366 | *OPLAH* |
|  |  |  | 81212927 | 81221609 | *WDR97* |
|  |  |  | 81291158 | 81310006 | *BOP1* |
|  |  |  | 81295505 | 81299508 | *SCX* |
|  |  |  | 81310095 | 81328764 | *HSF1* |
|  |  |  | 81329989 | 81338811 | *DGAT1* |
|  |  |  | 81342592 | 81348797 | *SCRT1* |
|  |  |  | 81362356 | 81365018 | *SLC52A2* |
|  |  |  | 81239632 | 81291078 | *MROH1* |
| 14 | 81291766 | snp23118-scaffold2304-126585 | 81189485 | 81192839 | *GPAA1* |
|  |  |  | 81205652 | 81209620 | *SHARPIN* |
|  |  |  | 81209801 | 81212830 | *MAF1* |
|  |  |  | 81226123 | 81229696 | *HGH1* |
|  |  |  | 81250250 | 81250321 | *MIR1839* |
|  |  |  | 81356433 | 81358207 | *TMEM249* |
|  |  |  | 81359261 | 81362291 | *FBXL6* |
|  |  |  | 81212927 | 81221609 | *WDR97* |
|  |  |  | 81291158 | 81310006 | *BOP1* |
|  |  |  | 81295505 | 81299508 | *SCX* |
|  |  |  | 81310095 | 81328764 | *HSF1* |
|  |  |  | 81329989 | 81338811 | *DGAT1* |
|  |  |  | 81342592 | 81348797 | *SCRT1* |
|  |  |  | 81362356 | 81365018 | *SLC52A2* |
|  |  |  | 81372718 | 81386296 | *ADCK5* |
|  |  |  | 81239632 | 81291078 | *MROH1* |
|  |  |  | 81386288 | 81400707 | *CPSF1* |
| 14 | 91122307 | snp19629-scaffold1983-289157 | 91135660 | 91288451 | *FAM135A* |
| 15 | 75951851 | snp88-scaffold100-417512 | 75816546 | 75953978 | *YAP1* |
| 16 | 78915310 | snp41855-scaffold5454-26183 | 78910361 | 78917460 | *ARL8A* |
|  |  |  | 78797724 | 78862417 | *LGR6* |
|  |  |  | 78920266 | 78924249 | *GPR37L1* |
|  |  |  | 78900064 | 78909263 | *PTPN7* |
|  |  |  | 78954089 | 79036455 | *NAV1* |
| 17 | 8947492 | snp12120-scaffold1448-789150 | 8980630 | 8988187 | *ERP29* |
|  |  |  | 8828754 | 8861723 | *MAPKAPK5* |
|  |  |  | 8942445 | 8972421 | *TMEM116* |
|  |  |  | 8991136 | 9056242 | *NAA25* |
| 17 | 17982610 | snp44803-scaffold609-3176060 | 18046085 | 18051197 | *B3GNT4* |
|  |  |  | 17897145 | 17926782 | *BCL7A* |
|  |  |  | 18030284 | 18045738 | *LRRC43* |
|  |  |  | 18051141 | 18069073 | *DIABLO* |
|  |  |  | 17943309 | 18002178 | *MLXIP* |
|  |  |  | 18073347 | 18104964 | *VPS33A* |
| 18 | 20337955 | snp40187-scaffold512-1292428 | 20284238 | 20338337 | *N4BP1* |
| 18 | 56302975 | snp18290-scaffold1857-340926 | 56279755 | 56281333 | *SPACA4* |
|  |  |  | 56288135 | 56291940 | *RPL18* |
|  |  |  | 56300148 | 56306649 | *DBP* |
|  |  |  | 56321271 | 56326460 | *NTN5* |
|  |  |  | 56393899 | 56396881 | *IZUMO1* |
|  |  |  | 56399123 | 56402123 | *FUT1* |
|  |  |  | 56184531 | 56206117 | *LMTK3* |
|  |  |  | 56239547 | 56275810 | *SULT2B1* |
|  |  |  | 56276130 | 56286719 | *FAM83E* |
|  |  |  | 56292056 | 56299987 | *SPHK2* |
|  |  |  | 56306976 | 56313358 | *CA11* |
|  |  |  | 56373047 | 56378786 | *MAMSTR* |
| 19 | 14819207 | snp1630-scaffold1046-1400739 | 14753732 | 14756613 | *ZNF830* |
|  |  |  | 14713967 | 14735131 | *LIG3* |
|  |  |  | 14756667 | 14791901 | *CCT6B* |
| 19 | 34412900 | snp28462-scaffold303-4402023 | 34466190 | 34468560 | *RASD1* |
|  |  |  | 34427348 | 34463596 | *PEMT* |
|  |  |  | 34296405 | 34400733 | *RAI1* |
|  |  |  | 34469592 | 34475211 | *MED9* |
| 21 | 32270925 | snp6505-scaffold1230-1135091 | 32134459 | 32211943 | *HMG20A* |
|  |  |  | 32344170 | 32558067 | *LINGO1* |
| 24 | 12176434 | snp27730-scaffold296-191224 | - | - | *-* |
| 25 | 41460436 | snp38502-scaffold4864-10739 | 41471003 | 41479284 | *AMZ1* |
|  |  |  | 41495061 | 41505102 | *BRAT1* |
|  |  |  | 41506648 | 41546404 | *IQCE* |
|  |  |  | 41387384 | 41460769 | *GNA12* |
| 26 | 134674 | snp6389-scaffold1226-48340 | 220917 | 224733 | *SPRN* |
|  |  |  | 224976 | 243234 | *MTG1* |
| 26 | 14182177 | snp18571-scaffold1878-249261 | 14142772 | 14149499 | *EMX2* |
| 27 | 19963783 | snp55752-scaffold864-3177522 | 19926832 | 19943941 | *DUSP4* |
|  |  |  | 19958922 | 20112234 | *TNKS* |
| 28 | 13109332 | snp39795-scaffold509-2468879 | 12259450 | 13028177 | *KCNMA1* |
| 29 | 50448626 | snp26537-scaffold2765-185379 | 50402134 | 50409520 | *TH* |
|  |  |  | 50411481 | 50412858 | *INS* |
|  |  |  | 50430375 | 50438275 | *IGF2* |
|  |  |  | 50436652 | 50436719 | *MIR483* |

Table S10

Title: GO term biological processes

| GO Term | P-value | Genes |
| --- | --- | --- |
| positive regulation of insulin receptor signaling pathway (GO:0046628) | 3.92425E-05 | *OSBPL8;IGF2;INS* |
| positive regulation of cellular response to insulin stimulus (GO:1900078) | 0.000130539 | *OSBPL8;IGF2;INS* |
| positive regulation of glycogen biosynthetic process (GO:0045725) | 0.000205278 | *IGF2;GCK;INS* |
| positive regulation of glycogen metabolic process (GO:0070875) | 0.000303248 | *IGF2;GCK;INS* |
| synaptic vesicle transport (GO:0048489) | 0.00106956 | *BLOC1S6;SYNJ1;TH;DNAJC5* |
| establishment of synaptic vesicle localization (GO:0097480) | 0.00106956 | *BLOC1S6;TH;SYNJ1;DNAJC5* |
| regulation of glucan biosynthetic process (GO:0010962) | 0.00152014 | *IGF2;GCK;INS* |
| regulation of glycogen biosynthetic process (GO:0005979) | 0.00152014 | *IGF2;GCK;INS* |
| positive regulation of glucose metabolic process (GO:0010907) | 0.002028028 | *IGF2;GCK;INS* |
| opioid receptor signaling pathway (GO:0038003) | 0.002152778 | *NPBWR2;OPRL1* |
| regulation of glycogen metabolic process (GO:0070873) | 0.002630193 | *IGF2;GCK;INS* |
| regulation of polysacchDrye biosynthetic process (GO:0032885) | 0.002630193 | *IGF2;GCK;INS* |
| negative regulation of gluconeogenesis (GO:0045721) | 0.002677039 | *GCK;INS* |
| regulation of insulin receptor signaling pathway (GO:0046626) | 0.002852812 | *OSBPL8;IGF2;INS* |
| regulation of polysacchDrye metabolic process (GO:0032881) | 0.004138392 | *IGF2;GCK;INS* |
| positive regulation of glycolytic process (GO:0045821) | 0.004568581 | *GCK;INS* |
| regulation of cellular response to insulin stimulus (GO:1900076) | 0.005383608 | *OSBPL8;IGF2;INS* |
| establishment of vesicle localization (GO:0051650) | 0.006842349 | *BLOC1S6;TH;SYNJ1;DNAJC5* |
| positive regulation of lipid biosynthetic process (GO:0046889) | 0.008054691 | *IGF2;LDLR;INS* |
| peptidyl-tyrosine dephosphorylation (GO:0035335) | 0.008755251 | *PTPRR;PTPRB;PTPN7;PTPRK* |
| positive regulation of cellular carbohydrate metabolic process (GO:0010676) | 0.009874347 | *IGF2;GCK;INS* |
| stem cell maintenance (GO:0019827) | 0.010987896 | *YAP1;TCF7L1;SMARCA4;DIS3L2* |
| regulation of keratinocyte proliferation (GO:0010837) | 0.01291544 | *YAP1;PTPRK* |
| positive regulation of carbohydrate metabolic process (GO:0045913) | 0.013591446 | *IGF2;GCK;INS* |
| vasculogenesis (GO:0001570) | 0.016026436 | *YAP1;SOX18;SMARCA4* |
| positive regulation of vasodilation (GO:0045909) | 0.01780101 | *NPPC;INS* |
| RNA phosphodiester bond hydrolysis, exonucleolytic (GO:0090503) | 0.01780101 | *EXOSC4;DIS3L2* |
| regulation of glycolytic process (GO:0006110) | 0.01780101 | *GCK;INS* |
| negative regulation of oxidative stress-induced cell death (GO:1903202) | 0.020492444 | *GPR37L1;INS* |
| regulation of generation of precursor metabolites and energy (GO:0043467) | 0.020840446 | *IGF2;GCK;INS* |
| negative regulation of cellular response to oxidative stress (GO:1900408) | 0.0218978 | *GPR37L1;INS* |
| negative regulation of response to oxidative stress (GO:1902883) | 0.0218978 | *GPR37L1;INS* |
| vesicle docking involved in exocytosis (GO:0006904) | 0.0218978 | *BLOC1S6;YKT6* |
| positive regulation of protein kinase B signaling (GO:0051897) | 0.023909118 | *OSBPL8;IGF2;INS* |
| regulation of carbohydrate biosynthetic process (GO:0043255) | 0.024712446 | *IGF2;GCK;INS* |
| nuclear-transcribed mRNA catabolic process, exonucleolytic (GO:0000291) | 0.024824283 | *EXOSC4;DIS3L2* |
| negative regulation of intracellular steroid hormone receptor signaling pathway (GO:0033144) | 0.026344033 | *KANK2;SMARCA4* |
| regulation of cellular carbohydrate catabolic process (GO:0043471) | 0.027900552 | *GCK;INS* |
| regulation of carbohydrate catabolic process (GO:0043470) | 0.027900552 | *GCK;INS* |
| ribosome biogenesis (GO:0042254) | 0.029493171 | *BOP1;MTG1* |
| vesicle docking (GO:0048278) | 0.029493171 | *BLOC1S6;YKT6* |
| negative regulation of cellular carbohydrate metabolic process (GO:0010677) | 0.032784078 | *GCK;INS* |
| regulation of gluconeogenesis (GO:0006111) | 0.032784078 | *GCK;INS* |
| RNA phosphodiester bond hydrolysis (GO:0090501) | 0.033538 | *BOP1;EXOSC4;DIS3L2* |
| negative regulation of inflammatory response (GO:0050728) | 0.033538 | *SHARPIN;INS;IL22RA2* |
| response to nicotine (GO:0035094) | 0.036211574 | *TH;IGF2* |
| cellular response to abiotic stimulus (GO:0071214) | 0.037237728 | *YAP1;IGF2;SCX;PTPRK;N4BP1* |
| regulation of vasodilation (GO:0042312) | 0.037974957 | *NPPC;INS* |
| regulation of oxidative stress-induced cell death (GO:1903201) | 0.037974957 | *GPR37L1;INS* |
| endocrine pancreas development (GO:0031018) | 0.037974957 | *GCK;INS* |
| regulation of glucose metabolic process (GO:0010906) | 0.038486001 | *IGF2;GCK;INS* |
| protein dephosphorylation (GO:0006470) | 0.039017093 | *PTPRR;PTPRB;PTPN7;PTPRK* |
| chondrocyte differentiation (GO:0002062) | 0.041597895 | *NPPC;SCX* |
| positive regulation of mitosis (GO:0045840) | 0.04345623 | *IGF2;INS* |
| establishment of organelle localization (GO:0051656) | 0.044446422 | *BLOC1S6;SYNJ1;TH;DNAJC5* |
| negative regulation of carbohydrate metabolic process (GO:0045912) | 0.045345012 | *GCK;INS* |
| regulation of lipid catabolic process (GO:0050994) | 0.047263648 | *LDLR;INS* |
| somatic stem cell maintenance (GO:0035019) | 0.047263648 | *YAP1;TCF7L1* |
| positive regulation of lipid metabolic process (GO:0045834) | 0.048271802 | *IGF2;LDLR;INS* |
| regulation of cellular response to oxidative stress (GO:1900407) | 0.049211557 | *GPR37L1;INS* |
| membrane docking (GO:0022406) | 0.049211557 | *BLOC1S6;YKT6* |

Table S11

Title: MAF (Minor Allele Frequency) and major allele of the 13 SNPs shared by Dry and Temperate/Continental groups.

|  |  |  | **Dry** | | **Temparate** | | **Continental** | |
| --- | --- | --- | --- | --- | --- | --- | --- | --- |
| **chr** | **SNP** | **pos** | **Major Allele** | **MAF** | **Major Allele** | **MAF** | **Major Allele** | **MAF** |
| 1 | snp46704-scaffold65-2970760 | 107099805 | A | 0.02638 | G | 0.4731 | G | 0.2333 |
| 8 | snp28156-scaffold300-6834721 | 42685359 | A | 0.04146 | C | 0.4652 | C | 0.3708 |
| 9 | snp59063-scaffold969-1582519 | 31573311 | C | 0.1457 | A | 0.307 | A | 0.0875 |
| 11 | snp17869-scaffold185-2798635 | 71084889 | G | 0.1149 | A | 0.4122 | A | 0.0875 |
| 12 | snp13627-scaffold1526-1179360 | 9769049 | A | 0.02387 | C | 0.4984 | C | 0.1708 |
| 14 | snp11114-scaffold14-1879194 | 22918759 | A | 0.09987 | G | 0.3647 | G | 0.1875 |
| **14** | snp37028-scaffold449-542638 | 59903218 | C | 0.1112 | A | 0.307 | A | 0.1125 |
| 14 | snp37024-scaffold449-380304 | 60065266 | A | 0.06784 | G | 0.356 | G | 0.3083 |
| 15 | snp54732-scaffold837-2384884 | 25035757 | G | 0.0804 | A | 0.3821 | A | 0.2625 |
| 15 | snp26554-scaffold277-769315 | 60451258 | G | 0.1237 | A | 0.3204 | A | 0.07917 |
| 16 | snp11346-scaffold141-1075613 | 42716941 | G | 0.08668 | A | 0.3663 | A | 0.2958 |
| 18 | snp18308-scaffold1857-1119035 | 57070577 | A | 0.1124 | C | 0.3046 | C | 0.0625 |
| 22 | snp15368-scaffold163-3716418 | 37449646 | A | 0.1338 | C | 0.2745 | C | 0.1583 |

Table S12

Title: Samβada significant results for the SNPs of the *F*_ST_ analyses. For each SNP probe, chromosome and position are reported, as well as Environment, Gscore, WaldScore, AIC, Abs_Beta1 and the Köppen group in which the SNP has been detected by the Fst analyses.

Description: For each SNP probe: chromosome and position are reported, as well as Environment, G score, Wald Score, AIC, Abs_Beta1 and the Köppen group in which the SNP was detected by the *F*_ST_ analyses.

|  |  |  |  |  | > 0.999 quantile | > 0.999 quantile |  | > 0.999 quantile |  |
| --- | --- | --- | --- | --- | --- | --- | --- | --- | --- |
|  |  |  |  |  | > 0.99 quantile | > 0.99 quantile | < 0.01 quantile | > 0.99 quantile |  |
|  |  |  |  |  | > 0.95 quantile | > 0.95 quantile | < 0.05 quantile | > 0.95 quantile |  |
|  |  |  |  |  | > 0.9 quantile | > 0.9 quantile | < 0.1 quantile | > 0.9 quantile |  |
|  |  |  |  |  |  |  |  |  |  |
| **Probe name** | **genotype** | **chr** | **pos** | **Environment** | **Gscore** | **WaldScore** | **AIC** | **abs(Beta_1)** | **Koeppen group** |
| snp10004-scaffold1356-853276 | GG | 7 | 56859625 | bio2 | 719.28 | 552.27 | 2968.16 | 1.19 | Temperate |
| snp10004-scaffold1356-853276 | AA | 7 | 56859625 | bio1 | 492.97 | 392.18 | 2401.50 | 1.11 | Temperate |
| snp10004-scaffold1356-853276 | AG | 7 | 56859625 | bio7 | 89.29 | 83.86 | 3080.20 | 0.42 | Temperate |
| snp10880-scaffold139-195858 | GG | 15 | 16017630 | bio1 | 628.24 | 480.43 | 2581.89 | 1.20 | Temperate |
| snp10880-scaffold139-195858 | AA | 15 | 16017630 | bio10 | 408.76 | 350.08 | 3054.59 | 0.88 | Temperate |
| snp10880-scaffold139-195858 | AG | 15 | 16017630 | bio4 | 62.23 | 61.29 | 3319.15 | 0.33 | Temperate |
| snp11114-scaffold14-1879194 | AA | 14 | 22918759 | bio1 | 978.95 | 649.42 | 2613.42 | 1.51 | Dry, Temperate |
| snp11114-scaffold14-1879194 | GG | 14 | 22918759 | bio1 | 905.65 | 534.46 | 1809.64 | 1.83 | Dry, Temperate |
| snp11114-scaffold14-1879194 | AG | 14 | 22918759 | bio10 | 68.62 | 64.67 | 2575.19 | 0.42 | Dry, Temperate |
| snp11346-scaffold141-1075613 | GG | 16 | 42716941 | bio1 | 762.75 | 562.94 | 2868.59 | 1.25 | Dry, Temperate |
| snp11346-scaffold141-1075613 | AA | 16 | 42716941 | bio1 | 528.85 | 373.97 | 1798.19 | 1.40 | Dry, Temperate |
| snp11346-scaffold141-1075613 | AG | 16 | 42716941 | bio5 | 158.07 | 143.51 | 2937.22 | 0.58 | Dry, Temperate |
| snp12852-scaffold1497-1502133 | AA | 22 | 51641367 | bio1 | 690.20 | 466.71 | 1948.48 | 1.51 | Continental |
| snp12852-scaffold1497-1502133 | GG | 22 | 51641367 | bio1 | 558.06 | 447.37 | 3108.24 | 1.03 | Continental |
| snp12852-scaffold1497-1502133 | AG | 22 | 51641367 | bio7 | 65.67 | 62.68 | 3147.98 | 0.36 | Continental |
| snp13627-scaffold1526-1179360 | AA | 12 | 9769049 | bio1 | 920.02 | 609.06 | 2369.46 | 1.54 | Dry Continental |
| snp13627-scaffold1526-1179360 | CC | 12 | 9769049 | bio1 | 529.12 | 349.90 | 1561.13 | 1.55 | Dry Continental |
| snp13627-scaffold1526-1179360 | AC | 12 | 9769049 | bio2 | 280.27 | 235.66 | 2191.91 | 0.92 | Dry Continental |
| snp14283-scaffold157-1737427 | AA | 5 | 18389665 | bio3 | 497.88 | 410.55 | 2459.25 | 1.07 | Tropical |
| snp14283-scaffold157-1737427 | GG | 5 | 18389665 | bio4 | 424.58 | 356.97 | 3206.68 | 0.89 | Tropical |
| snp14283-scaffold157-1737427 | AG | 5 | 18389665 | bio8 | 50.50 | 49.53 | 3328.22 | 0.30 | Tropical |
| snp14385-scaffold1570-3335289 | AA | 20 | 55624307 | bio1 | 861.97 | 599.96 | 2587.68 | 1.41 | Temperate |
| snp14385-scaffold1570-3335289 | GG | 20 | 55624307 | bio1 | 594.20 | 395.66 | 1710.00 | 1.54 | Temperate |
| snp14385-scaffold1570-3335289 | AG | 20 | 55624307 | bio8 | 150.75 | 138.78 | 2476.11 | 0.62 | Temperate |
| snp15062-scaffold161-323182 | GG | 26 | 35849838 | bio4 | 750.92 | 547.71 | 2901.36 | 1.26 | Tropical |
| snp15062-scaffold161-323182 | AA | 26 | 35849838 | bio4 | 494.60 | 403.76 | 2491.63 | 1.09 | Tropical |
| snp15062-scaffold161-323182 | AG | 26 | 35849838 | bio7 | 84.48 | 79.91 | 3216.22 | 0.40 | Tropical |
| snp15166-scaffold1620-391821 | GG | 7 | 67690250 | bio4 | 621.98 | 464.34 | 2936.70 | 1.16 | Tropical |
| snp15166-scaffold1620-391821 | AA | 7 | 67690250 | bio6 | 432.53 | 349.70 | 2595.60 | 1.02 | Tropical |
| snp15166-scaffold1620-391821 | AG | 7 | 67690250 | bio7 | 46.77 | 45.42 | 3419.85 | 0.28 | Tropical |
| snp15366-scaffold163-3580360 | AA | 22 | 37586015 | bio11 | 1087.38 | 661.53 | 2379.24 | 1.71 | Temperate |
| snp15366-scaffold163-3580360 | GG | 22 | 37586015 | bio1 | 478.17 | 334.28 | 1634.73 | 1.43 | Temperate |
| snp15366-scaffold163-3580360 | AG | 22 | 37586015 | bio11 | 340.49 | 291.46 | 2468.69 | 0.92 | Temperate |
| snp15367-scaffold163-3645059 | GG | 22 | 37521057 | bio11 | 1196.90 | 684.50 | 2267.34 | 1.86 | Temperate |
| snp15367-scaffold163-3645059 | AA | 22 | 37521057 | bio1 | 480.96 | 334.64 | 1624.51 | 1.44 | Temperate |
| snp15367-scaffold163-3645059 | AG | 22 | 37521057 | bio11 | 396.30 | 329.43 | 2412.88 | 1.01 | Temperate |
| snp15368-scaffold163-3716418 | AA | 22 | 37449646 | bio1 | 1314.82 | 726.21 | 2355.18 | 1.90 | Dry, Temperate |
| snp15368-scaffold163-3716418 | CC | 22 | 37449646 | bio1 | 947.39 | 565.93 | 1911.83 | 1.79 | Dry, Temperate |
| snp15368-scaffold163-3716418 | AC | 22 | 37449646 | bio8 | 111.76 | 105.94 | 2773.98 | 0.50 | Dry, Temperate |
| snp16015-scaffold1686-235087 | GG | 16 | 77905914 | bio4 | 932.51 | 675.70 | 2611.87 | 1.42 | Tropical |
| snp16015-scaffold1686-235087 | AA | 16 | 77905914 | bio4 | 927.85 | 534.43 | 2487.58 | 1.66 | Tropical |
| snp16015-scaffold1686-235087 | AG | 16 | 77905914 | bio8 | 105.49 | 100.69 | 2983.42 | 0.46 | Tropical |
| snp1639-scaffold10469-54125 | GG | 13 | 53305564 | bio1 | 787.06 | 571.29 | 2711.12 | 1.31 | Continental |
| snp1639-scaffold10469-54125 | AA | 13 | 53305564 | bio1 | 612.71 | 390.48 | 1598.10 | 1.64 | Continental |
| snp1639-scaffold10469-54125 | AG | 13 | 53305564 | bio8 | 130.95 | 122.74 | 2695.21 | 0.55 | Continental |
| snp16418-scaffold173-930565 | AA | 16 | 3554107 | bio4 | 767.47 | 586.83 | 2628.49 | 1.29 | Tropical |
| snp16418-scaffold173-930565 | GG | 16 | 3554107 | bio4 | 676.76 | 472.73 | 2784.52 | 1.28 | Tropical |
| snp16418-scaffold173-930565 | AG | 16 | 3554107 | bio8 | 48.14 | 47.24 | 3245.43 | 0.29 | Tropical |
| snp17391-scaffold1802-55674 | CC | 5 | 25820184 | bio3 | 856.84 | 602.10 | 2604.76 | 1.38 | Tropical |
| snp17391-scaffold1802-55674 | AA | 5 | 25820184 | bio4 | 363.66 | 297.42 | 2960.47 | 0.89 | Tropical |
| snp17391-scaffold1802-55674 | AC | 5 | 25820184 | bio3 | 140.91 | 127.47 | 3190.73 | 0.53 | Tropical |
| snp17392-scaffold1802-98740 | AA | 5 | 25862995 | bio4 | 795.64 | 608.69 | 2802.58 | 1.27 | Tropical |
| snp17392-scaffold1802-98740 | GG | 5 | 25862995 | bio4 | 423.39 | 321.69 | 2734.84 | 1.03 | Tropical |
| snp17392-scaffold1802-98740 | AG | 5 | 25862995 | bio3 | 107.68 | 99.23 | 3199.35 | 0.46 | Tropical |
| snp17760-scaffold1842-340129 | GG | 11 | 3381675 | bio1 | 723.10 | 500.56 | 2145.49 | 1.45 | Continental |
| snp17760-scaffold1842-340129 | AA | 11 | 3381675 | bio1 | 574.14 | 455.76 | 3114.22 | 1.05 | Continental |
| snp17760-scaffold1842-340129 | AG | 11 | 3381675 | bio4 | 40.82 | 40.49 | 3156.91 | 0.27 | Continental |
| snp17863-scaffold185-2478573 | GG | 11 | 71403908 | bio5 | 747.46 | 477.07 | 2508.03 | 1.48 | Dry |
| snp17863-scaffold185-2478573 | AA | 11 | 71403908 | bio5 | 729.38 | 544.70 | 2868.38 | 1.23 | Dry |
| snp17863-scaffold185-2478573 | AG | 11 | 71403908 | bio4 | 66.82 | 65.74 | 3155.89 | 0.35 | Dry |
| snp17869-scaffold185-2798635 | GG | 11 | 71084889 | bio5 | 608.43 | 474.32 | 3071.87 | 1.09 | Dry, Continental |
| snp17869-scaffold185-2798635 | AA | 11 | 71084889 | bio5 | 534.39 | 371.13 | 2380.76 | 1.29 | Dry, Continental |
| snp17869-scaffold185-2798635 | AG | 11 | 71084889 | bio7 | 84.91 | 80.07 | 3125.81 | 0.41 | Dry, Continental |
| snp18308-scaffold1857-1119035 | CC | 18 | 57070577 | bio1 | 729.90 | 525.01 | 2405.39 | 1.35 | Dry, Temperate, Continental |
| snp18308-scaffold1857-1119035 | AA | 18 | 57070577 | bio1 | 704.73 | 530.46 | 2976.79 | 1.19 | Dry, Temperate, Continental |
| snp18308-scaffold1857-1119035 | CC | 18 | 57070577 | bio5 | 660.42 | 436.50 | 2474.86 | 1.40 | Dry, Temperate, Continental |
| snp19498-scaffold197-373855 | AA | 1 | 15839421 | bio2 | 576.51 | 465.74 | 3079.75 | 1.05 | Dry |
| snp19498-scaffold197-373855 | GG | 1 | 15839421 | bio1 | 525.03 | 415.22 | 2474.37 | 1.13 | Dry |
| snp19498-scaffold197-373855 | AG | 1 | 15839421 | bio7 | 79.29 | 74.89 | 3067.17 | 0.40 | Dry |
| snp19609-scaffold1980-395706 | AA | 5 | 47020710 | bio1 | 813.48 | 563.63 | 2384.24 | 1.44 | Continental |
| snp19609-scaffold1980-395706 | GG | 5 | 47020710 | bio11 | 663.11 | 378.33 | 1331.64 | 1.95 | Continental |
| snp19609-scaffold1980-395706 | AG | 5 | 47020710 | bio1 | 141.77 | 132.94 | 2238.72 | 0.63 | Continental |
| snp19670-scaffold1986-1002659 | AA | 16 | 2369067 | bio2 | 669.30 | 464.44 | 2253.93 | 1.39 | Dry |
| snp19670-scaffold1986-1002659 | CC | 16 | 2369067 | bio2 | 576.63 | 466.56 | 3102.73 | 1.04 | Dry |
| snp19670-scaffold1986-1002659 | AC | 16 | 2369067 | bio4 | 92.25 | 90.00 | 3142.69 | 0.41 | Dry |
| snp22492-scaffold2225-288080 | AA | 5 | 118885547 | bio4 | 656.00 | 527.85 | 2937.65 | 1.12 | Tropical |
| snp22492-scaffold2225-288080 | GG | 5 | 118885547 | bio4 | 462.46 | 334.10 | 2588.40 | 1.13 | Tropical |
| snp22492-scaffold2225-288080 | AG | 5 | 118885547 | bio11 | 96.80 | 93.67 | 3217.40 | 0.41 | Tropical |
| snp24965-scaffold2564-131990 | CC | 3 | 1091508 | bio1 | 1177.55 | 618.80 | 1793.66 | 2.09 | Continental |
| snp24965-scaffold2564-131990 | AA | 3 | 1091508 | bio1 | 641.05 | 312.94 | 1122.95 | 2.23 | Continental |
| snp24965-scaffold2564-131990 | AC | 3 | 1091508 | bio1 | 360.85 | 284.32 | 1828.55 | 1.15 | Continental |
| snp26554-scaffold277-769315 | AA | 15 | 60451258 | bio1 | 1079.80 | 589.24 | 1788.79 | 2.01 | Dry, Temperate, Continental |
| snp26554-scaffold277-769315 | GG | 15 | 60451258 | bio1 | 991.09 | 652.84 | 2697.13 | 1.51 | Dry, Temperate, Continental |
| snp26554-scaffold277-769315 | AG | 15 | 60451258 | bio8 | 41.73 | 41.02 | 2971.34 | 0.29 | Dry, Temperate, Continental |
| snp27211-scaffold29-3020379 | GG | 1 | 120984378 | bio4 | 544.86 | 432.06 | 3066.64 | 1.04 | Tropical |
| snp27211-scaffold29-3020379 | AA | 1 | 120984378 | bio4 | 469.07 | 384.33 | 2450.36 | 1.07 | Tropical |
| snp27211-scaffold29-3020379 | AG | 1 | 120984378 | bio8 | 54.13 | 53.02 | 3360.38 | 0.30 | Tropical |
| snp27872-scaffold299-2687550 | AA | 16 | 76091919 | bio5 | 628.23 | 487.44 | 3035.46 | 1.11 | Temperate |
| snp27872-scaffold299-2687550 | GG | 16 | 76091919 | bio2 | 446.61 | 349.33 | 2330.94 | 1.11 | Temperate |
| snp27872-scaffold299-2687550 | AG | 16 | 76091919 | bio15 | 115.08 | 108.00 | 3269.85 | 0.46 | Temperate |
| snp2807-scaffold1082-243110 | AA | 1 | 693125 | bio8 | 477.75 | 392.37 | 3213.74 | 0.94 | Dry |
| snp2807-scaffold1082-243110 | GG | 1 | 693125 | bio2 | 438.42 | 341.14 | 2276.87 | 1.12 | Dry |
| snp2807-scaffold1082-243110 | AG | 1 | 693125 | bio7 | 92.20 | 86.62 | 3141.00 | 0.42 | Dry |
| snp28156-scaffold300-6834721 | AA | 8 | 42685359 | bio2 | 817.89 | 573.88 | 2532.99 | 1.40 | Dry, Temperate |
| snp28156-scaffold300-6834721 | CC | 8 | 42685359 | bio2 | 452.04 | 282.75 | 1492.93 | 1.57 | Dry, Temperate |
| snp28156-scaffold300-6834721 | AC | 8 | 42685359 | bio2 | 244.06 | 214.93 | 2459.00 | 0.79 | Dry, Temperate |
| snp29430-scaffold318-2430727 | GG | 9 | 51803364 | bio1 | 697.39 | 396.93 | 1444.98 | 1.89 | Continental |
| snp29430-scaffold318-2430727 | AA | 9 | 51803364 | bio1 | 645.41 | 498.70 | 2785.35 | 1.16 | Continental |
| snp29430-scaffold318-2430727 | AG | 9 | 51803364 | bio2 | 85.34 | 82.29 | 2627.26 | 0.45 | Continental |
| snp30827-scaffold340-1374445 | GG | 6 | 14012747 | bio4 | 319.22 | 289.36 | 3214.85 | 0.75 | Tropical |
| snp30827-scaffold340-1374445 | AA | 6 | 14012747 | bio4 | 262.64 | 221.82 | 2867.50 | 0.78 | Tropical |
| snp32940-scaffold3820-24472 | GG | 24 | 12459187 | bio5 | 693.39 | 520.42 | 2927.99 | 1.19 | Dry |
| snp32940-scaffold3820-24472 | AA | 24 | 12459187 | bio5 | 598.72 | 393.68 | 2266.52 | 1.42 | Dry |
| snp32940-scaffold3820-24472 | AG | 24 | 12459187 | bio12 | 49.71 | 48.97 | 3181.68 | 0.30 | Dry |
| snp3395-scaffold1102-2100714 | AA | 28 | 3509281 | bio5 | 672.70 | 504.45 | 2996.40 | 1.17 | Dry |
| snp3395-scaffold1102-2100714 | GG | 28 | 3509281 | bio5 | 273.73 | 222.65 | 2401.71 | 0.90 | Dry |
| snp3395-scaffold1102-2100714 | AG | 28 | 3509281 | bio10 | 179.79 | 162.00 | 3060.87 | 0.60 | Dry |
| snp37022-scaffold449-312721 | CC | 14 | 60132731 | bio6 | 879.03 | 575.42 | 2740.08 | 1.46 | Tropical |
| snp37022-scaffold449-312721 | AA | 14 | 60132731 | bio11 | 593.60 | 458.33 | 2598.47 | 1.17 | Tropical |
| snp37022-scaffold449-312721 | AC | 14 | 60132731 | bio4 | 90.88 | 85.90 | 3124.47 | 0.42 | Tropical |
| snp37023-scaffold449-347941 | AA | 14 | 60097955 | bio3 | 585.36 | 341.98 | 2142.85 | 1.66 | Temperate |
| snp37023-scaffold449-347941 | GG | 14 | 60097955 | bio11 | 525.97 | 419.95 | 3161.72 | 1.00 | Temperate |
| snp37024-scaffold449-380304 | AA | 14 | 60065266 | bio1 | 1010.95 | 648.07 | 2412.04 | 1.60 | Dry, Temperate |
| snp37024-scaffold449-380304 | GG | 14 | 60065266 | bio3 | 755.62 | 351.23 | 1650.72 | 2.54 | Dry, Temperate |
| snp37024-scaffold449-380304 | AG | 14 | 60065266 | bio1 | 162.72 | 151.27 | 2309.86 | 0.66 | Dry, Temperate |
| snp37028-scaffold449-542638 | CC | 14 | 59903218 | bio11 | 939.29 | 618.66 | 2653.87 | 1.49 | Dry, Temperate |
| snp37028-scaffold449-542638 | AA | 14 | 59903218 | bio1 | 835.45 | 515.89 | 1860.91 | 1.72 | Dry, Temperate |
| snp37028-scaffold449-542638 | AC | 14 | 59903218 | bio3 | 62.55 | 57.69 | 2603.59 | 0.41 | Dry, Temperate |
| snp37066-scaffold45-427991 | GG | 18 | 1621854 | bio14 | 455.42 | 395.35 | 2065.67 | 1.02 | Continental |
| snp37066-scaffold45-427991 | AA | 18 | 1621854 | bio1 | 272.73 | 152.13 | 763.59 | 1.89 | Continental |
| snp37066-scaffold45-427991 | AG | 18 | 1621854 | bio1 | 171.29 | 154.87 | 1915.21 | 0.77 | Continental |
| snp38375-scaffold486-16164 | AA | 5 | 25748303 | bio4 | 1070.40 | 699.77 | 2216.87 | 1.68 | Tropical |
| snp38375-scaffold486-16164 | GG | 5 | 25748303 | bio4 | 792.03 | 568.87 | 2863.11 | 1.30 | Tropical |
| snp40572-scaffold519-3326486 | CC | 1 | 80898097 | bio6 | 577.22 | 439.60 | 2772.60 | 1.14 | Tropical |
| snp40572-scaffold519-3326486 | CC | 1 | 80898097 | bio4 | 532.82 | 444.78 | 2817.00 | 1.04 | Tropical |
| snp40572-scaffold519-3326486 | AA | 1 | 80898097 | bio3 | 431.03 | 322.79 | 2882.29 | 1.04 | Tropical |
| snp41061-scaffold531-913438 | AA | 12 | 66231248 | bio4 | 802.05 | 601.93 | 2881.65 | 1.27 | Tropical |
| snp41061-scaffold531-913438 | GG | 12 | 66231248 | bio4 | 421.06 | 297.33 | 2412.67 | 1.16 | Tropical |
| snp41061-scaffold531-913438 | AG | 12 | 66231248 | bio3 | 168.80 | 148.83 | 3085.97 | 0.60 | Tropical |
| snp41067-scaffold531-1161947 | GG | 12 | 66480495 | bio2 | 551.34 | 452.46 | 3138.30 | 1.01 | Dry |
| snp41067-scaffold531-1161947 | AA | 12 | 66480495 | bio2 | 529.83 | 387.26 | 2195.72 | 1.26 | Dry |
| snp41067-scaffold531-1161947 | AG | 12 | 66480495 | bio7 | 89.27 | 84.05 | 3157.01 | 0.42 | Dry |
| snp41285-scaffold537-479237 | GG | 19 | 56568731 | bio1 | 936.53 | 621.33 | 2436.34 | 1.53 | Temperate |
| snp41285-scaffold537-479237 | AA | 19 | 56568731 | bio1 | 537.32 | 359.00 | 1607.81 | 1.53 | Temperate |
| snp41285-scaffold537-479237 | AG | 19 | 56568731 | bio11 | 241.92 | 215.77 | 2352.15 | 0.80 | Temperate |
| snp42192-scaffold550-1425798 | GG | 18 | 55265271 | bio1 | 856.93 | 603.73 | 2714.11 | 1.37 | Temperate |
| snp42192-scaffold550-1425798 | AA | 18 | 55265271 | bio1 | 510.01 | 359.43 | 1739.33 | 1.41 | Temperate |
| snp42192-scaffold550-1425798 | AG | 18 | 55265271 | bio1 | 184.27 | 172.18 | 2777.94 | 0.62 | Temperate |
| snp43711-scaffold586-498658 | GG | 1 | 30689243 | bio1 | 686.39 | 492.41 | 2261.70 | 1.37 | Continental |
| snp43711-scaffold586-498658 | AA | 1 | 30689243 | bio5 | 396.52 | 222.87 | 1001.16 | 2.18 | Continental |
| snp43711-scaffold586-498658 | AG | 1 | 30689243 | bio1 | 251.47 | 220.71 | 2169.00 | 0.85 | Continental |
| snp45456-scaffold620-841281 | AA | 9 | 57974239 | bio5 | 724.04 | 506.56 | 2864.40 | 1.28 | Continental |
| snp45456-scaffold620-841281 | GG | 9 | 57974239 | bio1 | 624.49 | 417.03 | 1778.64 | 1.54 | Continental |
| snp45456-scaffold620-841281 | AG | 9 | 57974239 | bio10 | 102.29 | 95.02 | 2802.19 | 0.48 | Continental |
| snp46704-scaffold65-2970760 | AA | 1 | 107099805 | bio1 | 1123.42 | 654.36 | 2116.00 | 1.83 | Dry, Temperate |
| snp46704-scaffold65-2970760 | GG | 1 | 107099805 | bio1 | 645.37 | 398.03 | 1565.07 | 1.71 | Dry, Temperate |
| snp46704-scaffold65-2970760 | AG | 1 | 107099805 | bio2 | 266.16 | 220.32 | 2000.16 | 0.95 | Dry, Temperate |
| snp48651-scaffold691-601620 | GG | 13 | 61318983 | bio1 | 772.47 | 520.93 | 2120.16 | 1.51 | Continental |
| snp48651-scaffold691-601620 | AA | 13 | 61318983 | bio1 | 400.39 | 234.47 | 1063.09 | 1.80 | Continental |
| snp48651-scaffold691-601620 | AG | 13 | 61318983 | bio1 | 292.79 | 246.84 | 2004.62 | 0.97 | Continental |
| snp49737-scaffold710-867888 | AA | 6 | 20405718 | bio3 | 448.30 | 381.27 | 2764.58 | 0.95 | Tropical |
| snp49737-scaffold710-867888 | GG | 6 | 20405718 | bio3 | 319.60 | 261.69 | 3102.62 | 0.82 | Tropical |
| snp50169-scaffold717-4207960 | AA | 12 | 60950668 | bio5 | 605.23 | 387.33 | 2147.26 | 1.51 | Continental |
| snp50169-scaffold717-4207960 | GG | 12 | 60950668 | bio5 | 446.81 | 366.10 | 3195.86 | 0.92 | Continental |
| snp50169-scaffold717-4207960 | AG | 12 | 60950668 | bio16 | 57.99 | 58.63 | 2751.18 | 0.34 | Continental |
| snp50170-scaffold717-4247907 | CC | 12 | 60910543 | bio5 | 563.22 | 373.72 | 2212.72 | 1.41 | Continental |
| snp50170-scaffold717-4247907 | AA | 12 | 60910543 | bio14 | 371.67 | 304.29 | 3289.06 | 0.82 | Continental |
| snp50170-scaffold717-4247907 | AC | 12 | 60910543 | bio16 | 31.91 | 32.57 | 2846.35 | 0.25 | Continental |
| snp50216-scaffold717-6126750 | GG | 12 | 59028158 | bio12 | 498.50 | 377.47 | 3152.14 | 1.01 | Continental |
| snp50216-scaffold717-6126750 | AA | 12 | 59028158 | bio17 | 416.48 | 366.49 | 2036.65 | 1.00 | Continental |
| snp50216-scaffold717-6126750 | AG | 12 | 59028158 | bio7 | 176.55 | 156.49 | 2912.70 | 0.63 | Continental |
| snp50262-scaffold718-122726 | AA | 7 | 12489513 | bio1 | 970.59 | 616.87 | 2243.44 | 1.63 | Continental |
| snp50262-scaffold718-122726 | GG | 7 | 12489513 | bio1 | 571.24 | 355.41 | 1464.98 | 1.69 | Continental |
| snp50262-scaffold718-122726 | AG | 7 | 12489513 | bio8 | 277.53 | 231.84 | 2120.20 | 0.94 | Continental |
| snp50263-scaffold718-182724 | AA | 7 | 12549550 | bio1 | 971.84 | 622.08 | 2290.39 | 1.62 | Continental |
| snp50263-scaffold718-182724 | GG | 7 | 12549550 | bio1 | 583.75 | 361.65 | 1480.38 | 1.70 | Continental |
| snp50263-scaffold718-182724 | AG | 7 | 12549550 | bio8 | 268.27 | 226.40 | 2173.06 | 0.91 | Continental |
| snp52390-scaffold780-436708 | AA | 7 | 94571063 | bio3 | 713.84 | 532.22 | 2372.44 | 1.30 | Tropical |
| snp52390-scaffold780-436708 | CC | 7 | 94571063 | bio3 | 698.28 | 487.41 | 2929.24 | 1.26 | Tropical |
| snp52390-scaffold780-436708 | AC | 7 | 94571063 | bio14 | 39.30 | 36.46 | 3203.97 | 0.28 | Tropical |
| snp53345-scaffold804-2344420 | AA | 11 | 98388887 | bio2 | 685.19 | 519.98 | 2734.35 | 1.22 | Continental |
| snp53345-scaffold804-2344420 | GG | 11 | 98388887 | bio1 | 513.40 | 341.38 | 1546.66 | 1.54 | Continental |
| snp53345-scaffold804-2344420 | AG | 11 | 98388887 | bio2 | 207.32 | 187.27 | 2542.07 | 0.71 | Continental |
| snp54732-scaffold837-2384884 | GG | 15 | 25035757 | bio1 | 785.11 | 572.96 | 2800.70 | 1.29 | Dry, Temperate |
| snp54732-scaffold837-2384884 | AA | 15 | 25035757 | bio1 | 567.86 | 395.31 | 1822.01 | 1.44 | Dry, Temperate |
| snp54732-scaffold837-2384884 | AG | 15 | 25035757 | bio1 | 116.15 | 111.79 | 2792.50 | 0.50 | Dry, Temperate |
| snp57917-scaffold938-380104 | AA | 7 | 55625453 | bio4 | 908.48 | 664.83 | 2705.88 | 1.38 | Tropical |
| snp57917-scaffold938-380104 | GG | 7 | 55625453 | bio4 | 849.30 | 512.82 | 2552.39 | 1.56 | Tropical |
| snp57917-scaffold938-380104 | AG | 7 | 55625453 | bio15 | 132.05 | 119.77 | 2854.75 | 0.55 | Tropical |
| snp57919-scaffold938-455107 | AA | 7 | 55549326 | bio4 | 818.89 | 482.48 | 2484.14 | 1.59 | Tropical |
| snp57919-scaffold938-455107 | GG | 7 | 55549326 | bio4 | 790.23 | 606.03 | 2799.00 | 1.26 | Tropical |
| snp57919-scaffold938-455107 | AG | 7 | 55549326 | bio15 | 147.24 | 134.16 | 3043.21 | 0.55 | Tropical |
| snp59063-scaffold969-1582519 | AA | 9 | 31573311 | bio1 | 870.99 | 568.05 | 2161.41 | 1.59 | Dry, Temperate |
| snp59063-scaffold969-1582519 | CC | 9 | 31573311 | bio1 | 661.41 | 504.95 | 3025.91 | 1.15 | Dry, Temperate |
| snp59063-scaffold969-1582519 | AC | 9 | 31573311 | bio7 | 34.69 | 33.67 | 3037.47 | 0.26 | Dry, Temperate |
| snp7213-scaffold1266-2438708 | CC | 18 | 36745379 | bio4 | 969.70 | 692.28 | 2647.38 | 1.45 | Tropical |
| snp7213-scaffold1266-2438708 | AA | 18 | 36745379 | bio4 | 714.99 | 456.87 | 2586.45 | 1.42 | Tropical |
| snp7213-scaffold1266-2438708 | AC | 18 | 36745379 | bio8 | 192.36 | 176.14 | 2928.02 | 0.63 | Tropical |
| snp8476-scaffold1307-388513 | AA | 1 | 9662226 | bio4 | 881.86 | 643.37 | 2796.39 | 1.35 | Tropical |
| snp8476-scaffold1307-388513 | GG | 1 | 9662226 | bio4 | 380.54 | 265.63 | 2249.16 | 1.17 | Tropical |
| snp8476-scaffold1307-388513 | AG | 1 | 9662226 | bio3 | 244.24 | 208.89 | 3170.86 | 0.70 | Tropical |

Table S13

Title: List of genes located nearby (± 100 kb) of the 65 SNPs selected with the *F*_ST_ approach and confirmed by Samβada.

Description: The * symbol indicates the SNPs with the highest value (>0.999) also in landscape genomic analysis.

| chr | Pos | probe | Group | Genes (± 100k) |
| --- | --- | --- | --- | --- |
| 1 | 693125 | snp2807-scaffold1082-243110 | Dry | *IFNAR1, IL10RB, IFNAR2* |
| 1 | 9662226 | snp8476-scaffold1307-388513 | Tropical | *-* |
| 1 | 15839421 | snp19498-scaffold197-373855 | Dry | *-* |
| 1 | 30689243 | snp43711-scaffold586-498658 | Continental | *-* |
| 1 | 80898097 | snp40572-scaffold519-3326486 | Tropical | *ETS* |
| 1 | 107099805 | snp46704-scaffold65-2970760 * | Dry. Temperate | *-* |
| 1 | 120984378 | snp27211-scaffold29-3020379 | Tropical | *-* |
| 3 | 1091508 | snp24965-scaffold2564-131990 * | Continental | *CAPN10, RNPEPL1, KIF1A, GPR35, DUSP28, GPC1, ANKMY1* |
| 5 | 18389665 | snp14283-scaffold157-1737427 | Tropical | *-* |
| 5 | 25748303 | snp38375-scaffold486-16164 | Tropical | *HOXC4, HOXC5, HOXC6, HOXC8, HOXC9, HOXC10, HOXC11, HOXC12, HOXC13* |
| 5 | 25820184 | snp17391-scaffold1802-55674 | Tropical |  |
| 5 | 25862995 | snp17392-scaffold1802-98740 | Tropical |  |
| 5 | 47020710 | snp19609-scaffold1980-395706 | Continental | *LLPH, TMBIM4, IRAK3* |
| 5 | 118885547 | snp22492-scaffold2225-288080 | Tropical | *CPT1B, MAPK8IP2, ARSA, SYCE3, CHKB, SHANK3, ACR* |
| 6 | 14012747 | snp30827-scaffold340-1374445 | Tropical | *-* |
| 6 | 20405718 | snp49737-scaffold710-867888 | Tropical | *TET2* |
| 7 | 12489513 | snp50262-scaffold718-122726 | Continental | *CHD1, RGMB* |
| 7 | 12549550 | snp50263-scaffold718-182724 | Continental |  |
| 7 | 55549326 | snp57919-scaffold938-455107 | Tropical | *-* |
| 7 | 55625453 | snp57917-scaffold938-380104 | Tropical | *-* |
| 7 | 56859625 | snp10004-scaffold1356-853276 | Temperate | *FGF1* |
| 7 | 67690250 | snp15166-scaffold1620-391821 | Tropical | *PGBD2* |
| 7 | 94571063 | snp52390-scaffold780-436708 | Tropical | *RDH8, C7H19orf66, EIF3G, S1PR2, COL5A3, ANGPTL6, DNMT1* |
| 8 | 42685359 | snp28156-scaffold300-6834721 | Dry, Temperate | *-* |
| 9 | 31573311 | snp59063-scaffold969-1582519 * | Dry, Temperate | *-* |
| 9 | 51803364 | snp29430-scaffold318-2430727 * | Continental | *TBX18* |
| 9 | 57974239 | snp45456-scaffold620-841281 | Continental | *TAAR5, VNN1, TAAR1, SLC18B1* |
| 11 | 3381675 | snp17760-scaffold1842-340129 | Continental | *VWA3B* |
| 11 | 71084889 | snp17869-scaffold185-2798635 | Dry, Continental | *FOSL2, PLB1, BRE* |
| 11 | 71403908 | snp17863-scaffold185-2478573 | Dry |  |
| 11 | 98388887 | snp53345-scaffold804-2344420 | Continental | *LCN2, SWI5, COQ4, SLC25A25, CIZ1, DNM1, GOLGA2, TRUB2, SLC27A4, PTGES2* |
| 12 | 9769049 | snp13627-scaffold1526-1179360 | Dry,Continental | *CLYBL* |
| 12 | 59028158 | snp50216-scaffold717-6126750 | Continental | *STARD13* |
| 12 | 60910543 | snp50170-scaffold717-4247907 | Continental | *NBEA, DCLK1,* |
| 12 | 60950668 | snp50169-scaffold717-4207960 | Continental |  |
| 12 | 66231248 | snp41061-scaffold531-913438 | Tropical | *DLEU7* |
| 12 | 66480495 | snp41067-scaffold531-1161947 | Dry | *-* |
| 13 | 53305564 | snp1639-scaffold10469-54125 | Continental | *ZNF512B, SLC2A4RG, LIME1, ARFRP1, STMN3, DNAJC5, TPD52L2, ABHD16B, ZGPAT, RTEL1, UCKL1, ZBTB46* |
| 13 | 61318983 | snp48651-scaffold691-601620 | Continental | *NOL4L* |
| 14 | 22918759 | snp11114-scaffold14-1879194 | Dry,Temperate | *ZFPM2* |
| 14 | 59903218 | snp37028-scaffold449-542638 * | Dry. Temperate | *SOX17* |
| 14 | 60065266 | snp37024-scaffold449-380304 * | Dry. Temperate | *MRPL15, LYPLA1, TCEA1, RGS20, ATP6V1H* |
| 14 | 60097955 | snp37023-scaffold449-347941 | Temperate |  |
| 14 | 60132731 | snp37022-scaffold449-312721 | Tropical |  |
| 15 | 16017630 | snp10880-scaffold139-195858 | Temperate | *RAG1, TRAF6, PRR5L* |
| 15 | 25035757 | snp54732-scaffold837-2384884 | Dry, Temperate | *BBOX1* |
| 15 | 60451258 | snp26554-scaffold277-769315 | Dry, Temperate, Continental | *LAYN, SIK2* |
| 16 | 2369067 | snp19670-scaffold1986-1002659 | Dry | *RBBP5, TMCC2, NUAK2, DSTYK* |
| 16 | 3554107 | snp16418-scaffold173-930565 | Tropical | *IKBKE, SRGAP2, RASSF5,* |
| 16 | 42716941 | snp11346-scaffold141-1075613 | Dry, Temperate | *SPSB1, H6PD* |
| 16 | 76091919 | snp27872-scaffold299-2687550 | Temperate | *ATP6V1G3, PTPRC* |
| 16 | 77905914 | snp16015-scaffold1686-235087 | Tropical | *KIF14, LOC106502996, DDX59, CAMSAP2* |
| 18 | 1621854 | snp37066-scaffold45-427991 | Continental | *-* |
| 18 | 36745379 | snp7213-scaffold1266-2438708 | Tropical | *EDC4, NRN1L, LCAT, DPEP3, DPEP2, DDX28, PSKH1, PSMB10, DUS2, NFATC3, SLC12A4* |
| 18 | 55265271 | snp42192-scaffold550-1425798 | Temperate | *BBC3, LOC102178104, CCDC9, C5AR1, SAE1, ZC3H4, INAFM1* |
| 18 | 57070577 | snp18308-scaffold1857-1119035 | Dry, Temperate, Continental | *FCGRT, PRRG2, RRAS, IRF3, PRMT1, RCN3, NOSIP, SCAF1, BCL2L12, ADM5, CPT1C, TSKS, PRR12, AP2A1* |
| 19 | 56568731 | snp41285-scaffold537-479237 | Temperate | *-* |
| 20 | 55624307 | snp14385-scaffold1570-3335289 | Temperate | *-* |
| 22 | 37449646 | snp15368-scaffold163-3716418 * | Dry, Temperate | *PSMD6, ATXN7* |
| 22 | 37521057 | snp15367-scaffold163-3645059 * | Temperate | *PSMD6, THOC7, ATXN7* |
| 22 | 37586015 | snp15366-scaffold163-3580360 | Temperate |  |
| 22 | 51641367 | snp12852-scaffold1497-1502133 | Continental | *SPINK8, NME6, MAP34-B, LOC102169231, LOC102169712, LOC102181444, MAP28, CDC25A* |
| 24 | 12459187 | snp32940-scaffold3820-24472 | Dry | *-* |
| 26 | 35849838 | snp15062-scaffold161-323182 | Tropical | *PLCE1* |
| 28 | 3509281 | snp3395-scaffold1102-2100714 | Dry | *GDF10, GDF2, RBP3, ZNF488* |
