# Supplementary material for: Signatures of selection and environmental adaptation across the goat genome post-domestication
Source: Genet Sel Evol. 2018 Nov 19;50:57. doi: 10.1186/s12711-018-0421-y (PMC6240954; doi:10.1186/s12711-018-0421-y)
Supplement: Supplementary file 4 — Additional file 4. Graphical representation of ROH and iHS results at the breed level of the signals FLK and hapFLK in the sub continental groups. Left panel: FLK (points) and hapFLK (line) signatures, with as header the chromosomal region investigated. Middle panels: ROH signatures. Right panels: iHS signatures [see Additional file 2] [file 12711_2018_421_MOESM4_ESM.pdf]

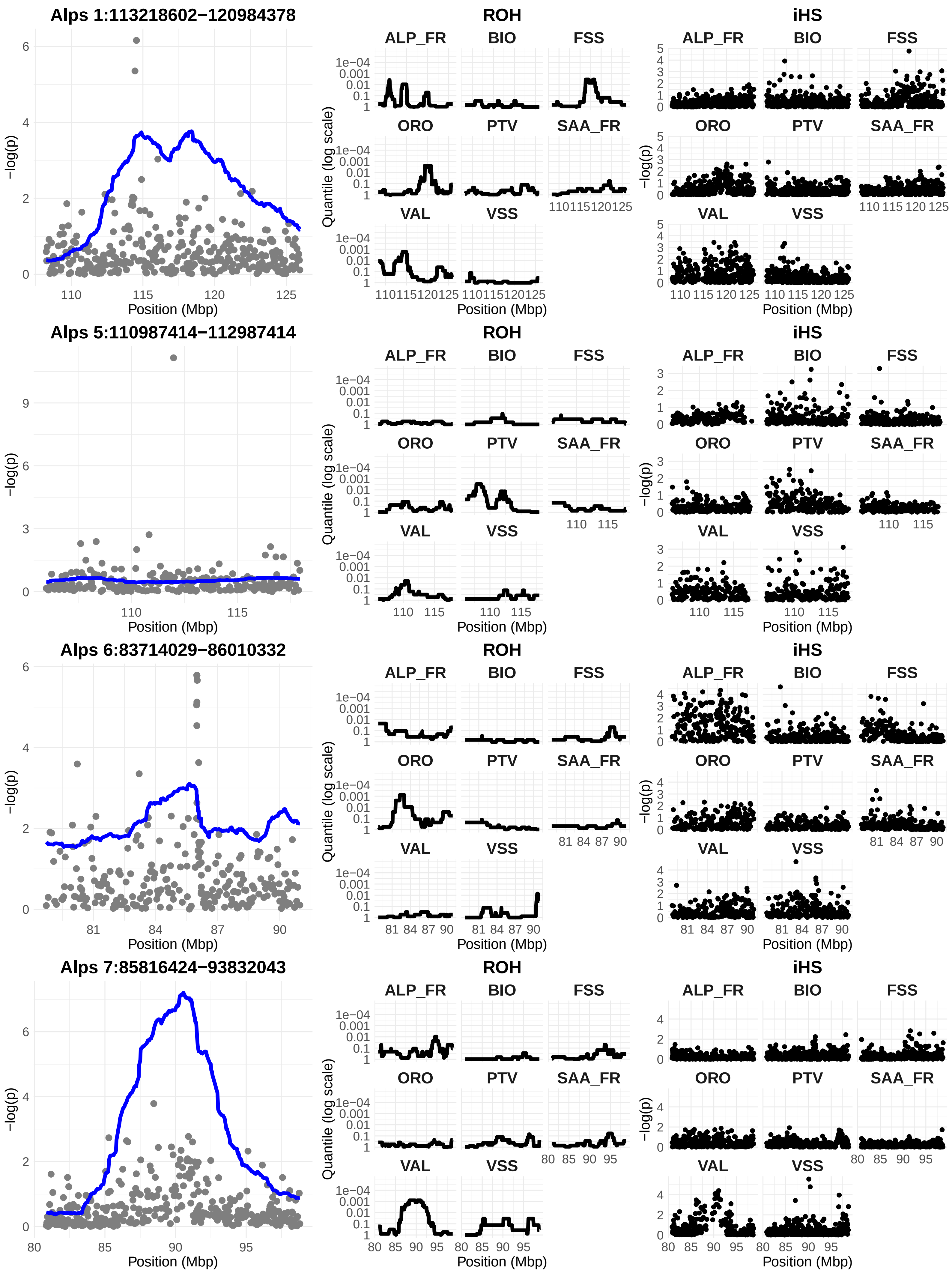
Alps 1:113218602−120984378
ROH
iHS
ALP_FR
BIO
FSS
ORO
PTV
SAA_FR
VAL
VSS
−log(p)
Quantile (log scale)
Position (Mbp)
Alps 5:110987414−112987414
Alps 6:83714029−86010332
Alps 7:85816424−93832043

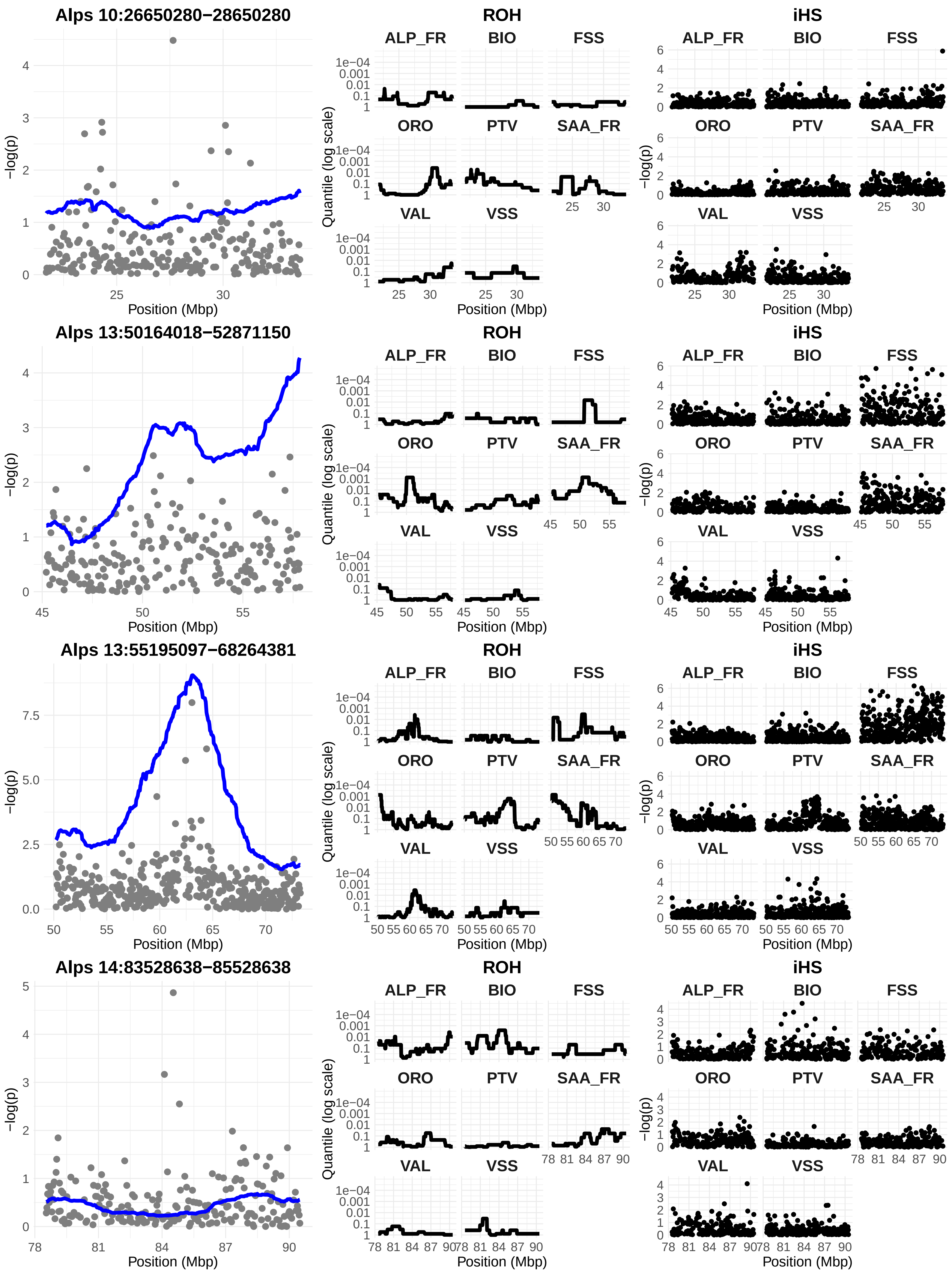
Alps 10:26650280−28650280
ROH
iHS
Alps 13:50164018−52871150
ROH
iHS
Alps 13:55195097−68264381
ROH
iHS
Alps 14:83528638−85528638
ROH
iHS
ALP_FR
BIO
FSS
ORO
PTV
SAA_FR
VAL
VSS
−log(p)
Quantile (log scale)
Position (Mbp)

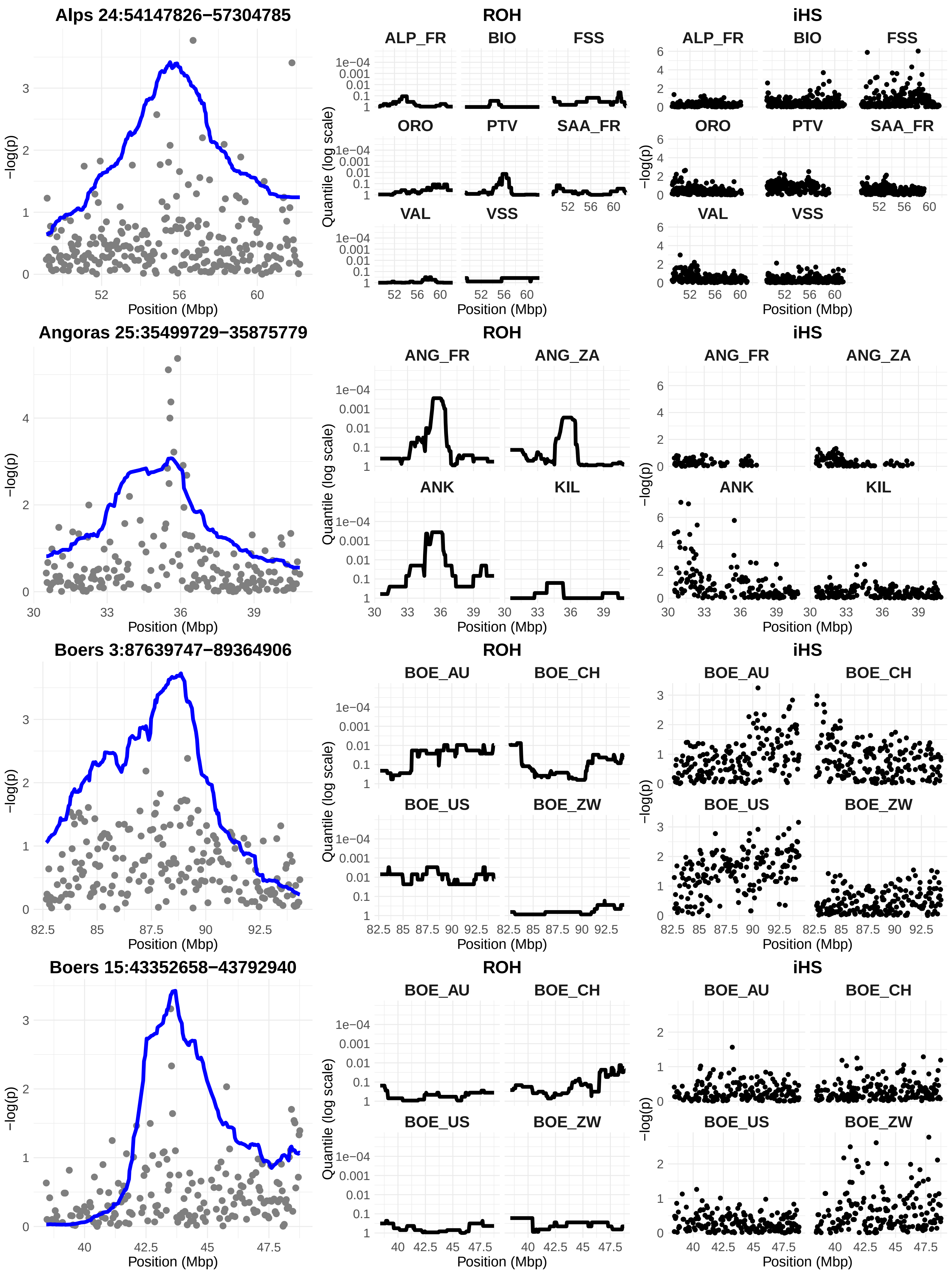
Alps 24:54147826−57304785
ROH
iHS
ALP_FR
BIO
FSS
ORO
PTV
SAA_FR
VAL
VSS
−log(p)
Quantile (log scale)
Position (Mbp)
Angoras 25:35499729−35875779
ANG_FR
ANG_ZA
ANK
KIL
Boers 3:87639747−89364906
BOE_AU
BOE_CH
BOE_US
BOE_ZW
Boers 15:43352658−43792940

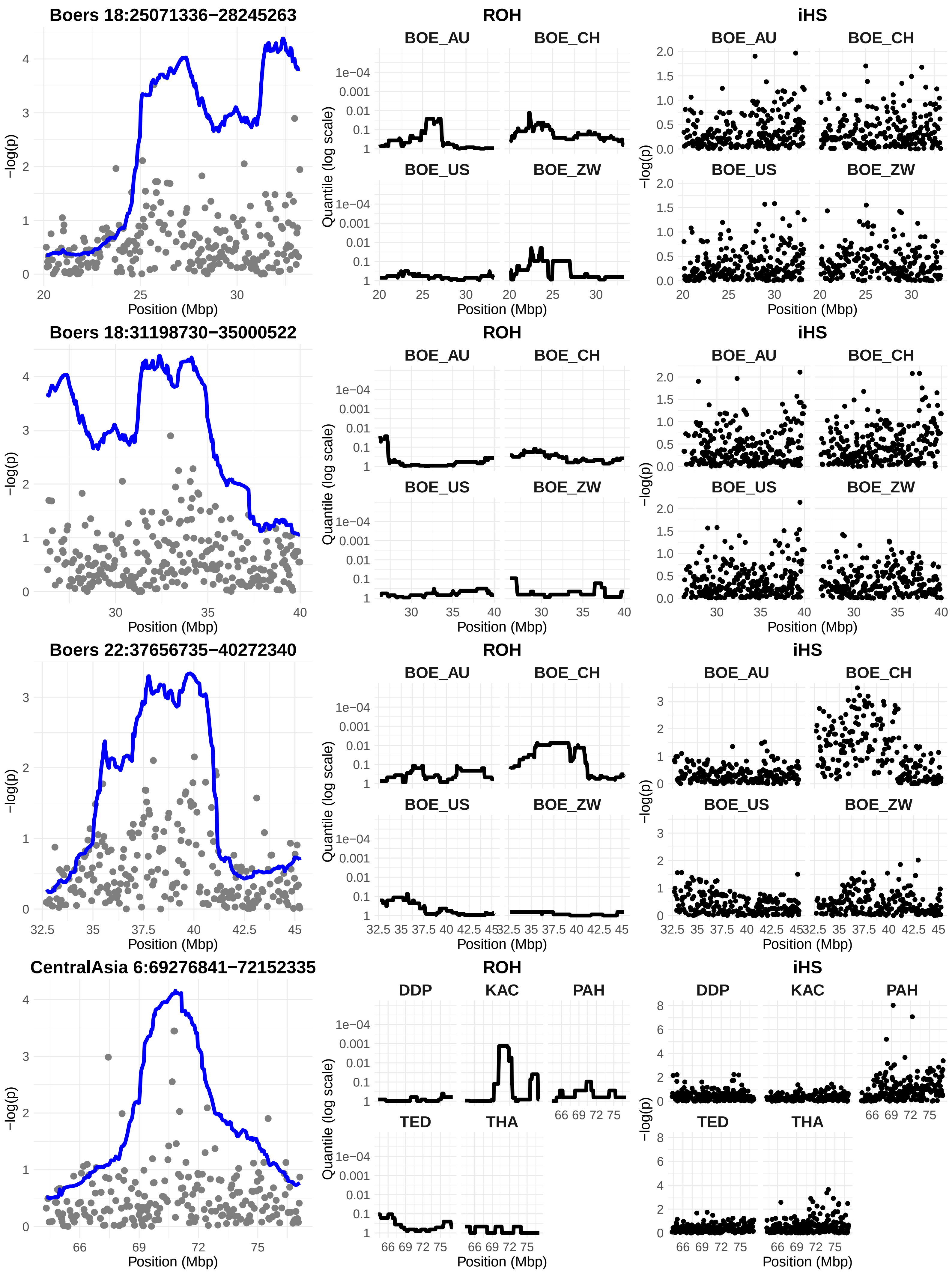

Boers 18:25071336−28245263
ROH
iHS
BOE_AU
BOE_CH
BOE_US
BOE_ZW
−log(p)
Quantile (log scale)
Position (Mbp)
Boers 18:31198730−35000522
ROH
iHS
BOE_AU
BOE_CH
BOE_US
BOE_ZW
−log(p)
Quantile (log scale)
Position (Mbp)
Boers 22:37656735−40272340
ROH
iHS
BOE_AU
BOE_CH
BOE_US
BOE_ZW
−log(p)
Quantile (log scale)
Position (Mbp)
CentralAsia 6:69276841−72152335
ROH
iHS
DDP
KAC
PAH
TED
THA
−log(p)
Quantile (log scale)
Position (Mbp)

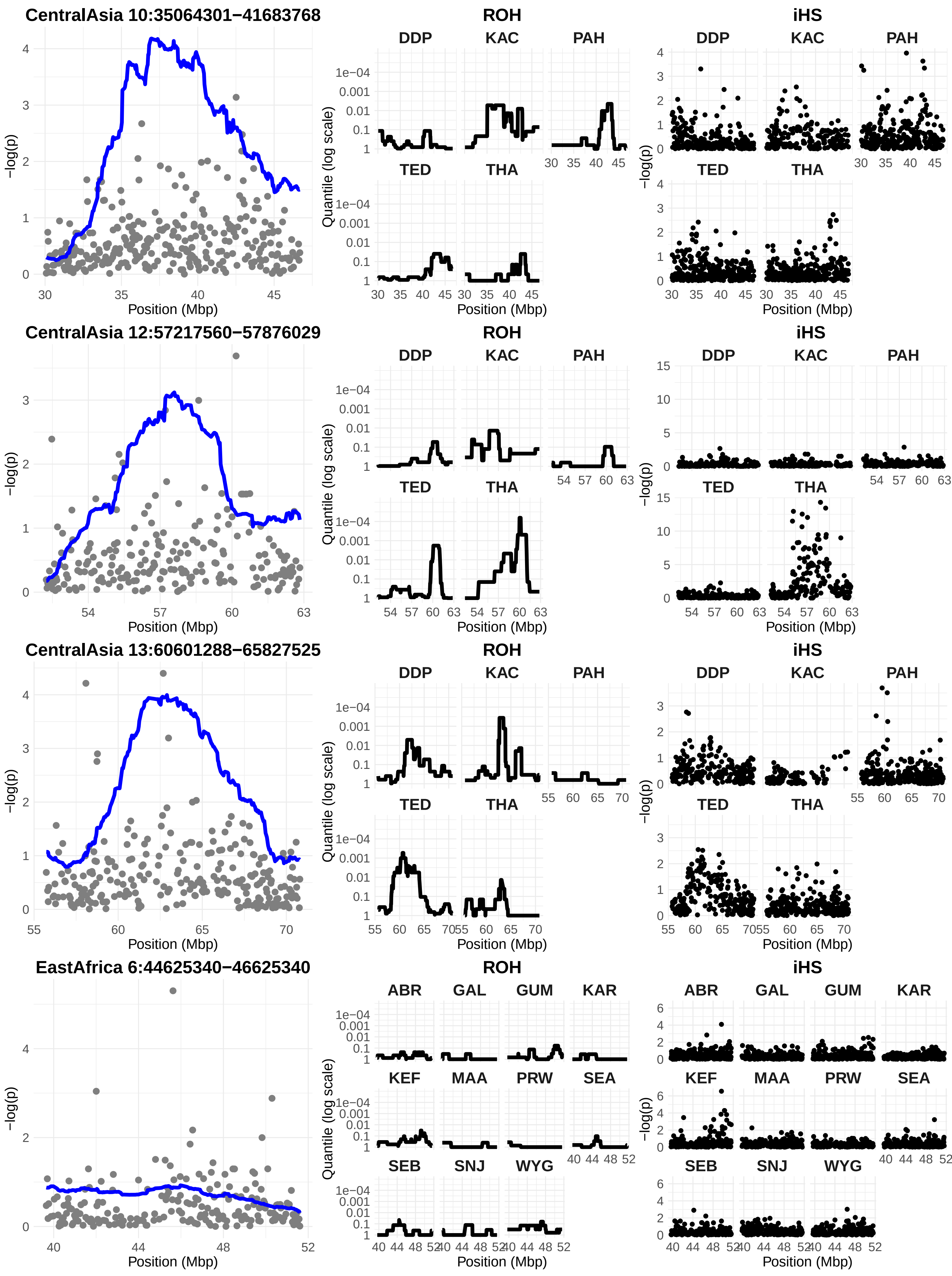
CentralAsia 10:35064301−41683768
ROH
iHS
DDP
KAC
PAH
TED
THA
−log(p)
Quantile (log scale)
Position (Mbp)
CentralAsia 12:57217560−57876029
ROH
iHS
DDP
KAC
PAH
TED
THA
−log(p)
Quantile (log scale)
Position (Mbp)
CentralAsia 13:60601288−65827525
ROH
iHS
DDP
KAC
PAH
TED
THA
−log(p)
Quantile (log scale)
Position (Mbp)
EastAfrica 6:44625340−46625340
ROH
iHS
ABR
GAL
GUM
KAR
KEF
MAA
PRW
SEA
SEB
SNJ
WYG
−log(p)
Quantile (log scale)
Position (Mbp)

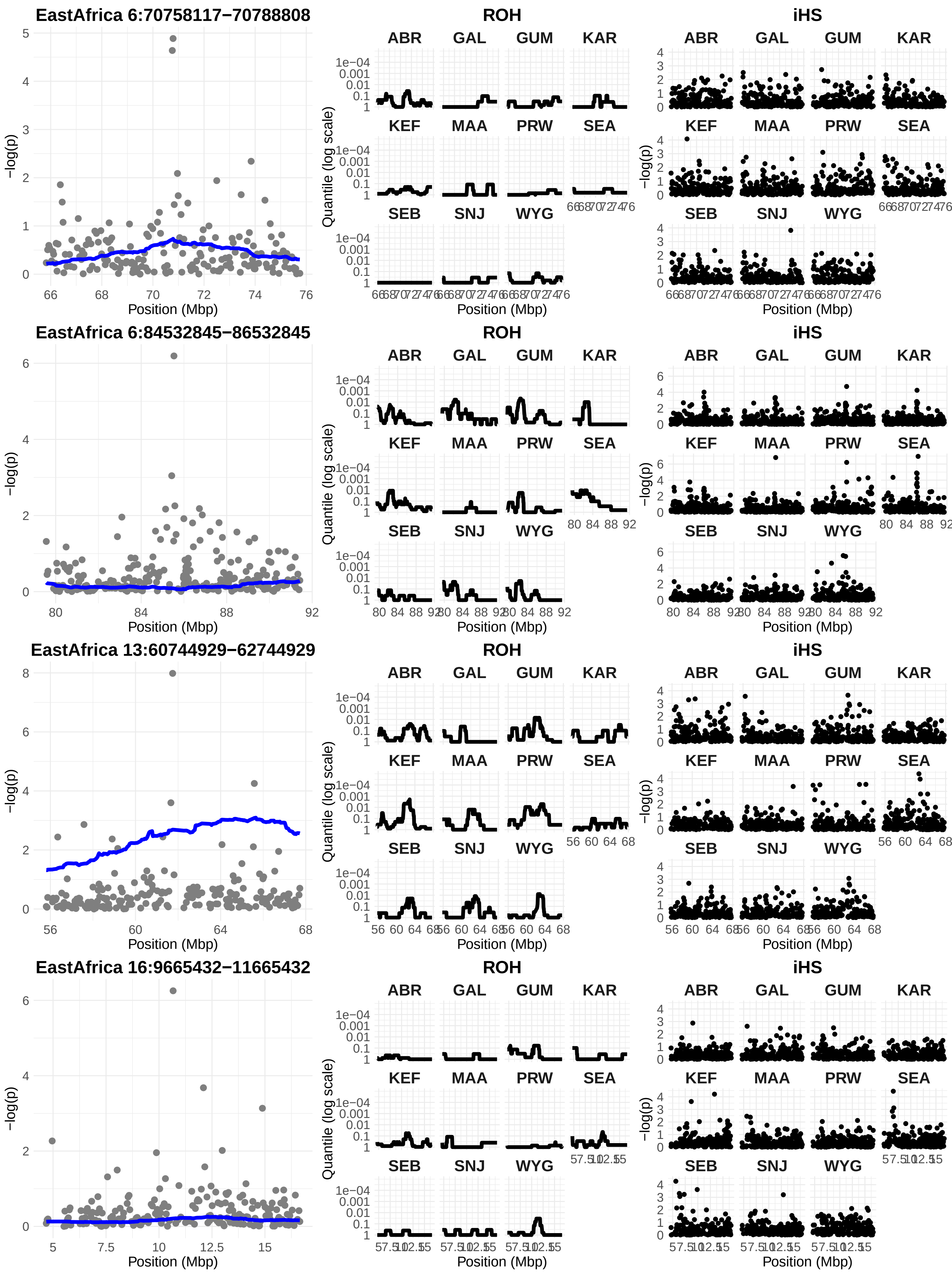

EastAfrica 6:70758117−70788808
−log(p)
Position (Mbp)
ROH
ABR
GAL
GUM
KAR
KEF
MAA
PRW
SEA
SEB
SNJ
WYG
Quantile (log scale)
Position (Mbp)
iHS
ABR
GAL
GUM
KAR
KEF
MAA
PRW
SEA
SEB
SNJ
WYG
−log(p)
Position (Mbp)
EastAfrica 6:84532845−86532845
−log(p)
Position (Mbp)
ROH
Quantile (log scale)
Position (Mbp)
iHS
−log(p)
Position (Mbp)
EastAfrica 13:60744929−62744929
−log(p)
Position (Mbp)
ROH
Quantile (log scale)
Position (Mbp)
iHS
−log(p)
Position (Mbp)
EastAfrica 16:9665432−11665432
−log(p)
Position (Mbp)
ROH
Quantile (log scale)
Position (Mbp)
iHS
−log(p)
Position (Mbp)

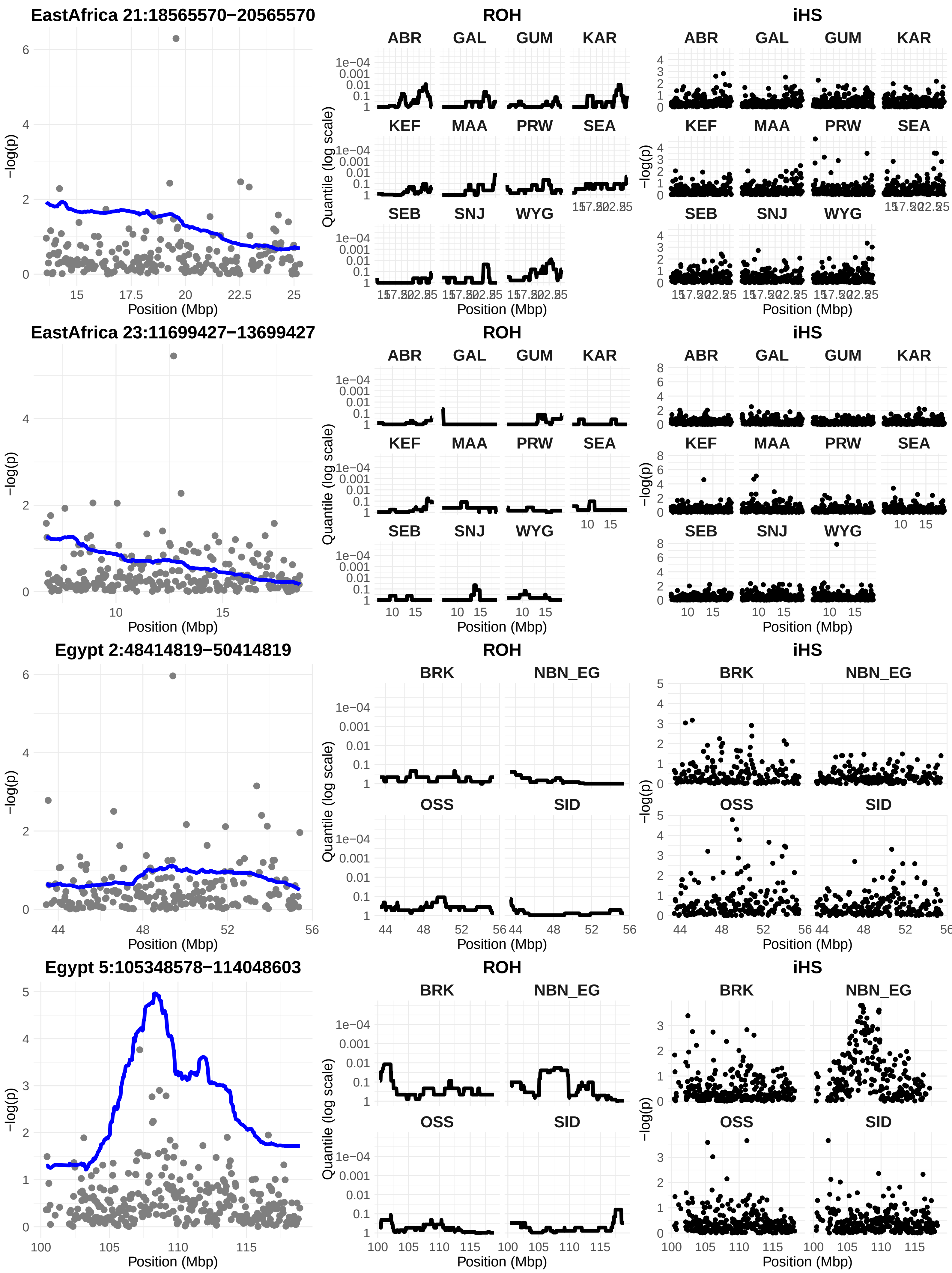

EastAfrica 21:18565570−20565570
ROH
iHS
ABR
GAL
GUM
KAR
KEF
MAA
PRW
SEA
SEB
SNJ
WYG
−log(p)
Quantile (log scale)
Position (Mbp)
EastAfrica 23:11699427−13699427
Egypt 2:48414819−50414819
BRK
NBN_EG
OSS
SID
Egypt 5:105348578−114048603

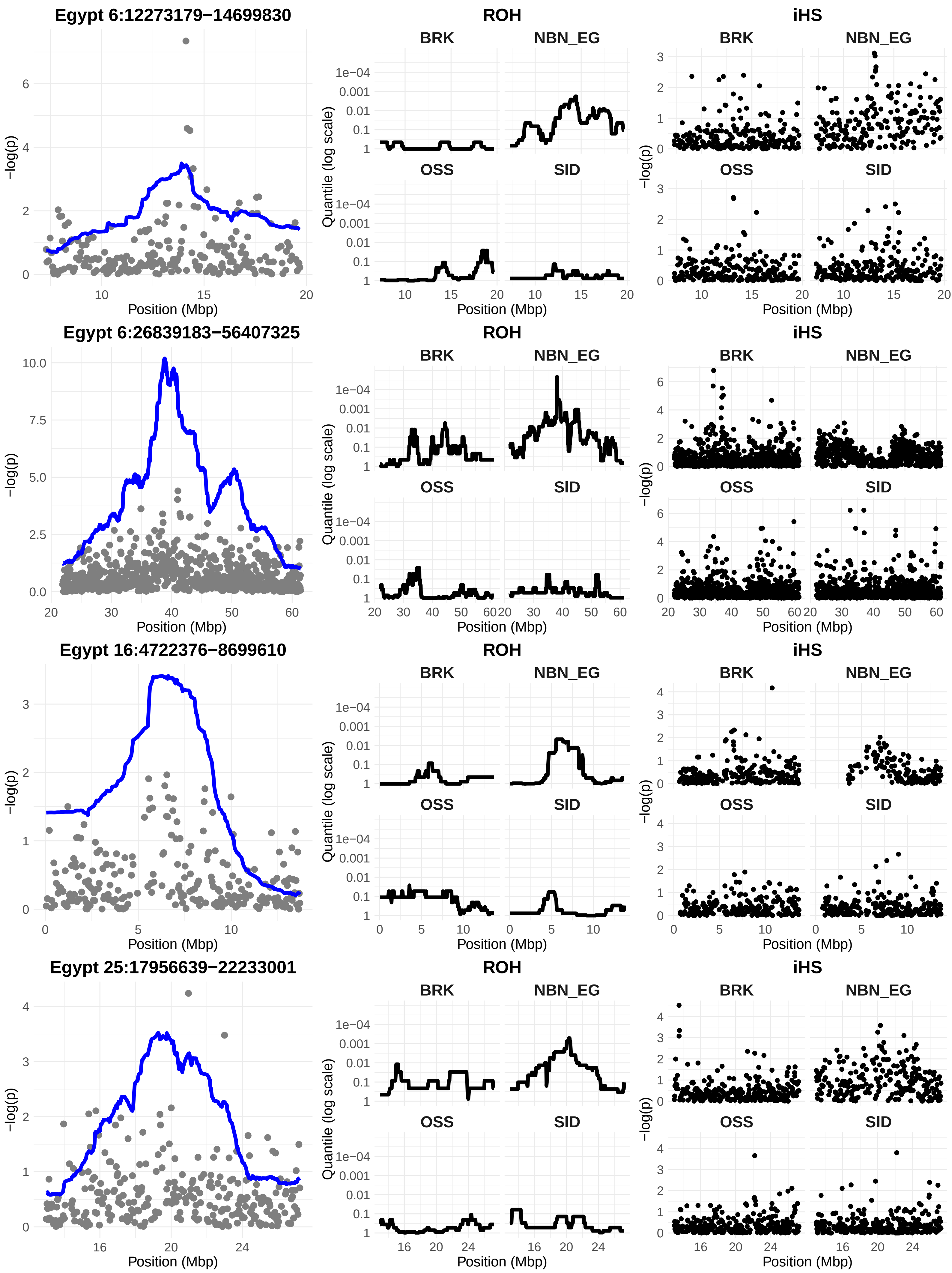

Egypt 6:12273179−14699830
ROH
iHS
BRK
NBN_EG
OSS
SID
−log(p)
Quantile (log scale)
Position (Mbp)
Egypt 6:26839183−56407325
ROH
iHS
BRK
NBN_EG
OSS
SID
−log(p)
Quantile (log scale)
Position (Mbp)
Egypt 16:4722376−8699610
ROH
iHS
BRK
NBN_EG
OSS
SID
−log(p)
Quantile (log scale)
Position (Mbp)
Egypt 25:17956639−22233001
ROH
iHS
BRK
NBN_EG
OSS
SID
−log(p)
Quantile (log scale)
Position (Mbp)

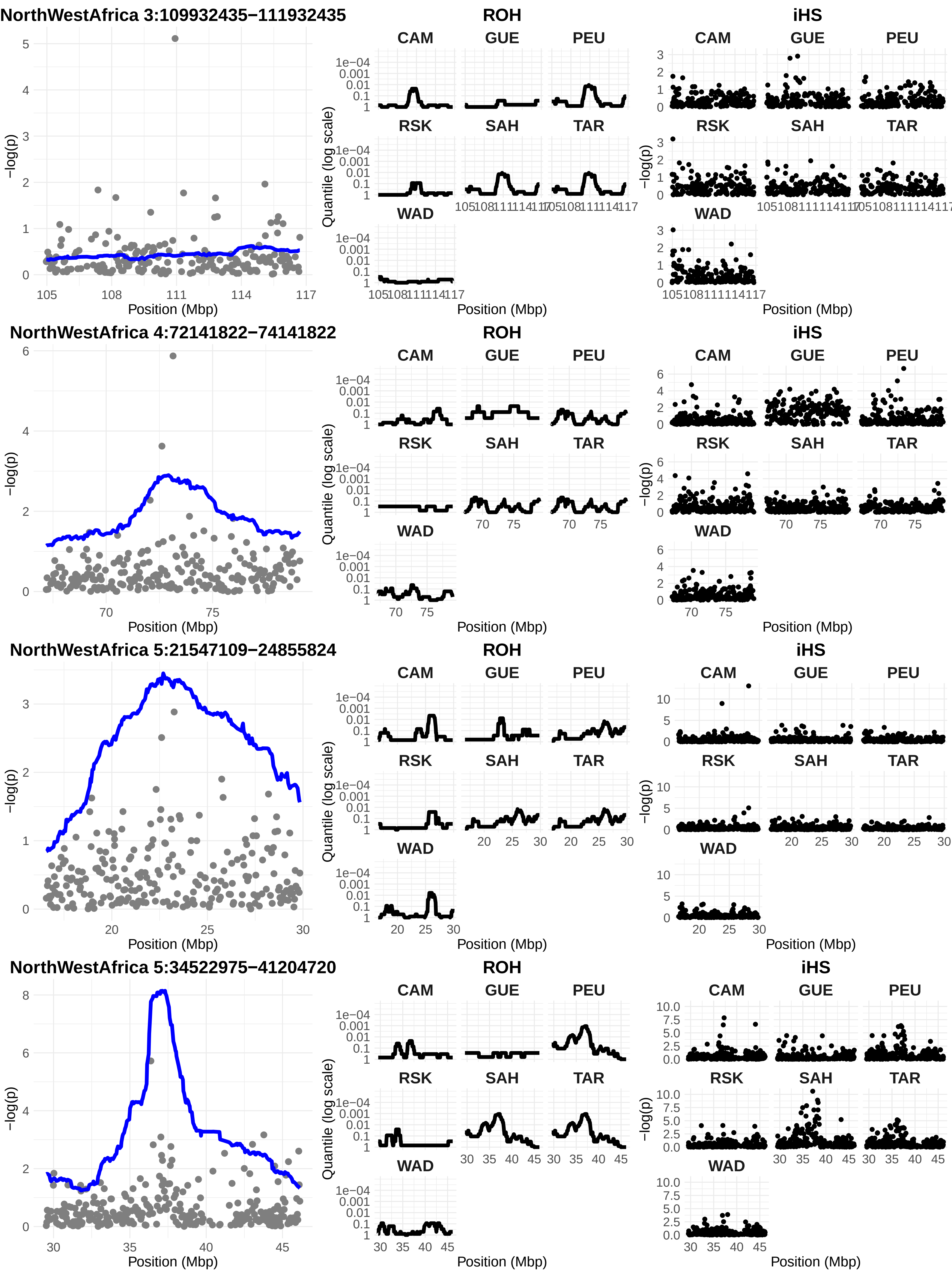
NorthWestAfrica 3:109932435−111932435
ROH
iHS
CAM
GUE
PEU
RSK
SAH
TAR
WAD
−log(p)
Quantile (log scale)
Position (Mbp)
NorthWestAfrica 4:72141822−74141822
ROH
iHS
CAM
GUE
PEU
RSK
SAH
TAR
WAD
−log(p)
Quantile (log scale)
Position (Mbp)
NorthWestAfrica 5:21547109−24855824
ROH
iHS
CAM
GUE
PEU
RSK
SAH
TAR
WAD
−log(p)
Quantile (log scale)
Position (Mbp)
NorthWestAfrica 5:34522975−41204720
ROH
iHS
CAM
GUE
PEU
RSK
SAH
TAR
WAD
−log(p)
Quantile (log scale)
Position (Mbp)

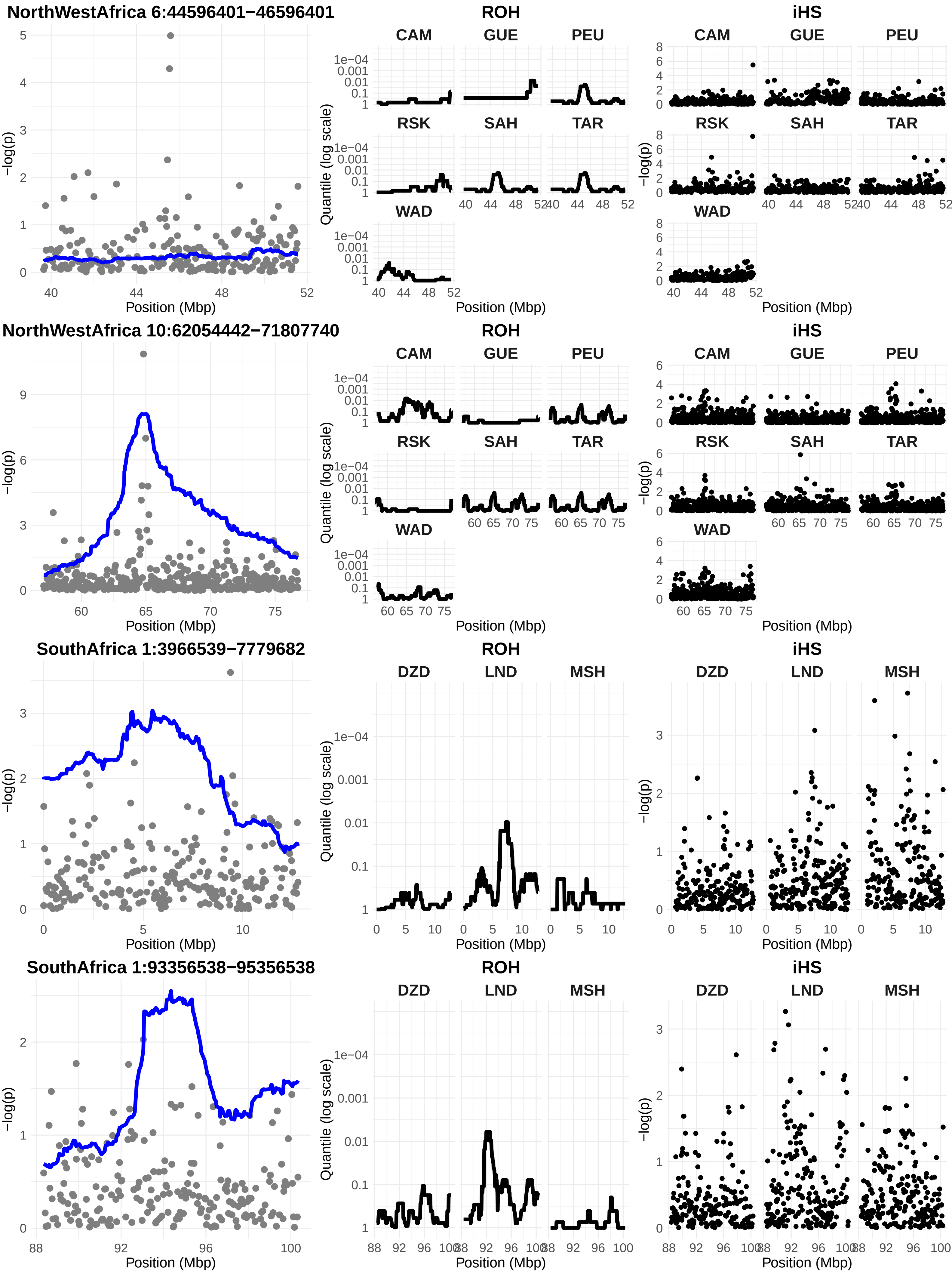
NorthWestAfrica 6:44596401−46596401
ROH
iHS
CAM
GUE
PEU
RSK
SAH
TAR
WAD
−log(p)
Quantile (log scale)
Position (Mbp)
NorthWestAfrica 10:62054442−71807740
SouthAfrica 1:3966539−7779682
DZD
LND
MSH
SouthAfrica 1:93356538−95356538

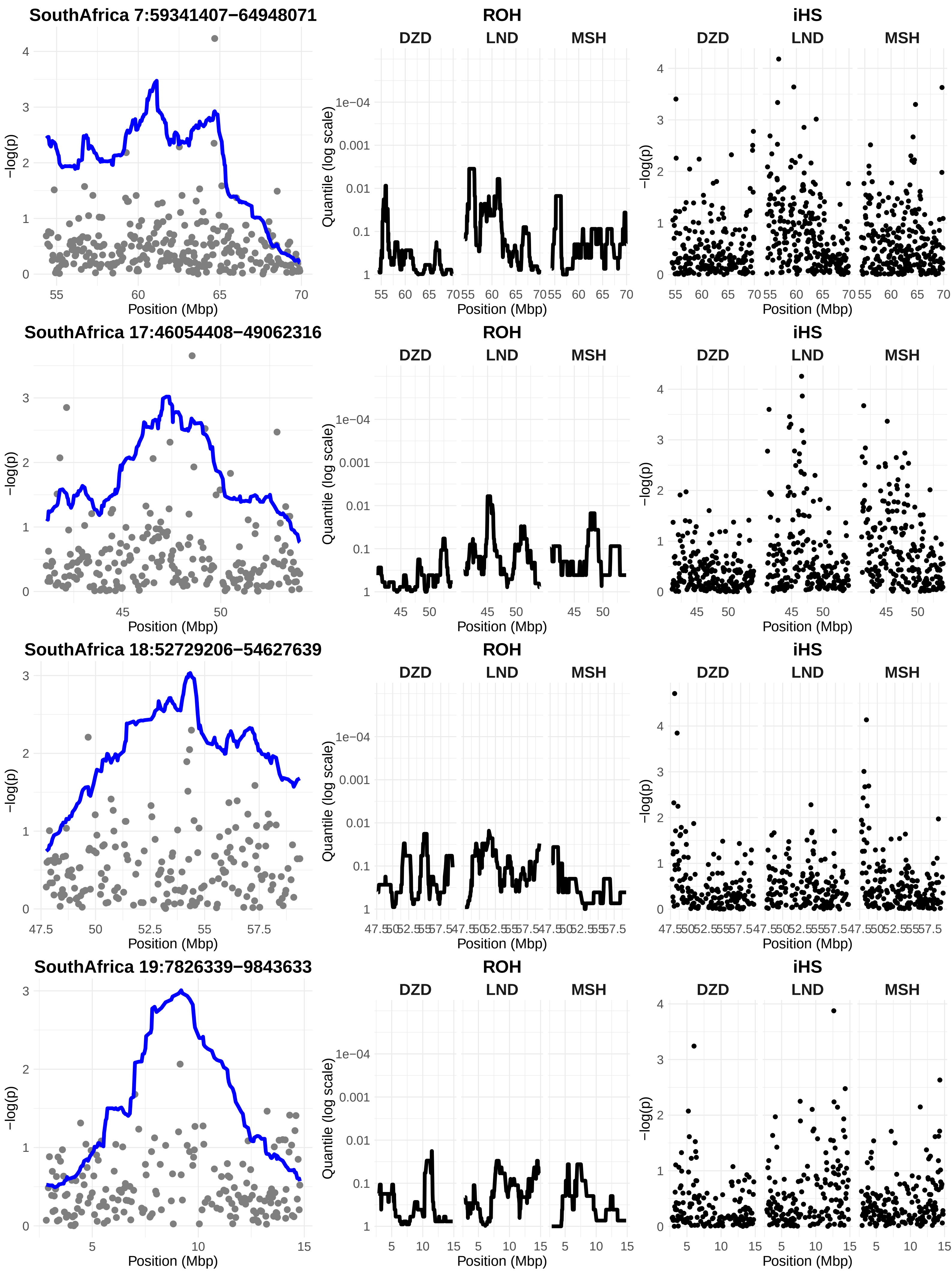

SouthAfrica 7:59341407−64948071
SouthAfrica 17:46054408−49062316
SouthAfrica 18:52729206−54627639
SouthAfrica 19:7826339−9843633
ROH
iHS
DZD
LND
MSH
−log(p)
Quantile (log scale)
Position (Mbp)

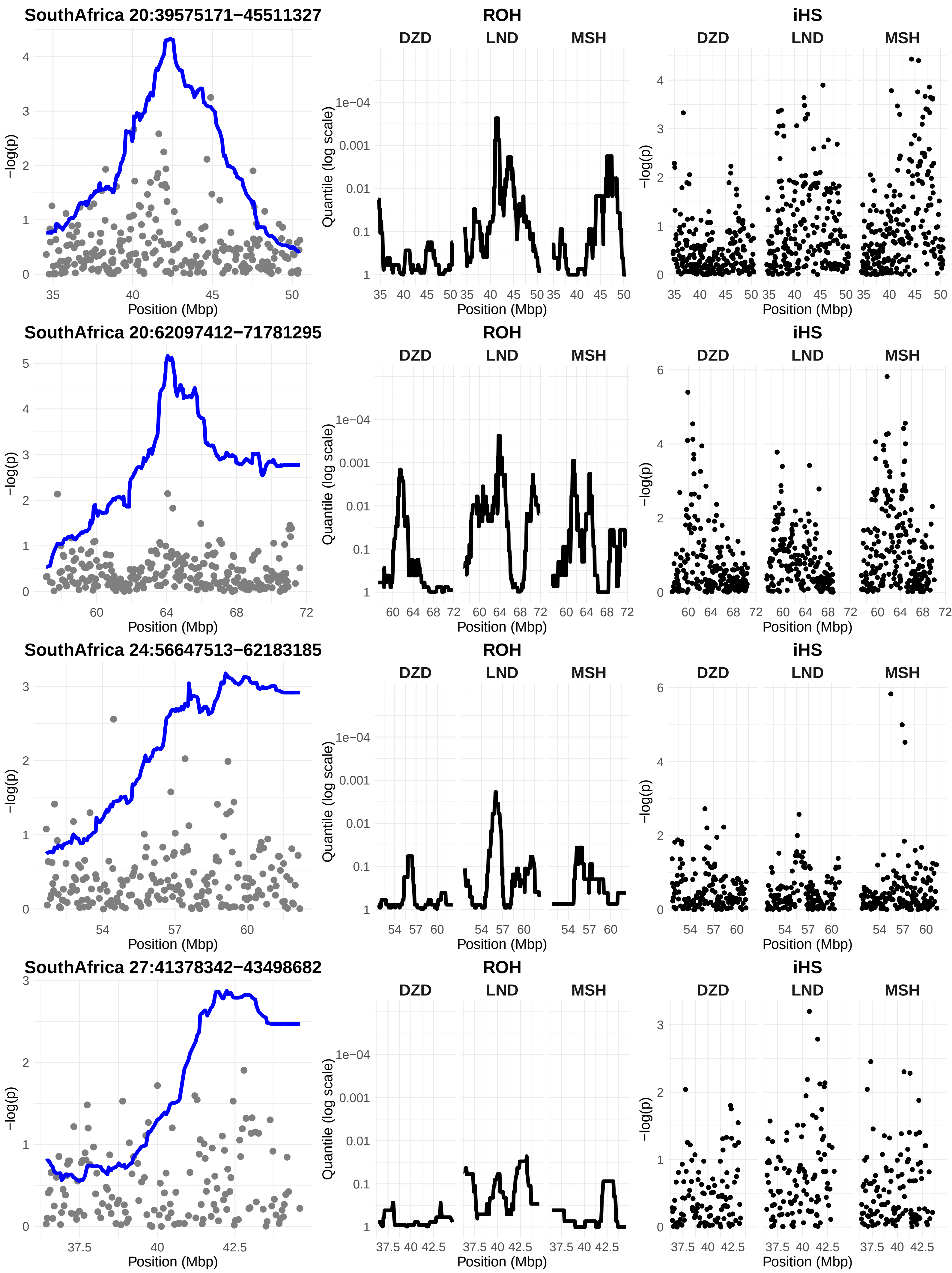

SouthAfrica 20:39575171−45511327
ROH
iHS
DZD
LND
MSH
−log(p)
Quantile (log scale)
Position (Mbp)
SouthAfrica 20:62097412−71781295
SouthAfrica 24:56647513−62183185
SouthAfrica 27:41378342−43498682

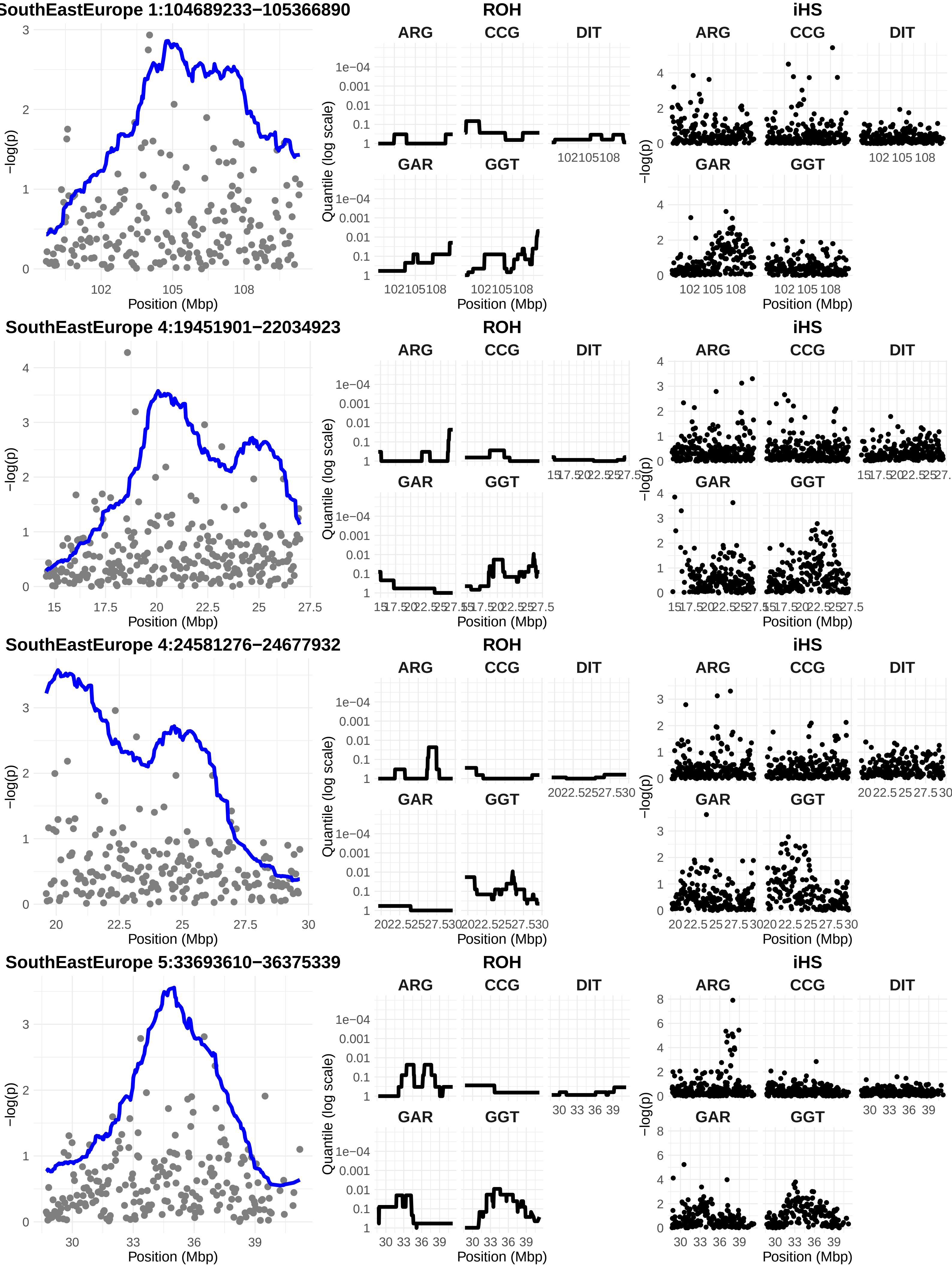
SouthEastEurope 1:104689233−105366890
ROH
iHS
ARG
CCG
DIT
GAR
GGT
−log(p)
Quantile (log scale)
Position (Mbp)
SouthEastEurope 4:19451901−22034923
ROH
iHS
ARG
CCG
DIT
GAR
GGT
−log(p)
Quantile (log scale)
Position (Mbp)
SouthEastEurope 4:24581276−24677932
ROH
iHS
ARG
CCG
DIT
GAR
GGT
−log(p)
Quantile (log scale)
Position (Mbp)
SouthEastEurope 5:33693610−36375339
ROH
iHS
ARG
CCG
DIT
GAR
GGT
−log(p)
Quantile (log scale)
Position (Mbp)

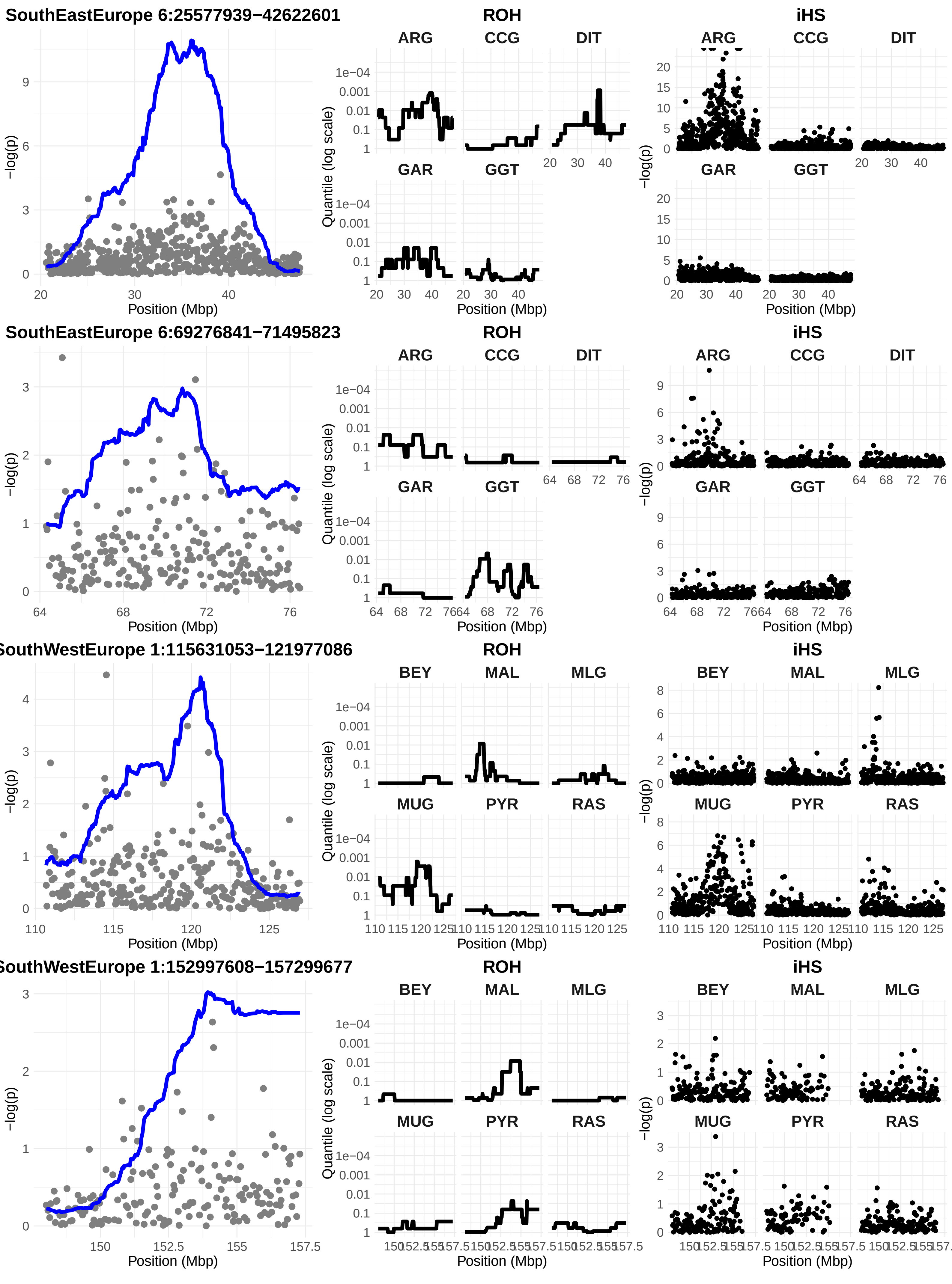
SouthEastEurope 6:25577939−42622601
ROH
iHS
ARG
CCG
DIT
GAR
GGT
−log(p)
Quantile (log scale)
Position (Mbp)
SouthEastEurope 6:69276841−71495823
SouthWestEurope 1:115631053−121977086
BEY
MAL
MLG
MUG
PYR
RAS
SouthWestEurope 1:152997608−157299677

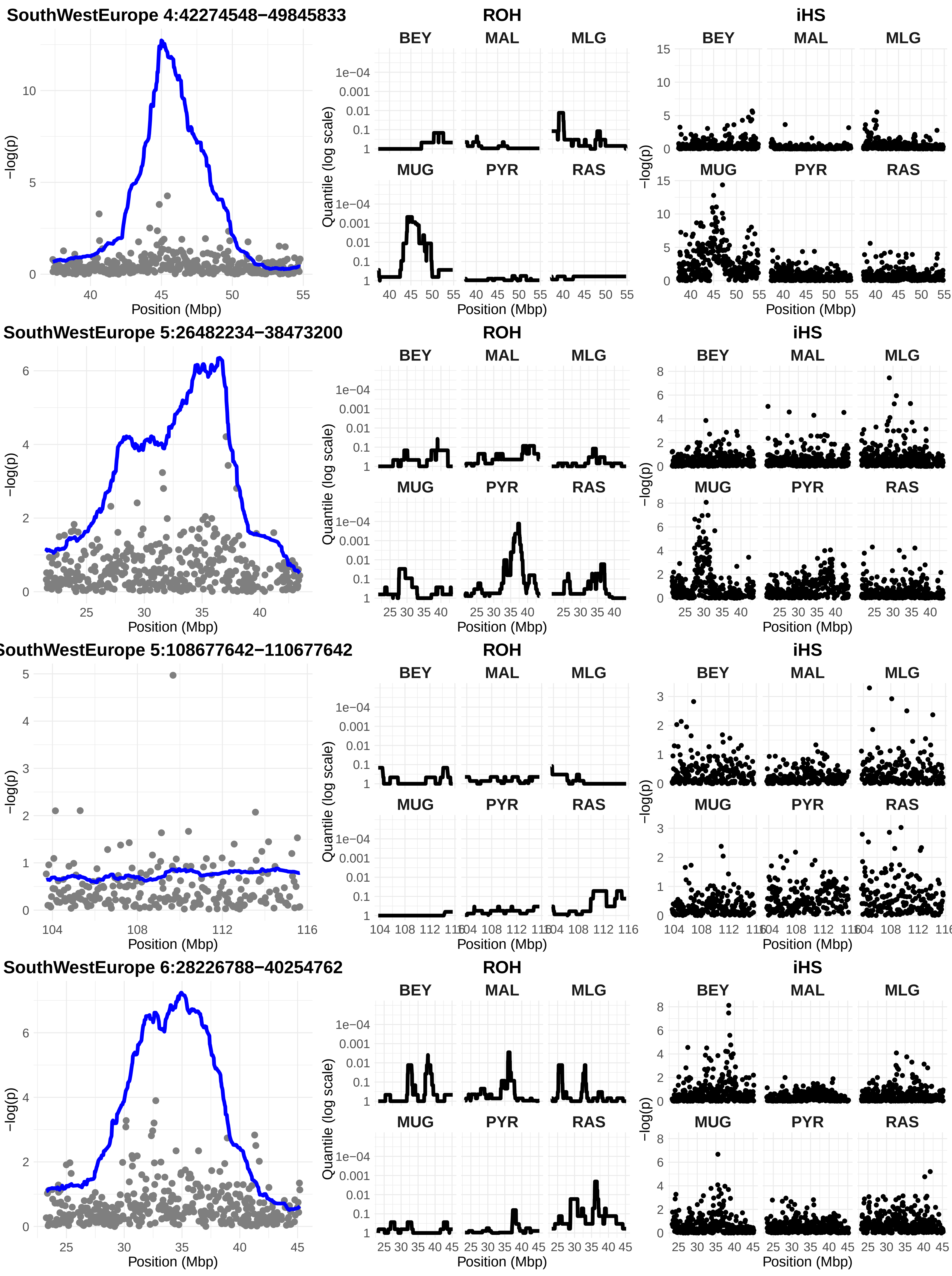
SouthWestEurope 4:42274548−49845833
ROH
iHS
BEY
MAL
MLG
MUG
PYR
RAS
−log(p)
Quantile (log scale)
Position (Mbp)
SouthWestEurope 5:26482234−38473200
SouthWestEurope 5:108677642−110677642
SouthWestEurope 6:28226788−40254762
1e−04
0.001
0.01
0.1
1

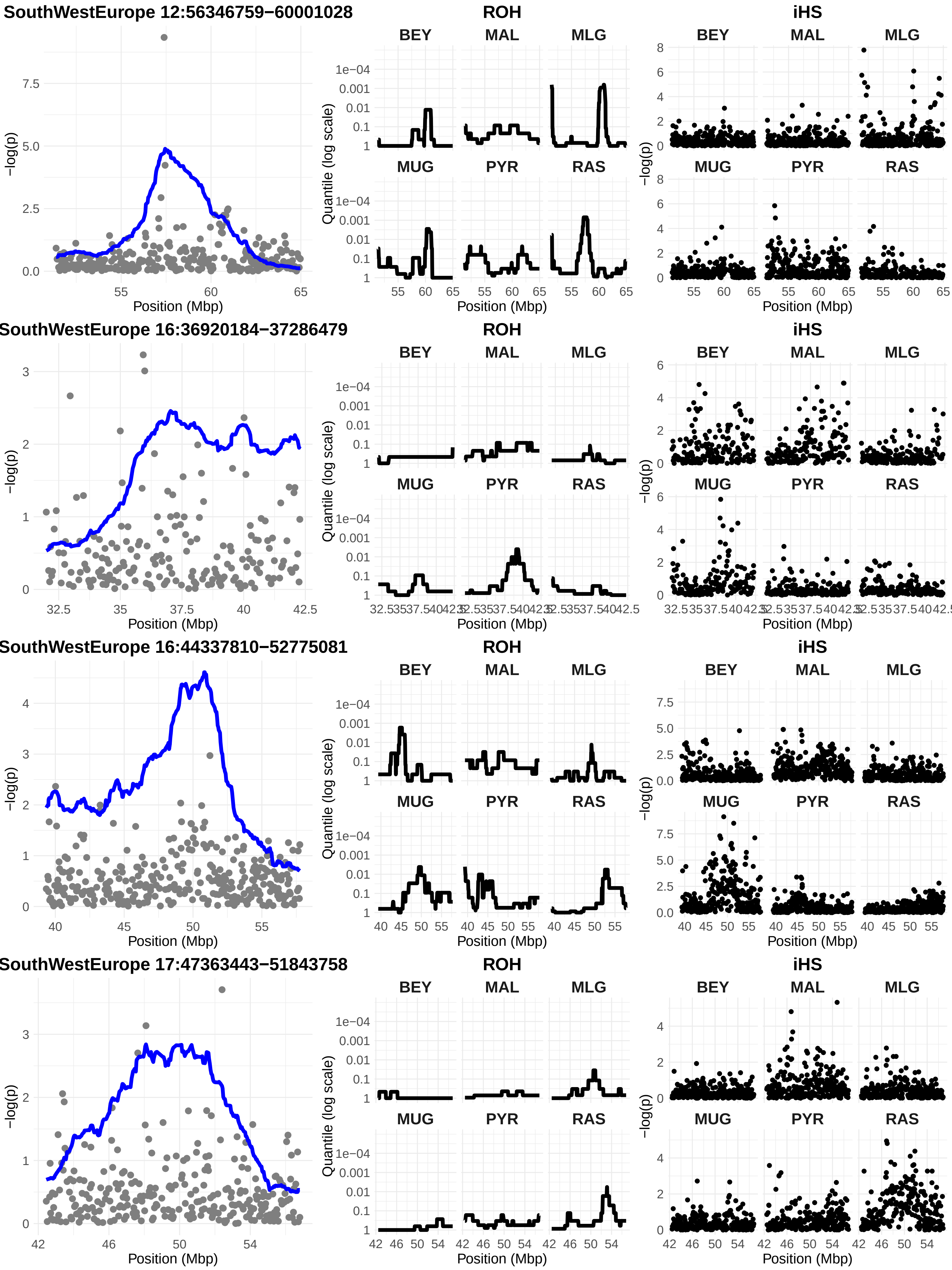

SouthWestEurope 12:56346759−60001028
ROH
iHS
BEY
MAL
MLG
MUG
PYR
RAS
−log(p)
Quantile (log scale)
Position (Mbp)
SouthWestEurope 16:36920184−37286479
SouthWestEurope 16:44337810−52775081
SouthWestEurope 17:47363443−51843758

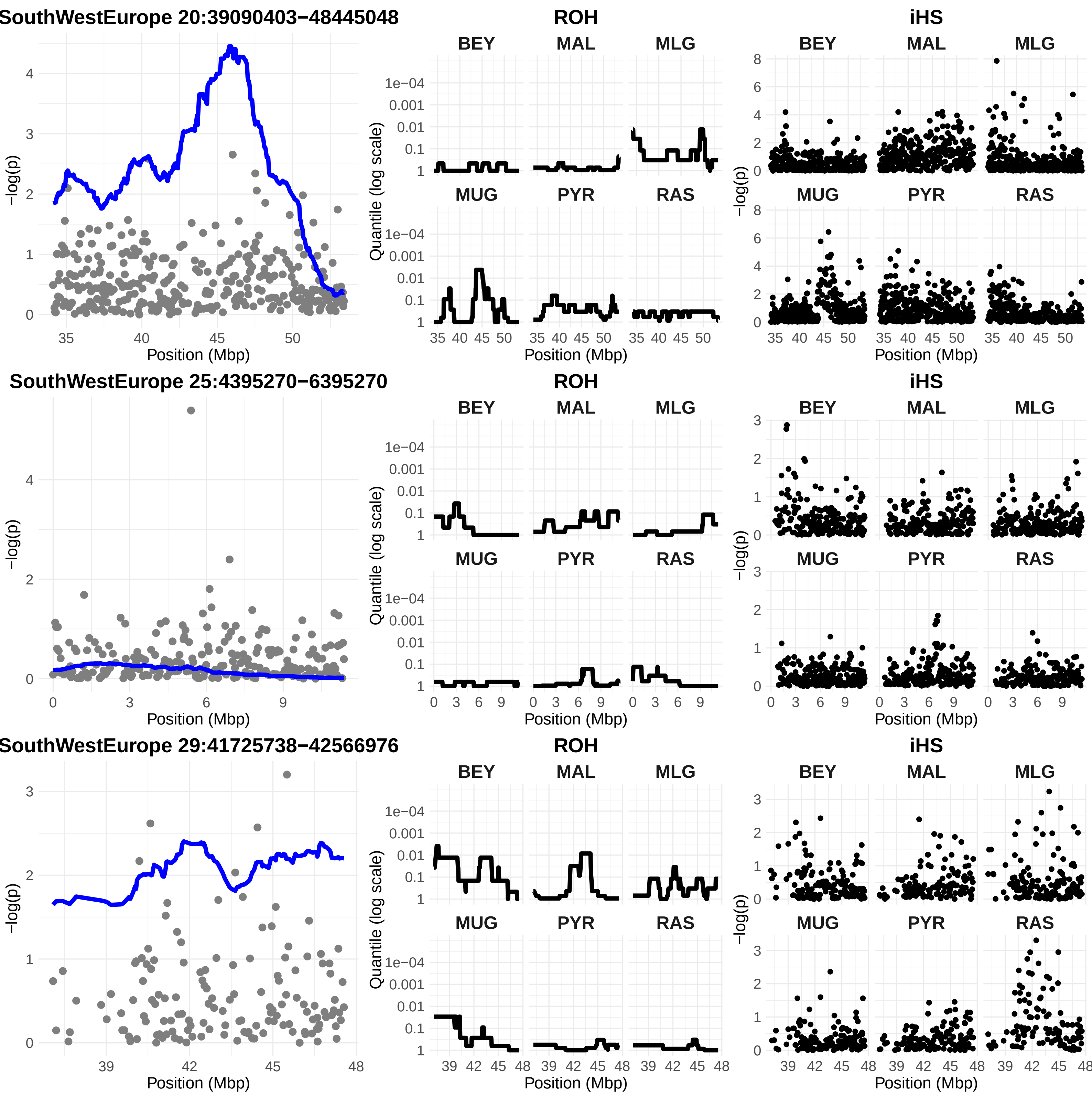
SouthWestEurope 20:39090403−48445048
ROH
iHS
BEY
MAL
MLG
MUG
PYR
RAS
−log(p)
Quantile (log scale)
Position (Mbp)
SouthWestEurope 25:4395270−6395270
ROH
iHS
BEY
MAL
MLG
MUG
PYR
RAS
−log(p)
Quantile (log scale)
Position (Mbp)
SouthWestEurope 29:41725738−42566976
ROH
iHS
BEY
MAL
MLG
MUG
PYR
RAS
−log(p)
Quantile (log scale)
Position (Mbp)
