# Supplementary material for: Signatures of selection and environmental adaptation across the goat genome post-domestication
Source: Genet Sel Evol. 2018 Nov 19;50:57. doi: 10.1186/s12711-018-0421-y (PMC6240954; doi:10.1186/s12711-018-0421-y)

snp15124-scaffold1614-514416

Chromosome: 9 : 20632

Best association

Environmental variable = bio1

G score = 645.14

Beta 1 = 1.65

AIC = 1645.86

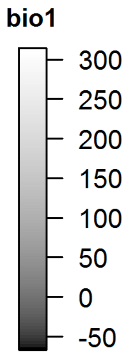

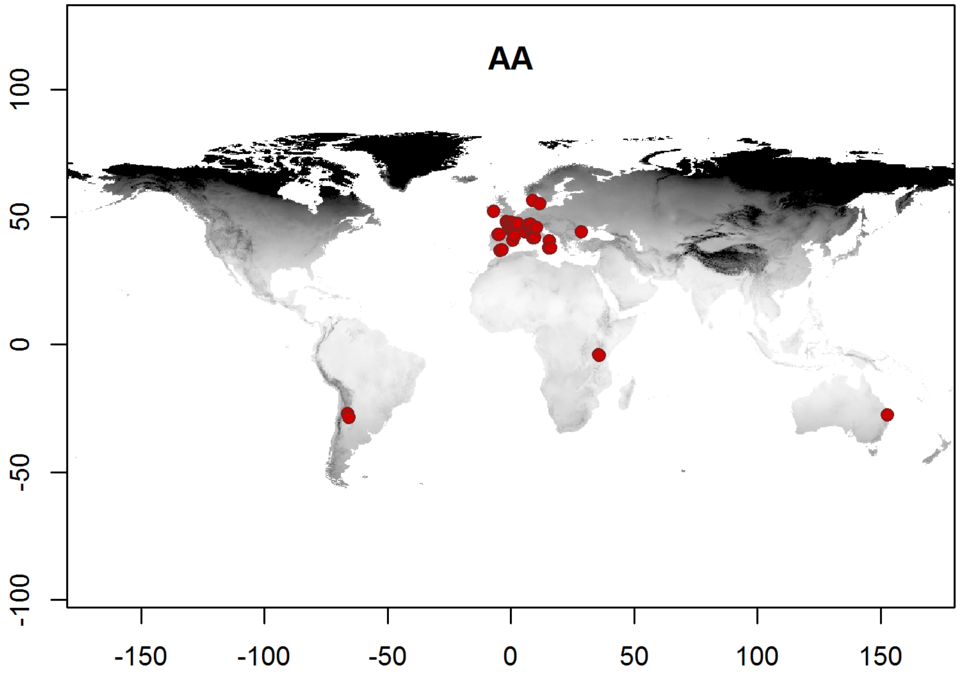

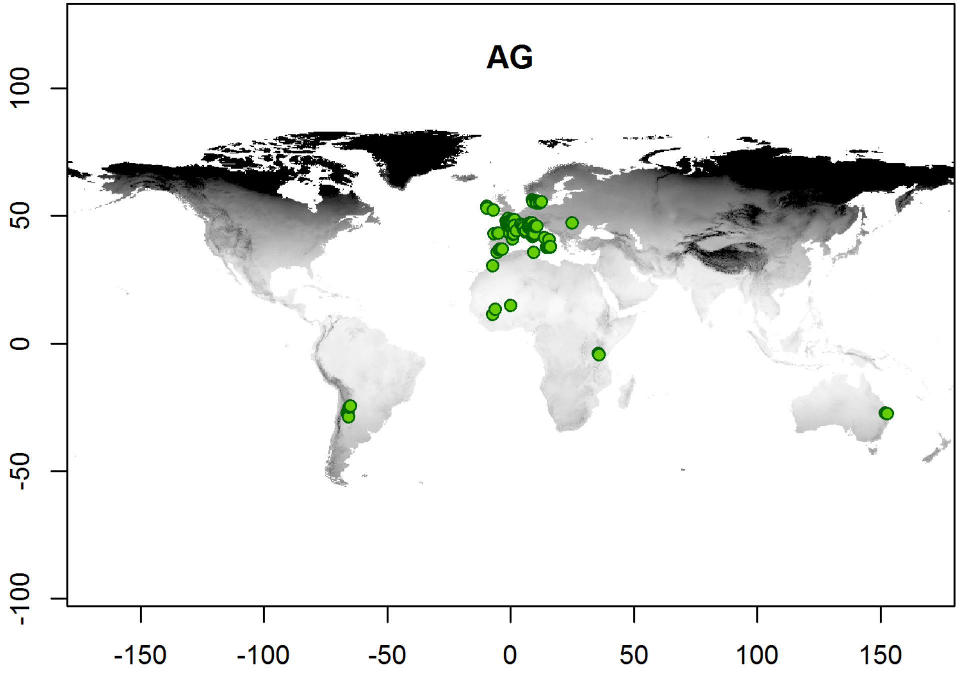

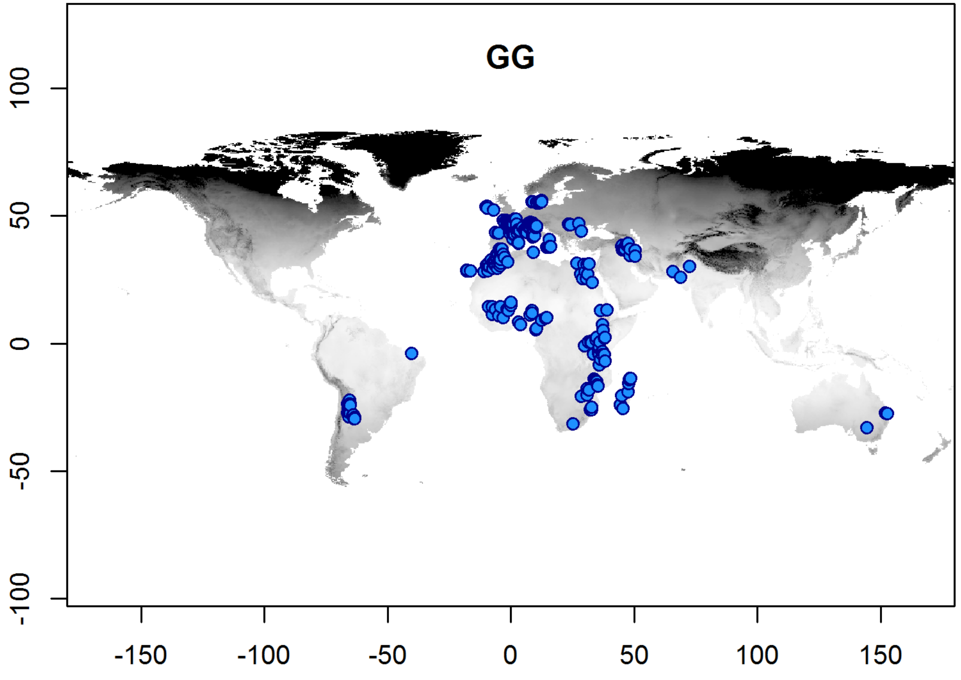

snp14167-scaffold1566-1187146

Chromosome: 4 : 90982814

Best association

Environmental variable = bio1

G score = 556.41

Beta 1 = 1.53

AIC = 1656.38

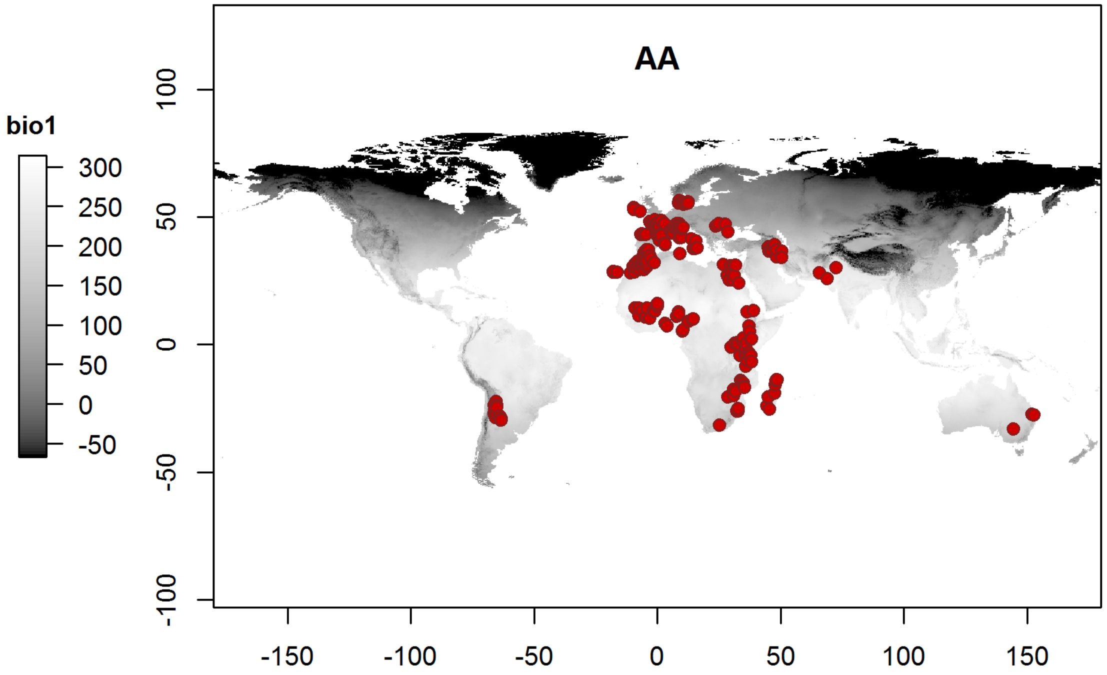

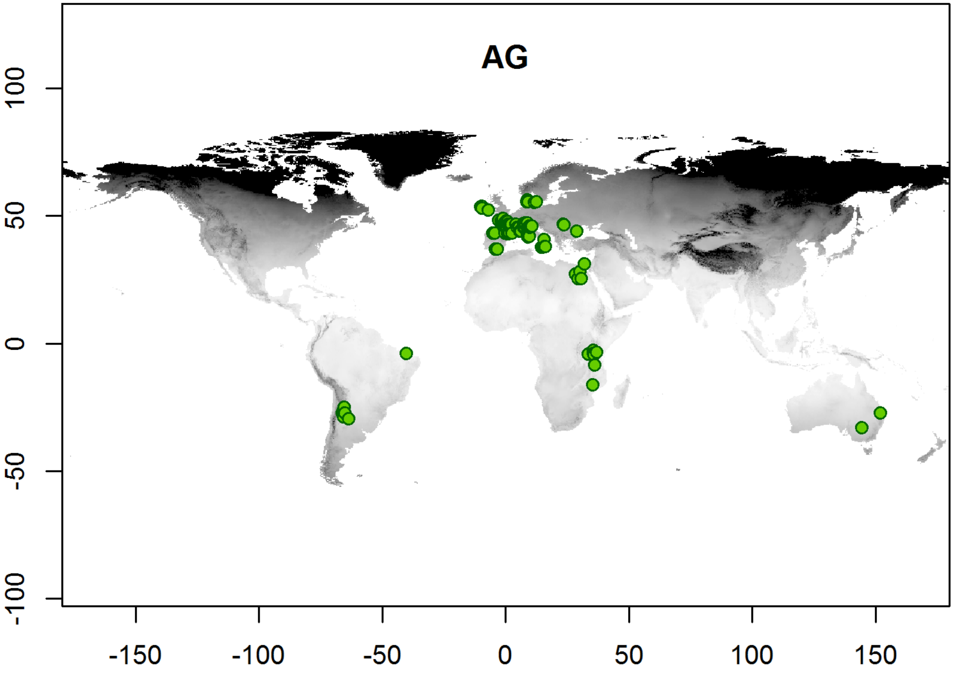

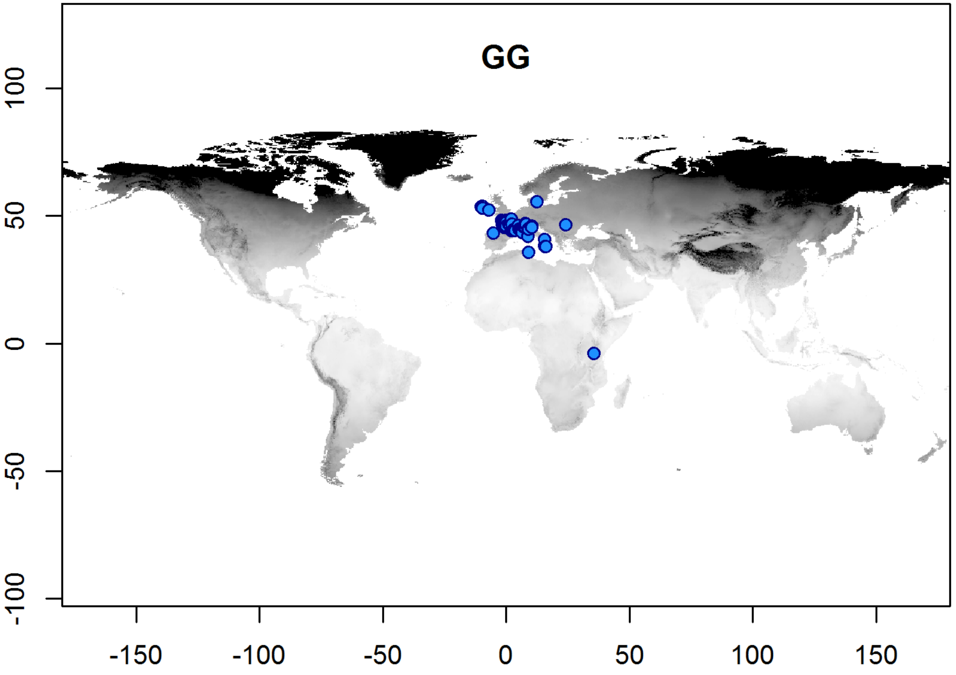

snp13612-scaffold1526-472688

Chromosome: 12 : 10476413

Best association

Environmental variable = bio1

G score = 738.5

Beta 1 = 1.59

AIC = 1913.28

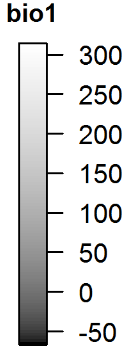

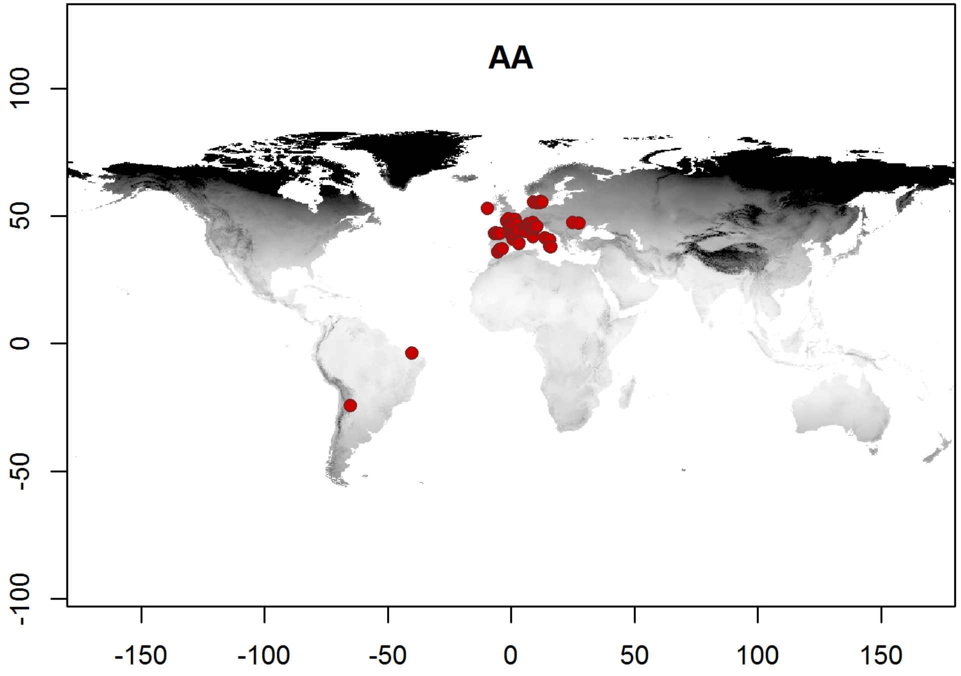

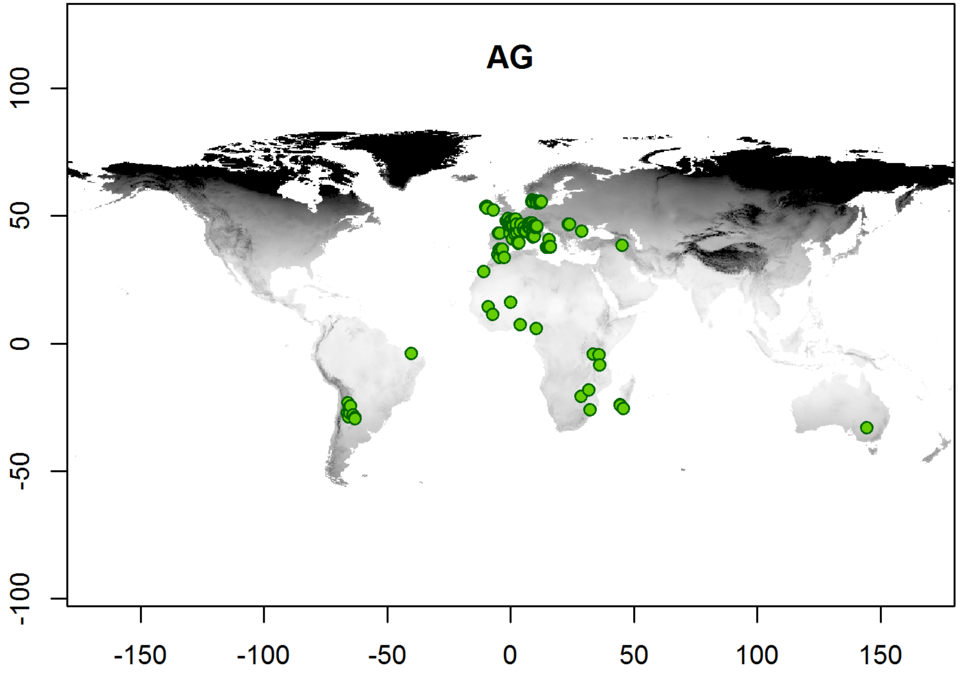

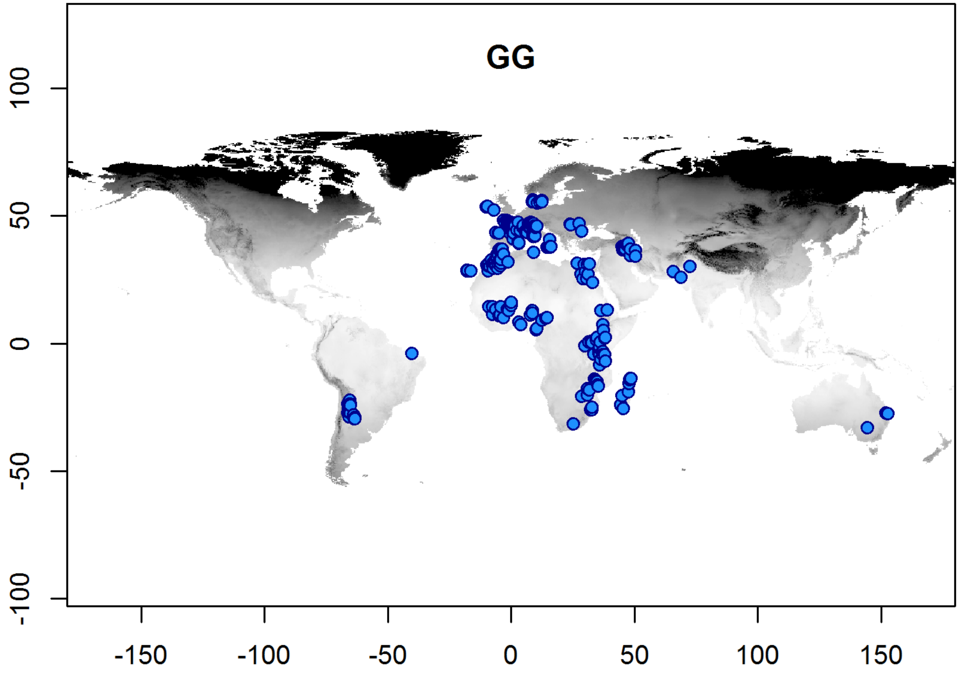

snp12120-scaffold1448-789150

Chromosome: 17 : 8947492

Best association

Environmental variable = bio1

G score = 870.62

Beta 1 = 1.98

AIC = 1577

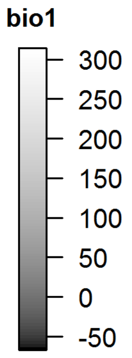

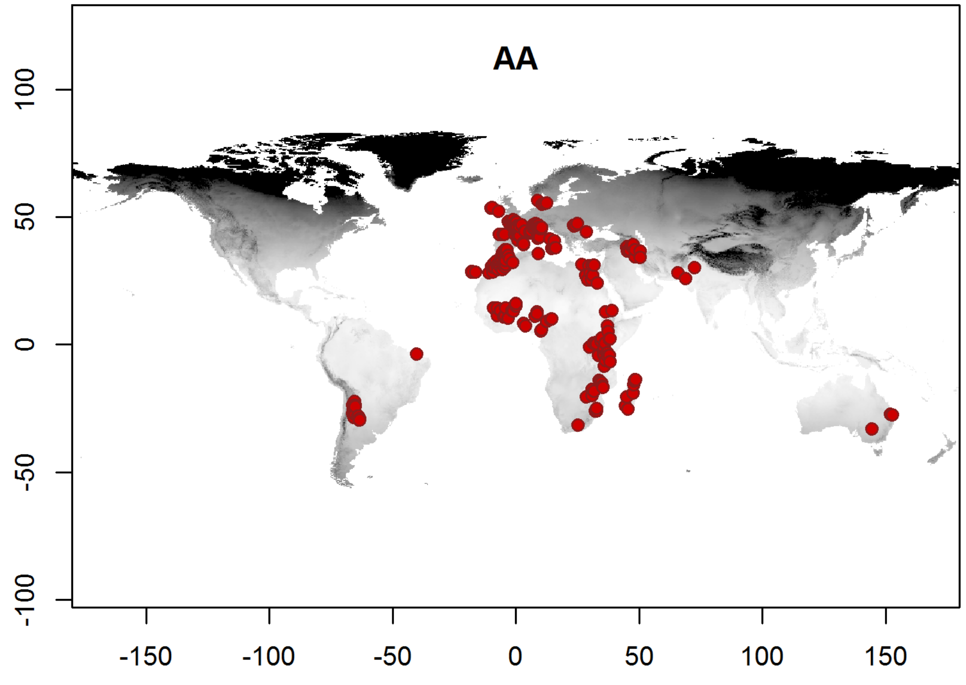

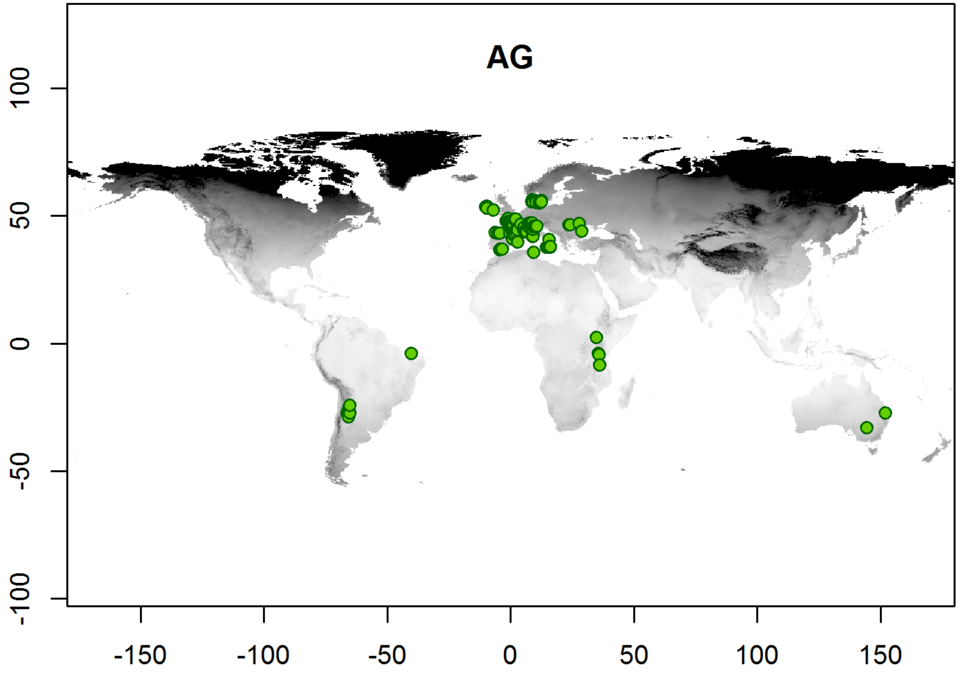

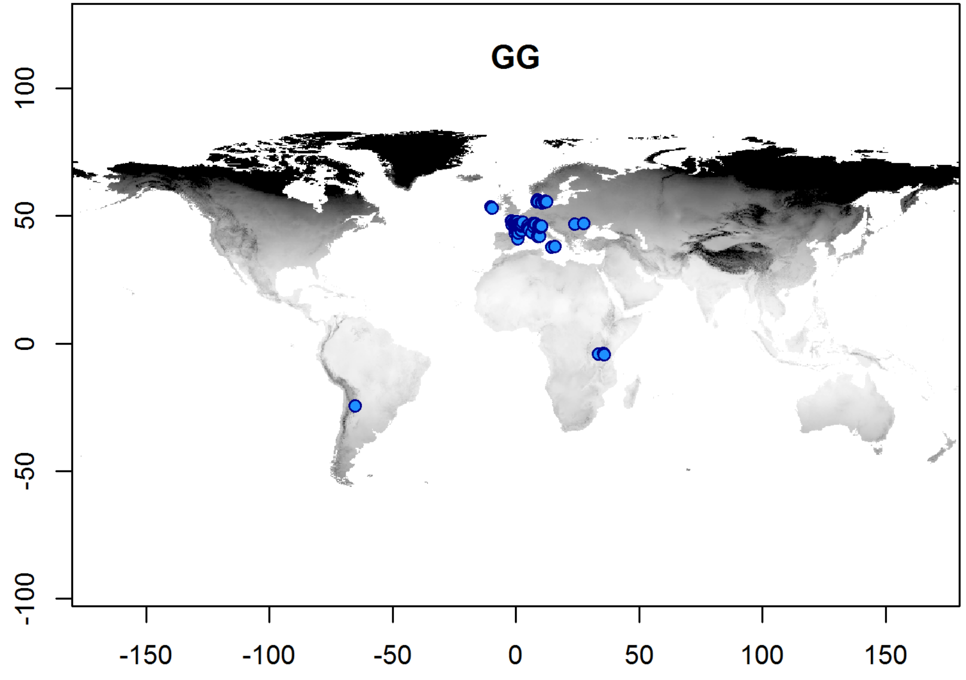

snp7527-scaffold127-6291318

Chromosome: 2 : 22057270

Best association

Environmental variable = bio1

G score = 746.41

Beta 1 = 1.69

AIC = 1768.64

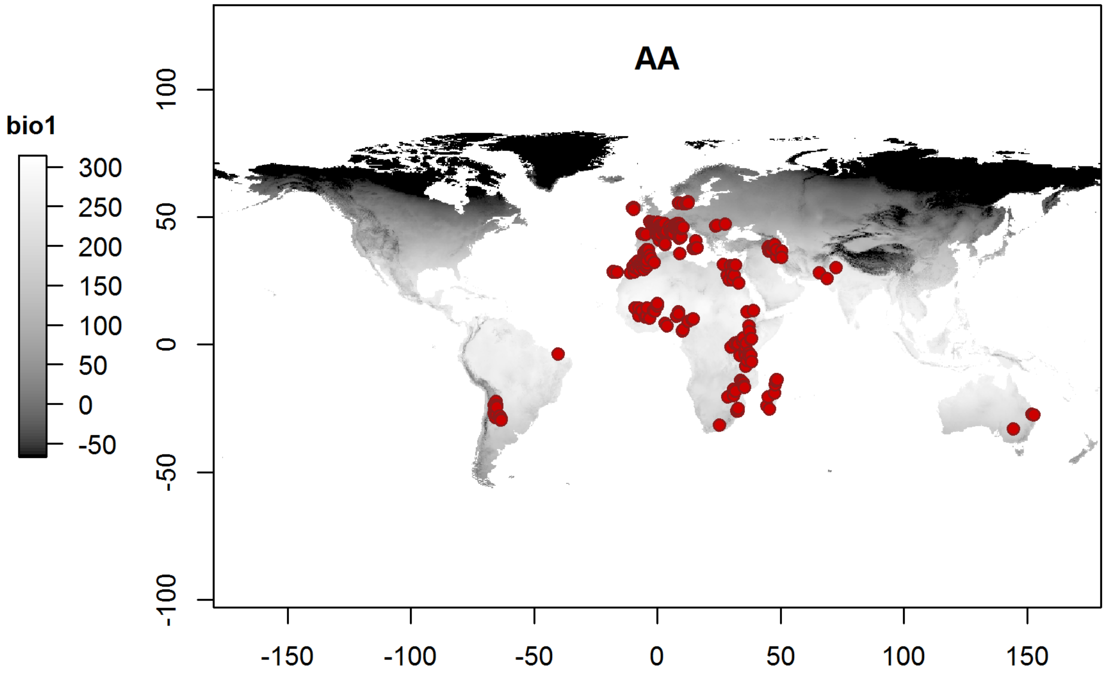

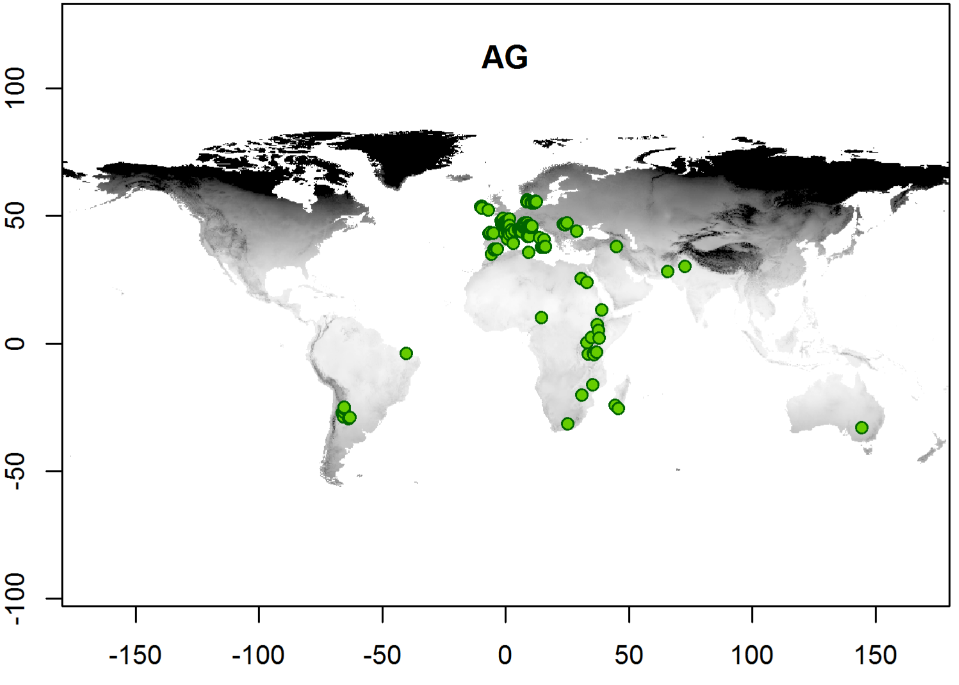

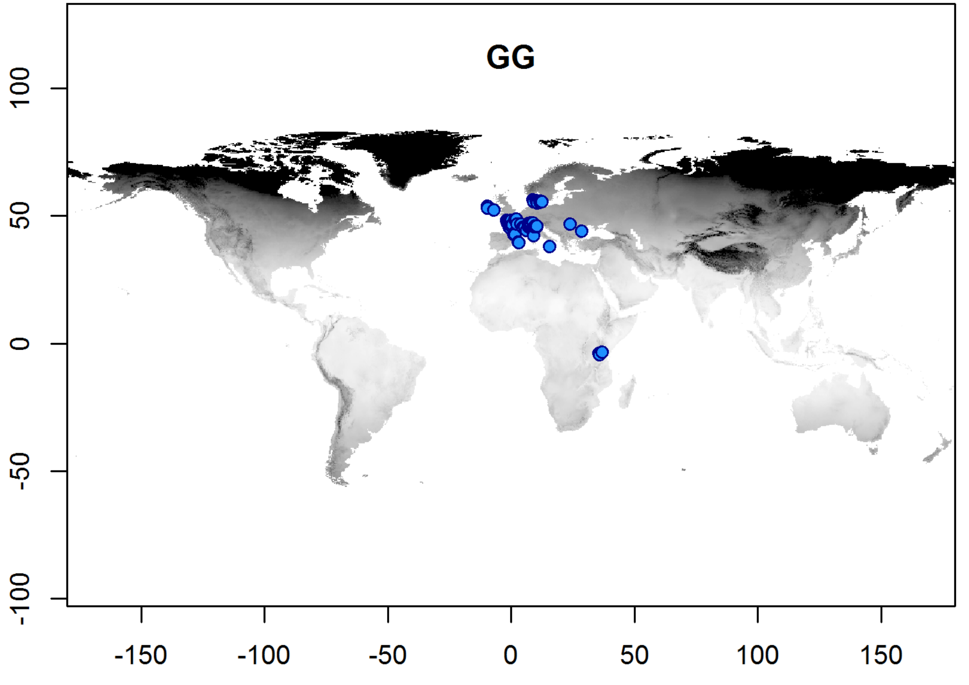

snp6505-scaffold1230-1135091

Chromosome: 21 : 32270925

Best association

Environmental variable = bio1

G score = 622.79

Beta 1 = 1.51

AIC = 1824.08

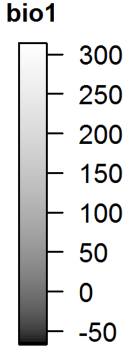

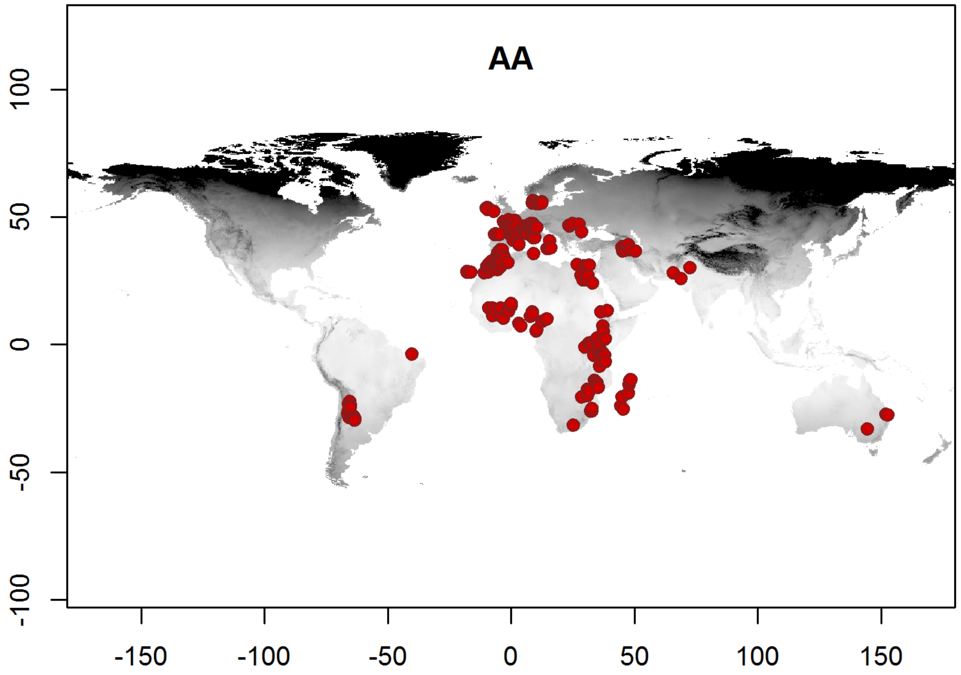

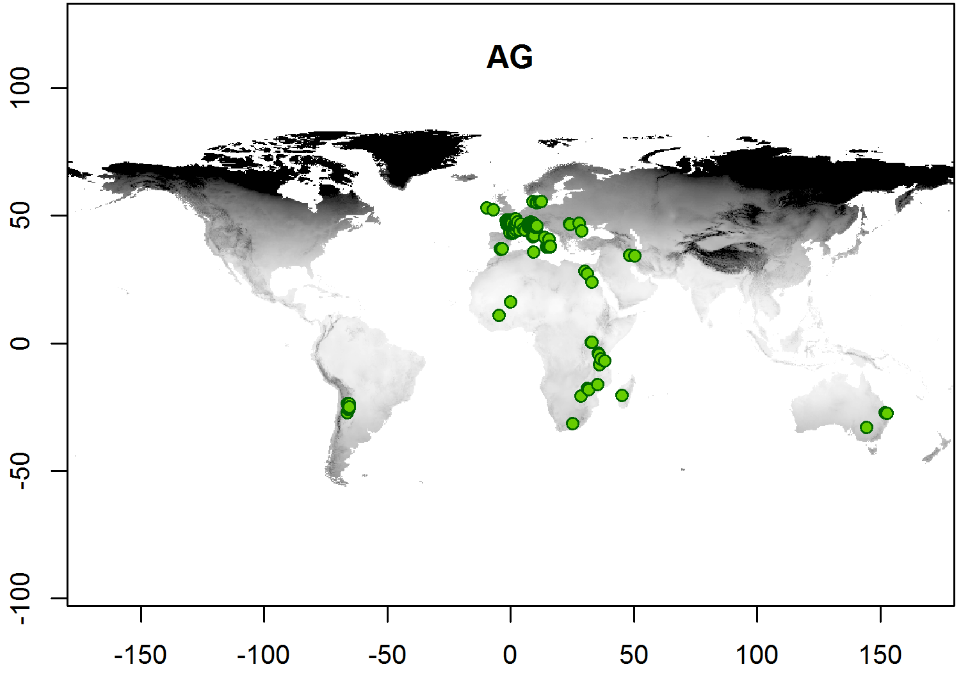

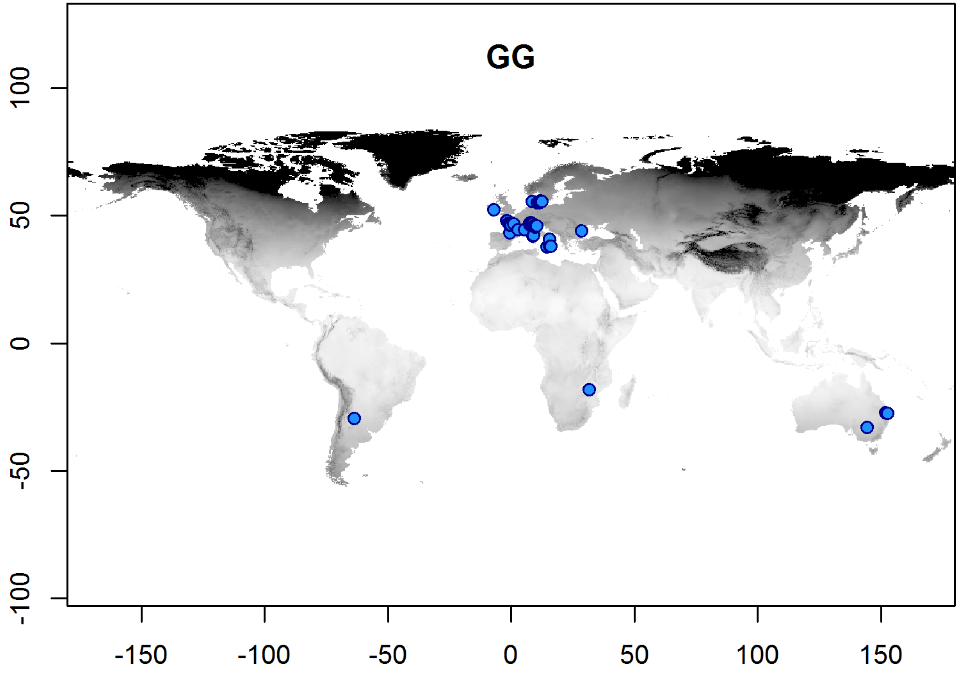

snp6389-scaffold1226-48340

Chromosome: 26 : 134674

Best association

Environmental variable = bio11

G score = 521.62

Beta 1 = 1.61

AIC = 1471.55

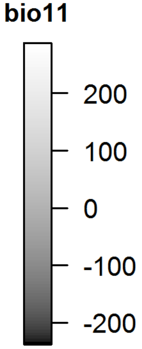

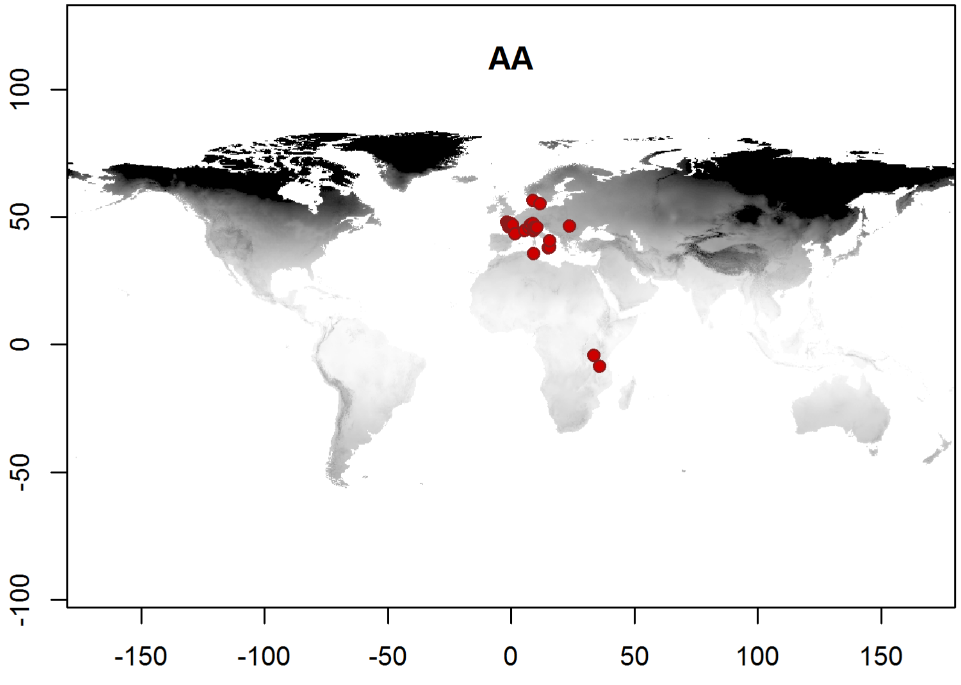

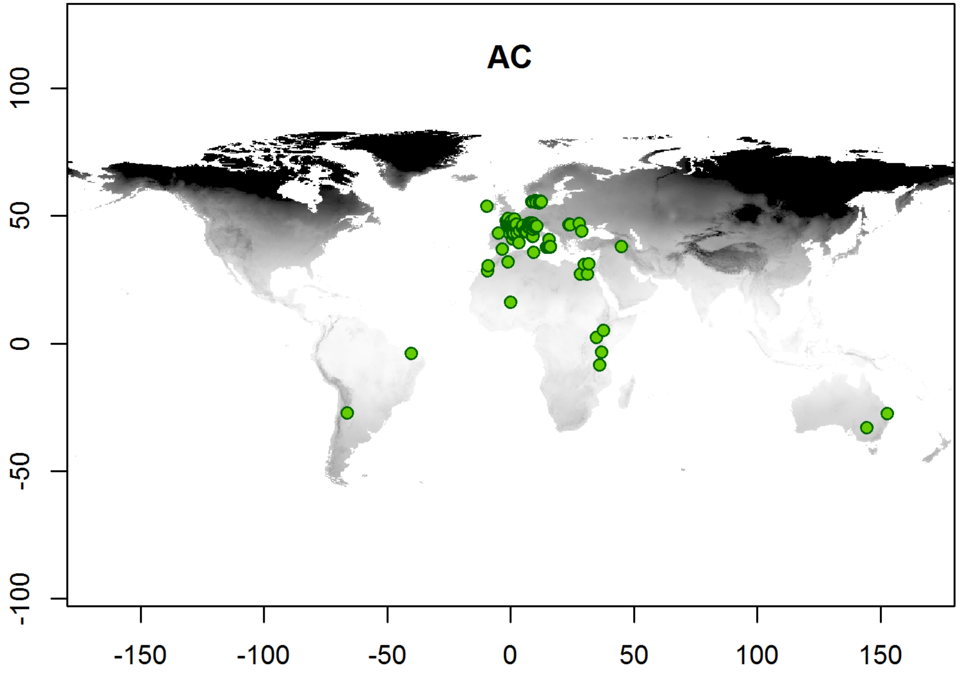

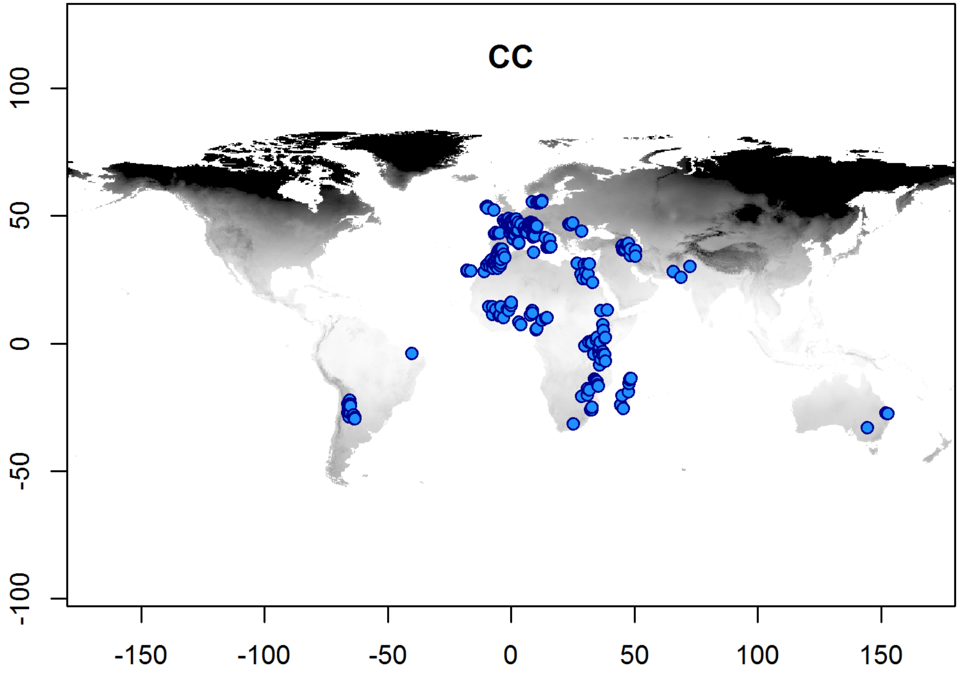

snp6177-scaffold1218-403823

Chromosome: 14 : 63784925

Best association

Environmental variable = bio11

G score = 524.3

Beta 1 = 1.54

AIC = 1569.68

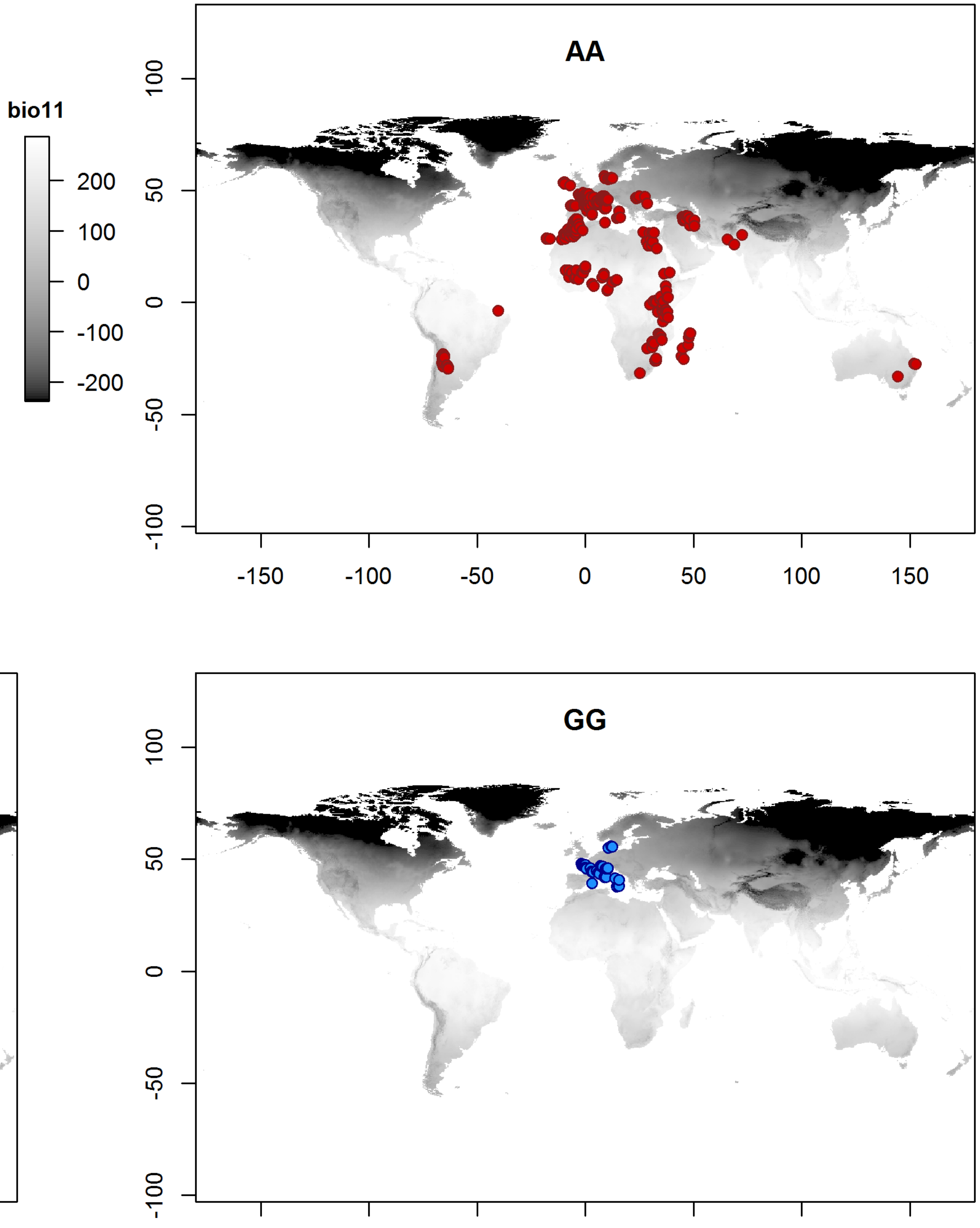

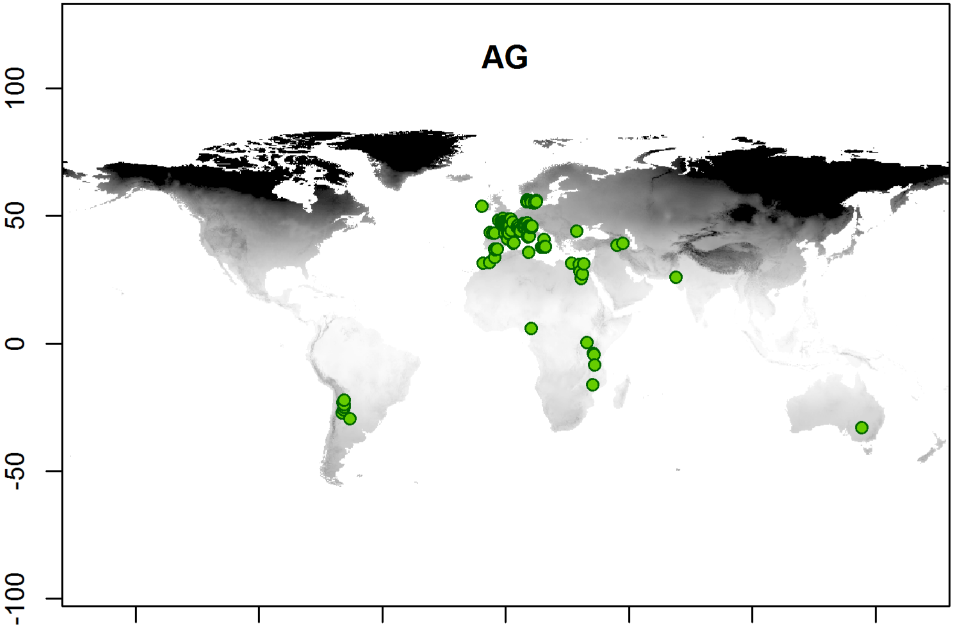

snp6037-scaffold1213-90216

Chromosome: 5 : 5722249

Best association

Environmental variable = bio1

G score = 870.56

Beta 1 = 1.93

AIC = 1626.24

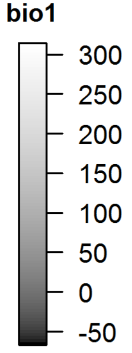

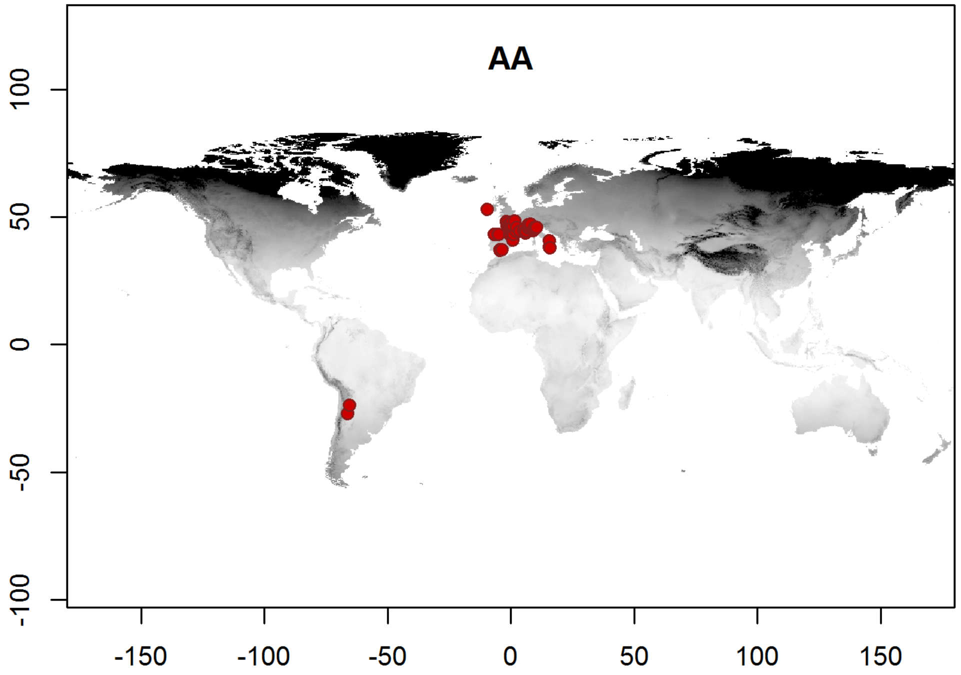

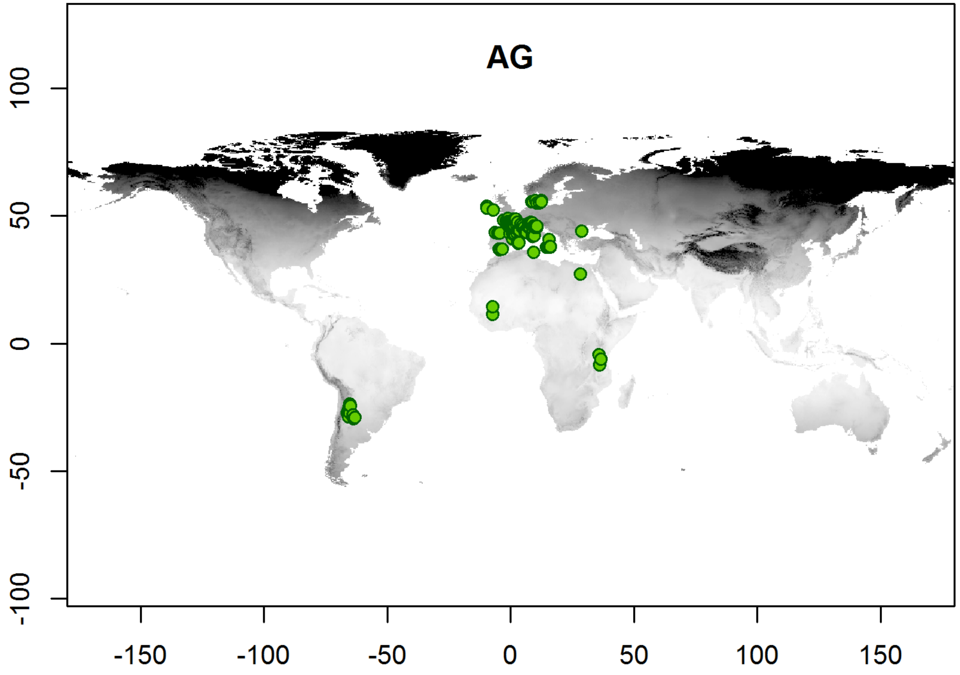

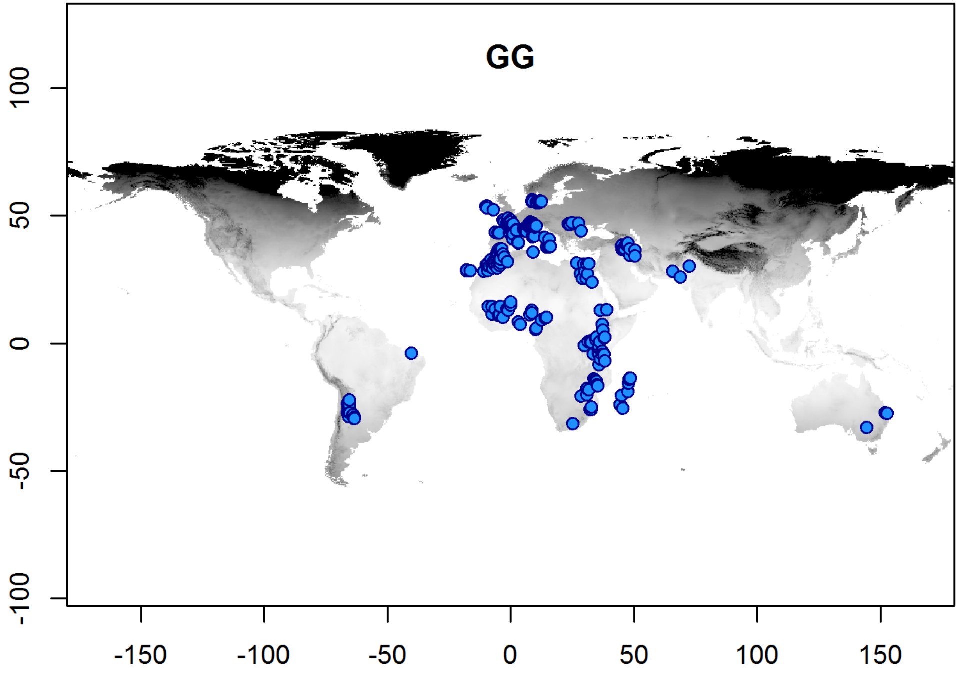

snp4913-scaffold1164-1343187

Chromosome: 18 : 1357647

Best association

Environmental variable = bio1

G score = 637.12

Beta 1 = 1.48

AIC = 1897.77

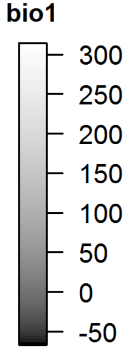

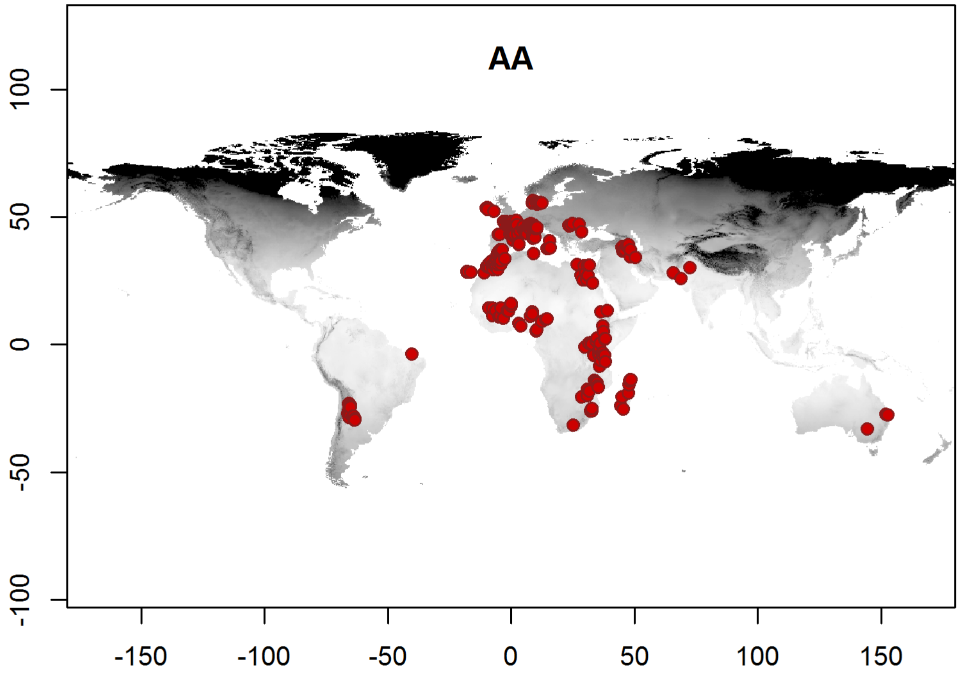

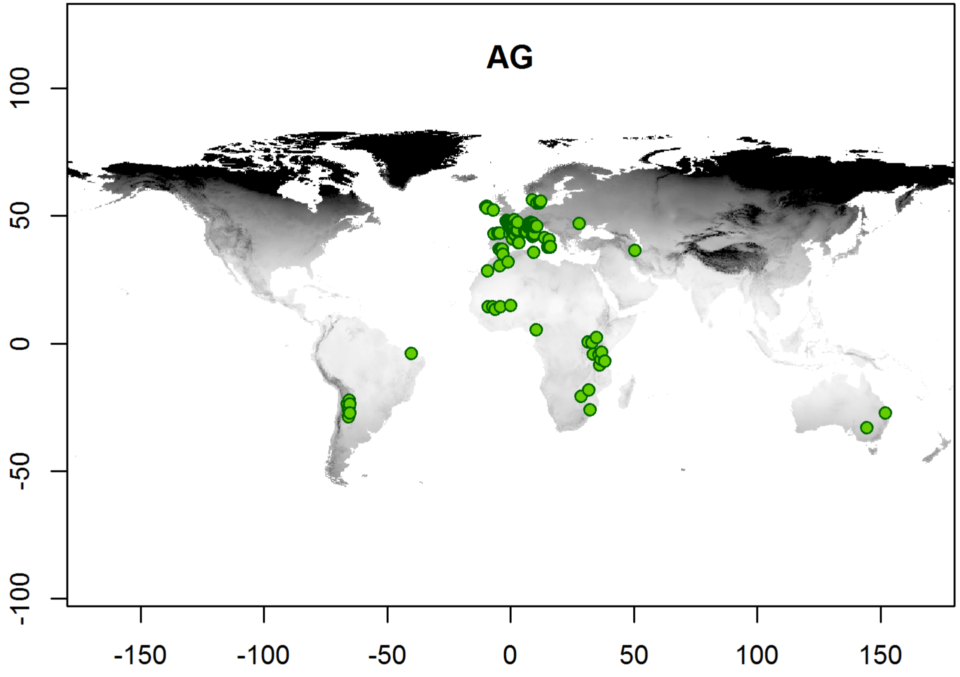

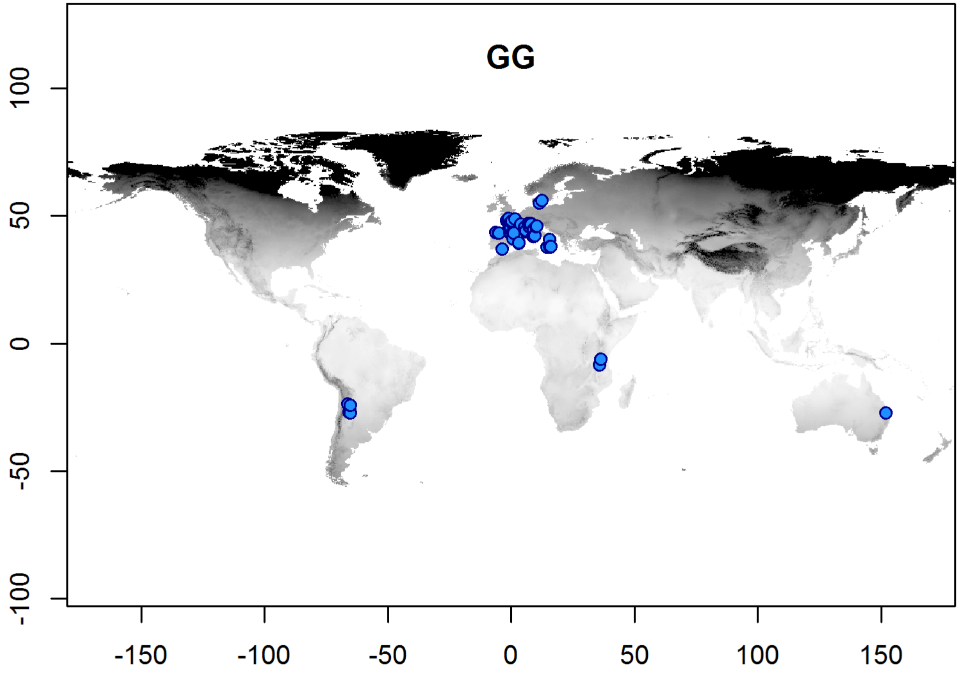

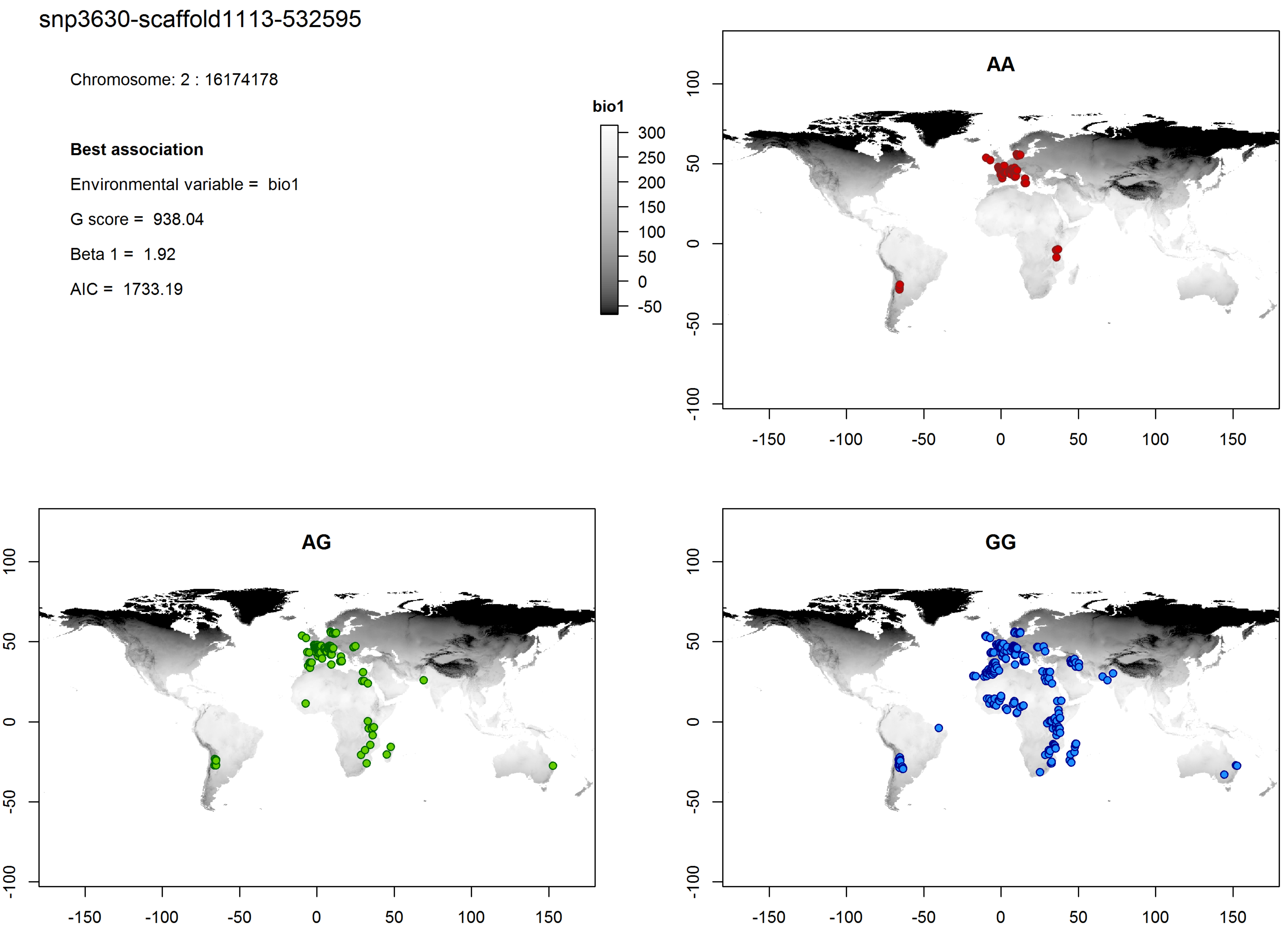
snp3630-scaffold1113-532595
Chromosome: 2 : 16174178
Best association
Environmental variable = bio1
G score = 938.04
Beta 1 = 1.92
AIC = 1733.19
bio1
300
250
200
150
100
50
0
-50
AA
AG
GG
100
50
0
-50
-100
-150
-100
-50
0
50
100
150

snp3602-scaffold1111-963076

Chromosome: 11 : 49353940

Best association

Environmental variable = bio1

G score = 640.32

Beta 1 = 1.59

AIC = 1727.12

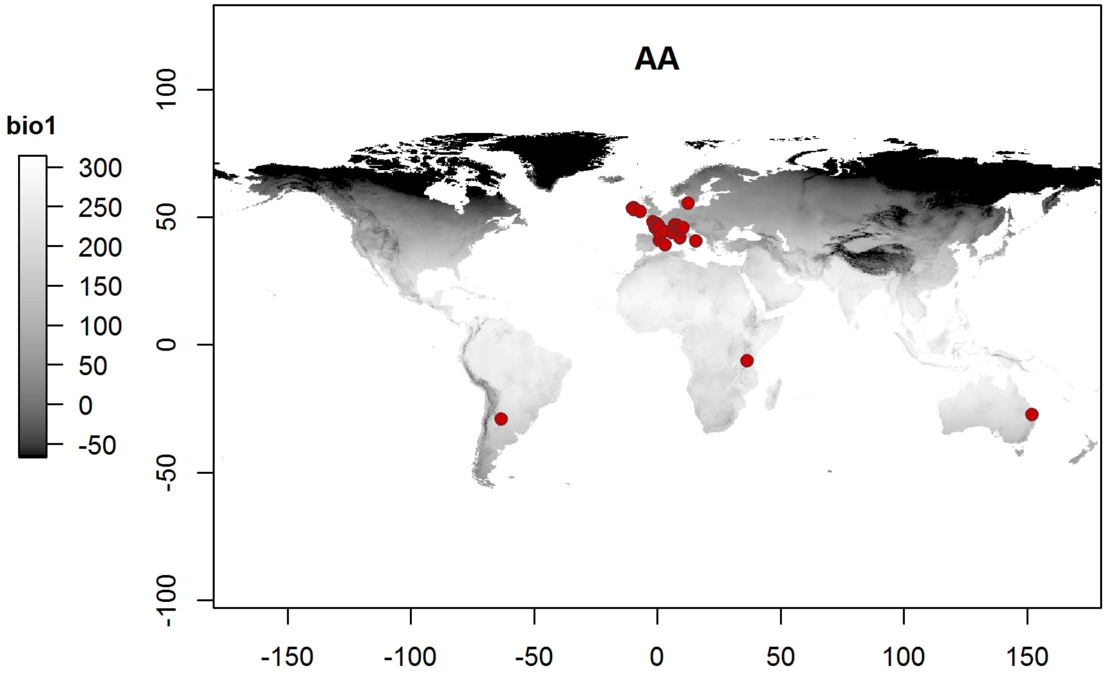

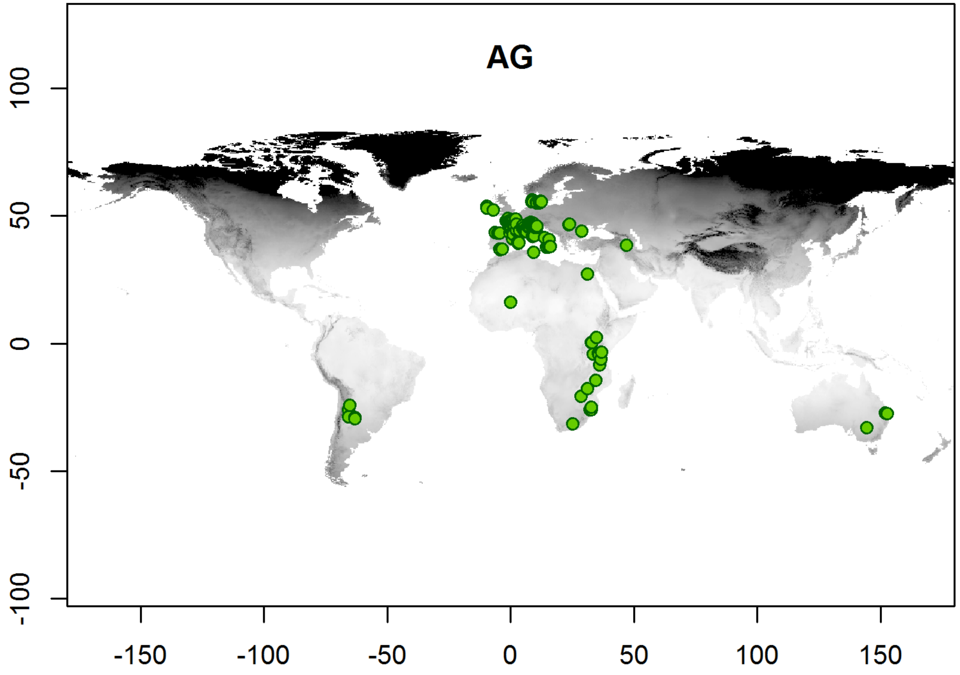

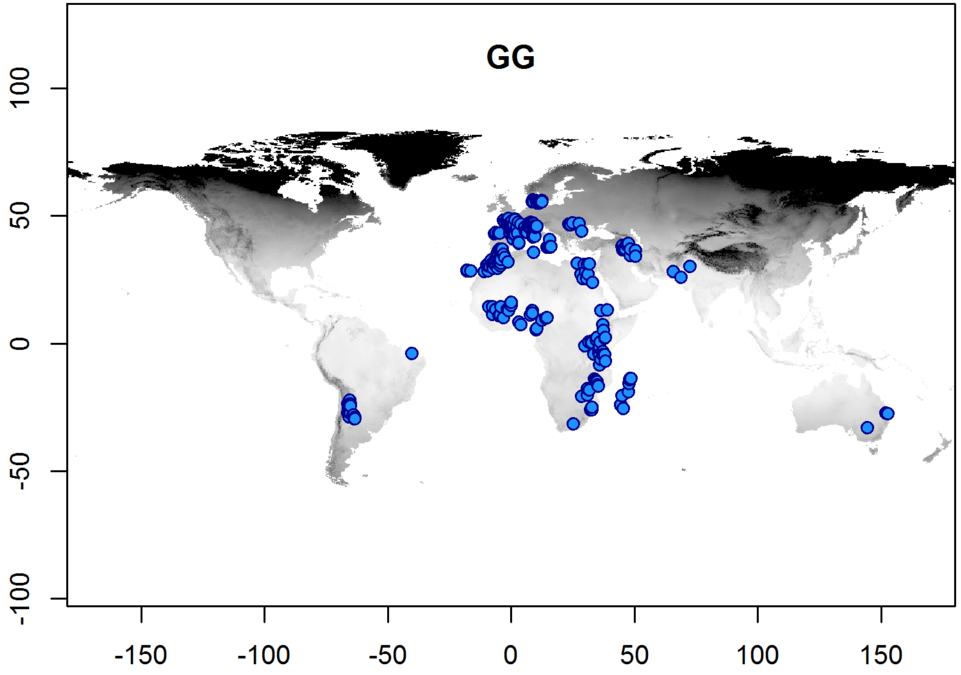

snp1858-scaffold1052-1225291

Chromosome: 14 : 24622151

Best association

Environmental variable = bio1

G score = 584.16

Beta 1 = 1.51

AIC = 1734.12

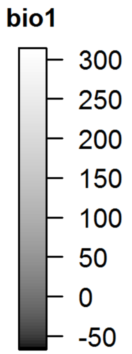

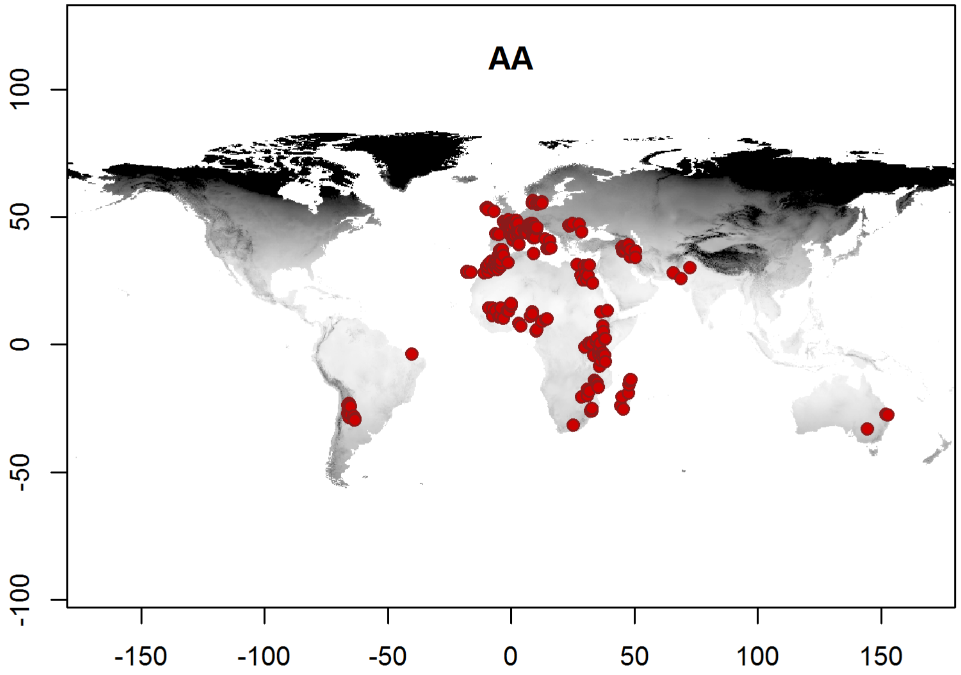

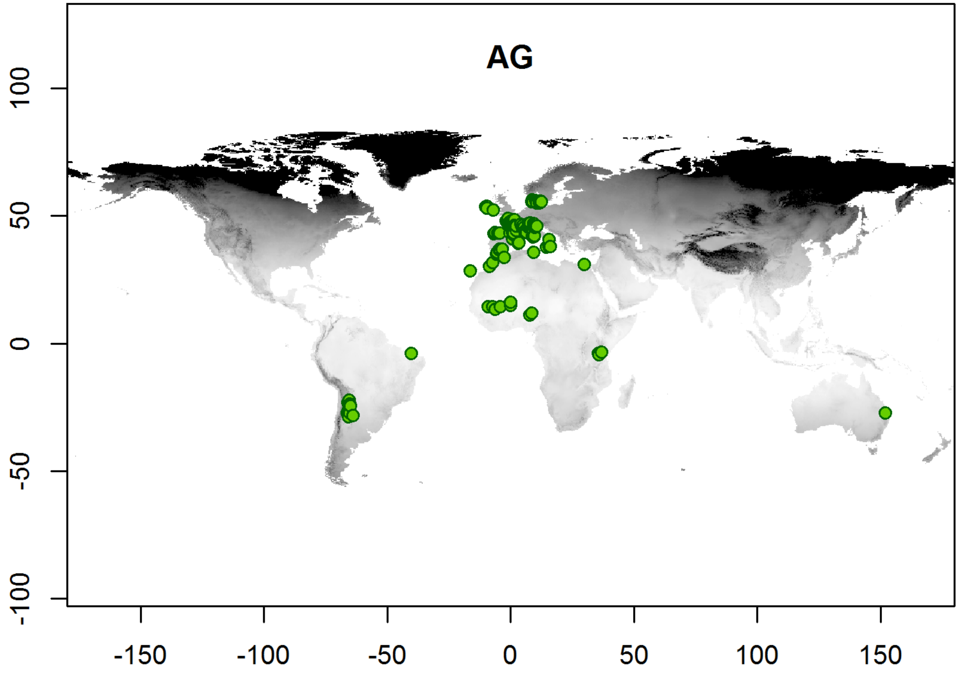

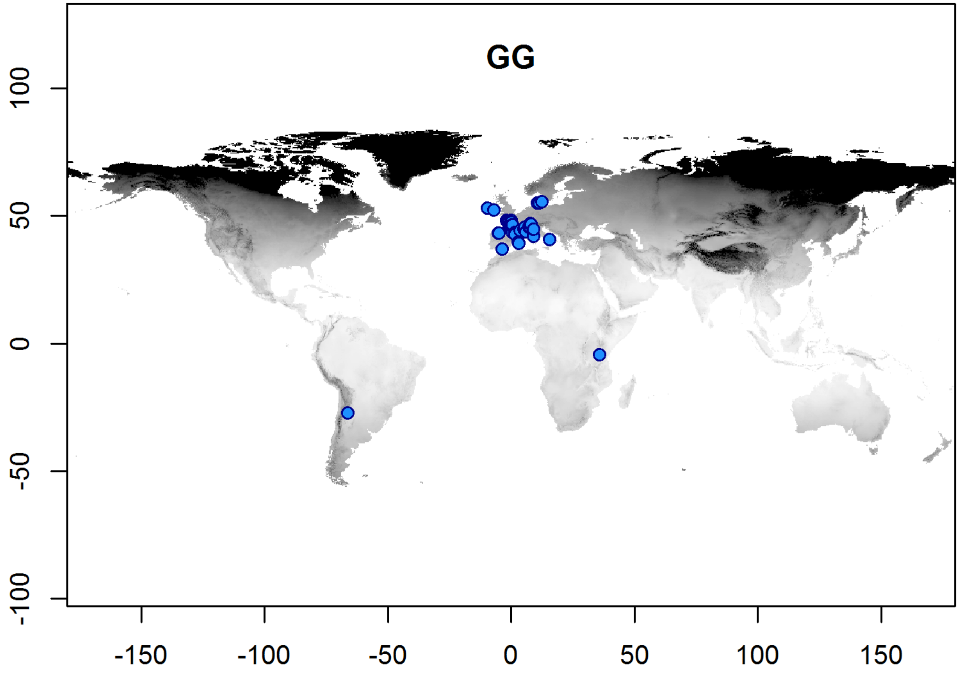

snp1640-scaffold10469-208327

Chromosome: 13 : 53160922

Best association

Environmental variable = bio1

G score = 784.03

Beta 1 = 1.73

AIC = 1762.86

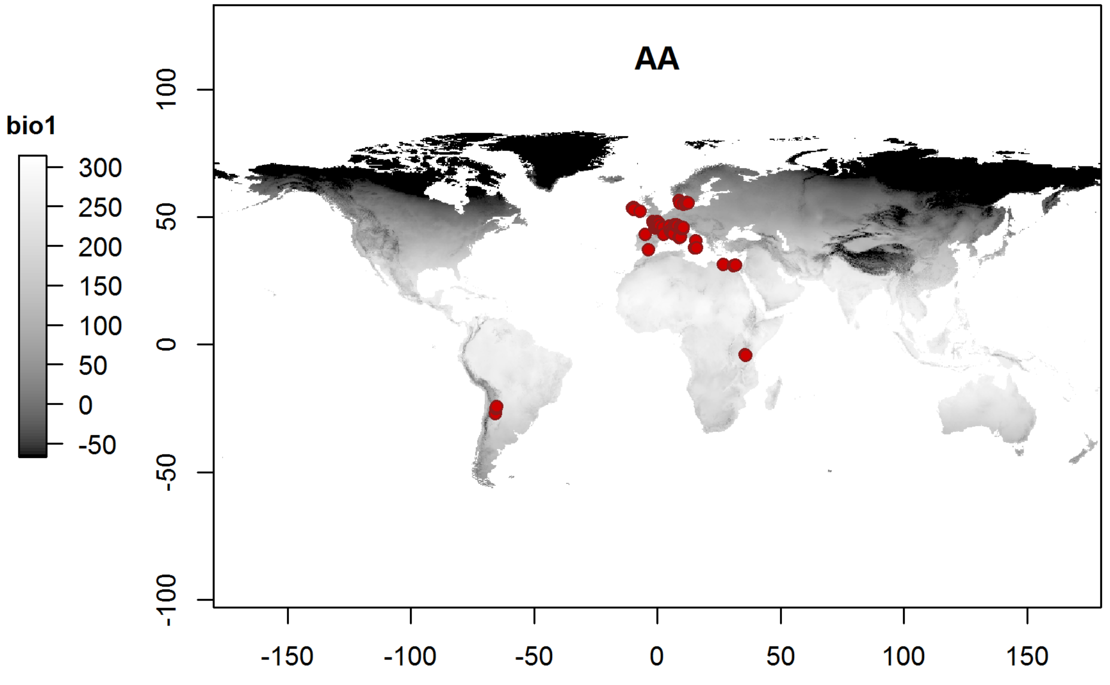

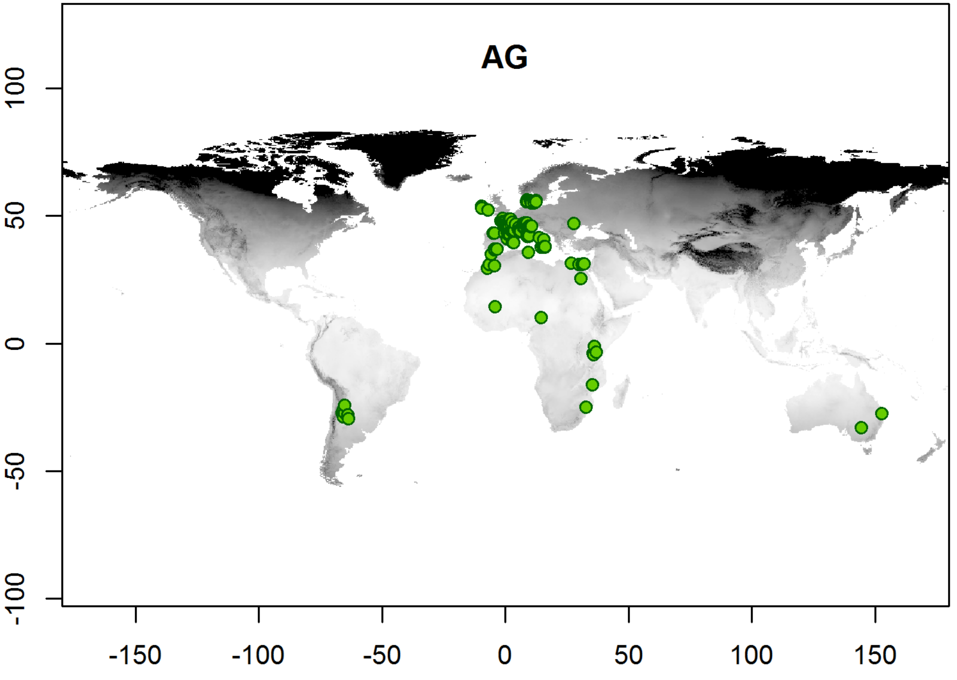

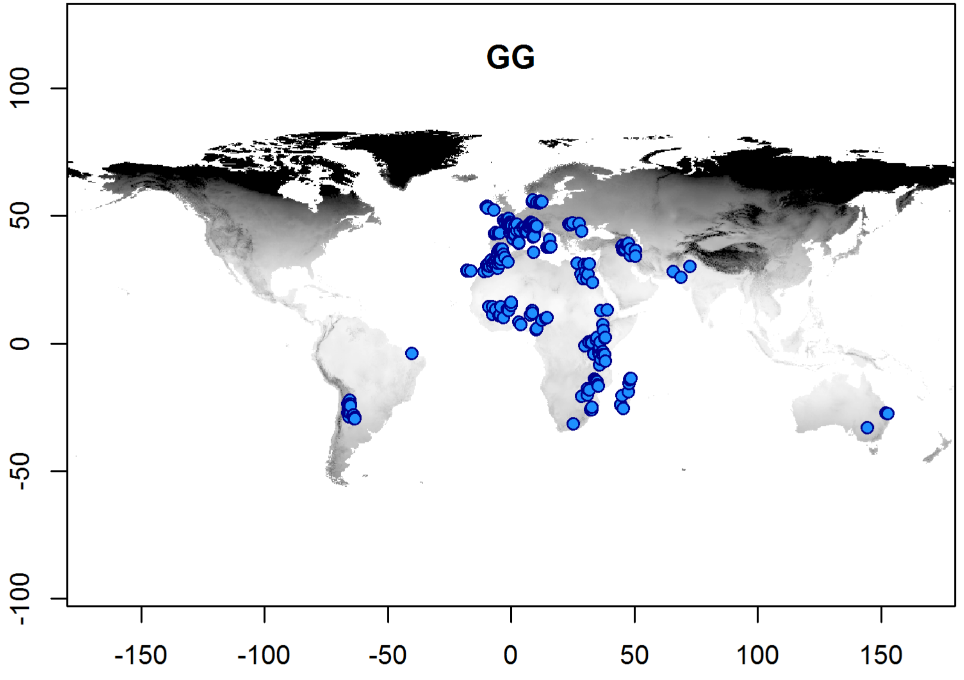

snp1630-scaffold1046-1400739

Chromosome: 19 : 14819207

Best association

Environmental variable = bio1

G score = 605.17

Beta 1 = 1.52

AIC = 1761.91

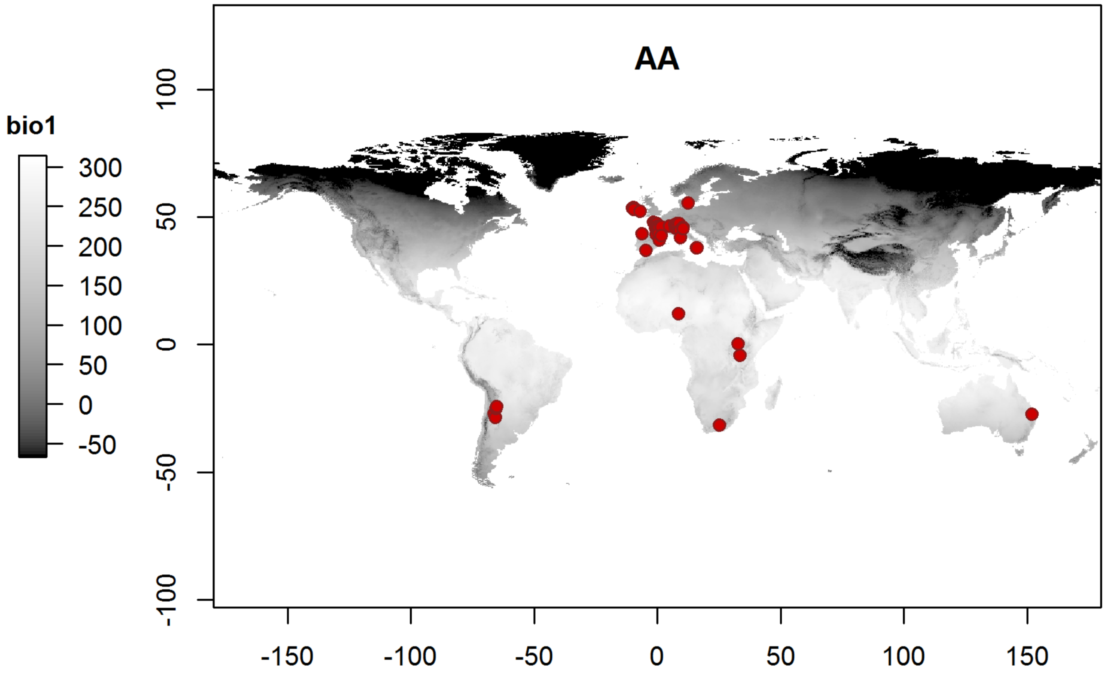

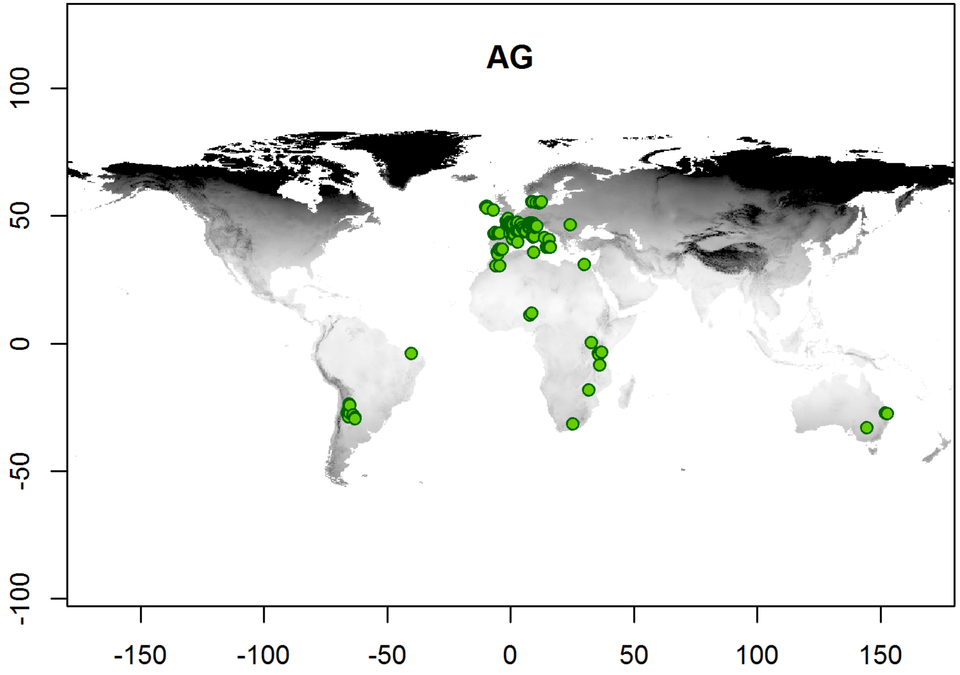

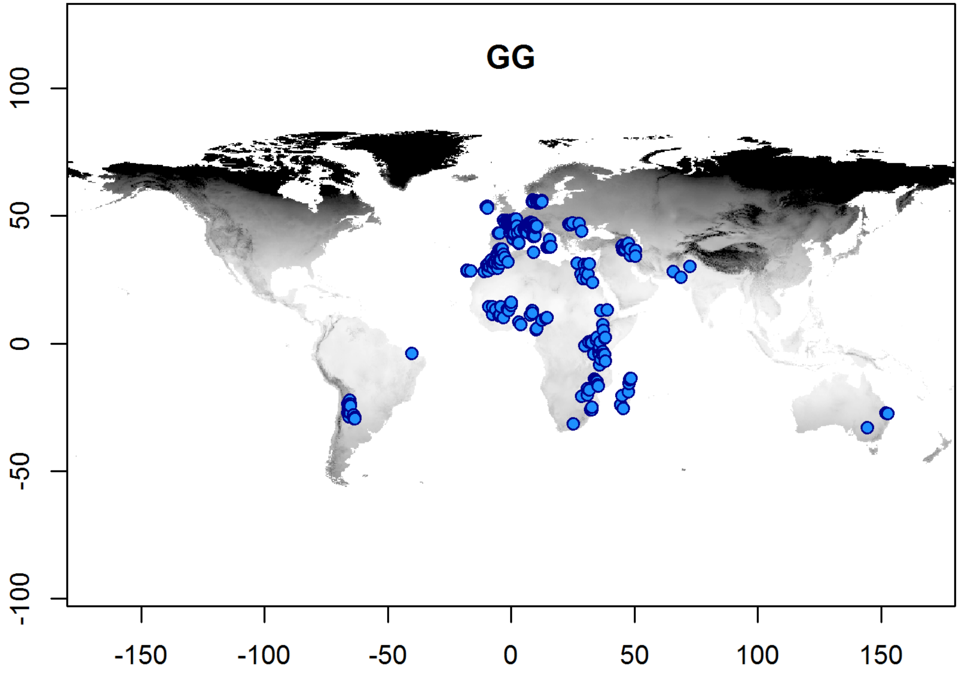

snp88-scaffold100-417512

Chromosome: 15 : 75951851

Best association

Environmental variable = bio1

G score = 495.54

Beta 1 = 1.4

AIC = 1729.33

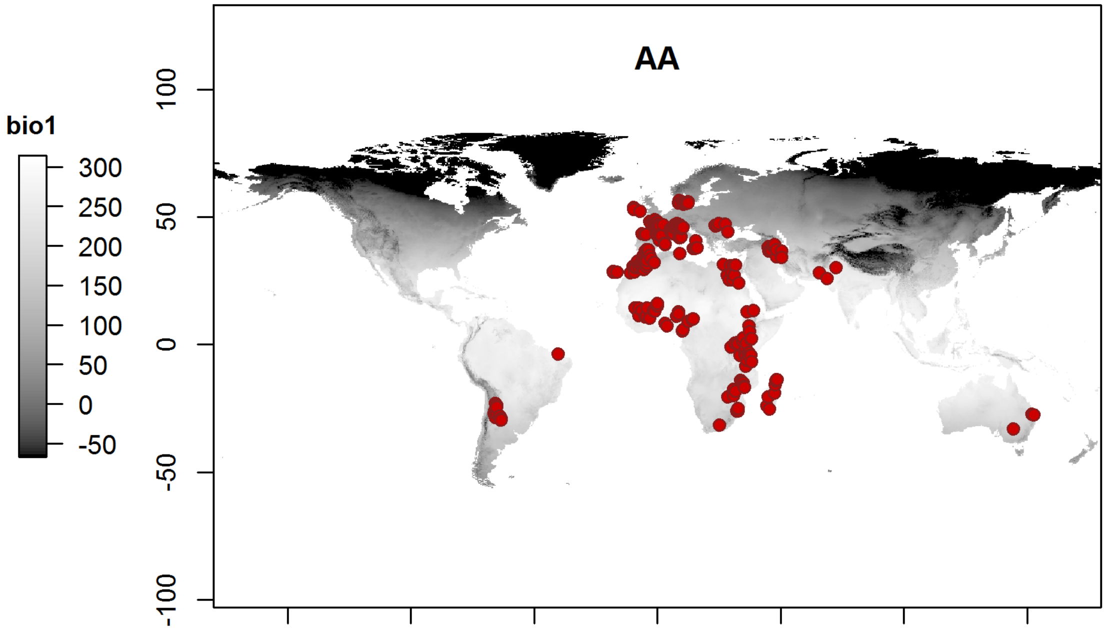

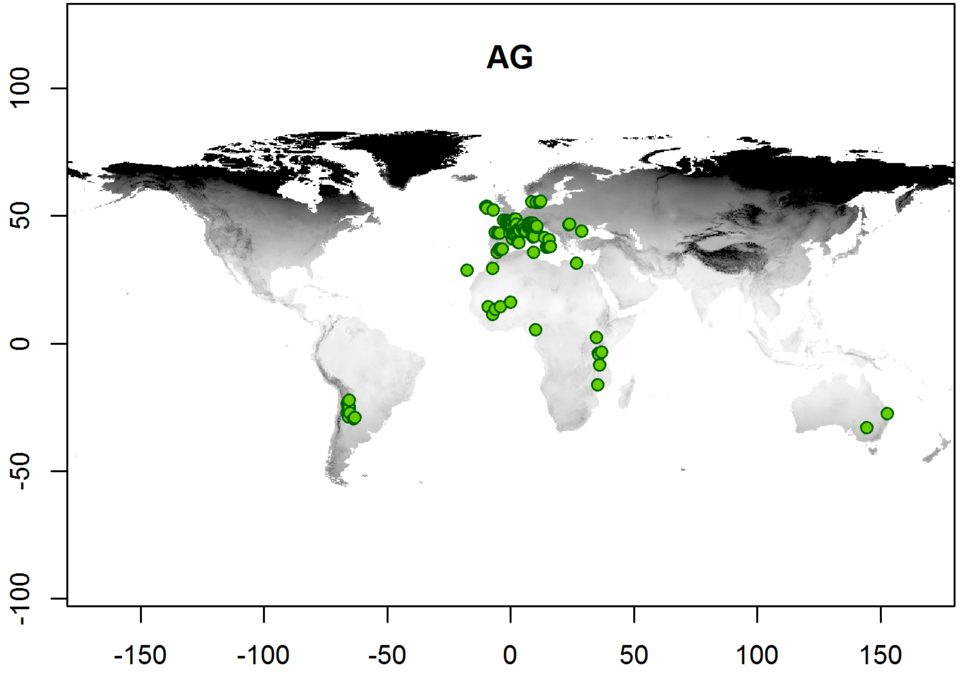

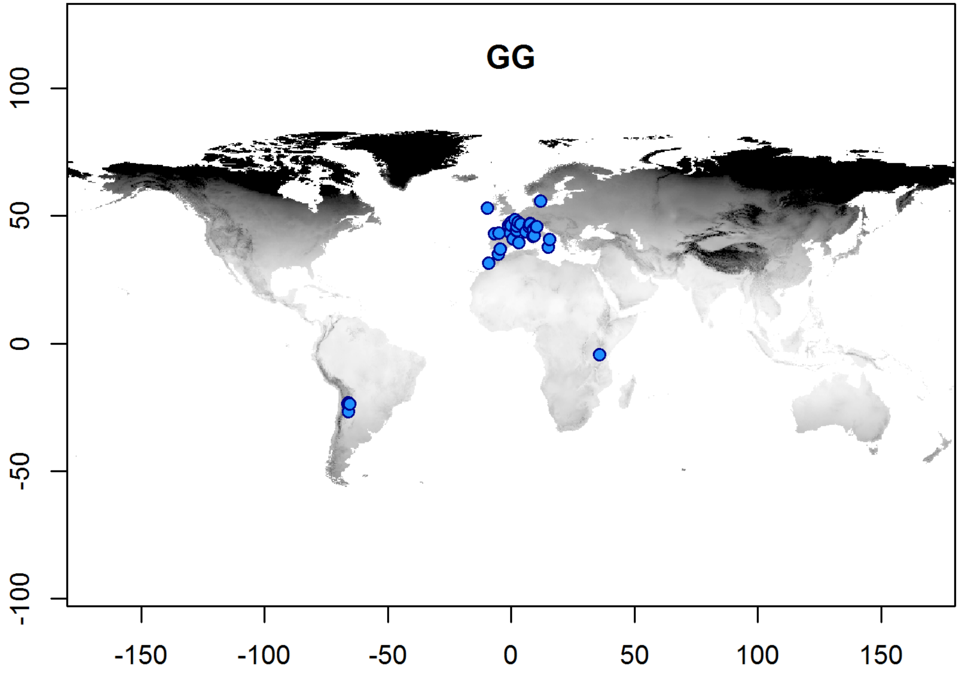

snp29393-scaffold318-794069

Chromosome: 9 : 53453711

Best association

Environmental variable = bio1

G score = 490.82

Beta 1 = 1.36

AIC = 1782.37

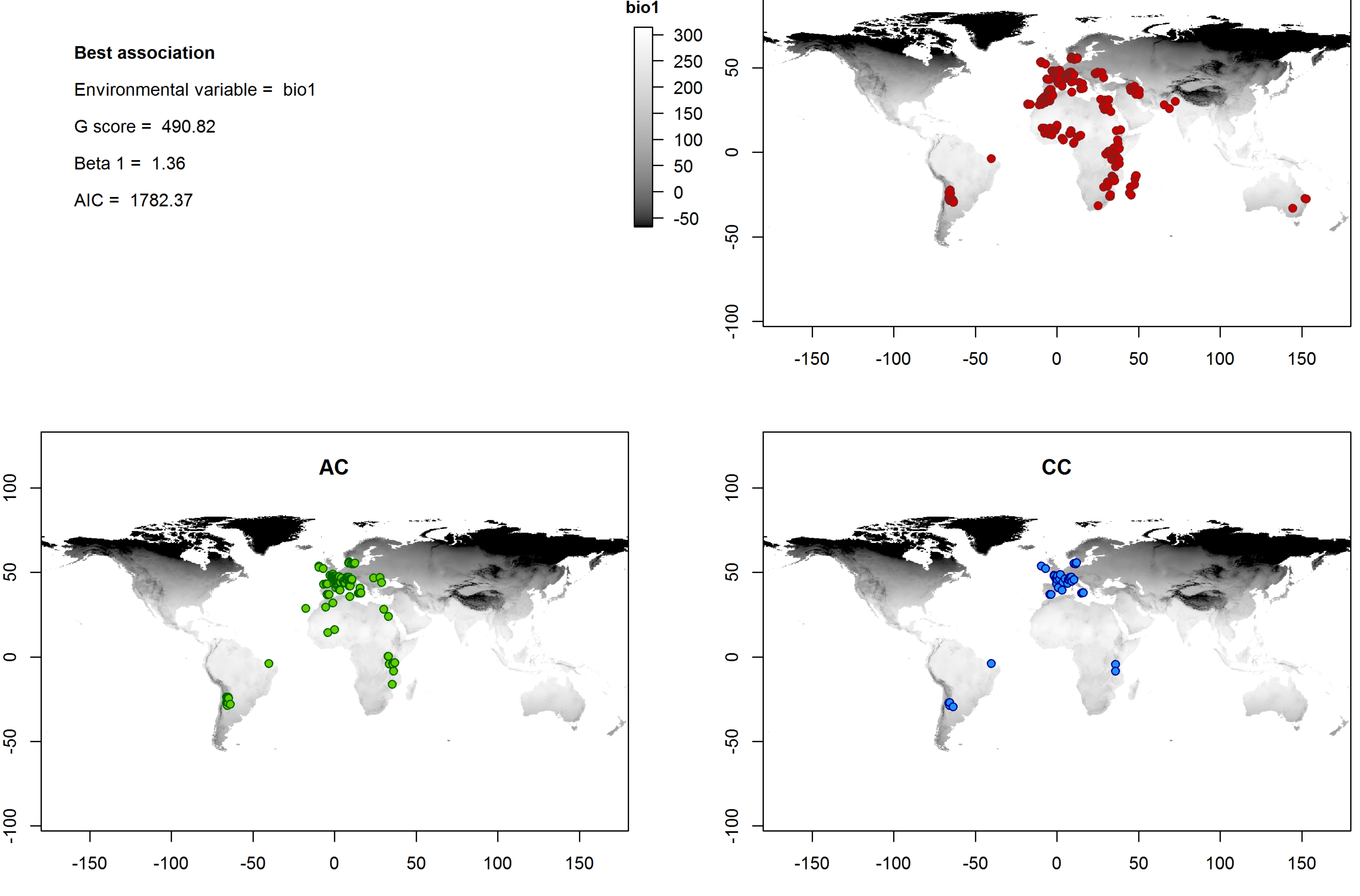

snp28462-scaffold303-4402023

Chromosome: 19 : 34412900

Best association

Environmental variable = bio1

G score = 905.26

Beta 1 = 1.74

AIC = 1935.29

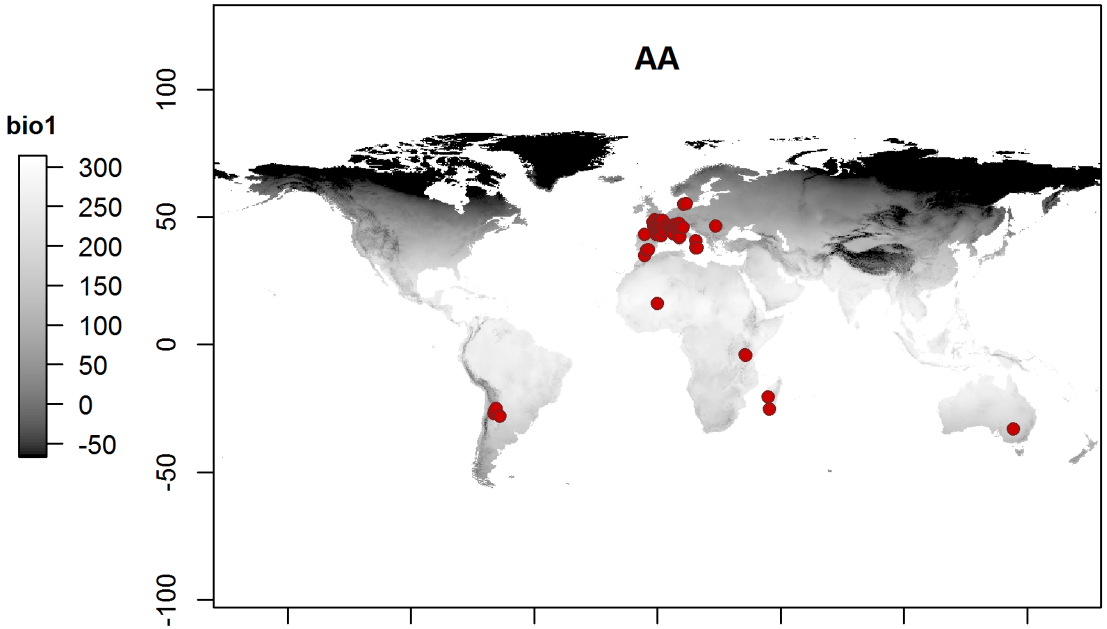

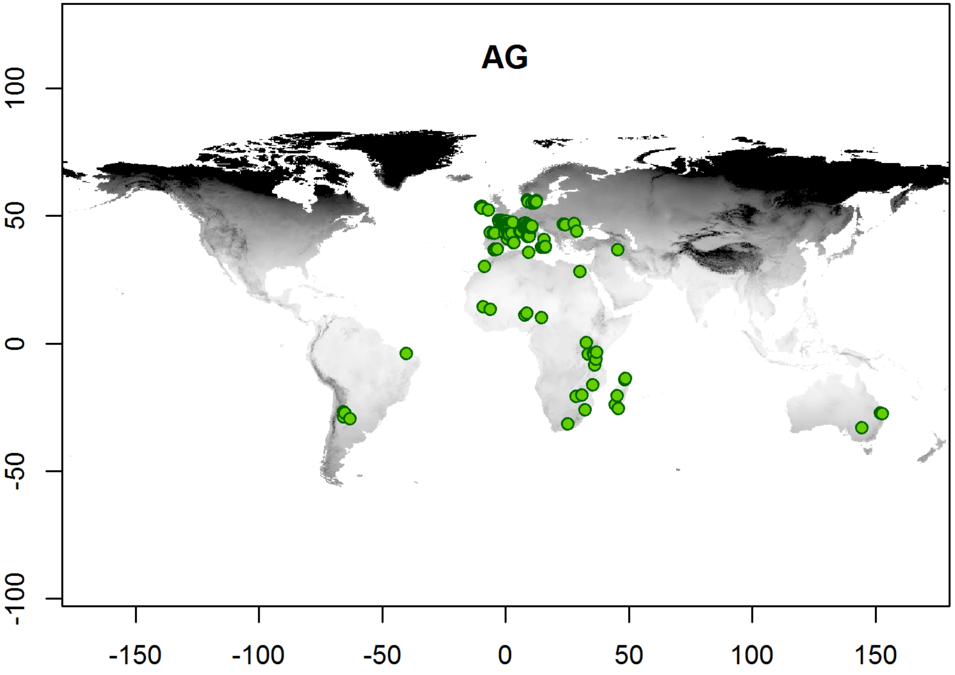

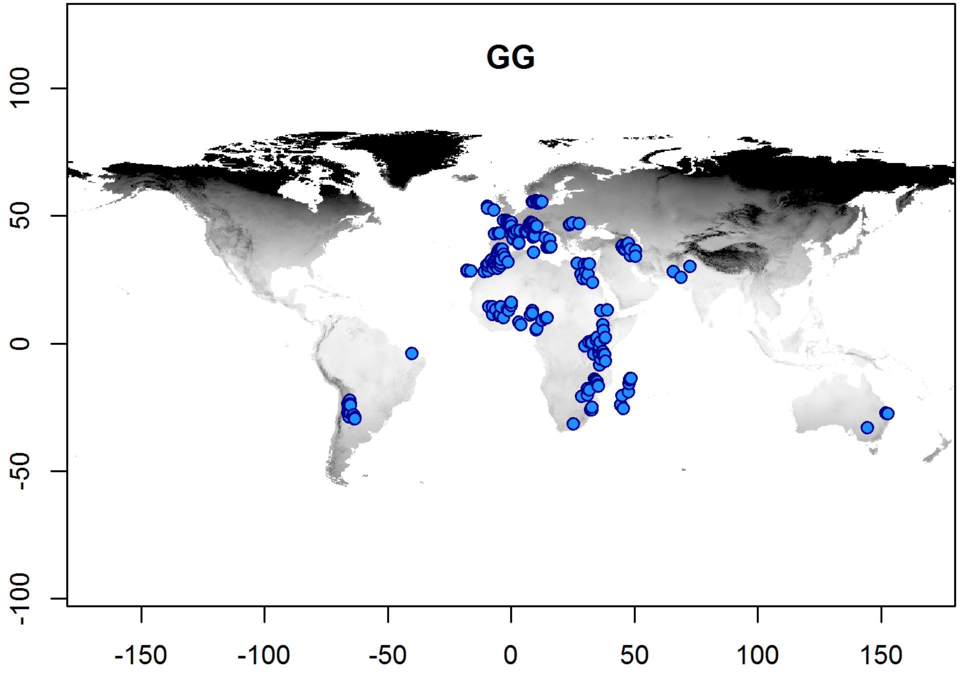

snp27993-scaffold30-2392105

Chromosome: 6 : 81955680

Best association

Environmental variable = bio1

G score = 936.5

Beta 1 = 1.89

AIC = 1752.56

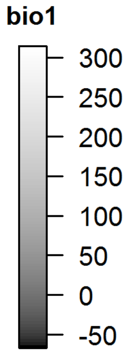

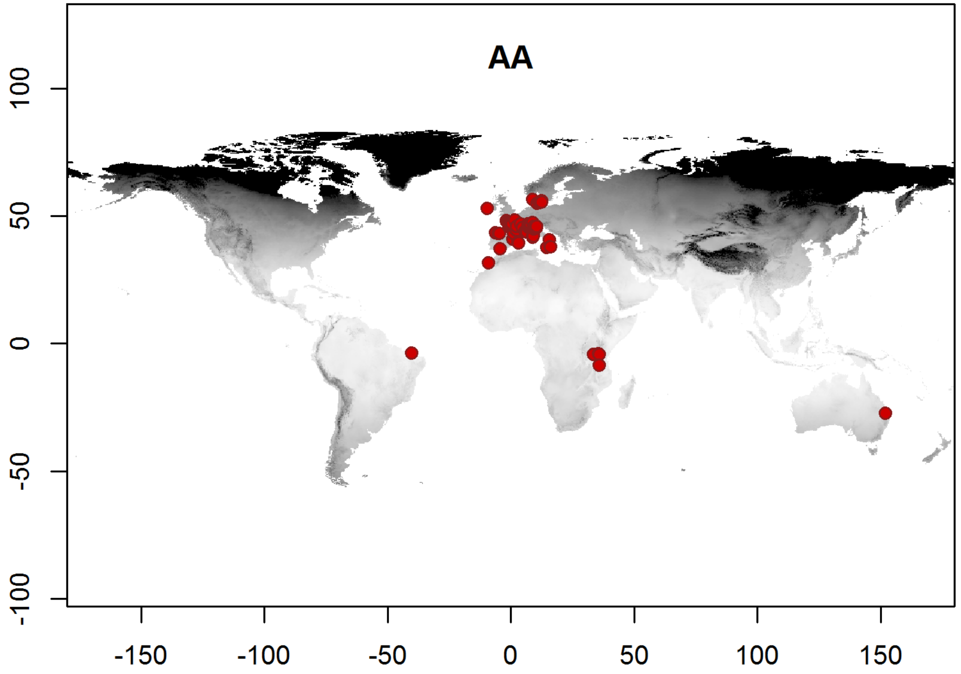

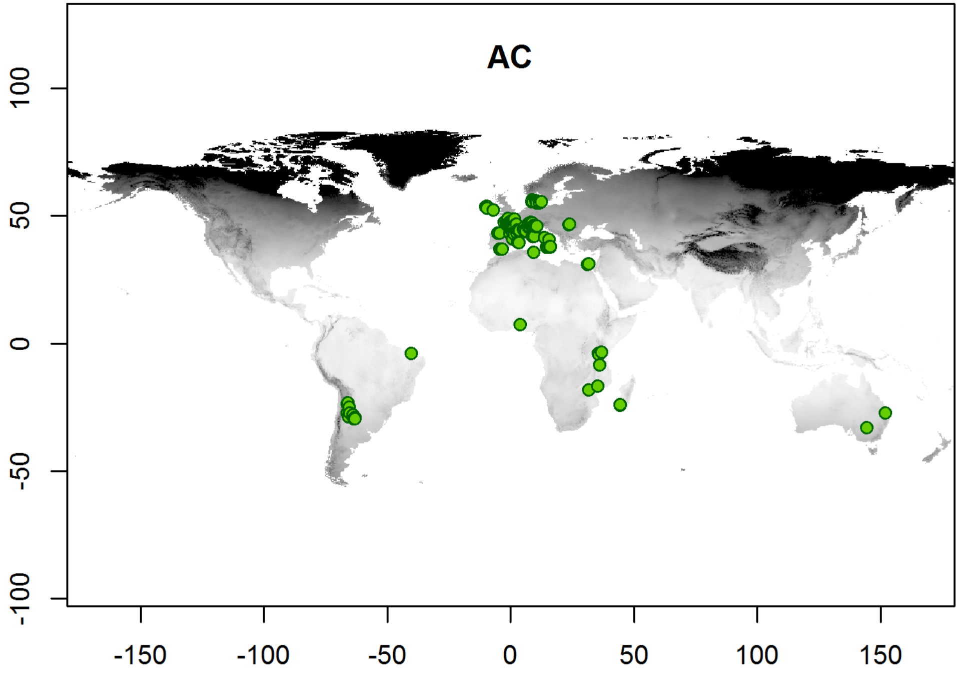

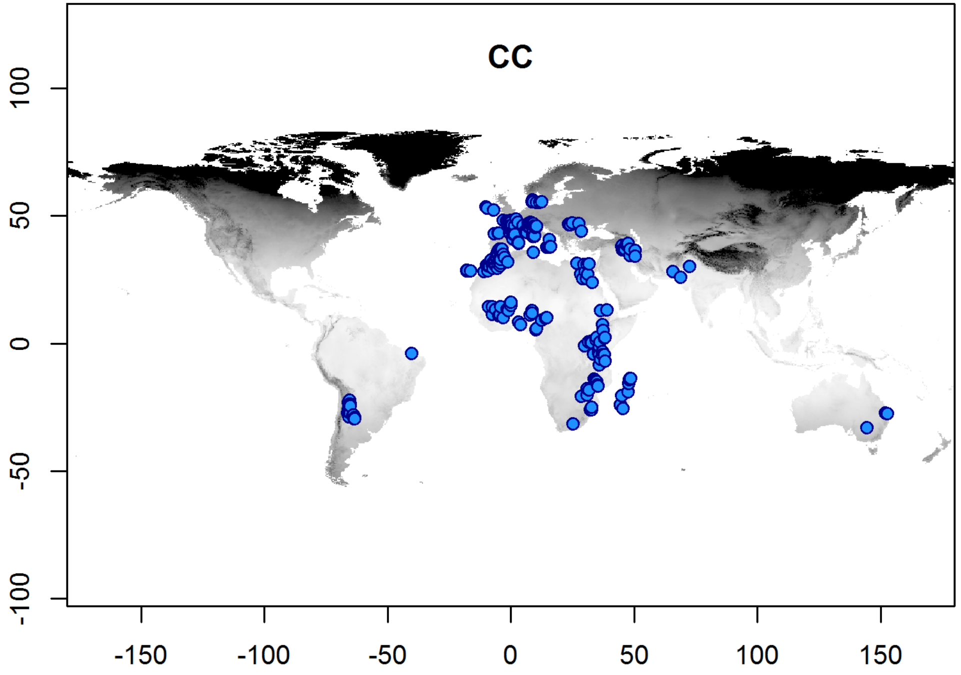

snp27730-scaffold296-191224

Chromosome: 24 : 12176434

Best association

Environmental variable = bio1

G score = 665.36

Beta 1 = 1.52

AIC = 1882.67

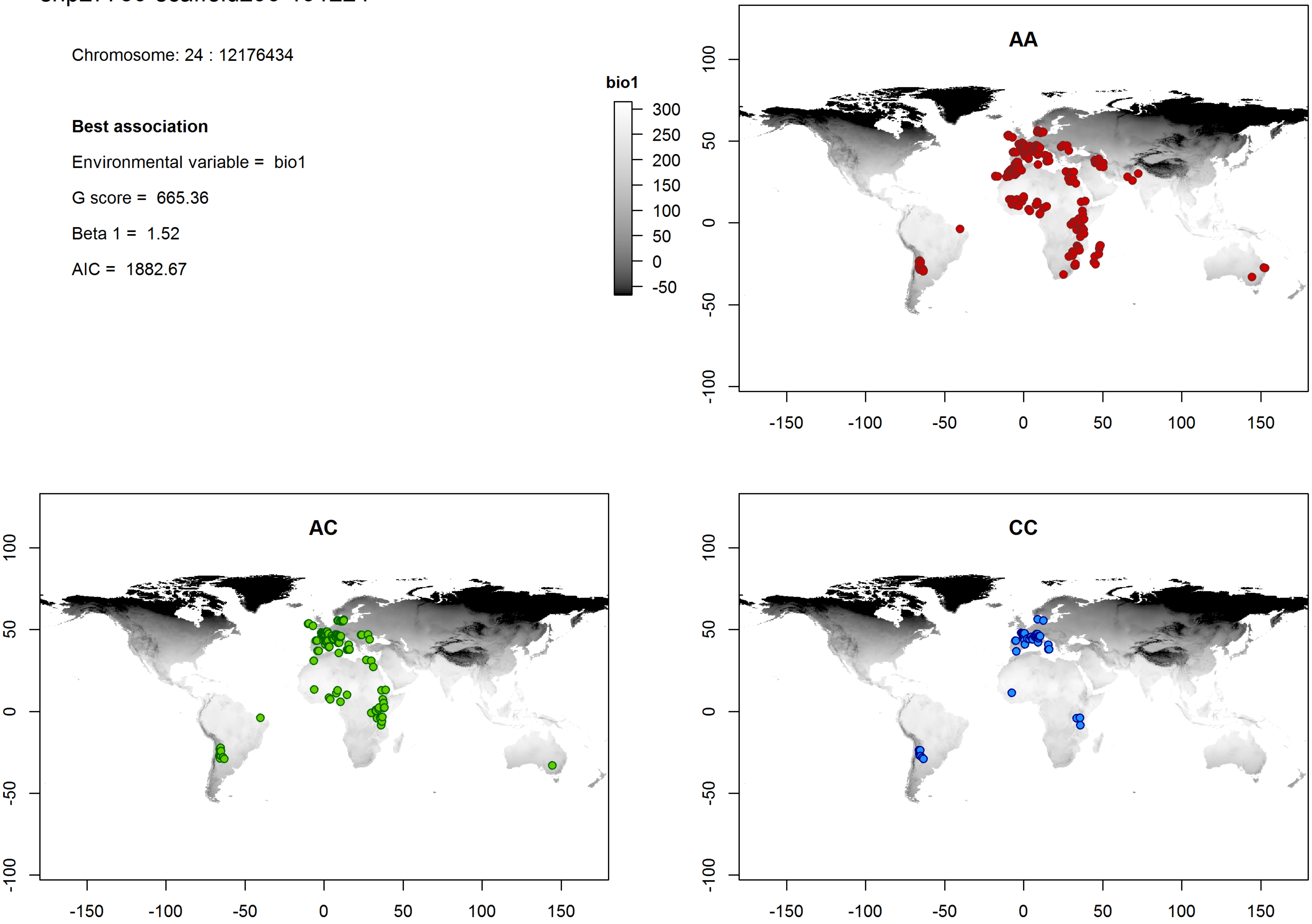

snp26989-scaffold287-727057

Chromosome: 2 : 17843753

Best association

Environmental variable = bio1

G score = 597.63

Beta 1 = 1.82

AIC = 1373.25

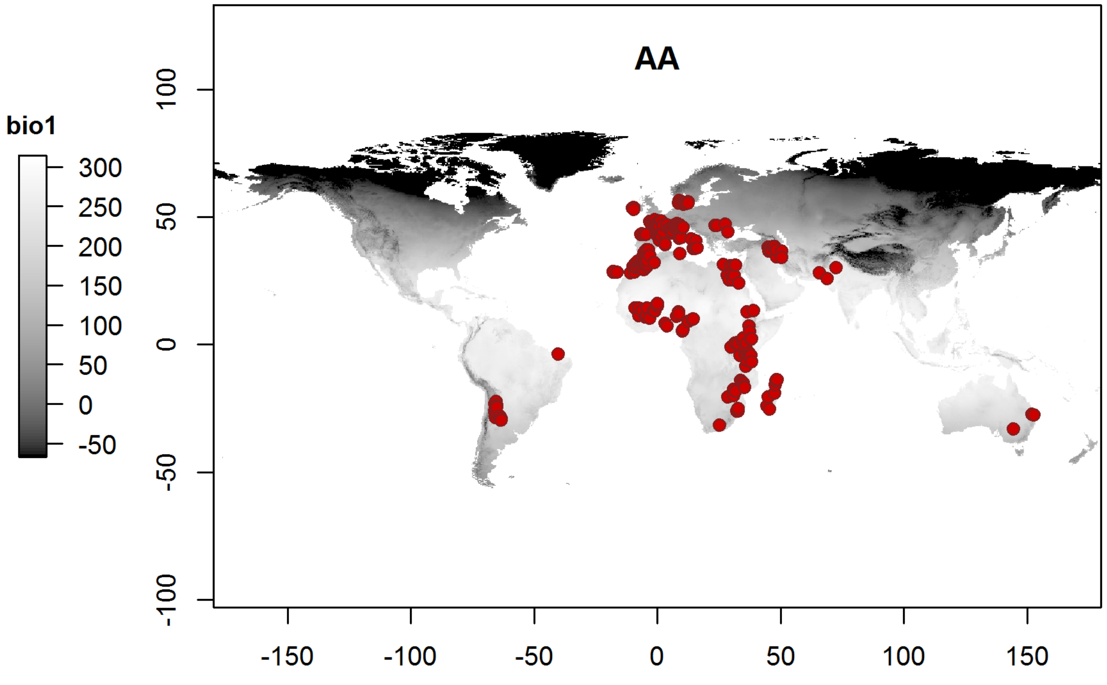

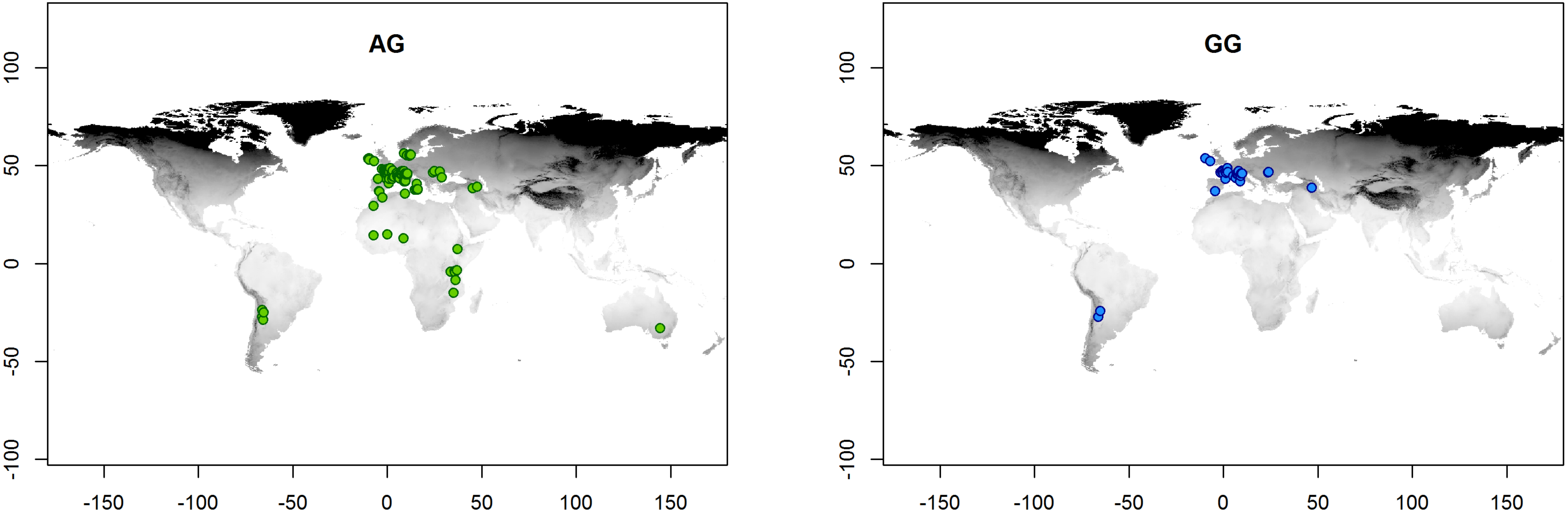

snp26988-scaffold287-681145

Chromosome: 2 : 17797516

Best association

Environmental variable = bio1

G score = 716.07

Beta 1 = 1.84

AIC = 1529.79

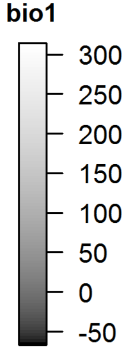

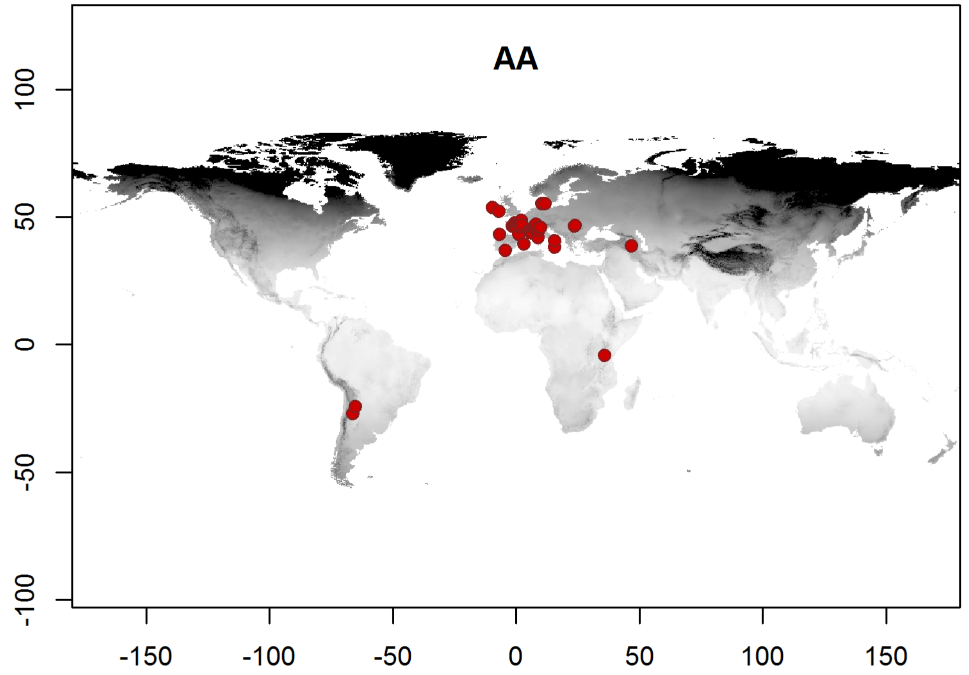

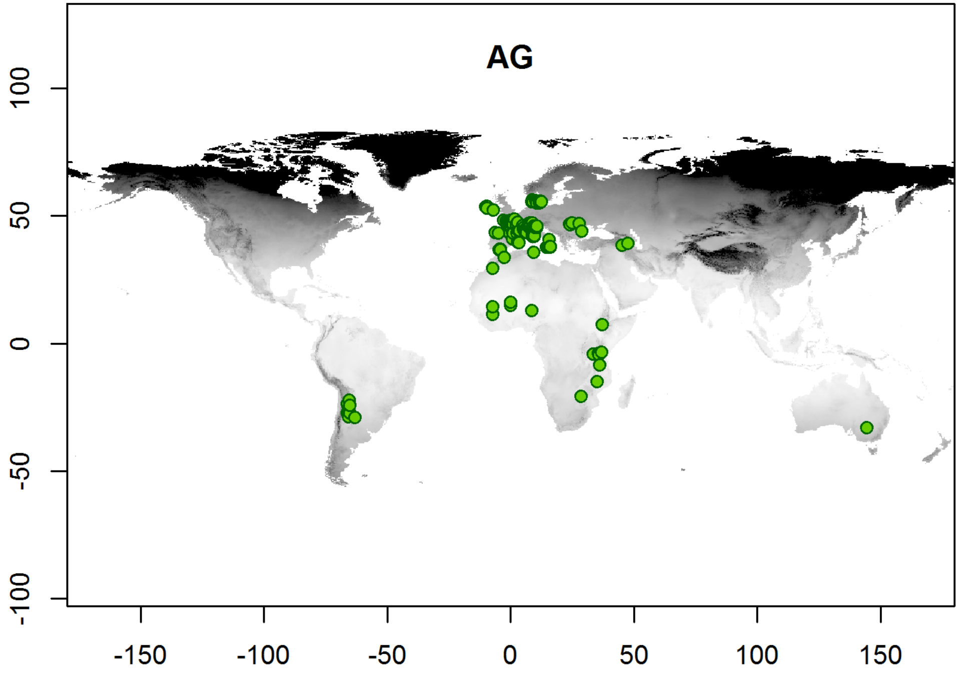

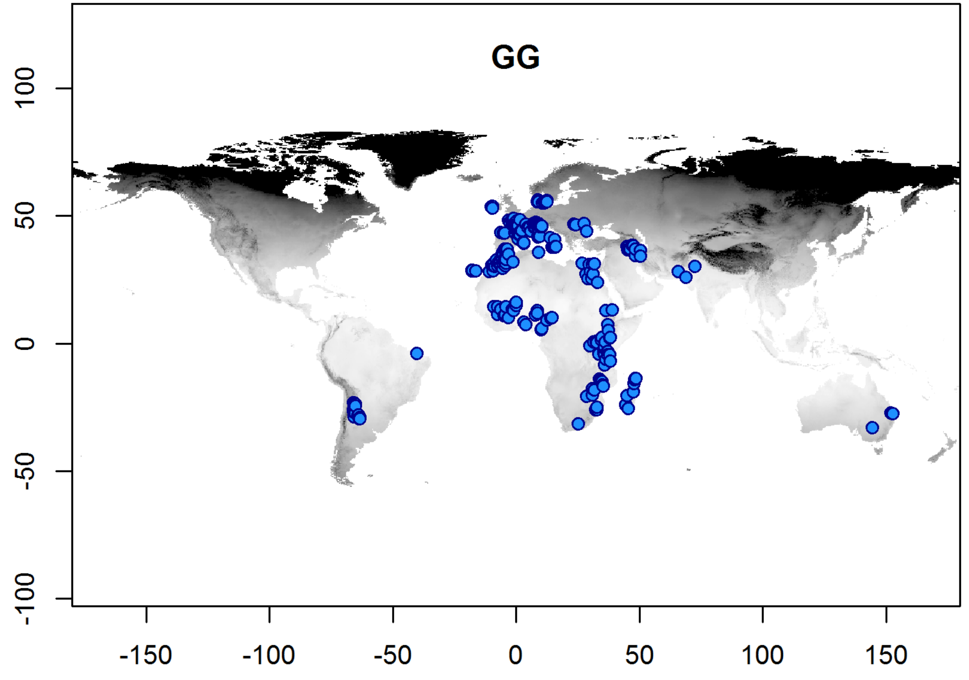

snp26537-scaffold2765-185379

Chromosome: 29 : 50448626

Best association

Environmental variable = bio1

G score = 881

Beta 1 = 1.76

AIC = 1867.44

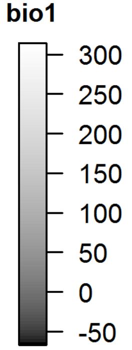

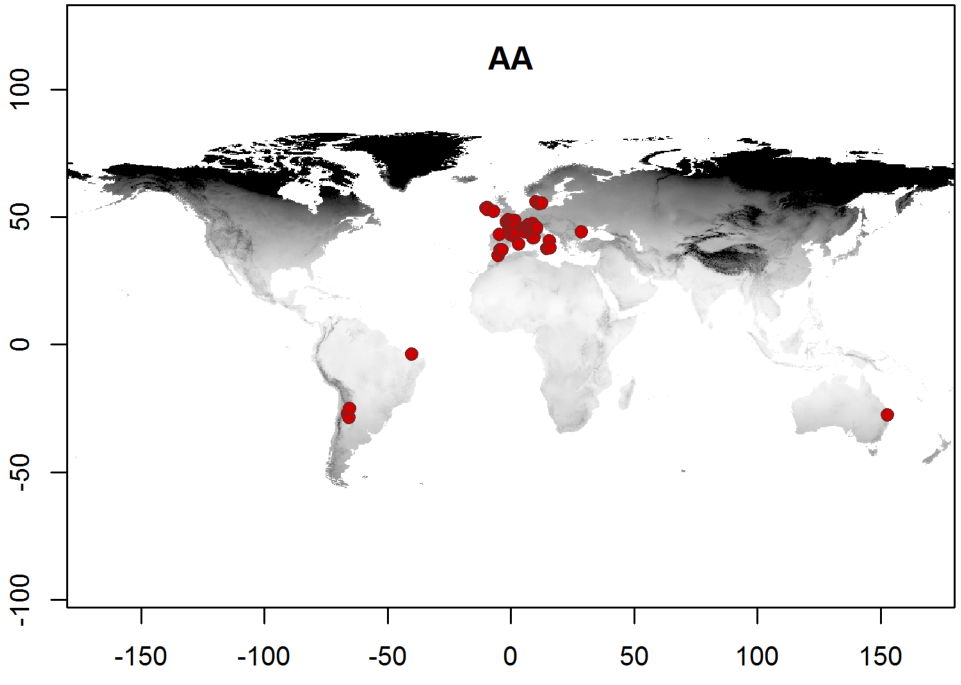

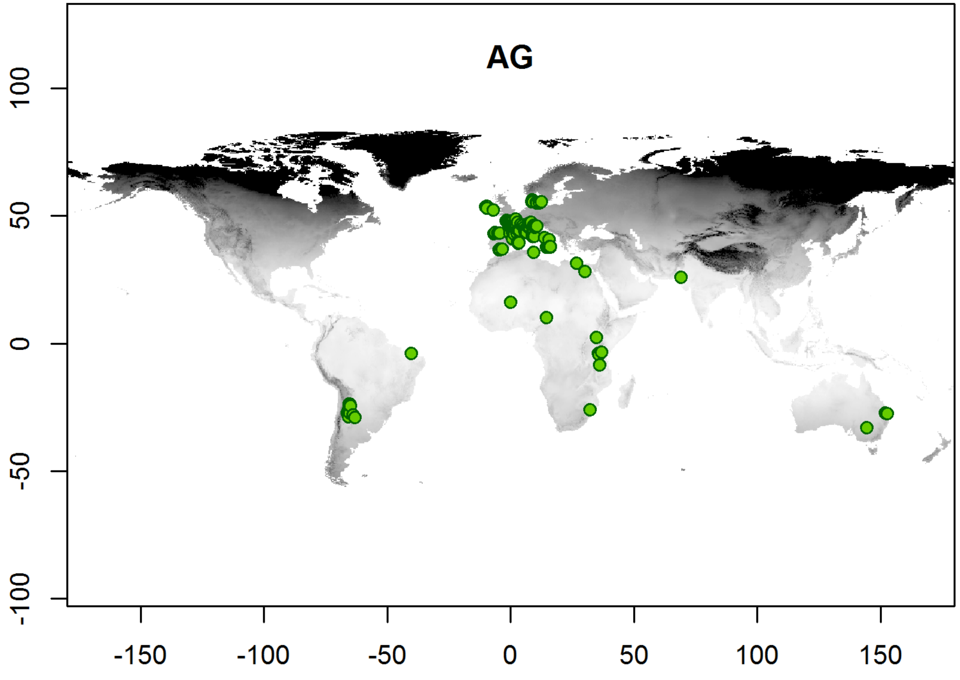

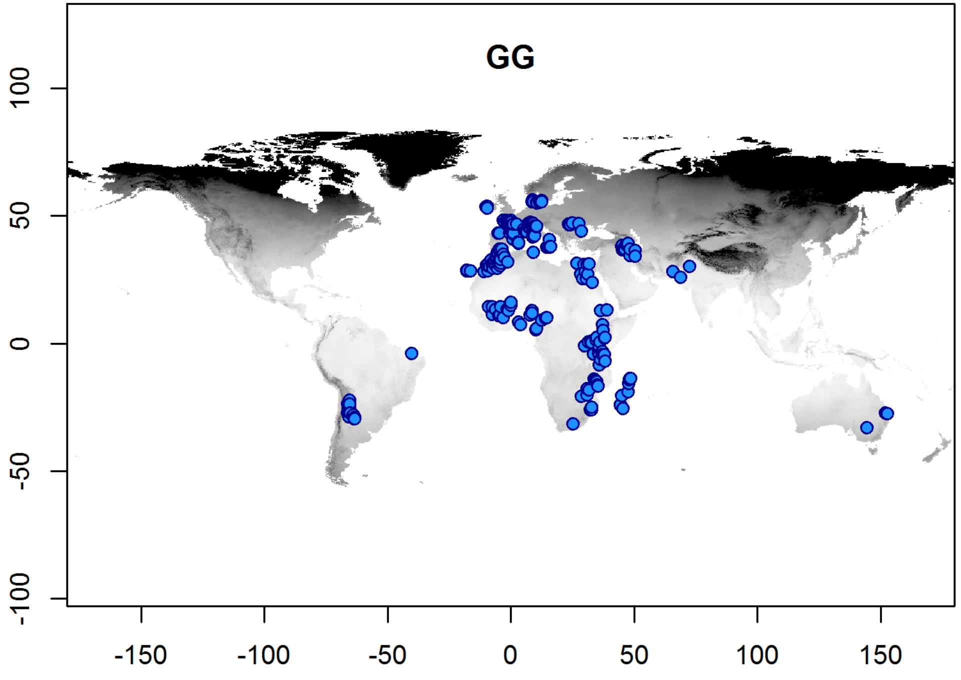

snp24975-scaffold257-240573

Chromosome: 3 : 55922240

Best association

Environmental variable = bio1

G score = 812.98

Beta 1 = 1.72

AIC = 1813.88

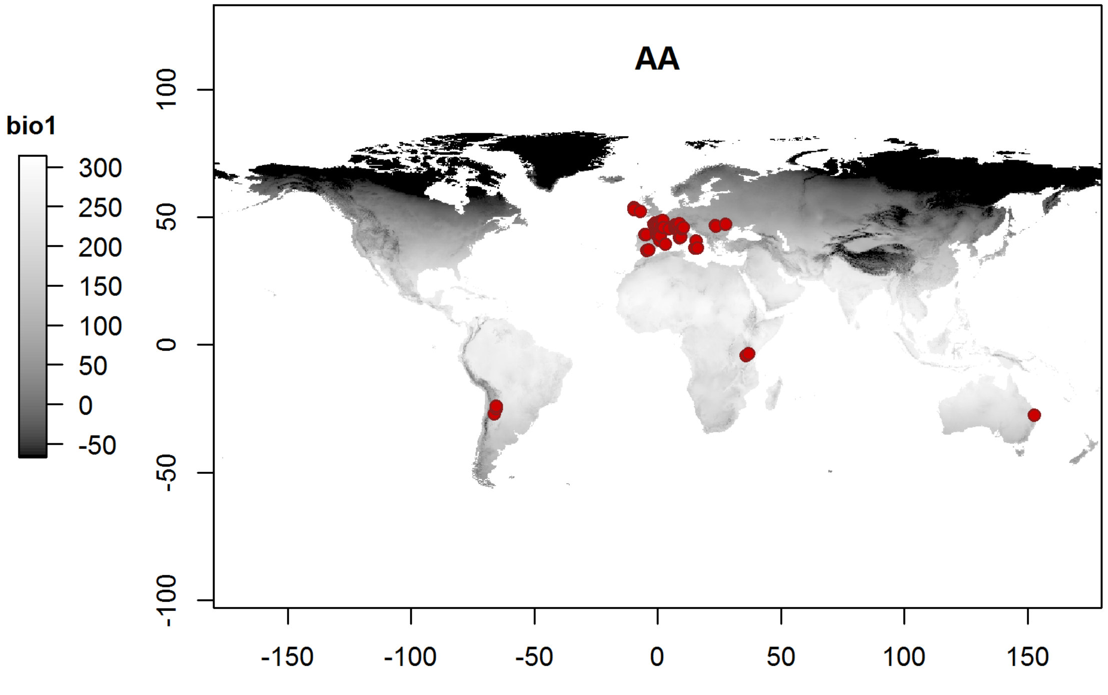

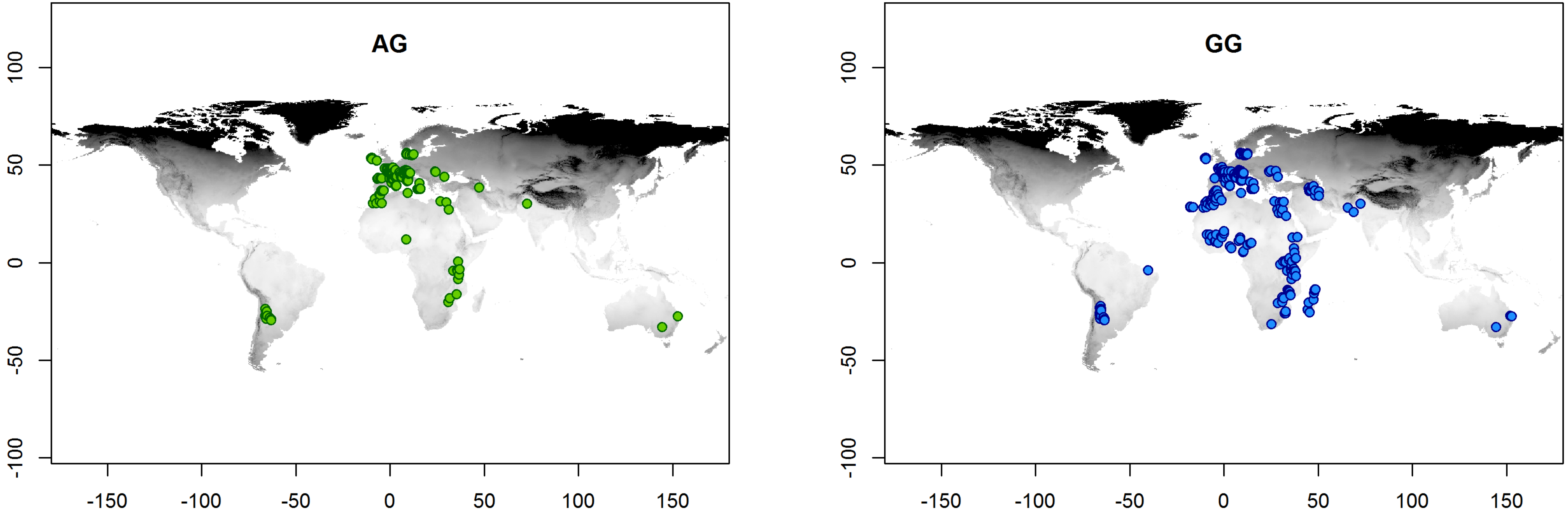

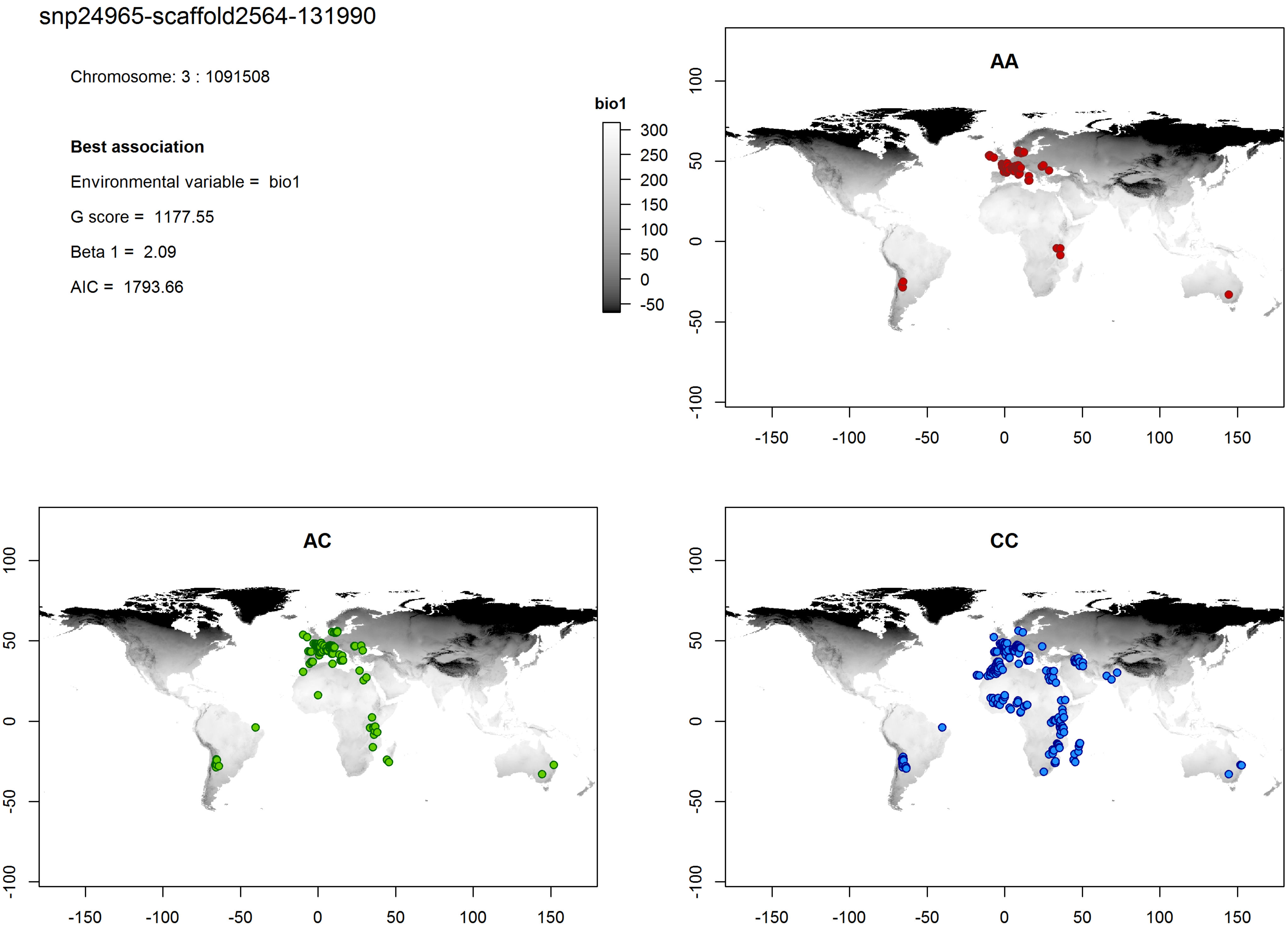
snp24965-scaffold2564-131990
Chromosome: 3 : 1091508
Best association
Environmental variable = bio1
G score = 1177.55
Beta 1 = 2.09
AIC = 1793.66
bio1
300
250
200
150
100
50
0
-50
AA
AC
CC
100
50
0
-50
-100
-150
-100
-50
0
50
100
150

snp23120-scaffold2304-195841

Chromosome: 14 : 81230482

Best association

Environmental variable = bio1

G score = 724.18

Beta 1 = 1.71

AIC = 1695.91

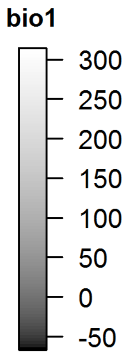

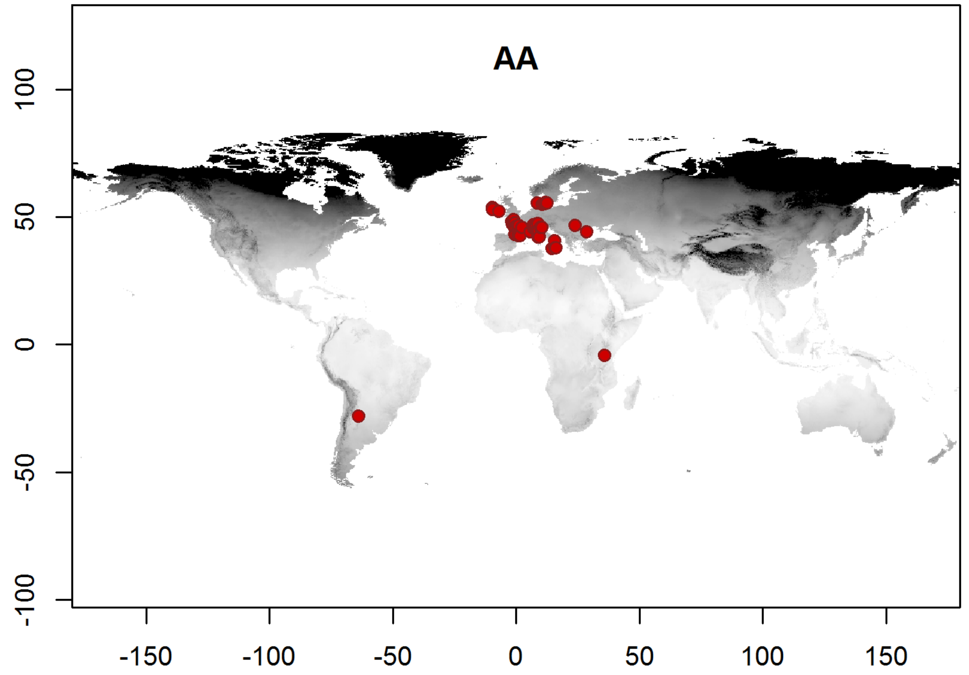

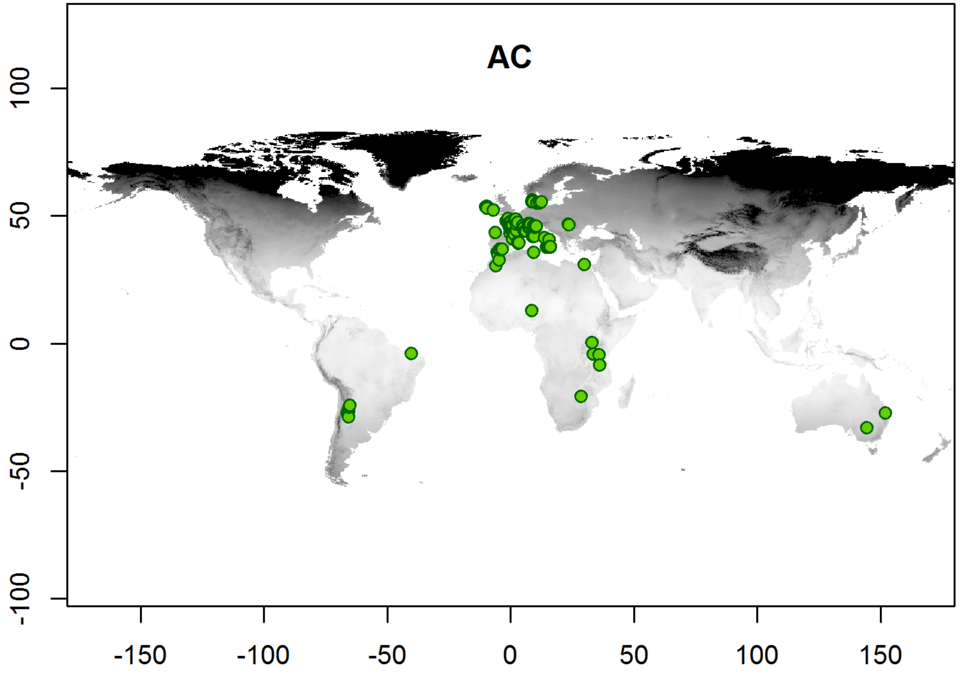

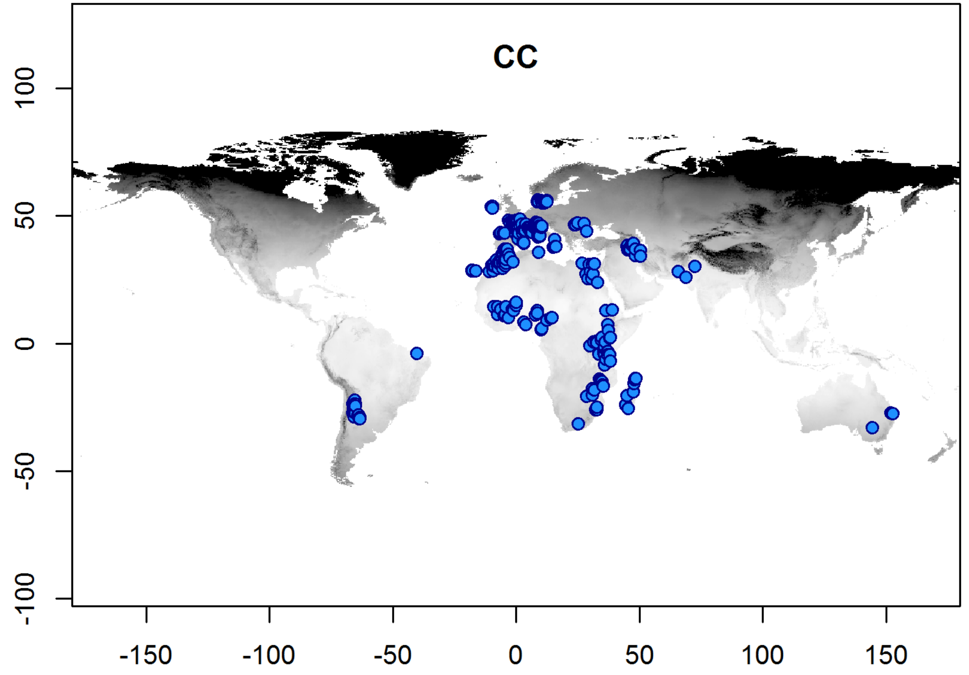

snp23119-scaffold2304-159526

Chromosome: 14 : 81267067

Best association

Environmental variable = bio1

G score = 732.64

Beta 1 = 1.75

AIC = 1658.95

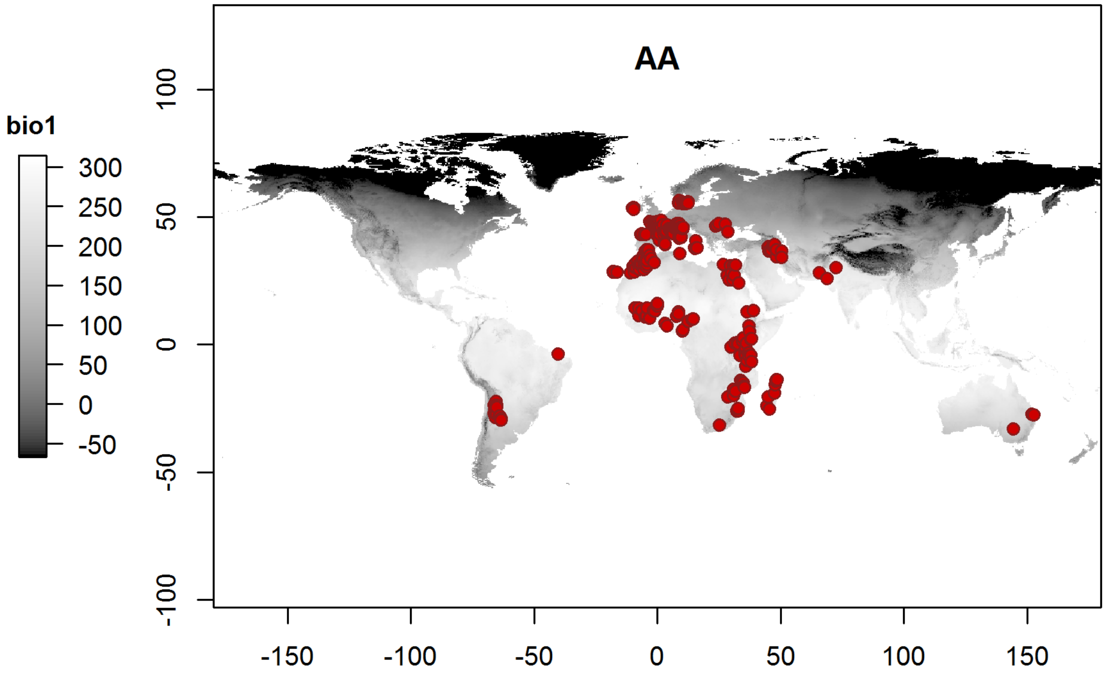

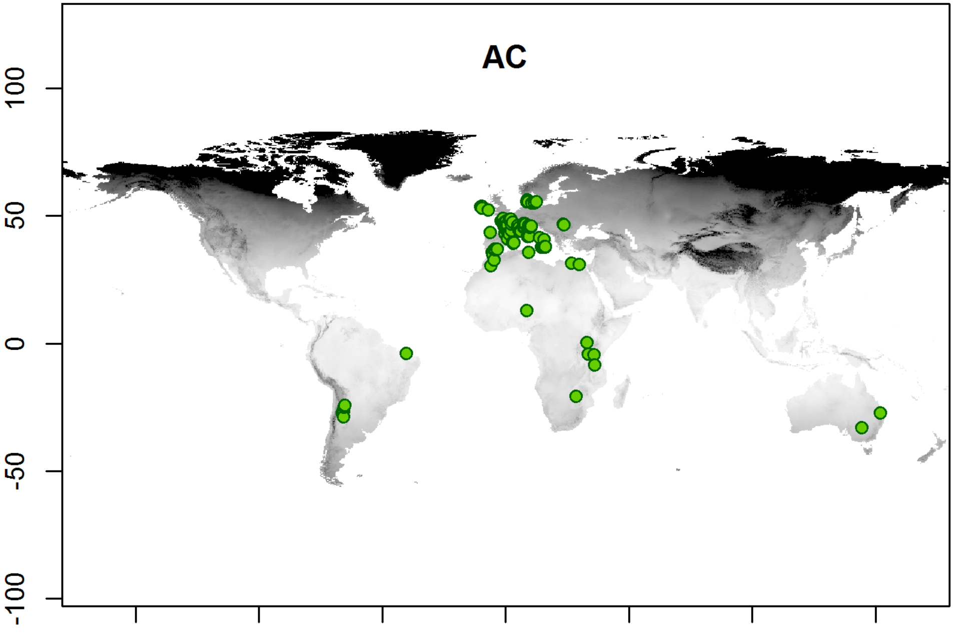

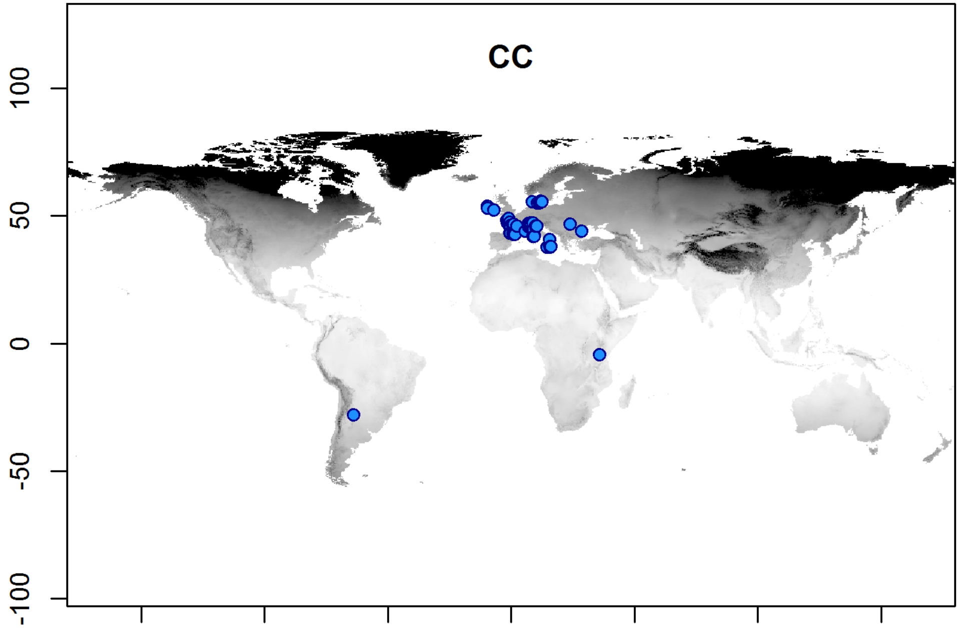

snp23118-scaffold2304-126585

Chromosome: 14 : 81291766

Best association

Environmental variable = bio11

G score = 552.08

Beta 1 = 1.62

AIC = 1510.94

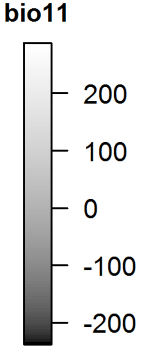

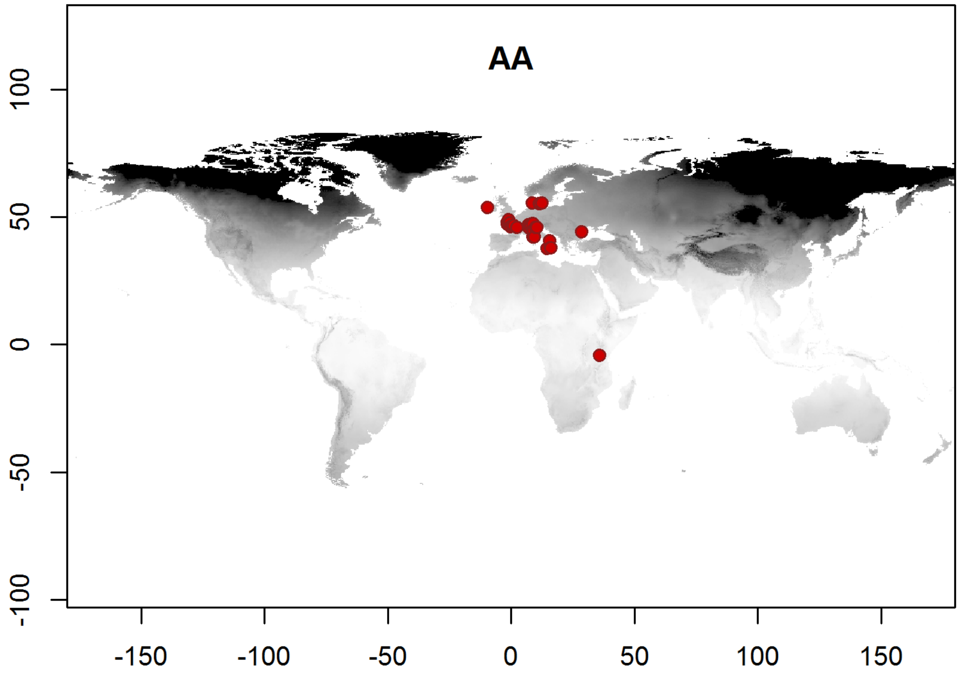

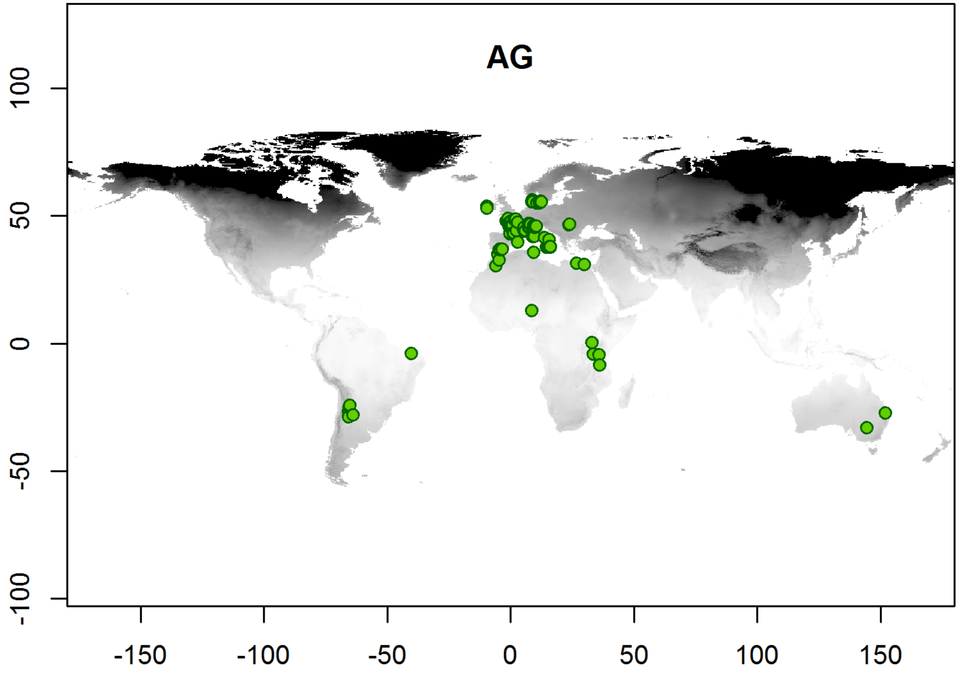

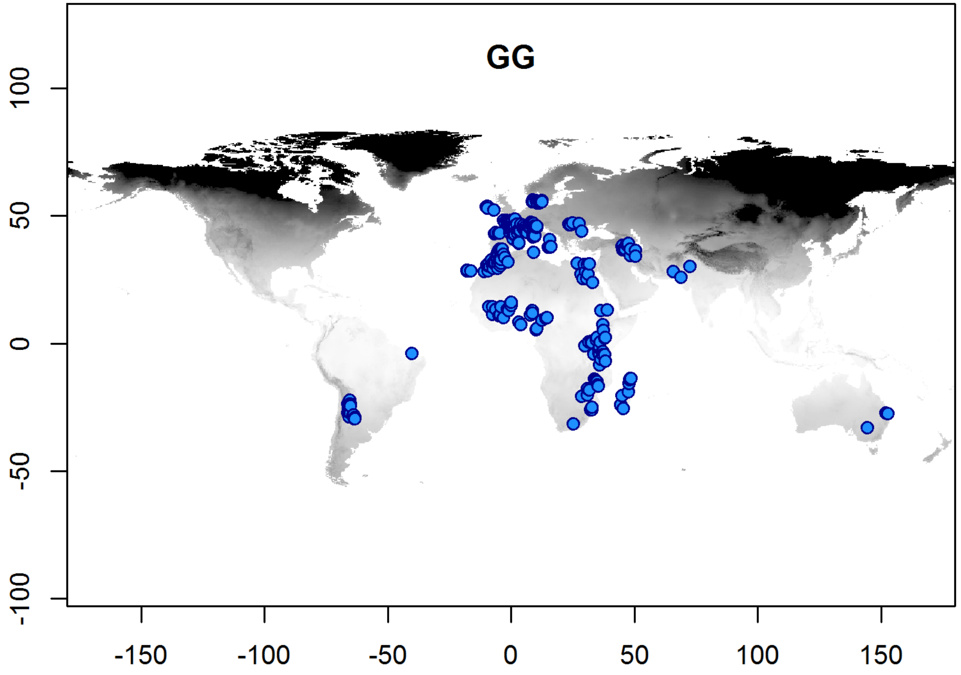

snp22598-scaffold2239-125488

Chromosome: 5 : 98061330

Best association

Environmental variable = bio1

G score = 676.53

Beta 1 = 1.63

AIC = 1733.27

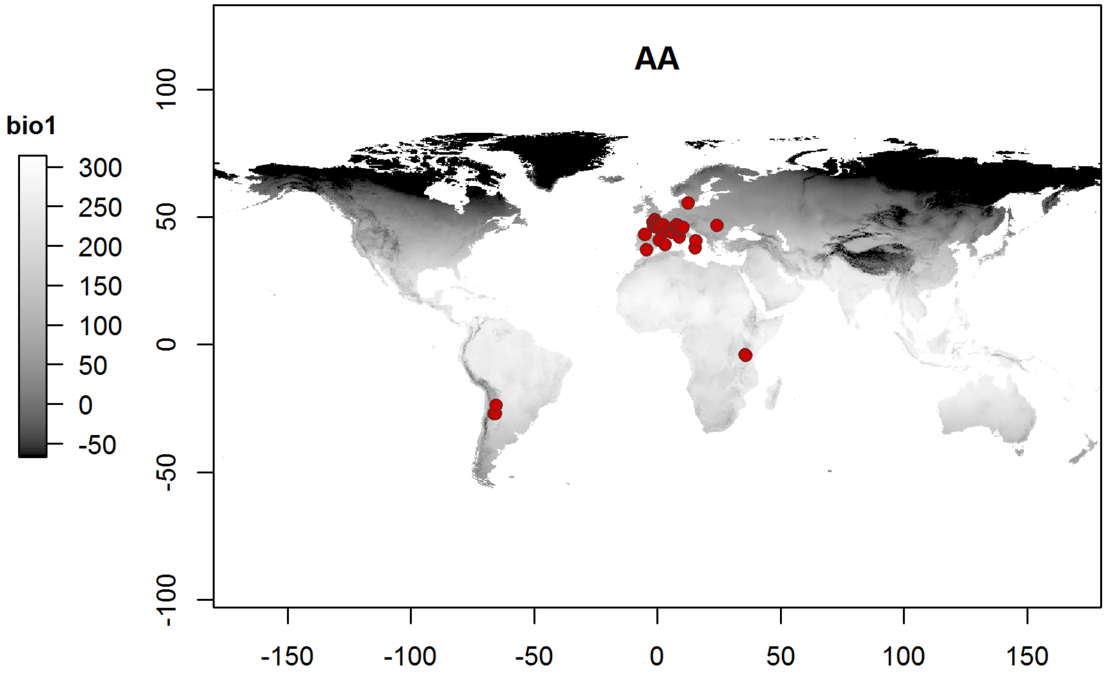

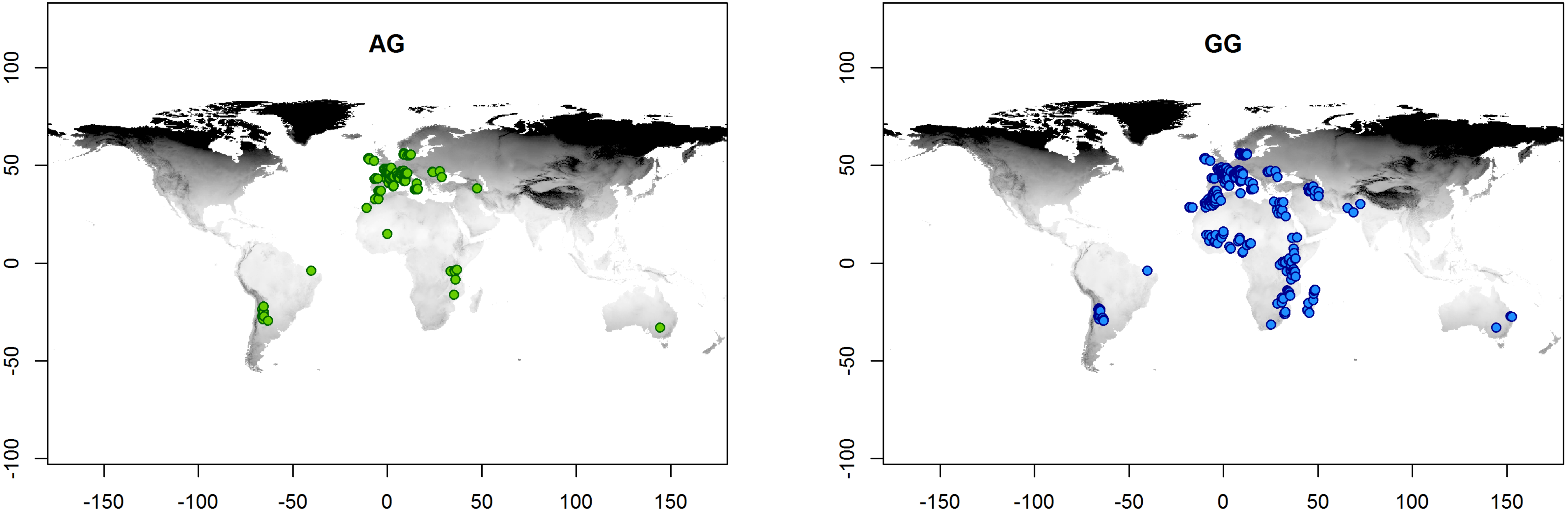

snp21944-scaffold2156-537302

Chromosome: 4 : 26776780

Best association

Environmental variable = bio1

G score = 695.59

Beta 1 = 1.58

AIC = 1836.71

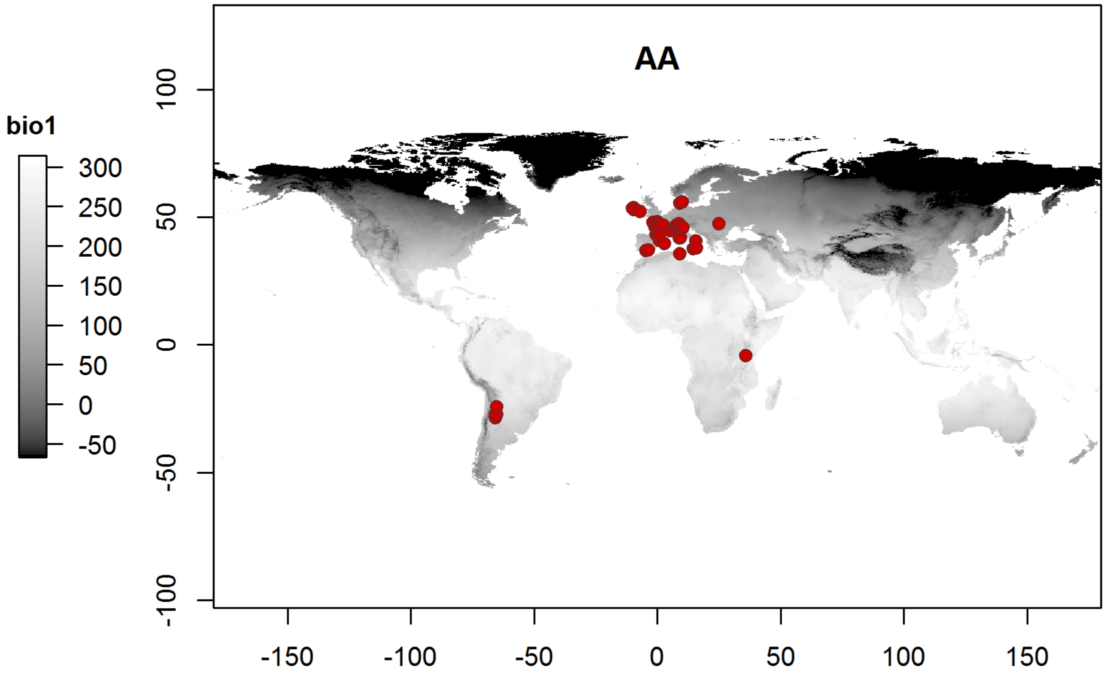

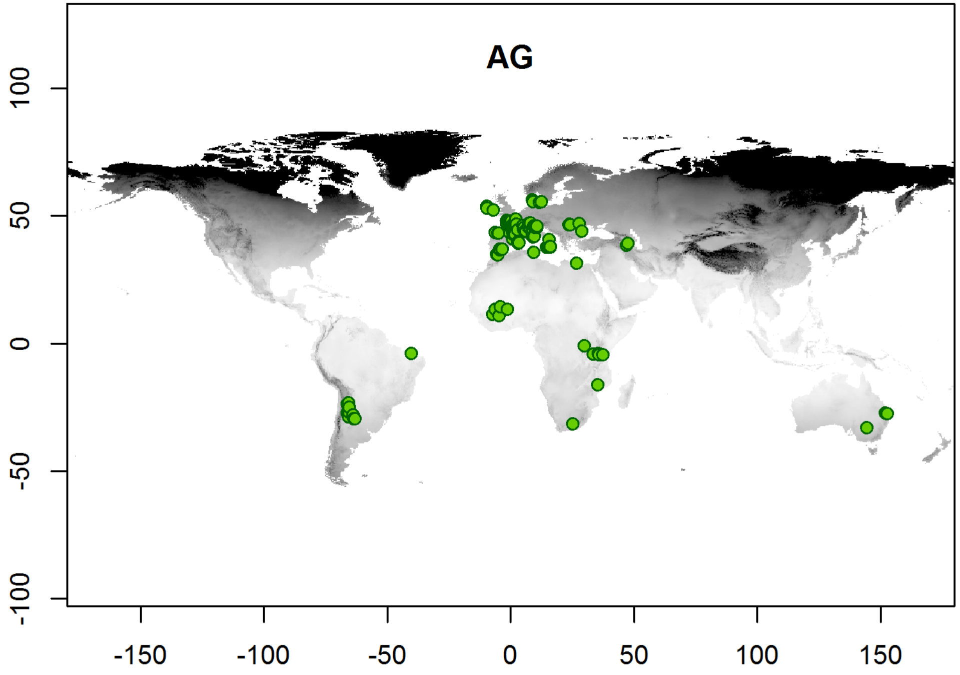

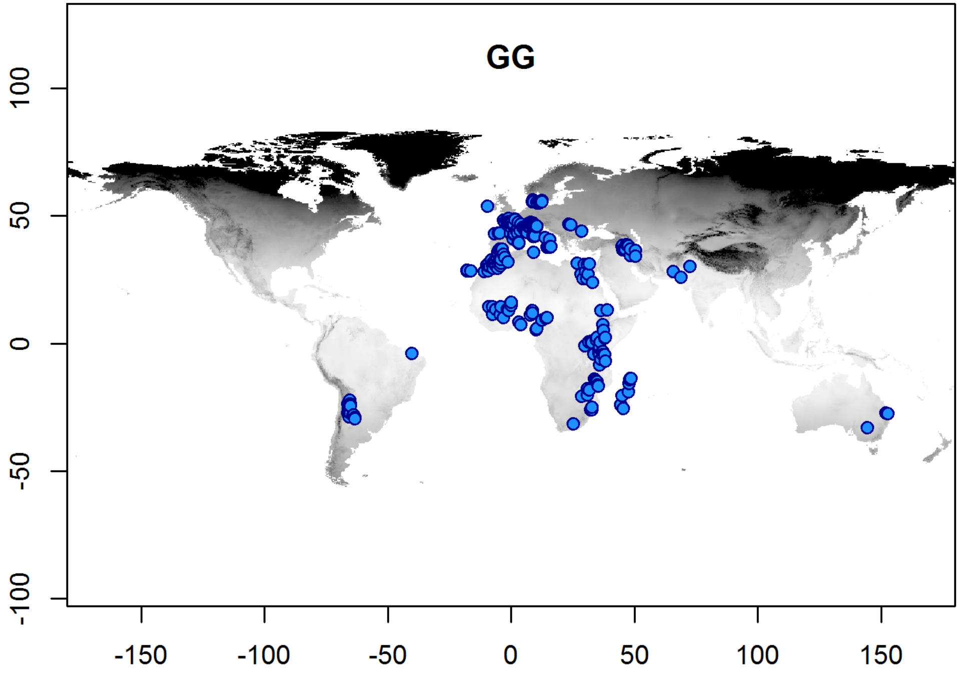

snp19629-scaffold1983-289157

Chromosome: 14 : 91122307

Best association

Environmental variable = bio1

G score = 541.49

Beta 1 = 1.53

AIC = 1619.09

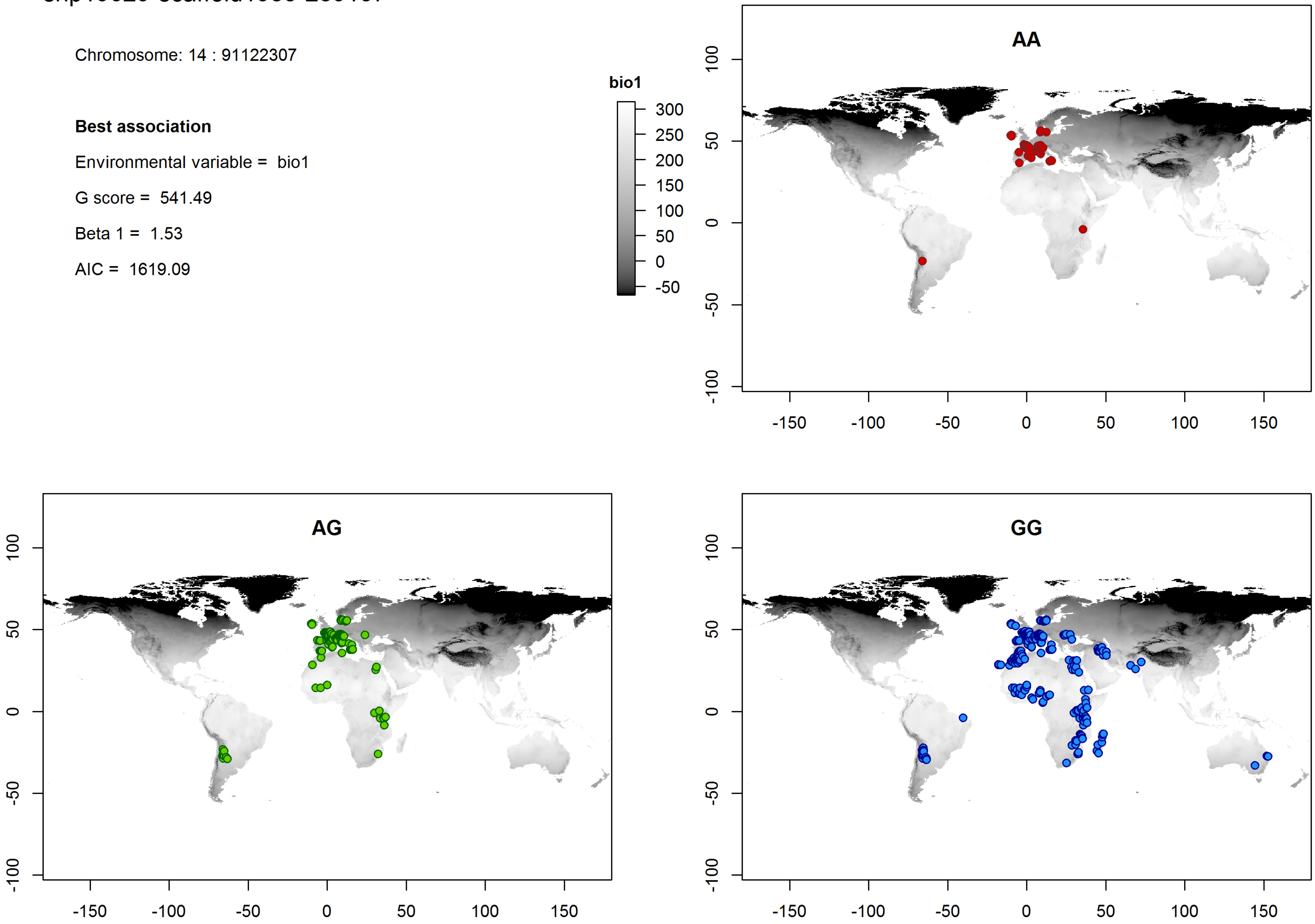

snp19202-scaffold1931-360047

Chromosome: 7 : 95367449

Best association

Environmental variable = bio1

G score = 429.09

Beta 1 = 1.47

AIC = 1453.42

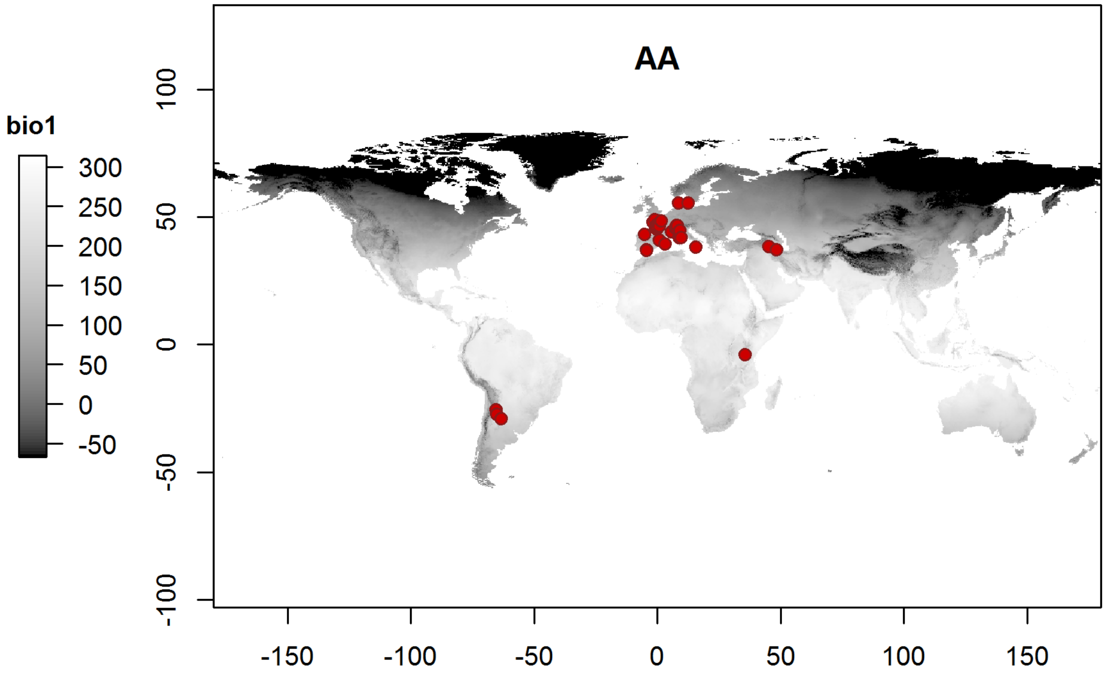

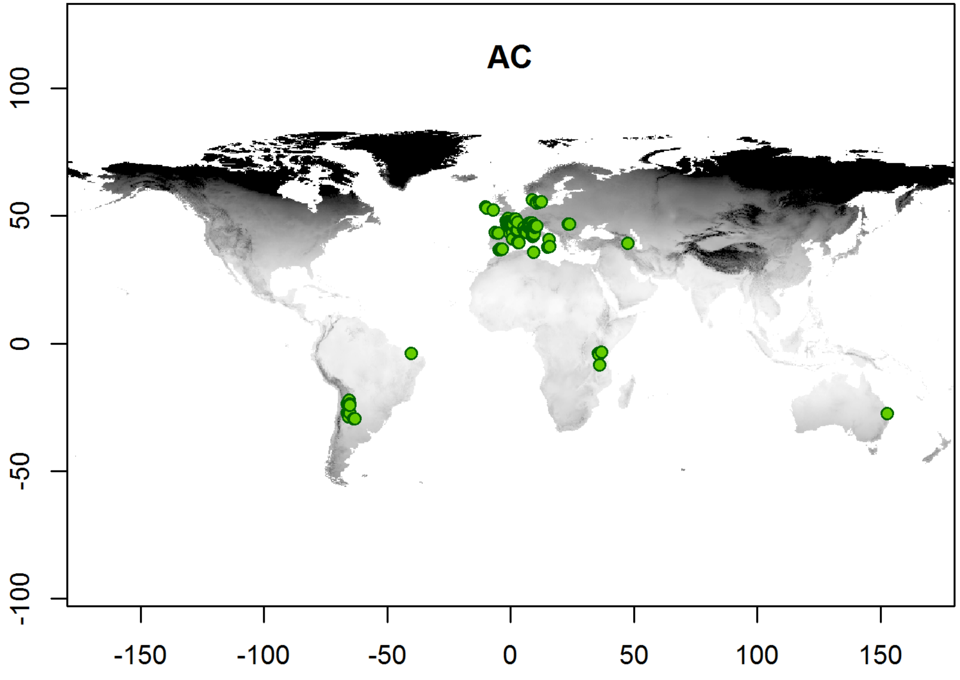

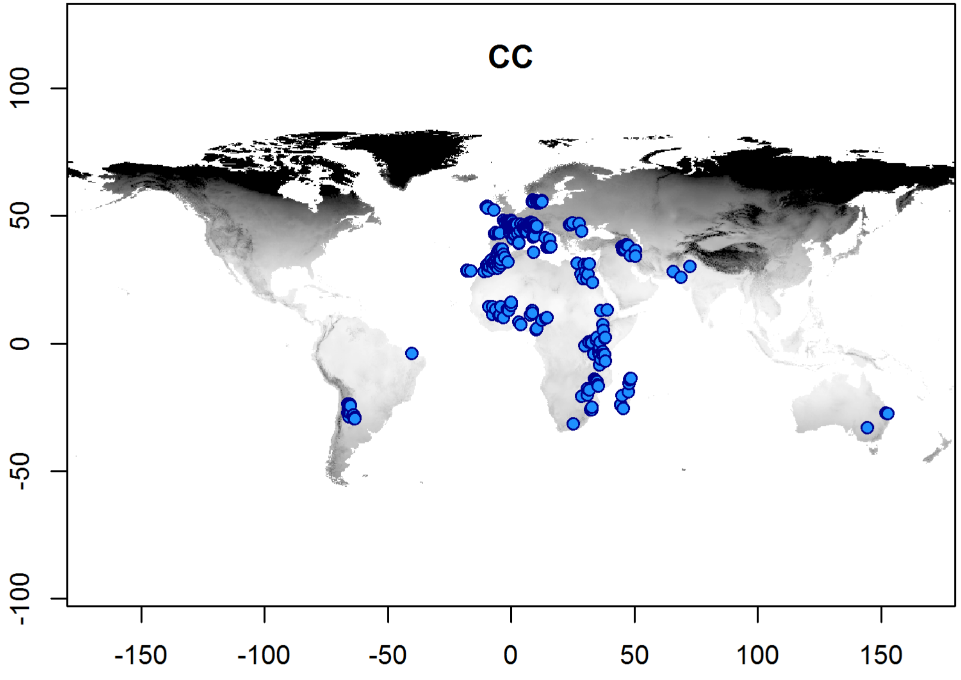

snp18571-scaffold1878-249261

Chromosome: 26 : 14182177

Best association

Environmental variable = bio1

G score = 511

Beta 1 = 1.49

AIC = 1619.58

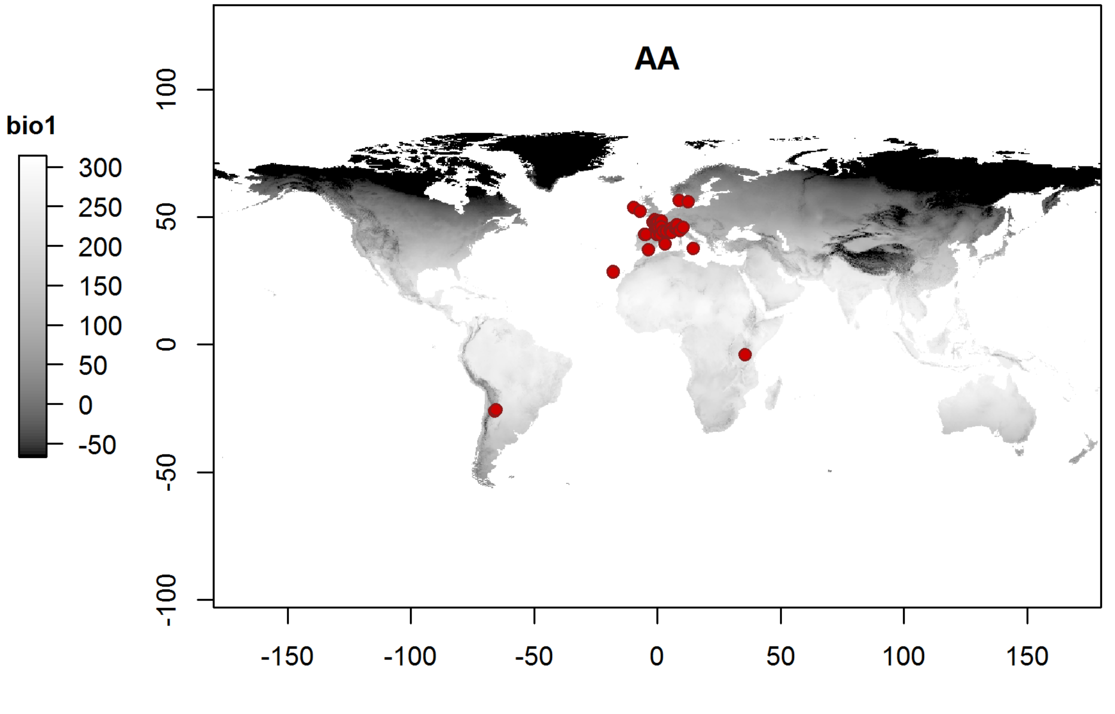

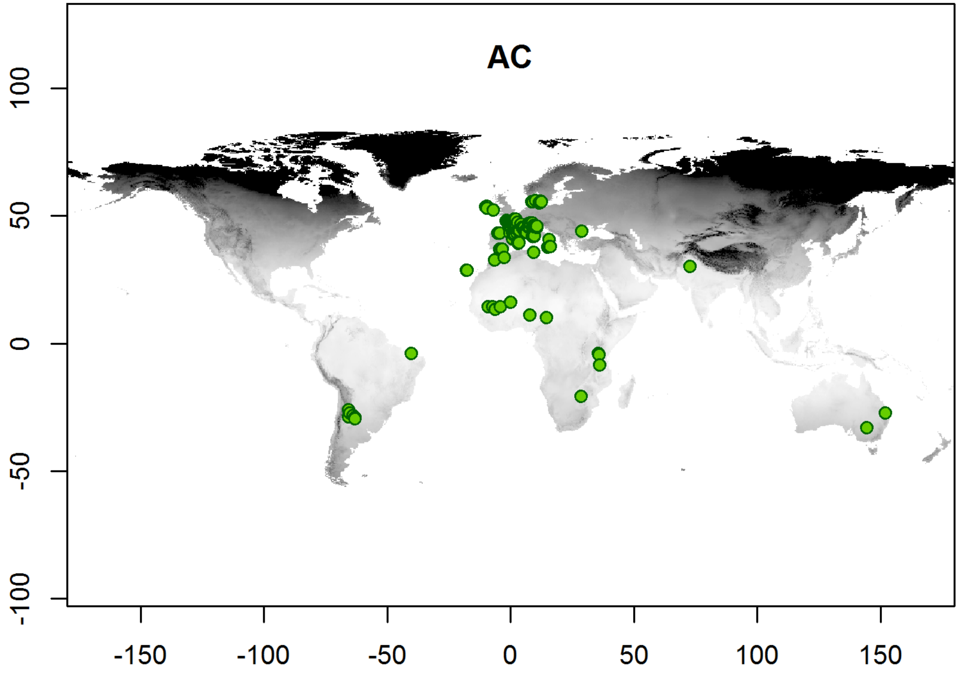

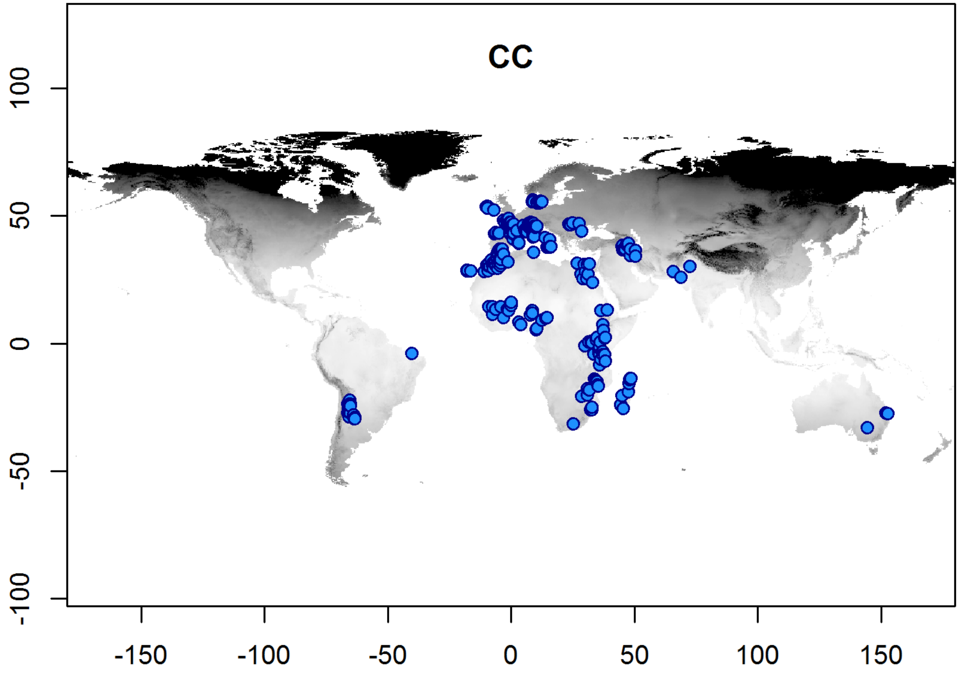

snp18290-scaffold1857-340926

Chromosome: 18 : 56302975

Best association

Environmental variable = bio1

G score = 806.68

Beta 1 = 1.72

AIC = 1807.5

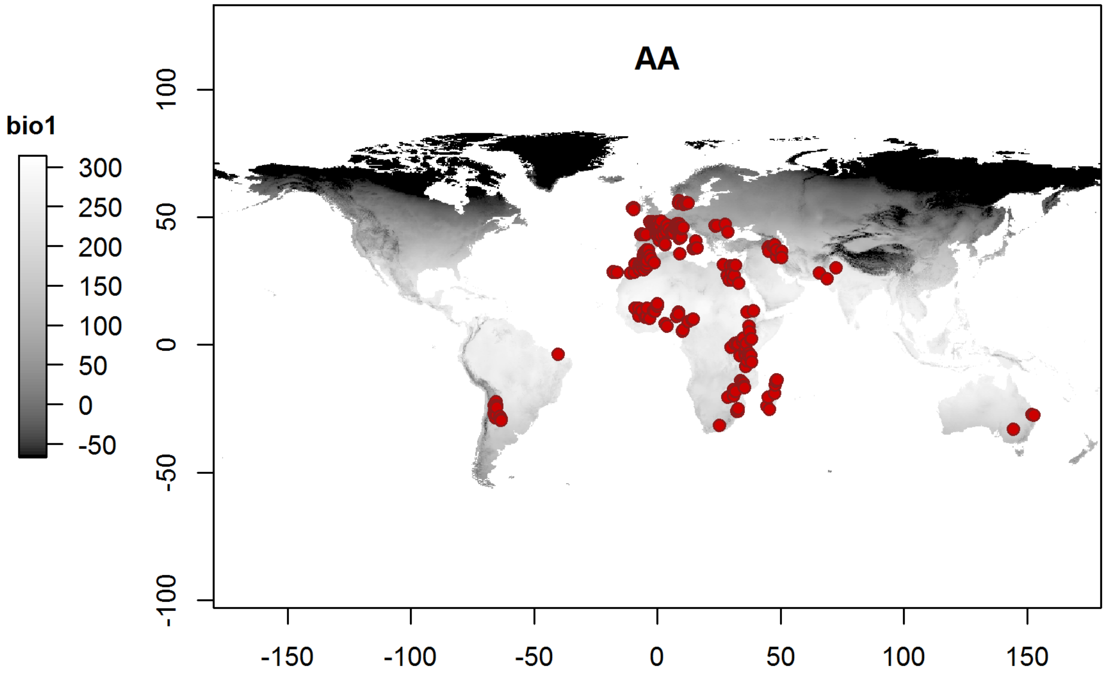

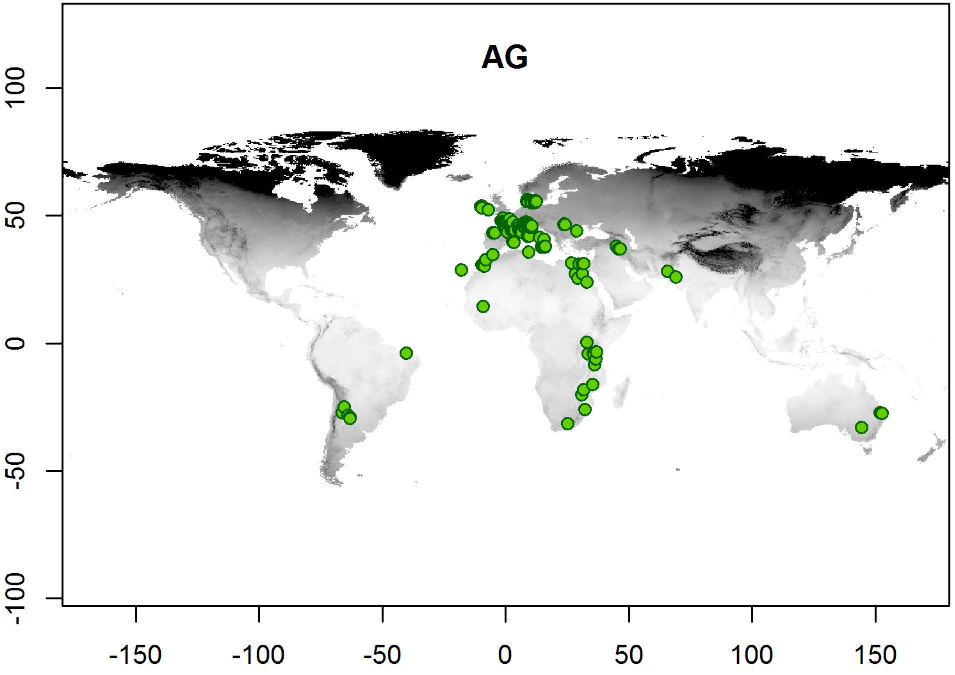

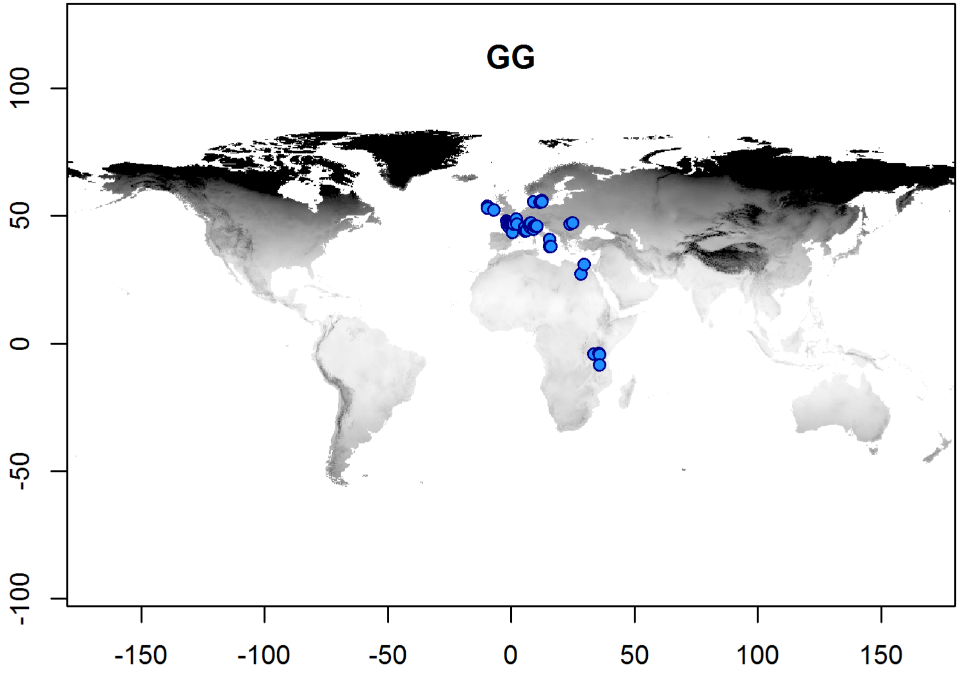

snp18275-scaffold1855-155165

Chromosome: 4 : 6347244

Best association

Environmental variable = bio1

G score = 518.43

Beta 1 = 1.39

AIC = 1799.52

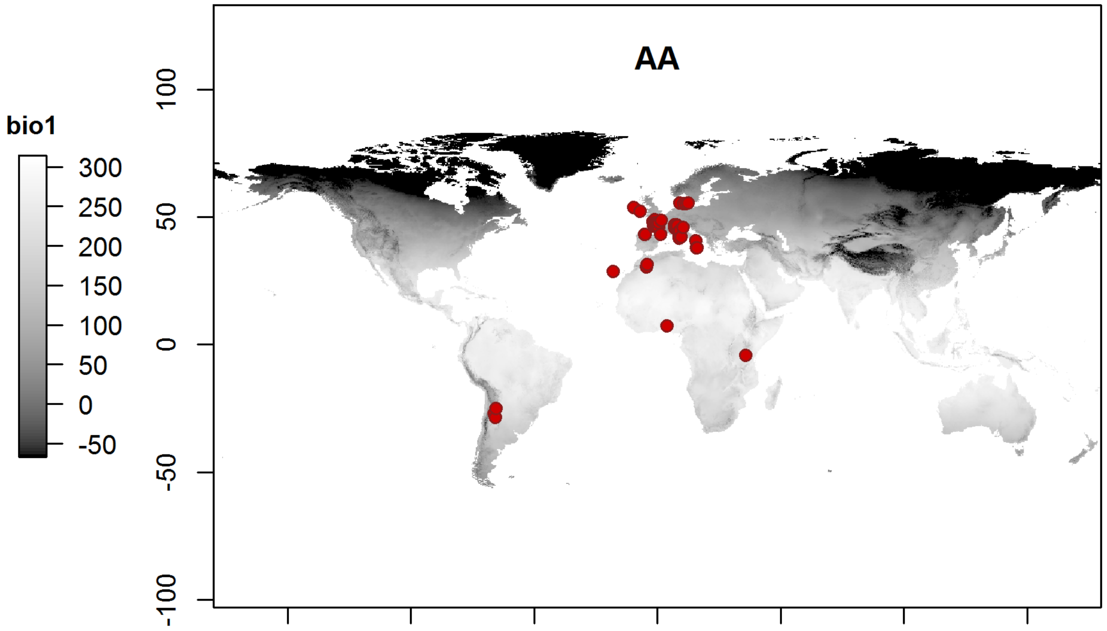

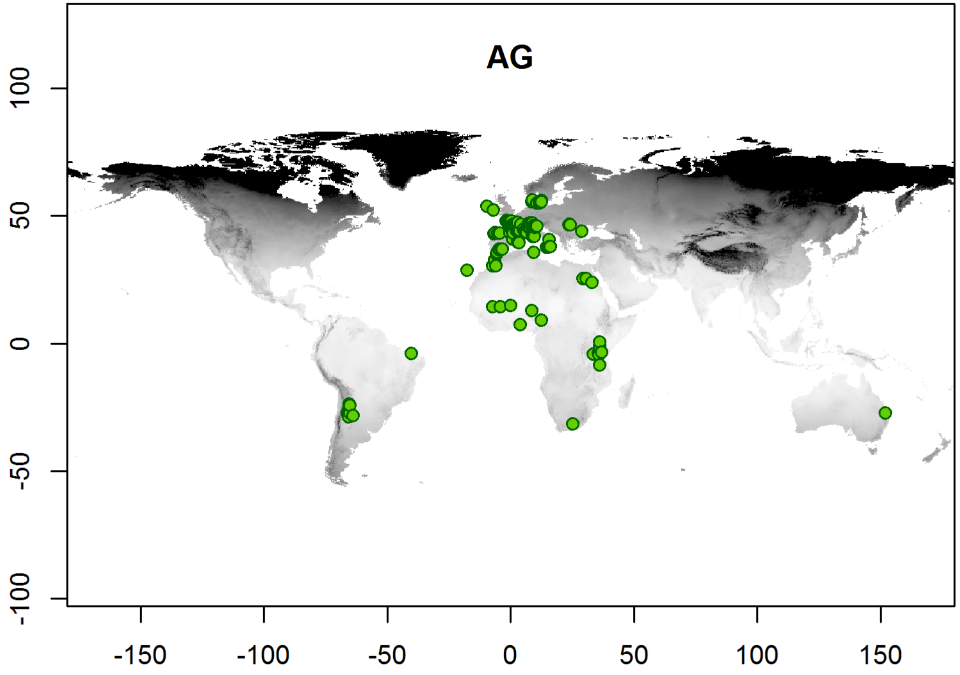

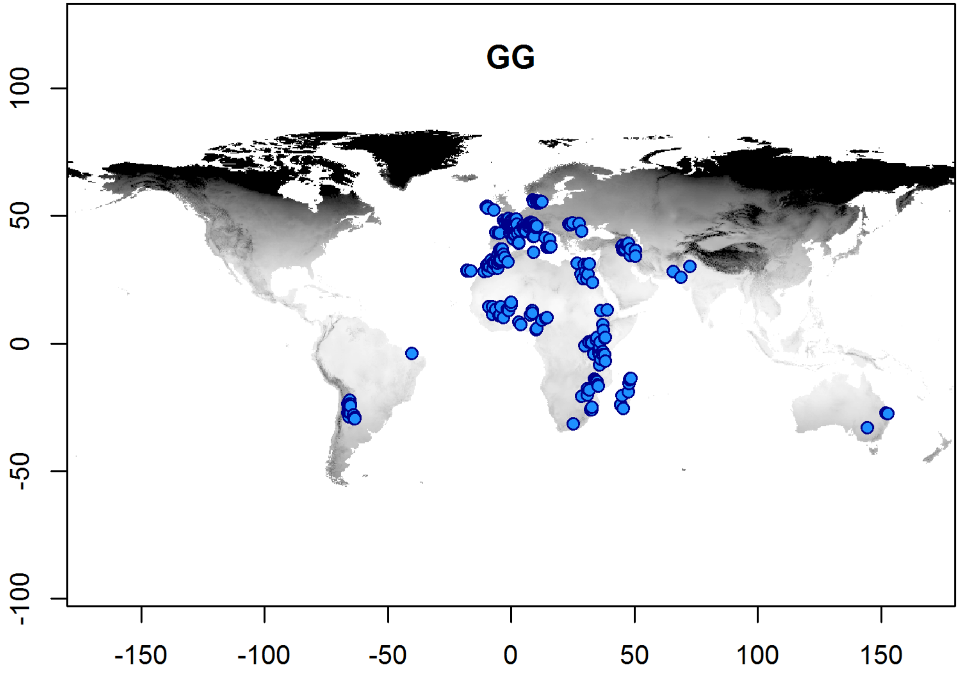

snp17046-scaffold1777-78487

Chromosome: 3 : 111943981

Best association

Environmental variable = bio1

G score = 774.66

Beta 1 = 1.66

AIC = 1856.56

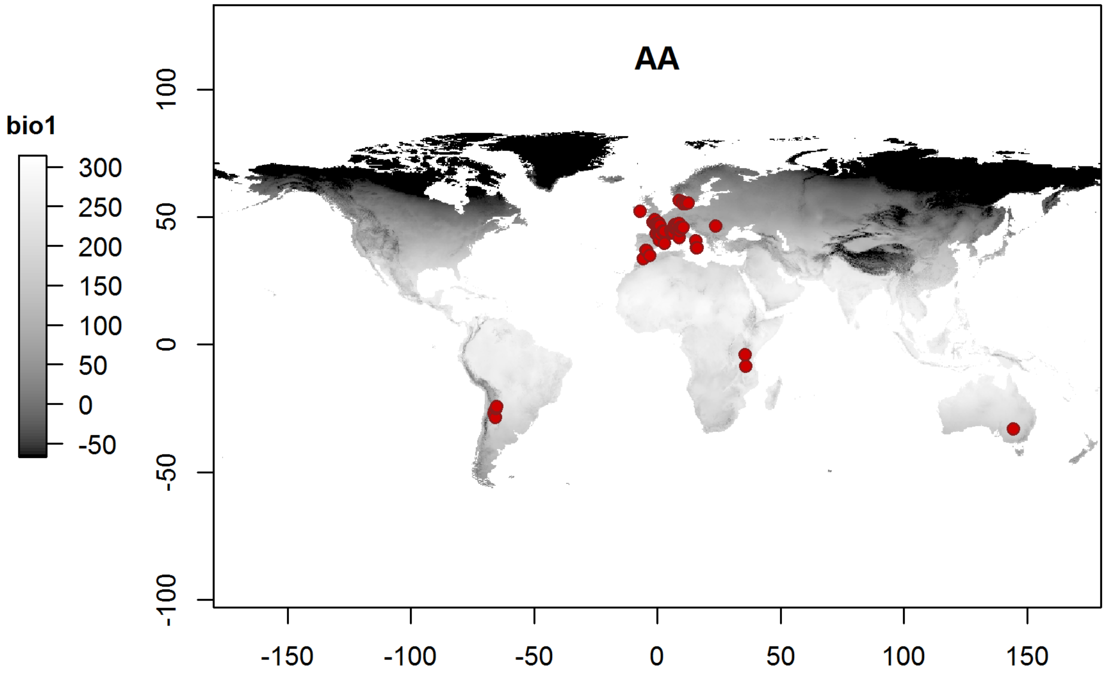

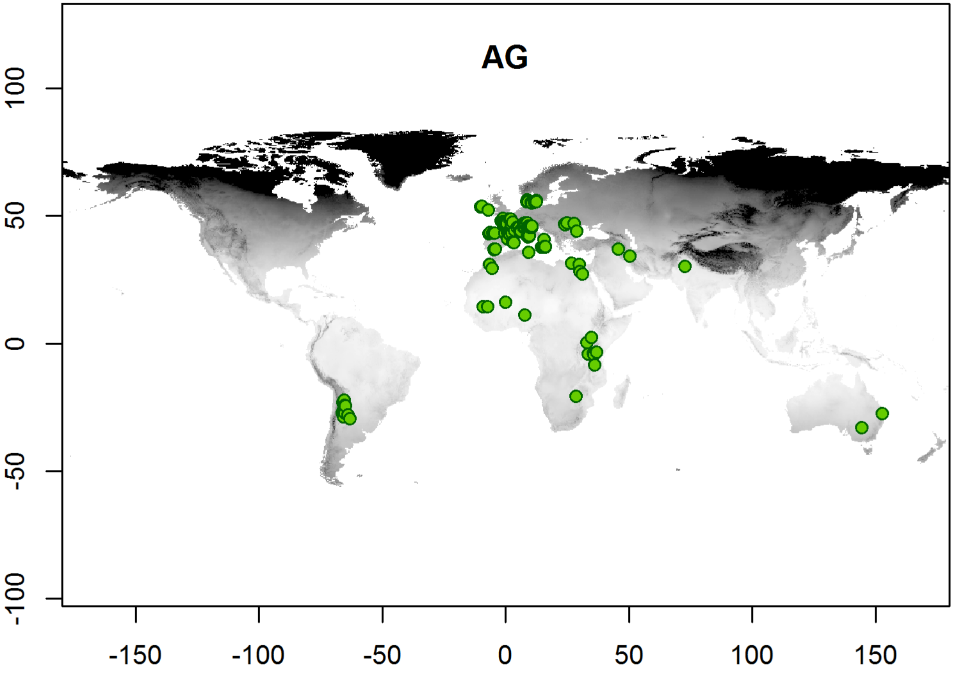

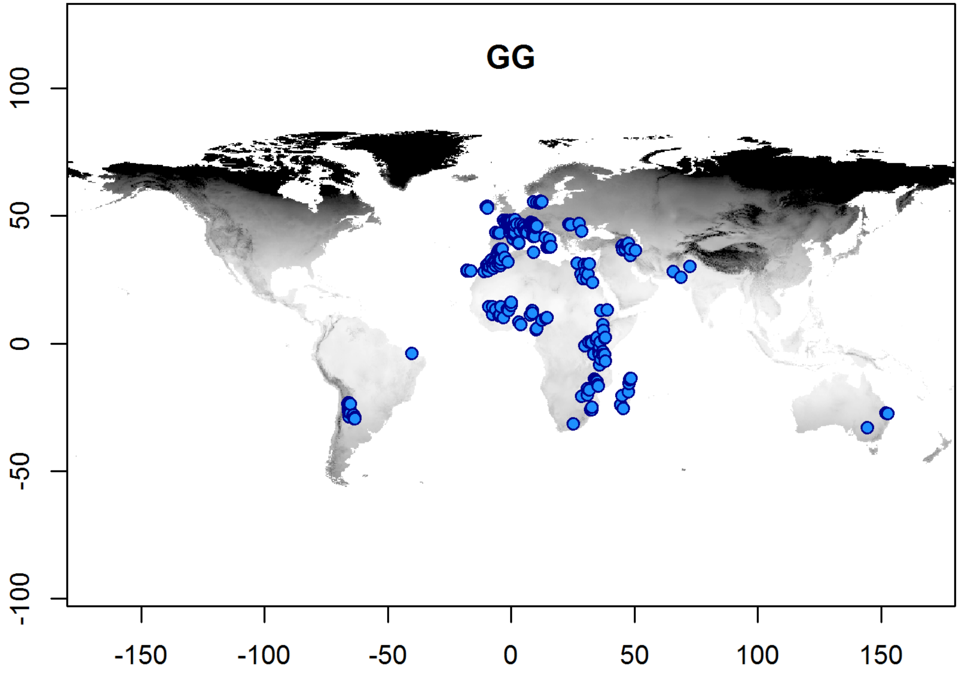

snp59380-scaffold979-422092

Chromosome: 1 : 1361457

Best association

Environmental variable = bio2

G score = 566.47

Beta 1 = 1.71

AIC = 1568.57

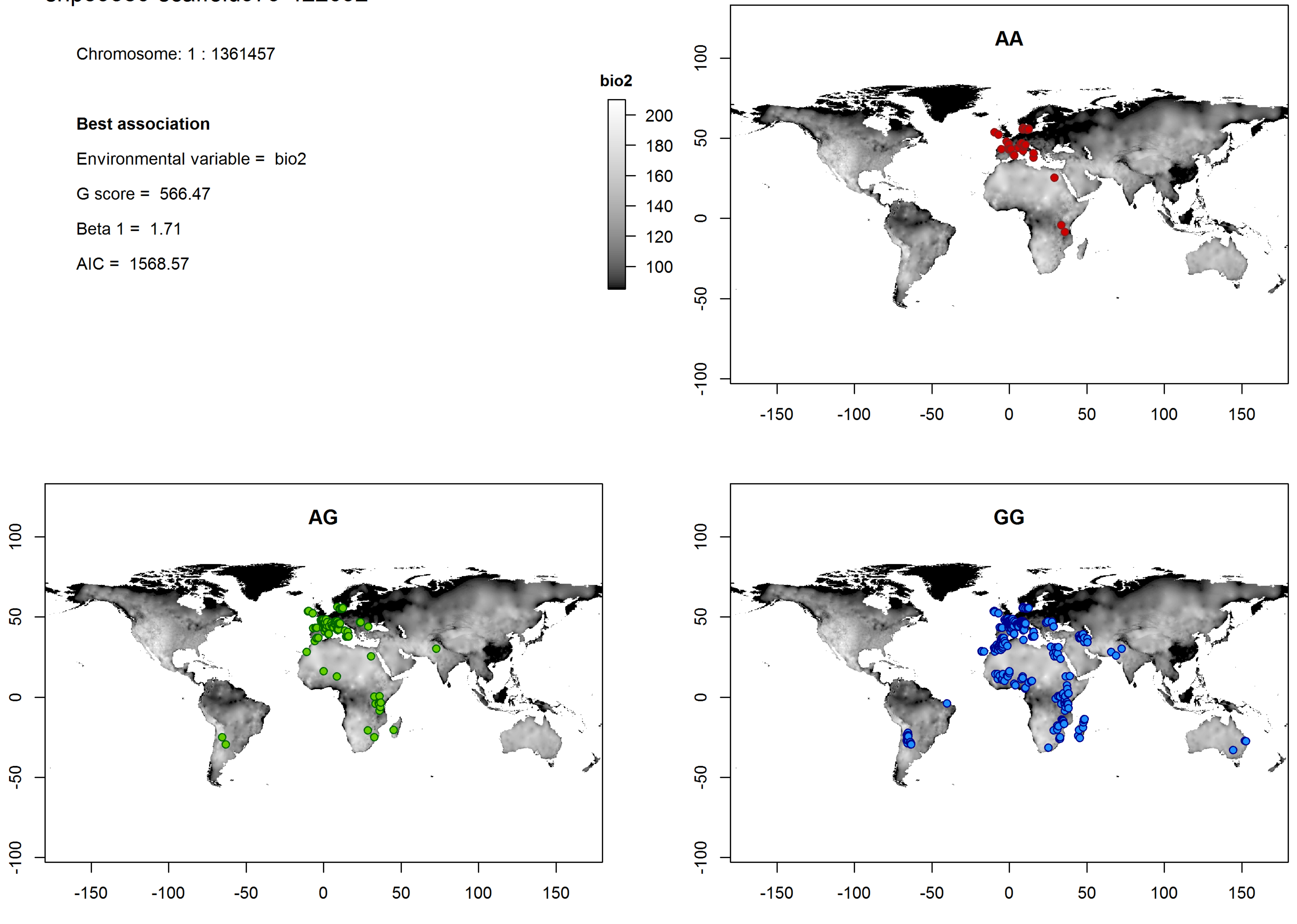

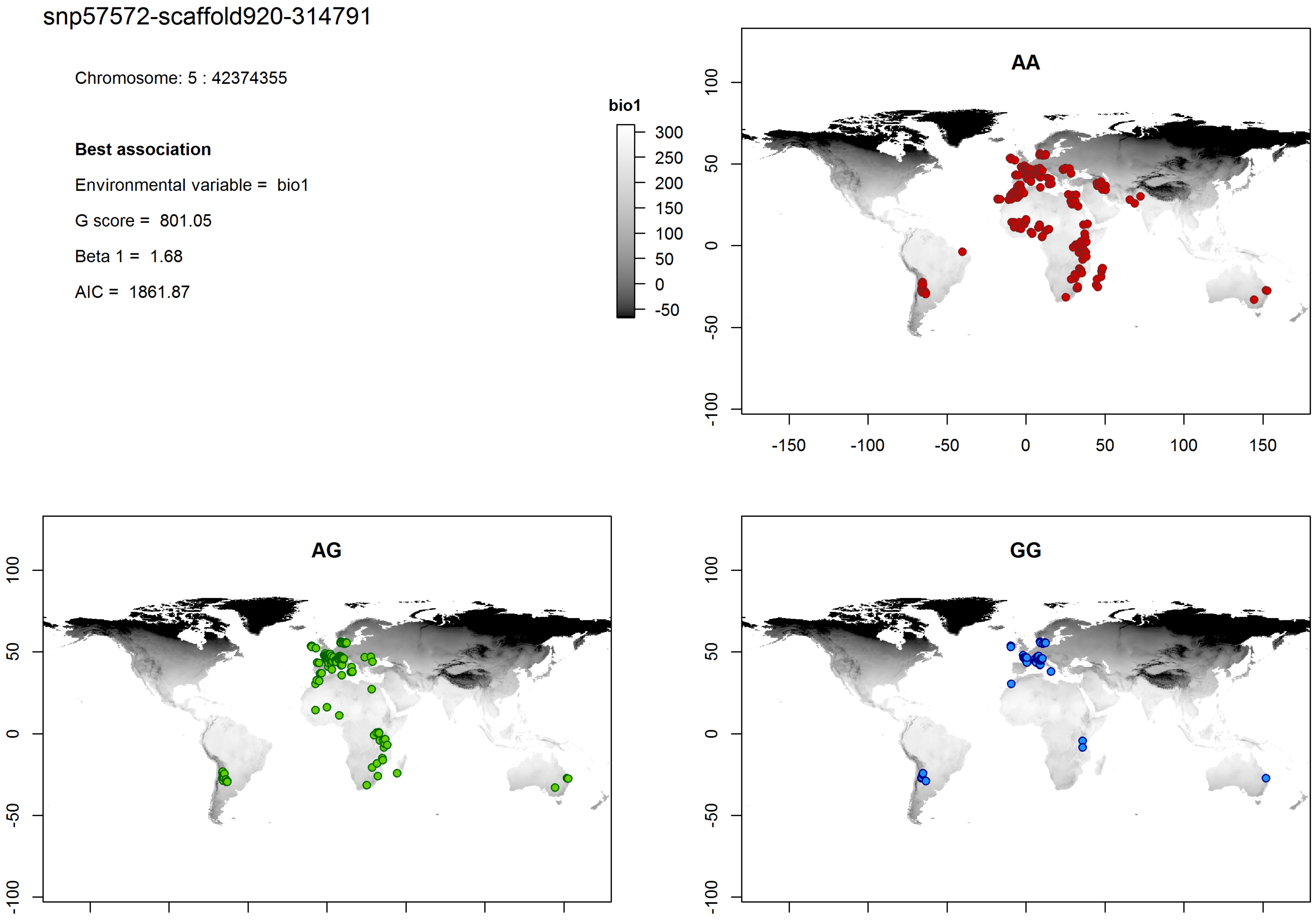
snp57572-scaffold920-314791
Chromosome: 5 : 42374355
Best association
Environmental variable = bio1
G score = 801.05
Beta 1 = 1.68
AIC = 1861.87
bio1
300
250
200
150
100
50
0
-50
AA
AG
GG
100
50
0
-50
-100
-150
-100
-50
0
50
100
150

snp56061-scaffold875-397187

Chromosome: 13 : 15504116

Best association

Environmental variable = bio1

G score = 470.88

Beta 1 = 1.43

AIC = 1615.9

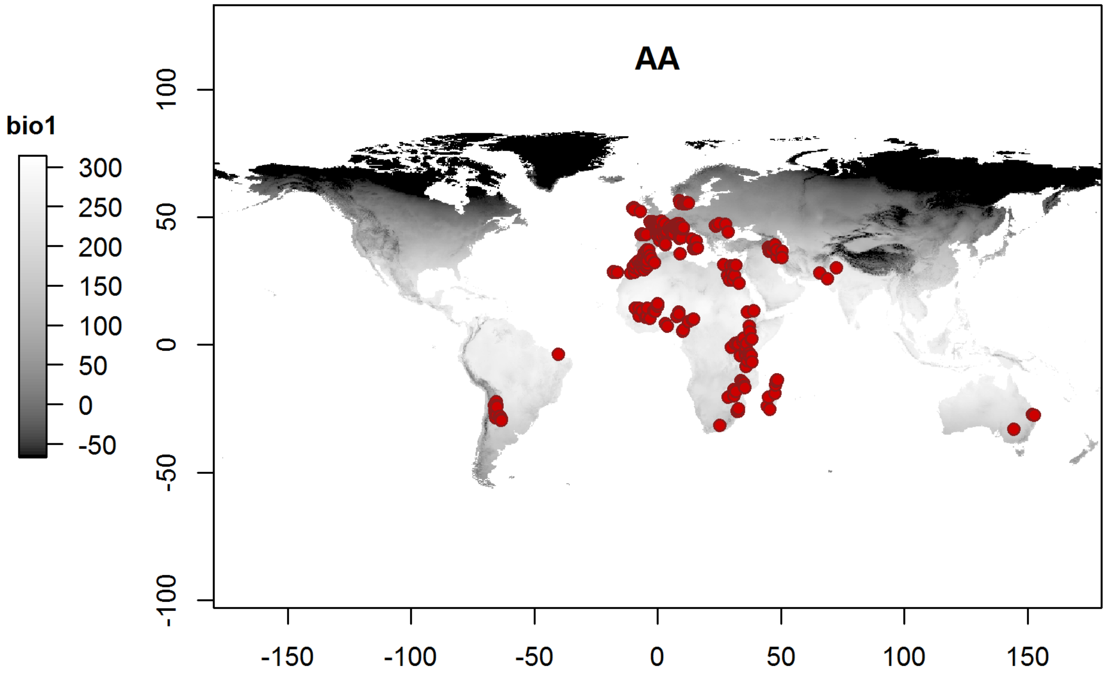

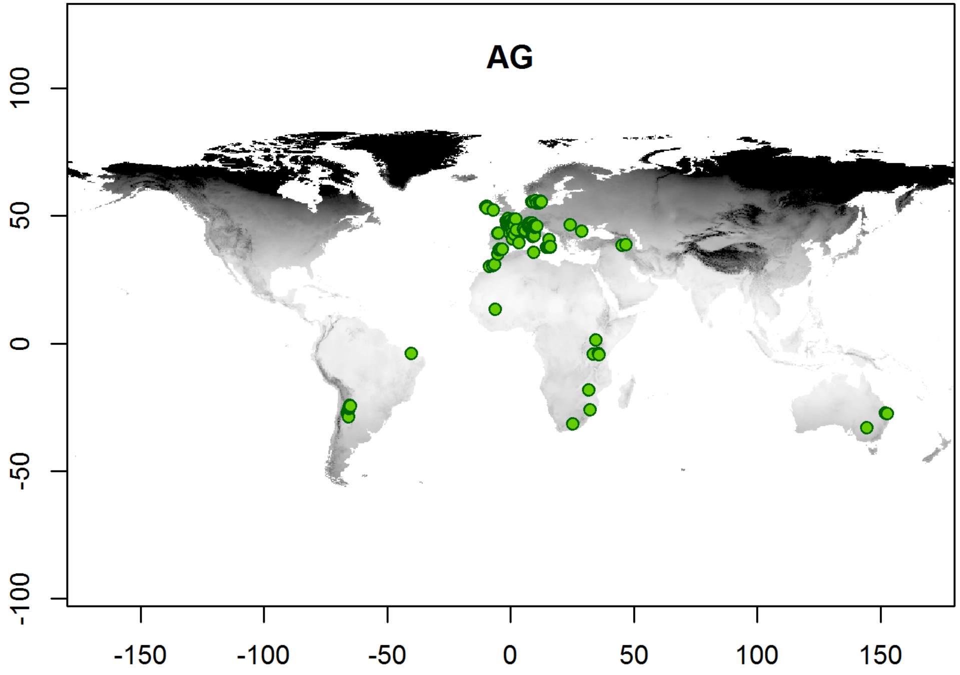

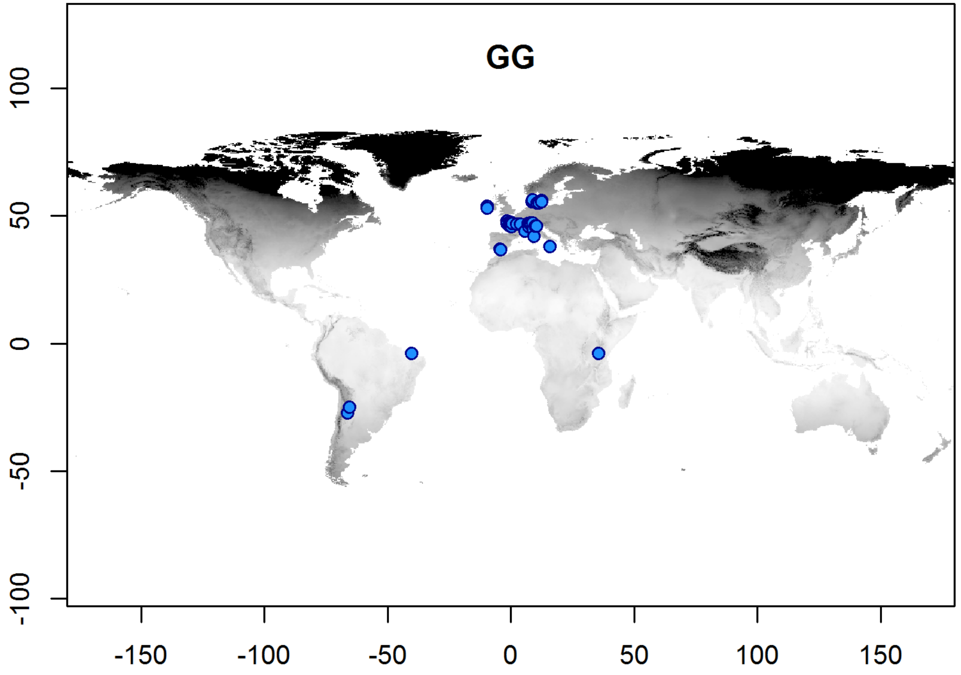

snp55752-scaffold864-3177522

Chromosome: 27 : 19963783

Best association

Environmental variable = bio1

G score = 578.7

Beta 1 = 1.62

AIC = 1570.08

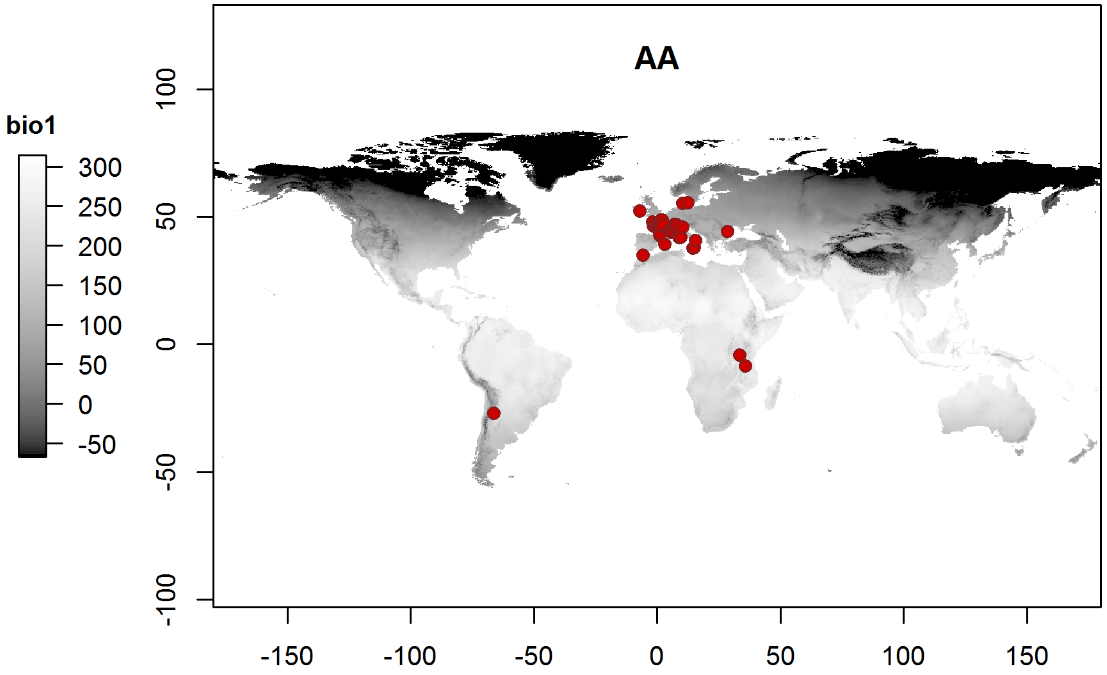

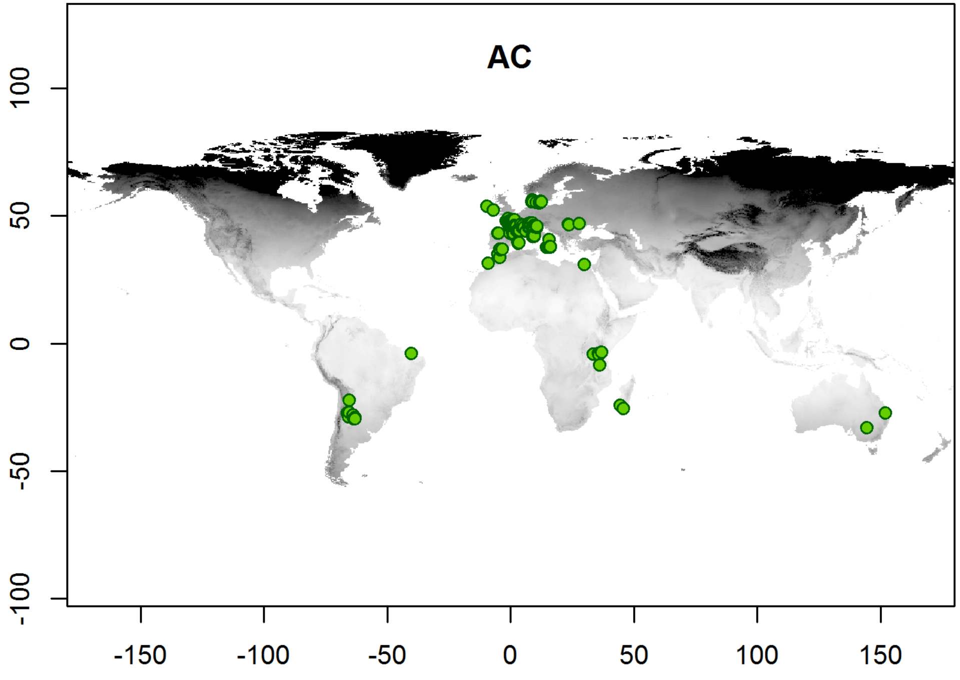

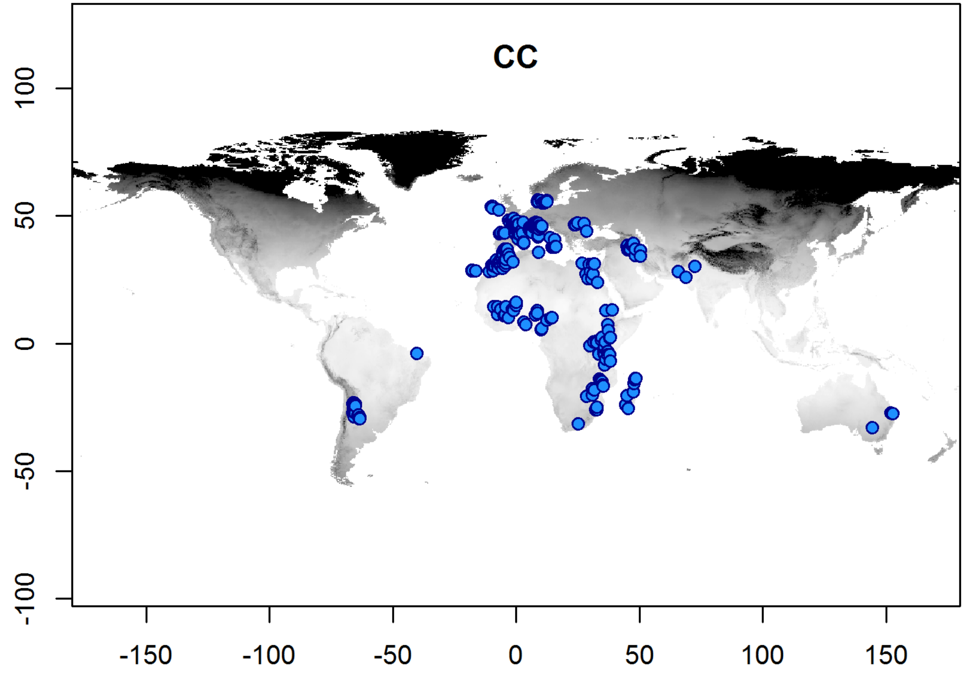

snp51272-scaffold75-5110850

Chromosome: 14 : 56659218

Best association

Environmental variable = bio3

G score = 449

Beta 1 = 2.01

AIC = 1513.94

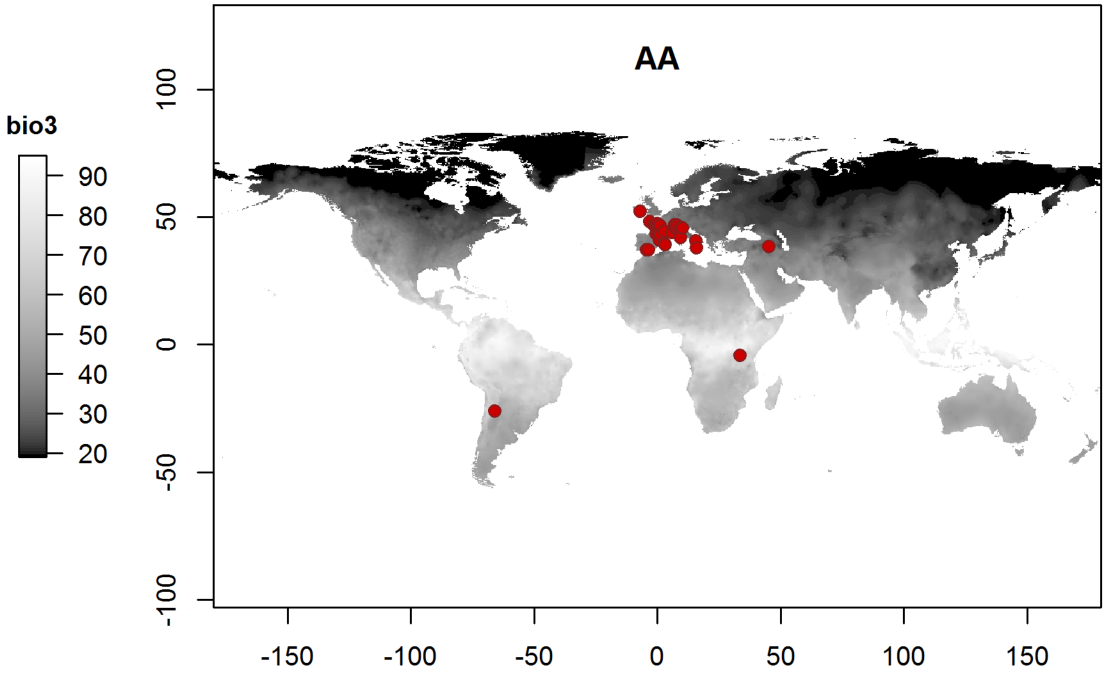

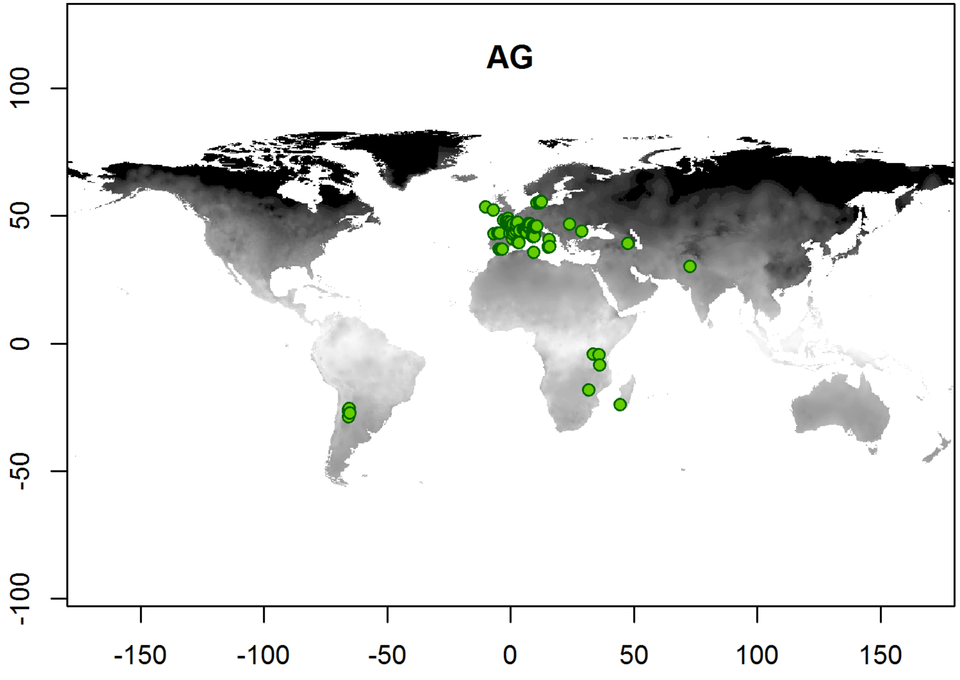

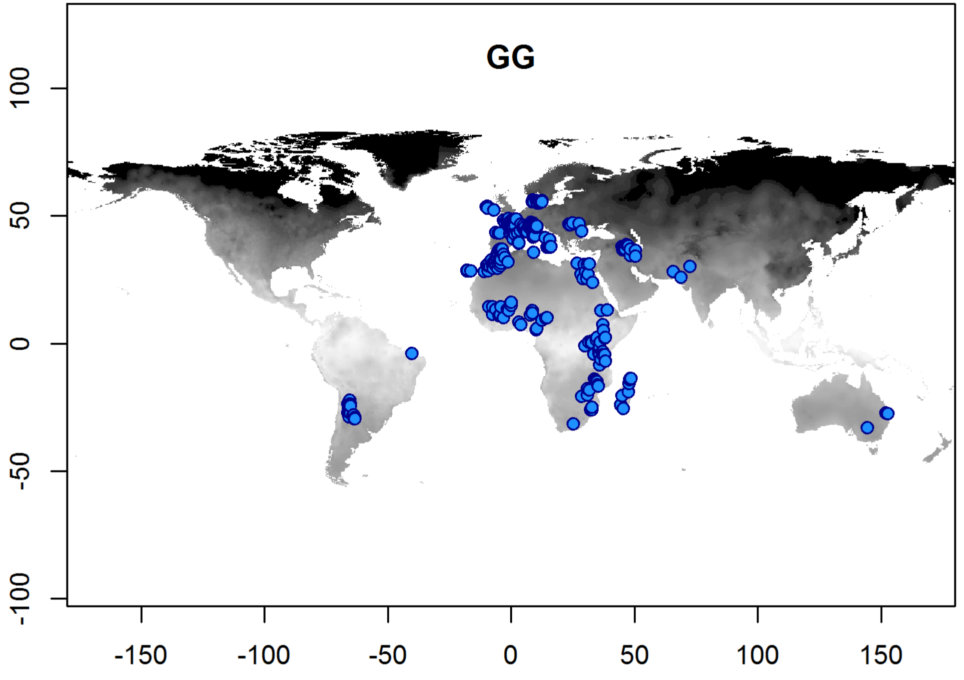

snp49766-scaffold710-2154639

Chromosome: 6 : 19118259

Best association

Environmental variable = bio1

G score = 532.02

Beta 1 = 1.48

AIC = 1674.57

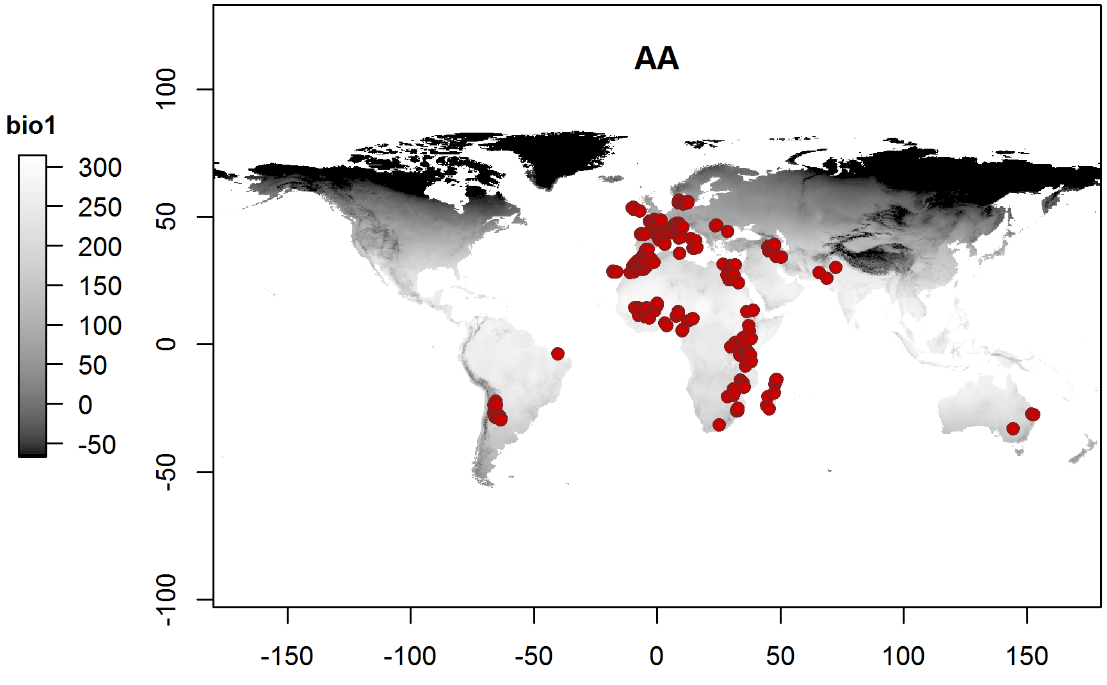

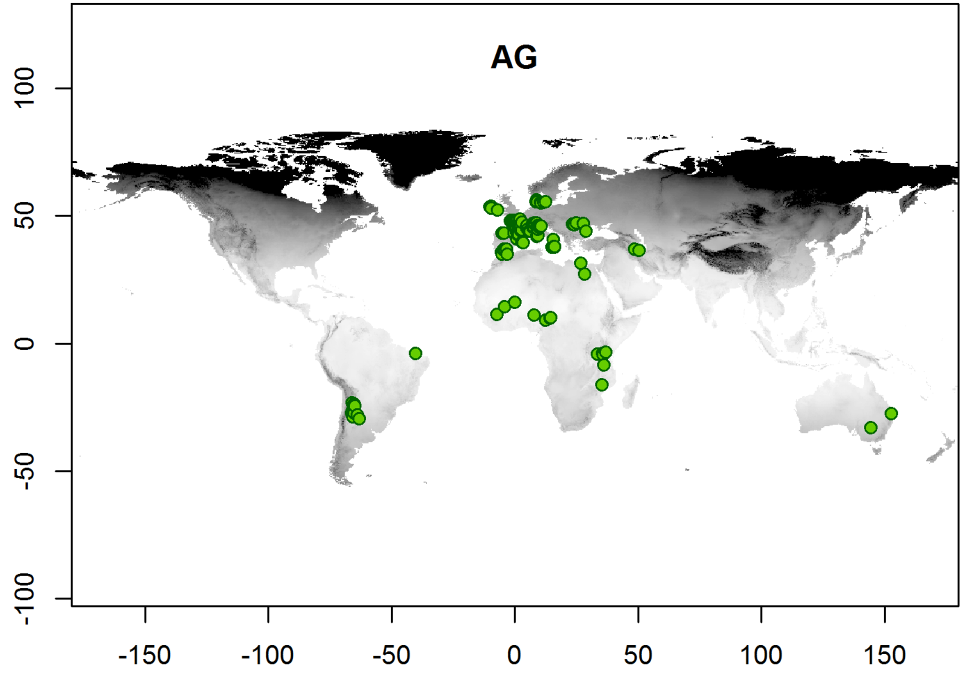

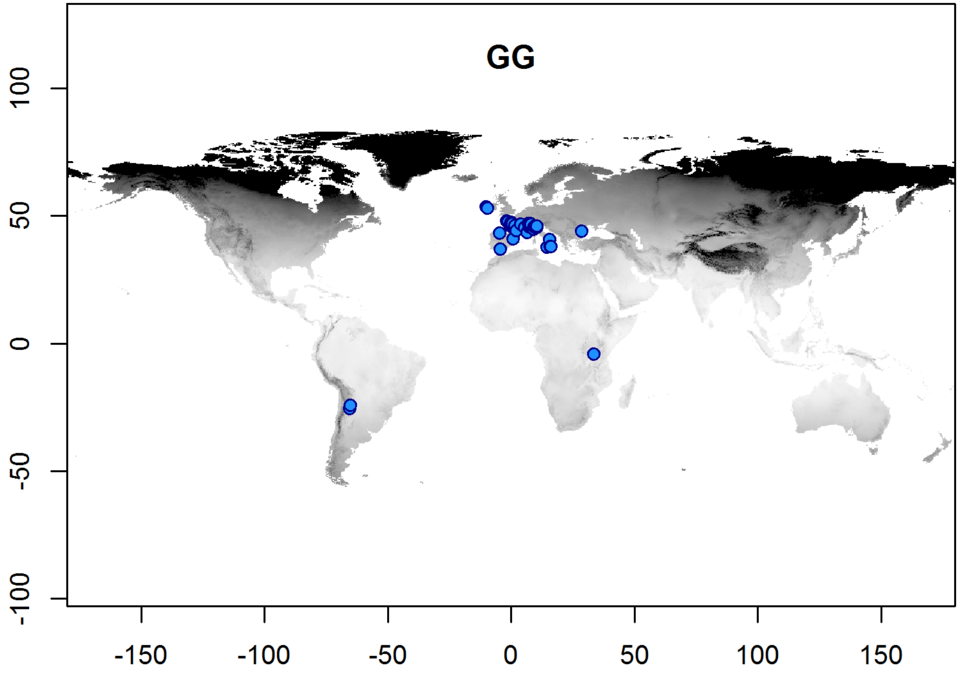

snp46696-scaffold65-2591416

Chromosome: 1 : 106720093

Best association

Environmental variable = bio1

G score = 501.71

Beta 1 = 1.43

AIC = 1687.69

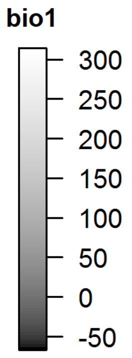

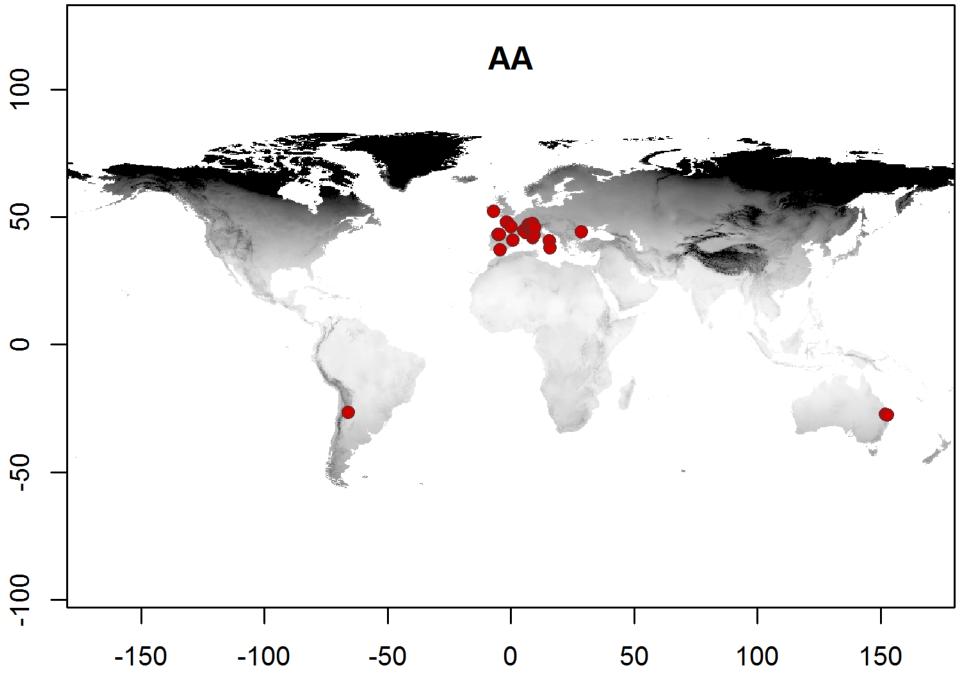

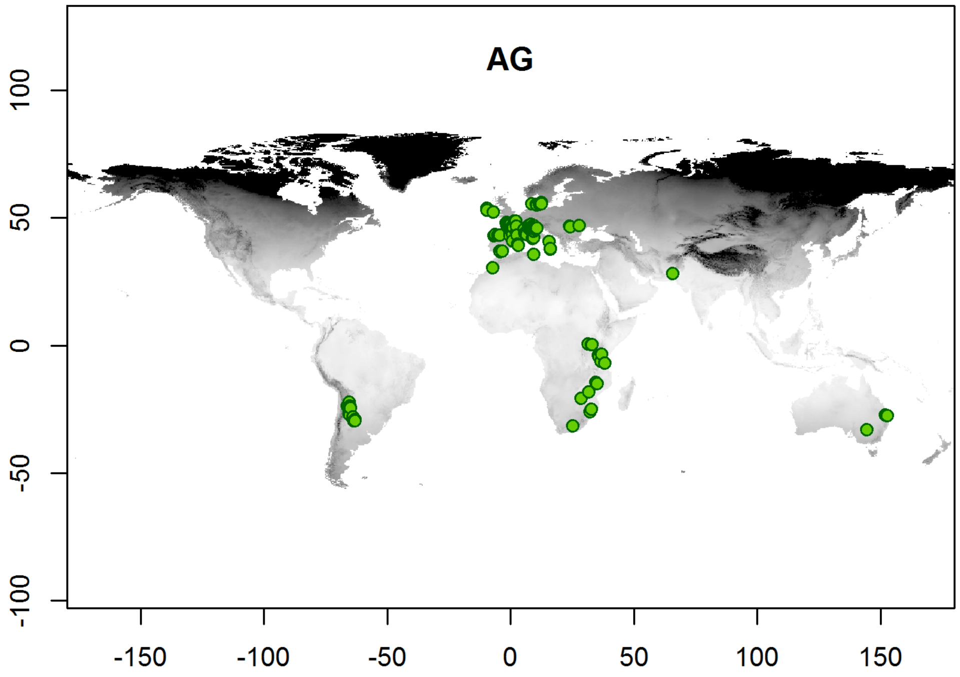

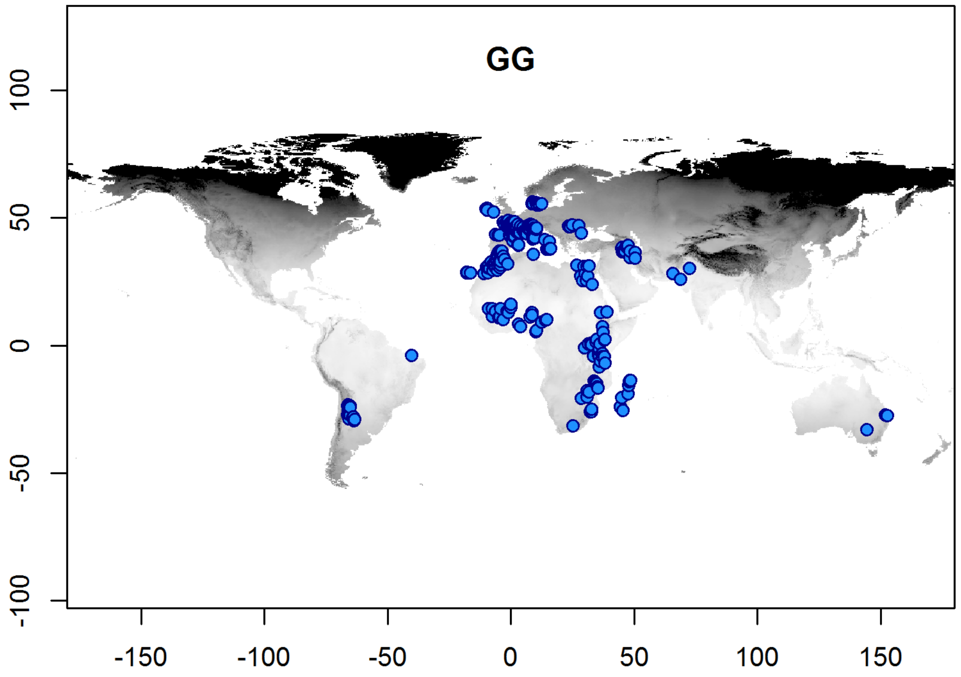

snp46383-scaffold640-488411

Chromosome: 1 : 24332845

Best association

Environmental variable = bio2

G score = 498.17

Beta 1 = 1.47

AIC = 1733.72

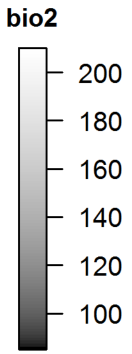

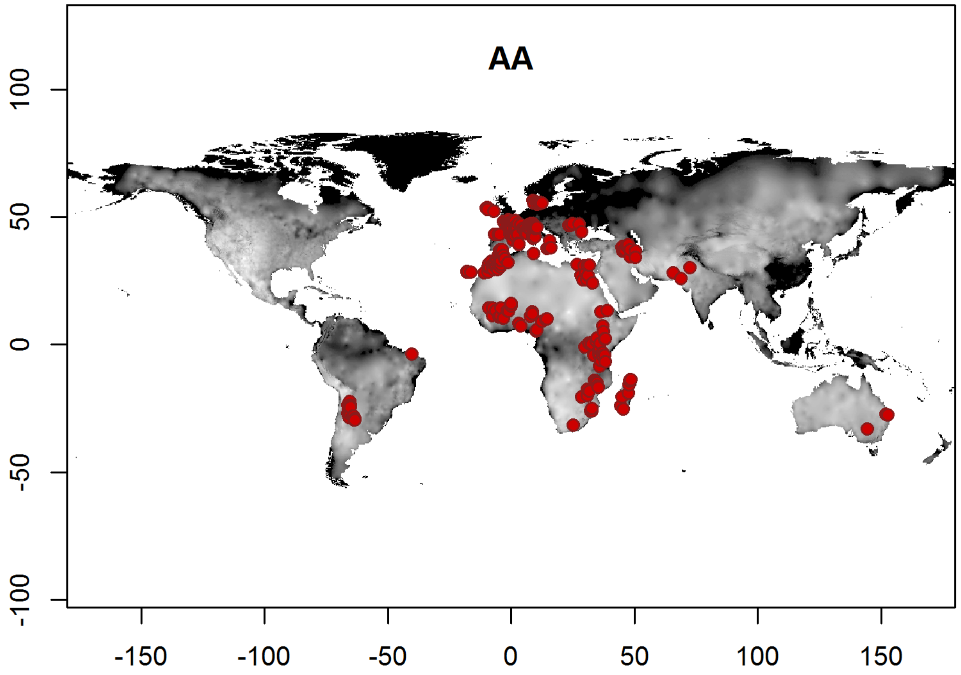

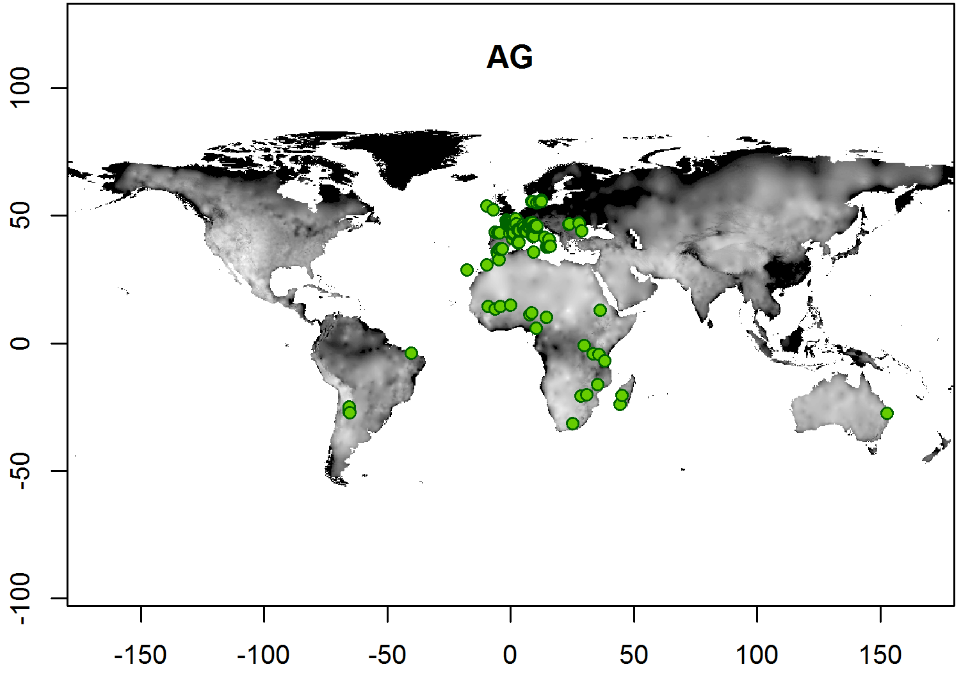

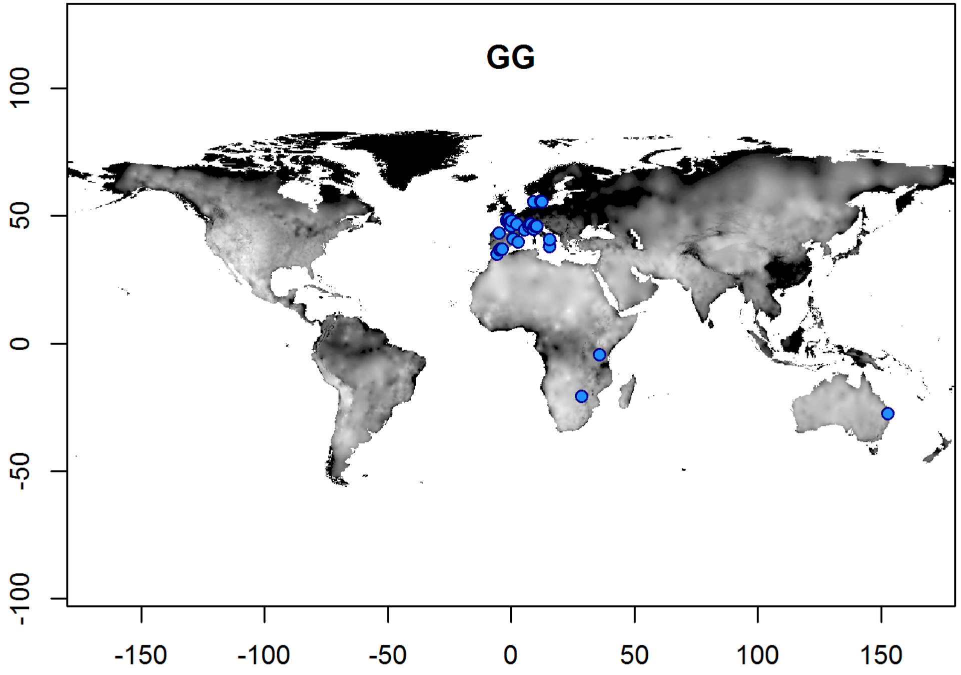

snp44803-scaffold609-3176060

Chromosome: 17 : 17982610

Best association

Environmental variable = bio1

G score = 582.43

Beta 1 = 1.5

AIC = 1760.89

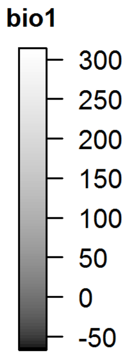

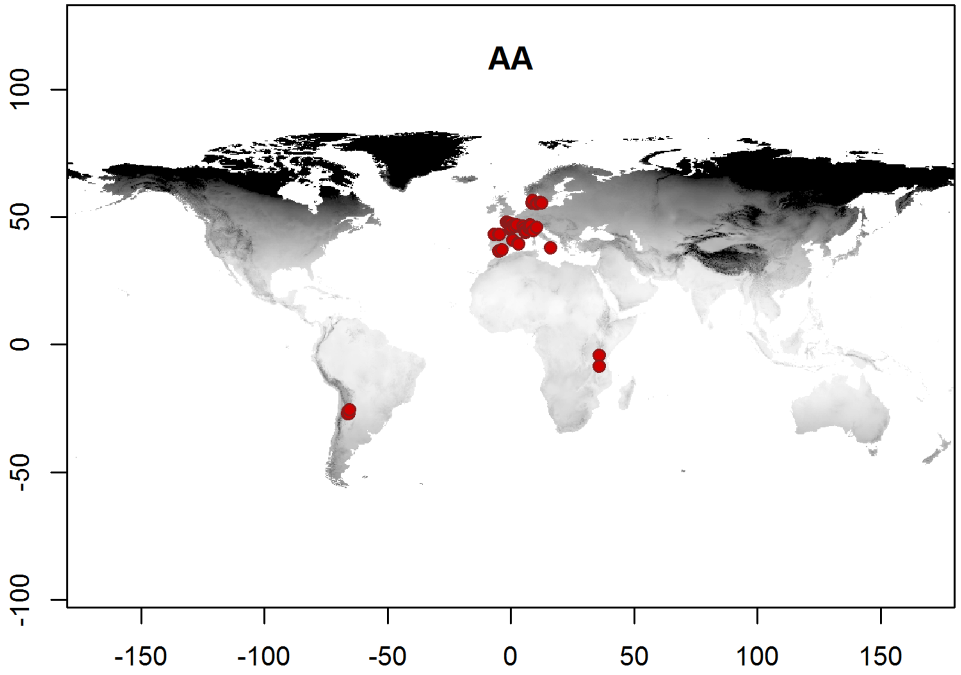

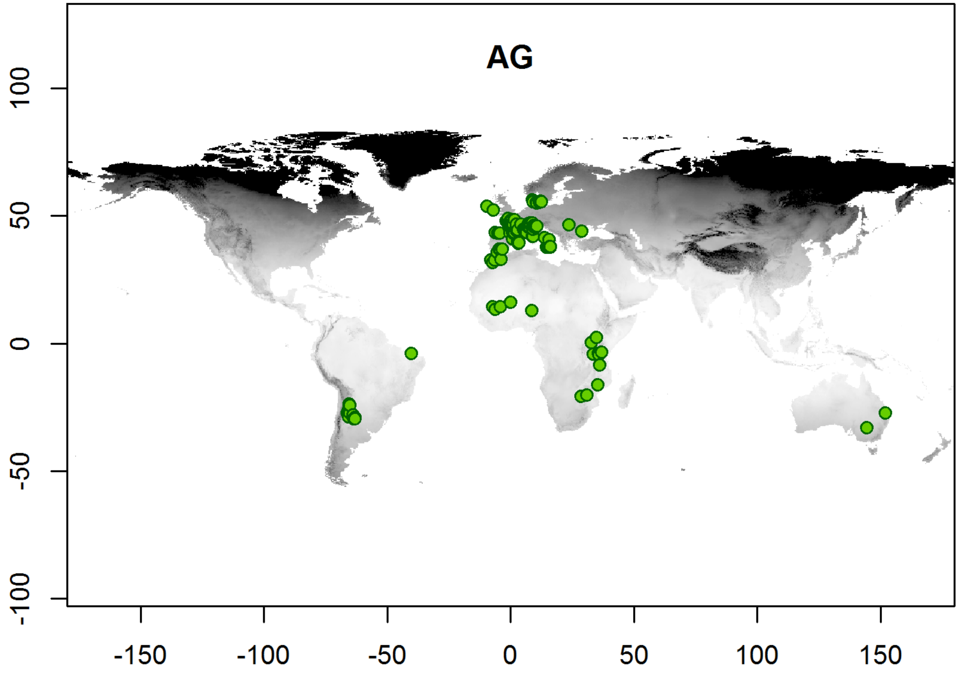

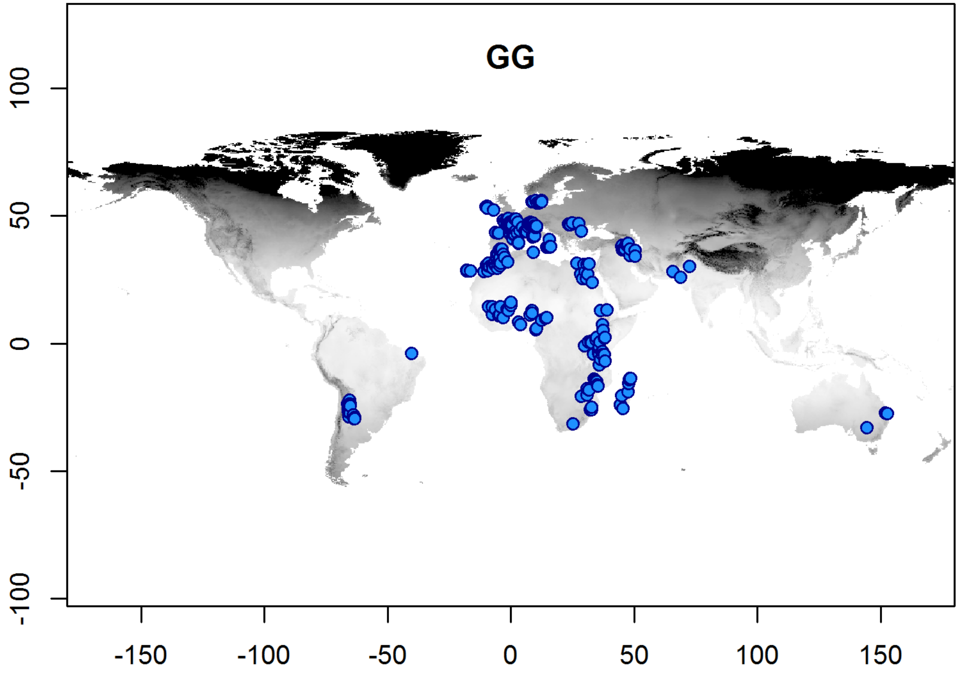

snp43684-scaffold585-2375977

Chromosome: 8 : 78374753

Best association

Environmental variable = bio1

G score = 1146.11

Beta 1 = 2.25

AIC = 1586.95

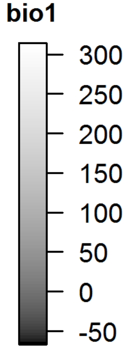

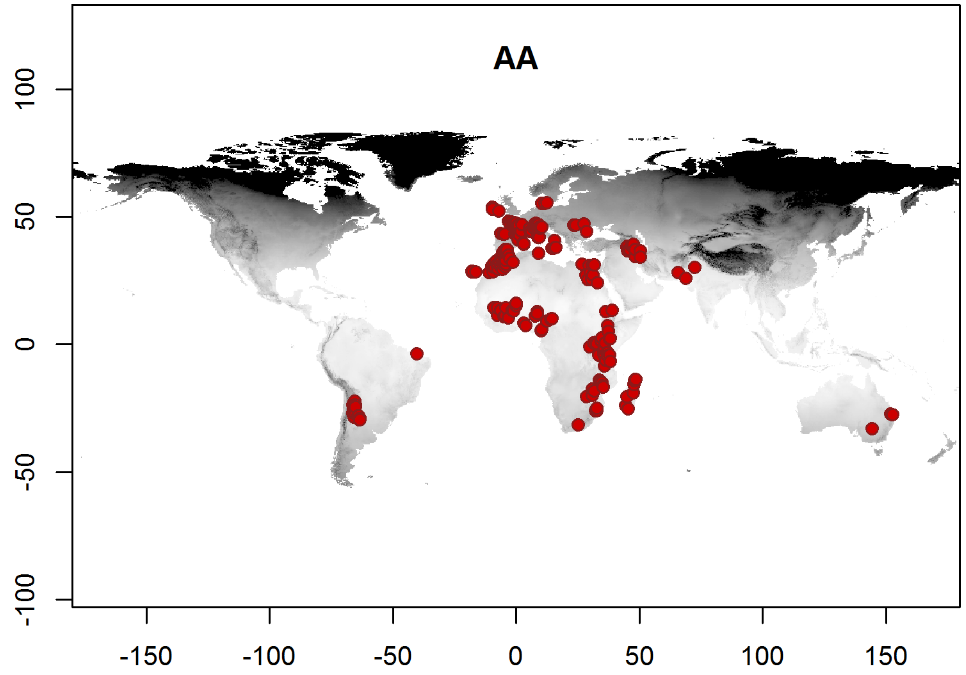

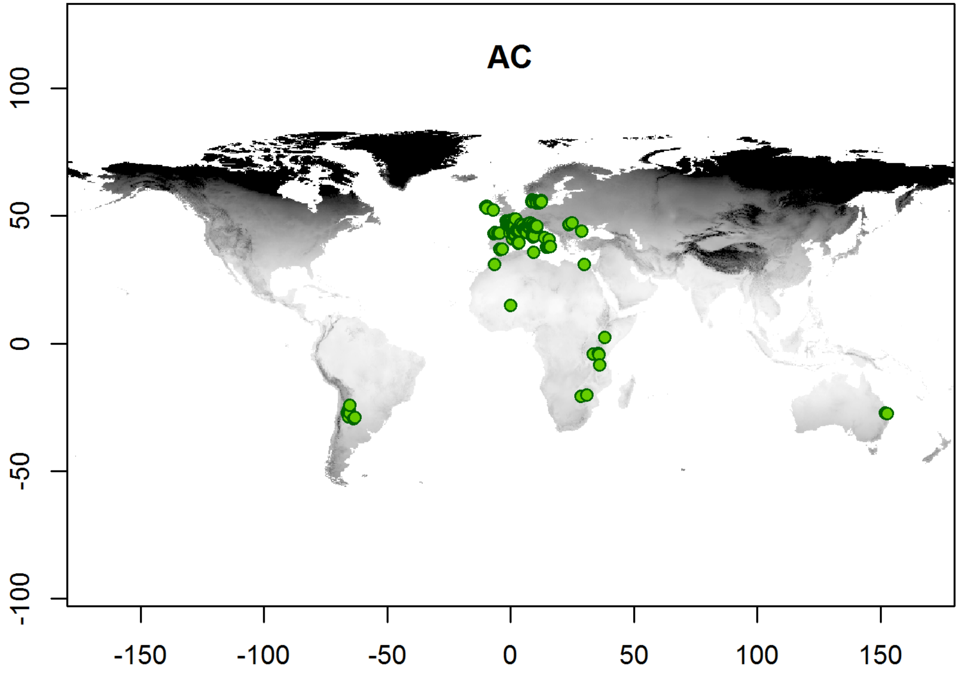

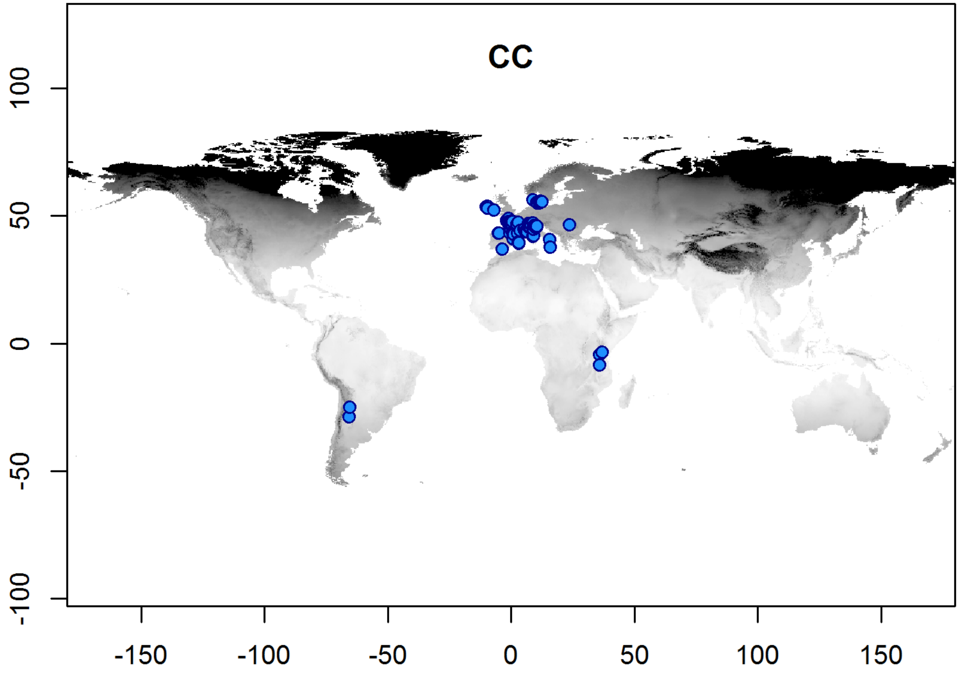

snp43455-scaffold579-4131867

Chromosome: 9 : 62199365

Best association

Environmental variable = bio1

G score = 540.32

Beta 1 = 1.52

AIC = 1632.57

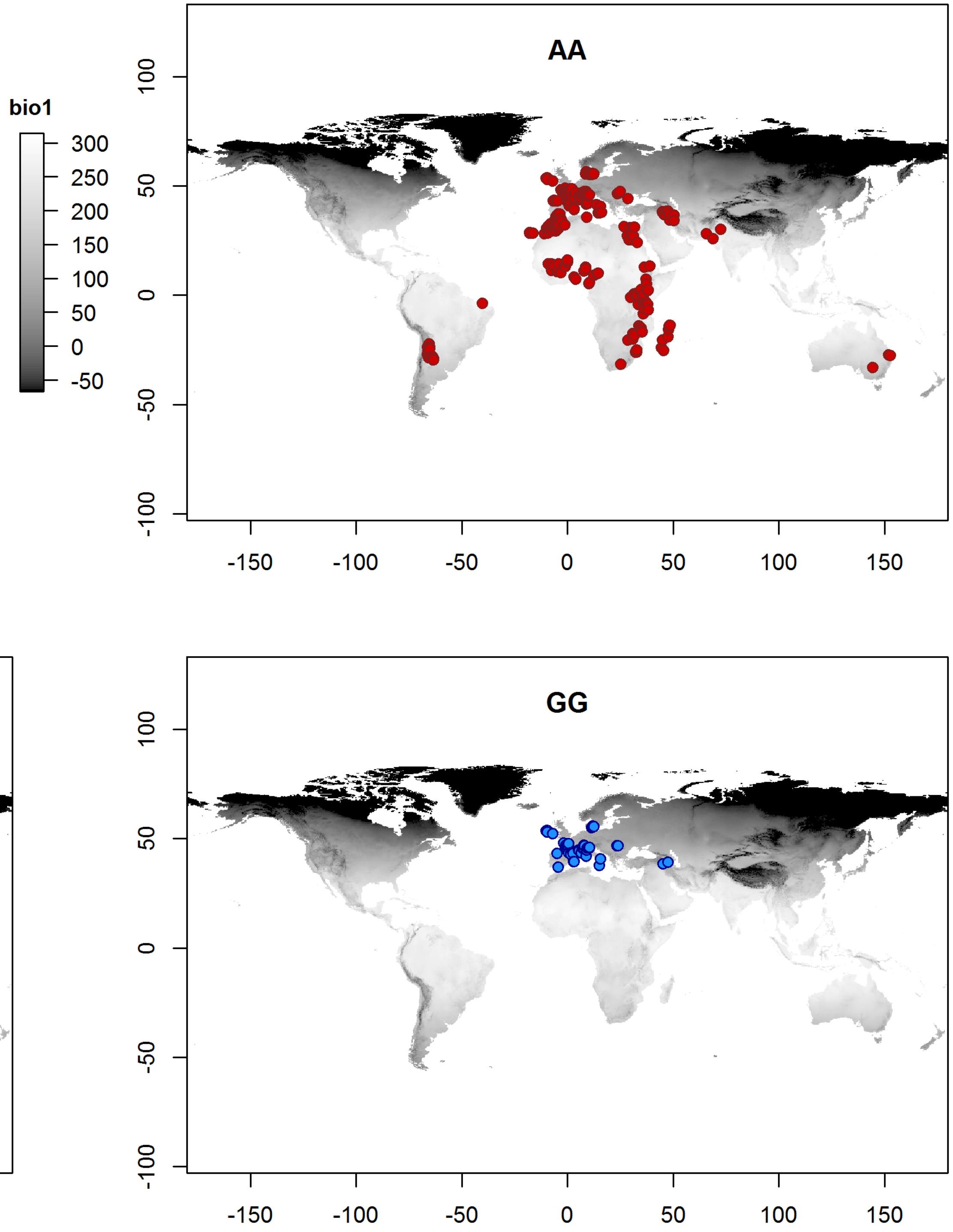

snp43454-scaffold579-4091420

Chromosome: 9 : 62239938

Best association

Environmental variable = bio1

G score = 684.63

Beta 1 = 1.58

AIC = 1820.53

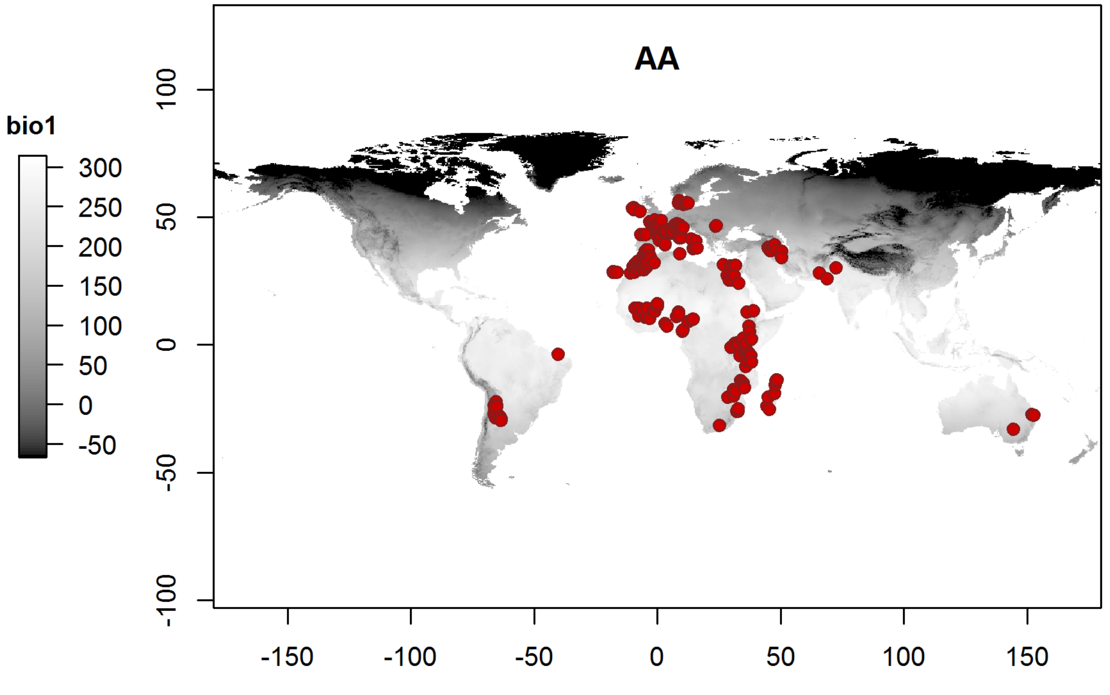

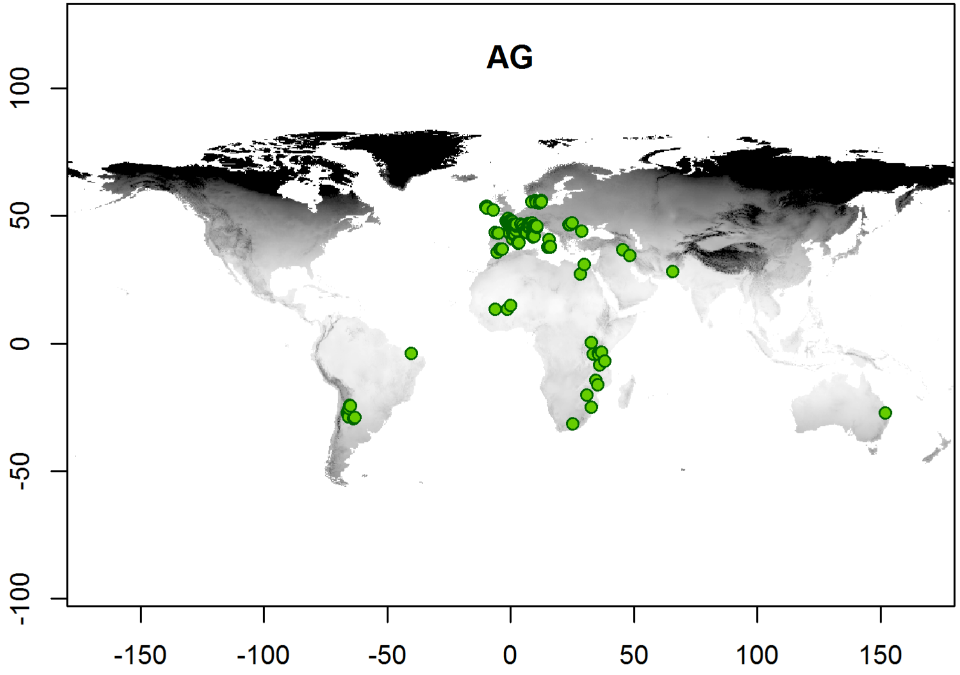

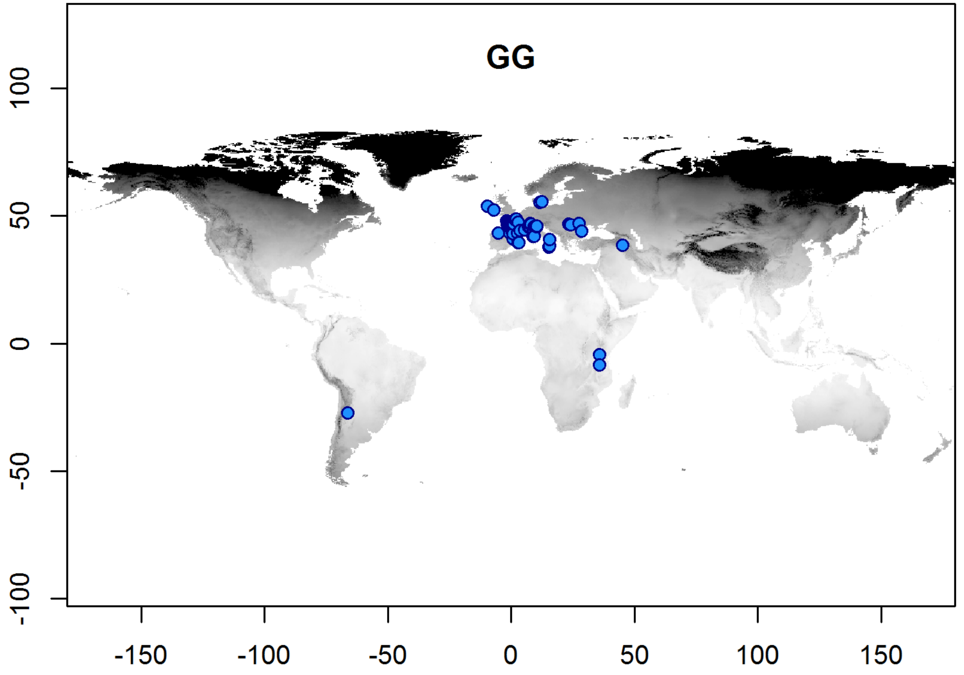

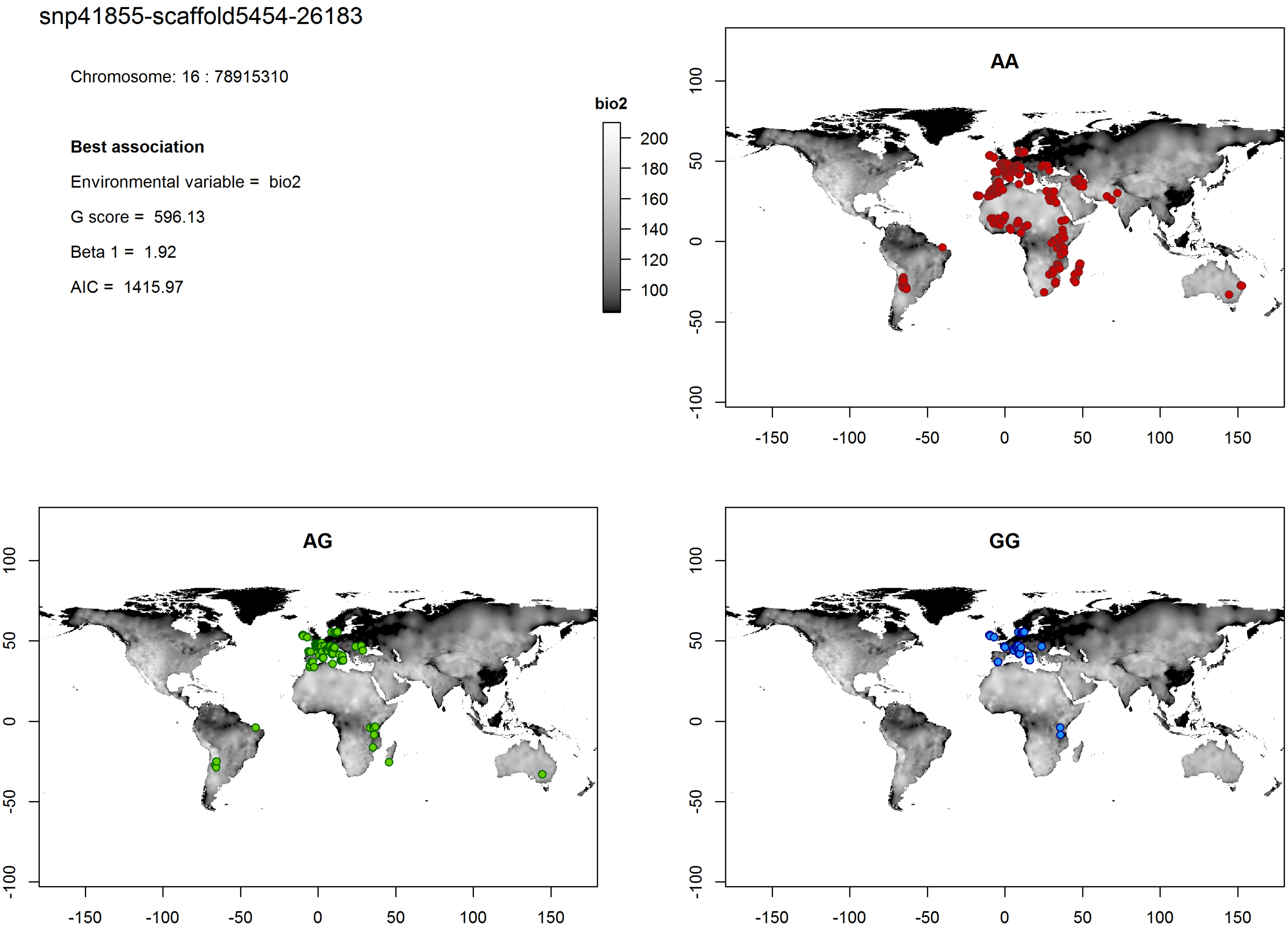
snp41855-scaffold5454-26183
Chromosome: 16 : 78915310
Best association
Environmental variable = bio2
G score = 596.13
Beta 1 = 1.92
AIC = 1415.97
bio2
200
180
160
140
120
100
AA
AG
GG
100
50
0
-50
-100
-150
-100
-50
0
50
100
150

snp40808-scaffold524-376781

Chromosome: 9 : 25627287

Best association

Environmental variable = bio1

G score = 536.66

Beta 1 = 1.5

AIC = 1652.74

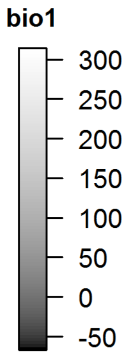

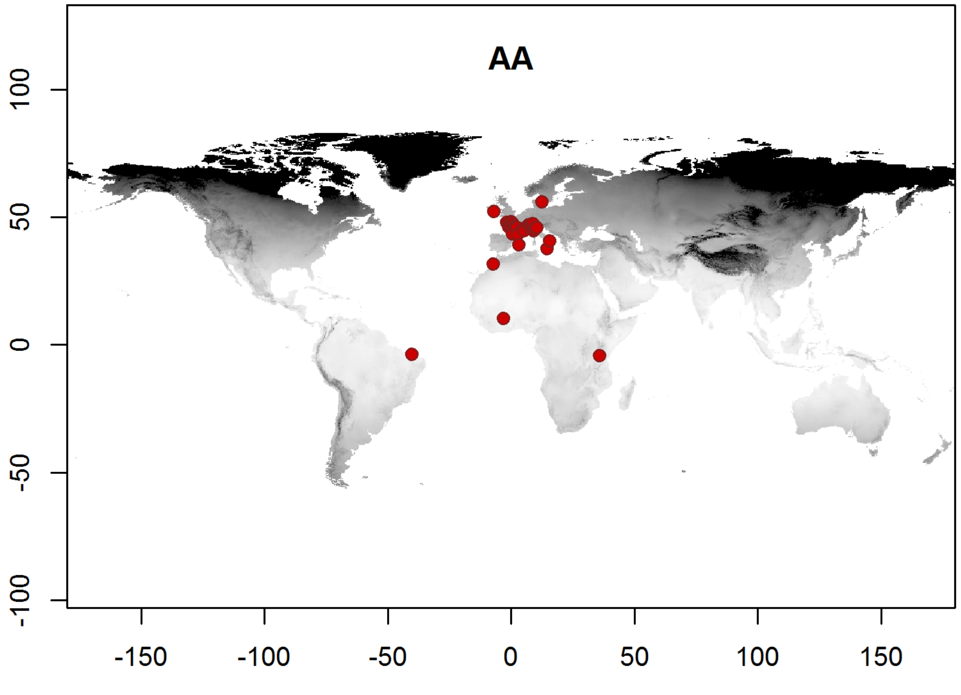

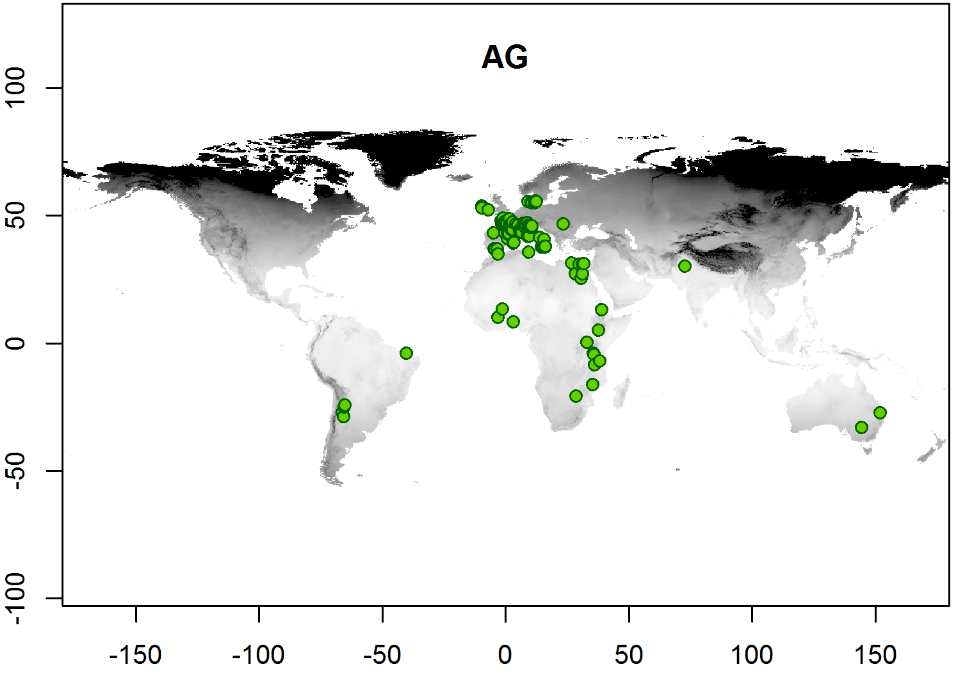

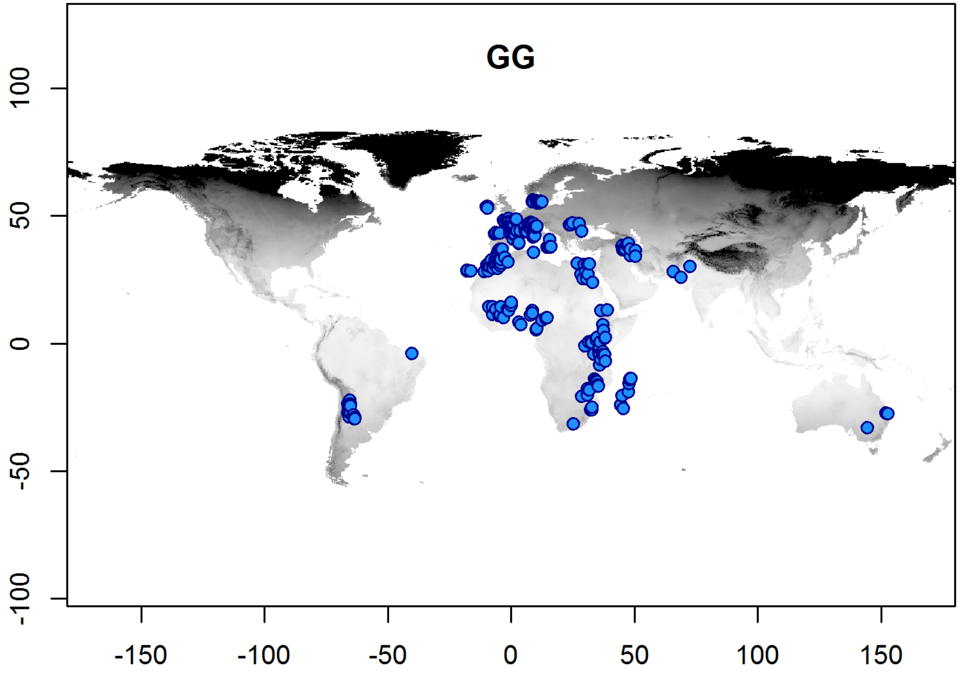

snp40187-scaffold512-1292428

Chromosome: 18 : 20337955

Best association

Environmental variable = bio1

G score = 493.44

Beta 1 = 1.53

AIC = 1520

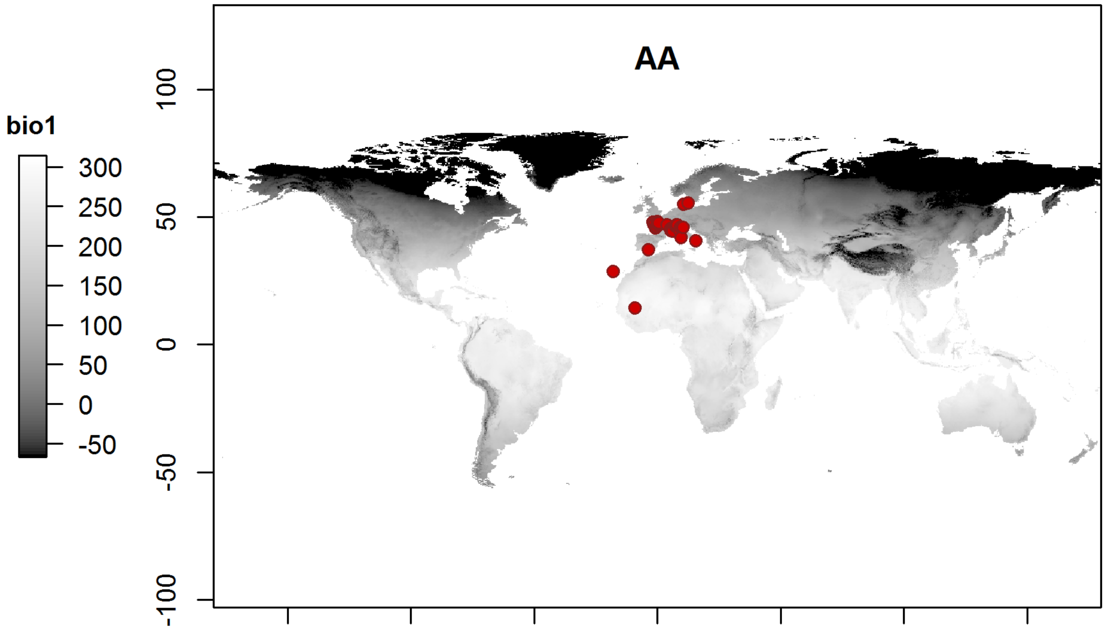

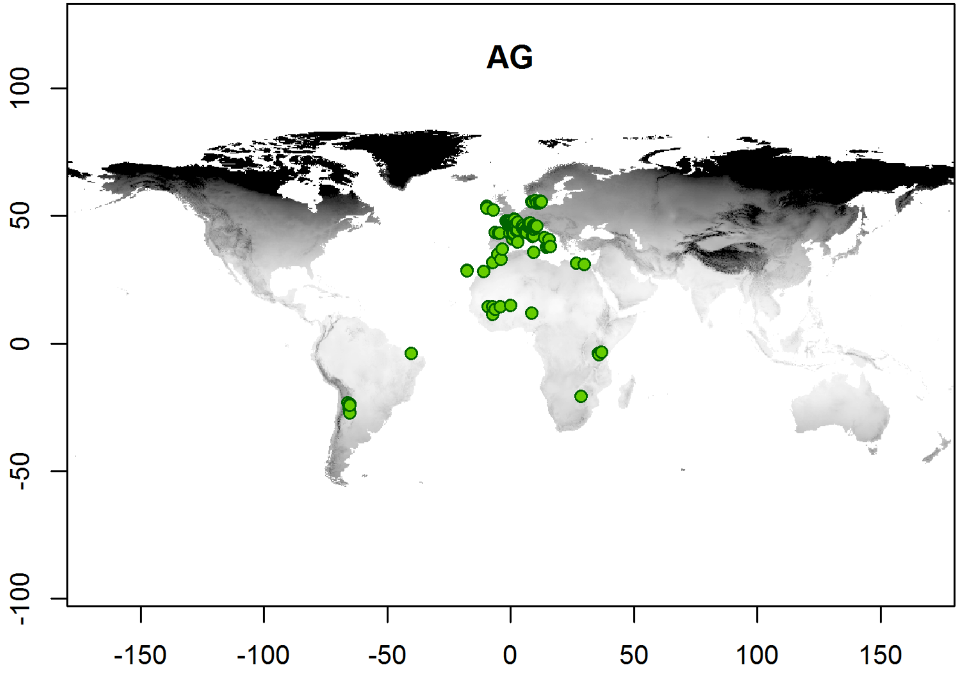

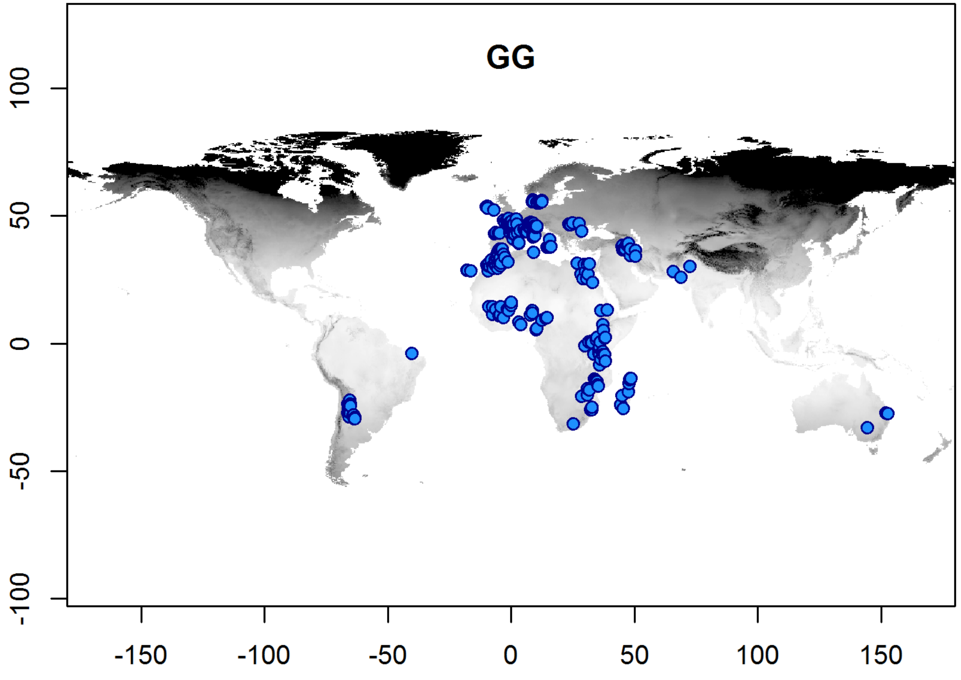

snp39795-scaffold509-2468879

Chromosome: 28 : 13109332

Best association

Environmental variable = bio1

G score = 635.45

Beta 1 = 1.5

AIC = 1866.54

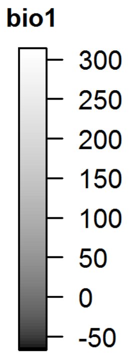

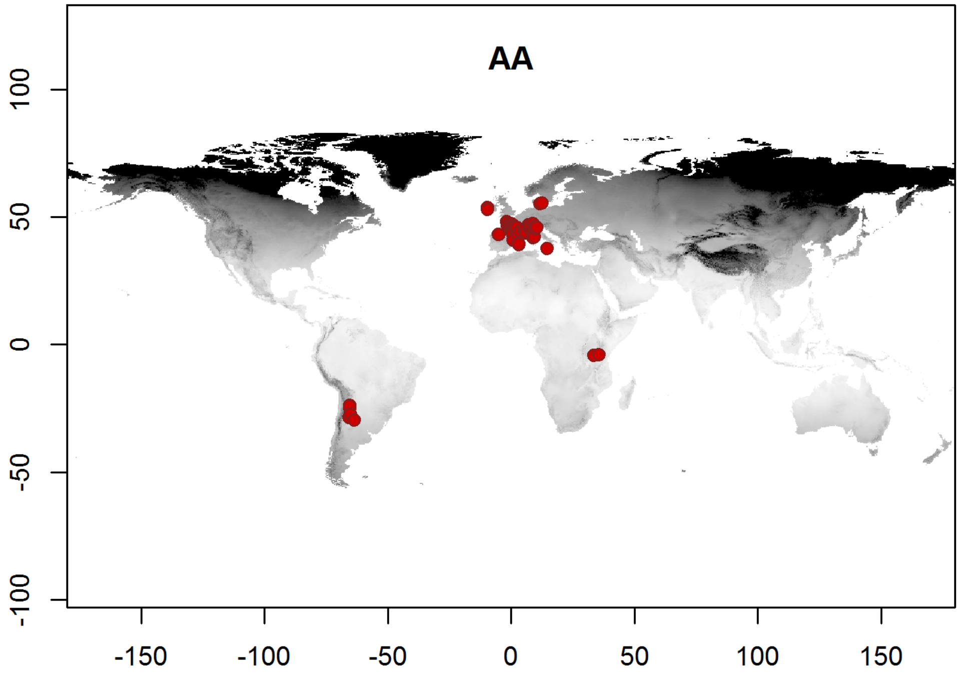

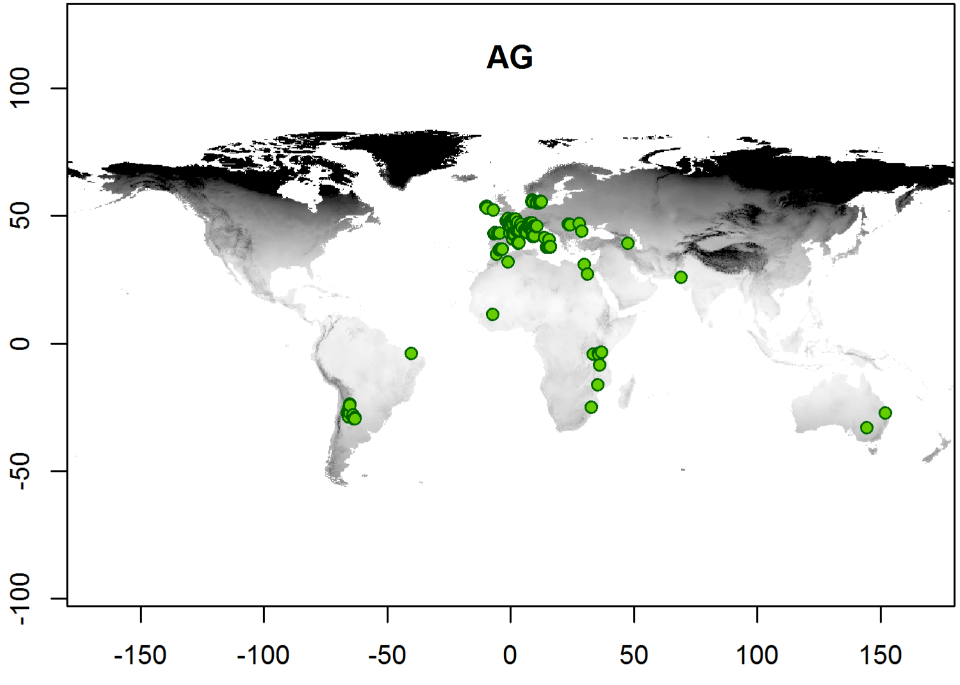

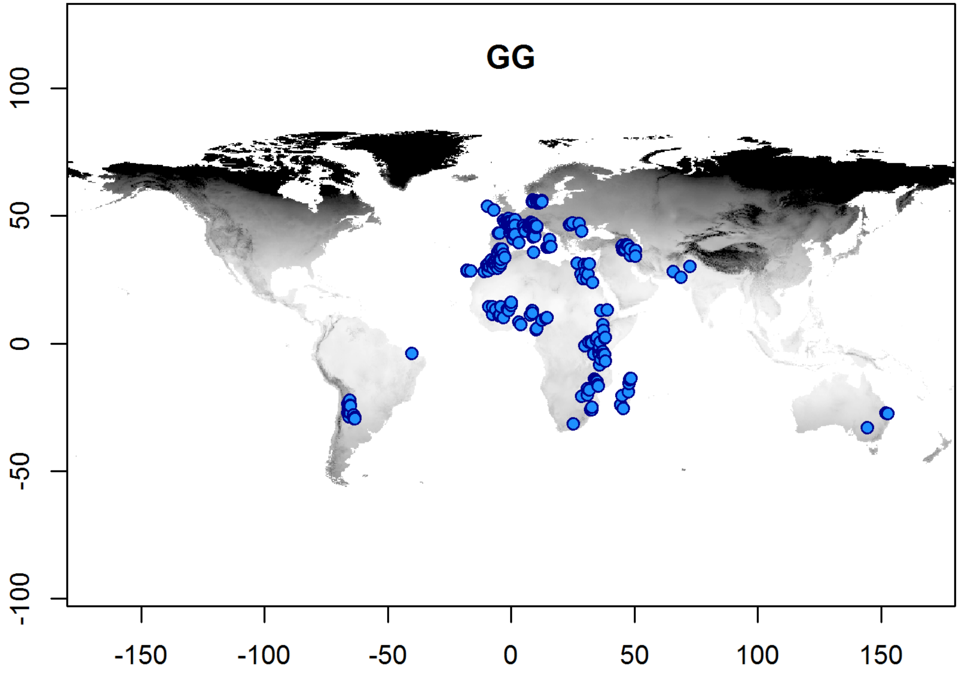

snp39015-scaffold494-5605944

Chromosome: 4 : 42973708

Best association

Environmental variable = bio1

G score = 707.71

Beta 1 = 1.74

AIC = 1636.66

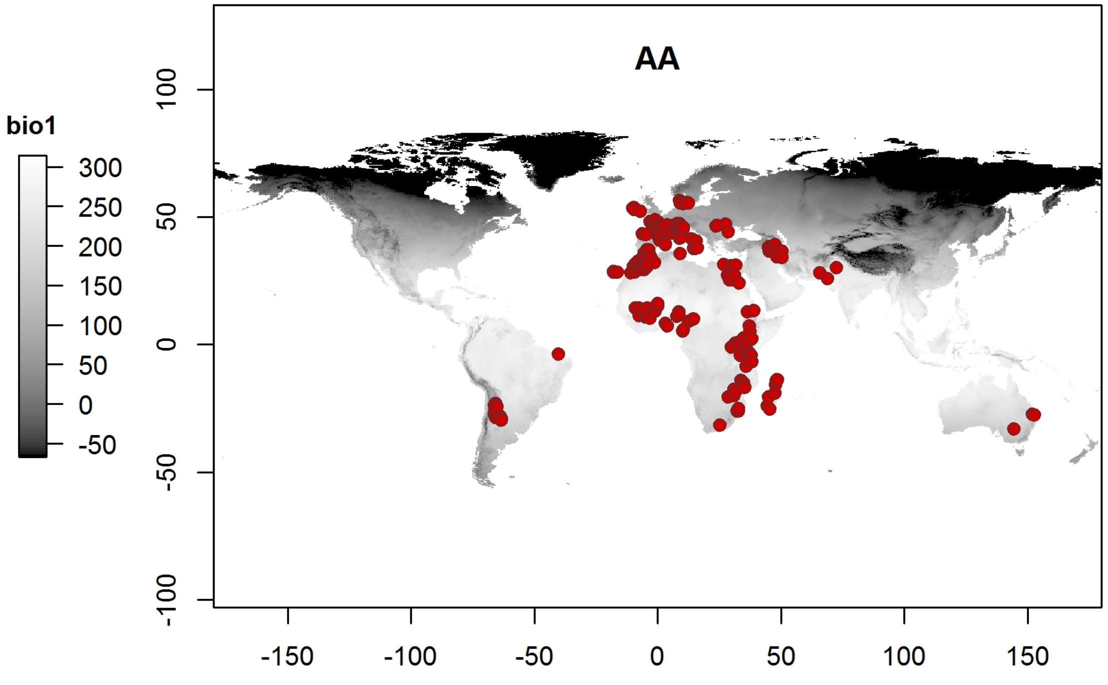

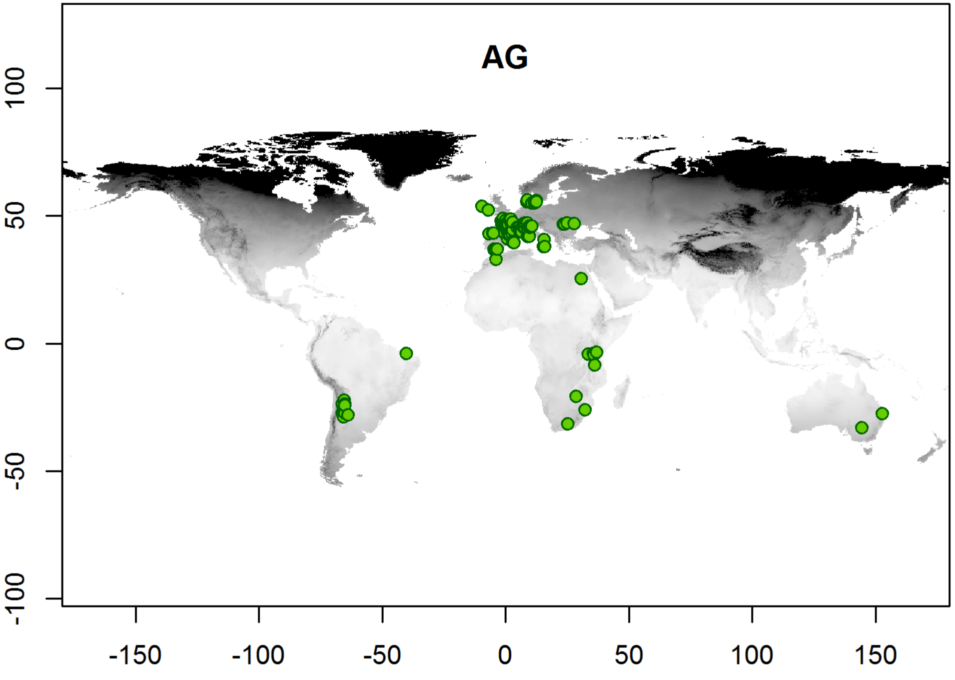

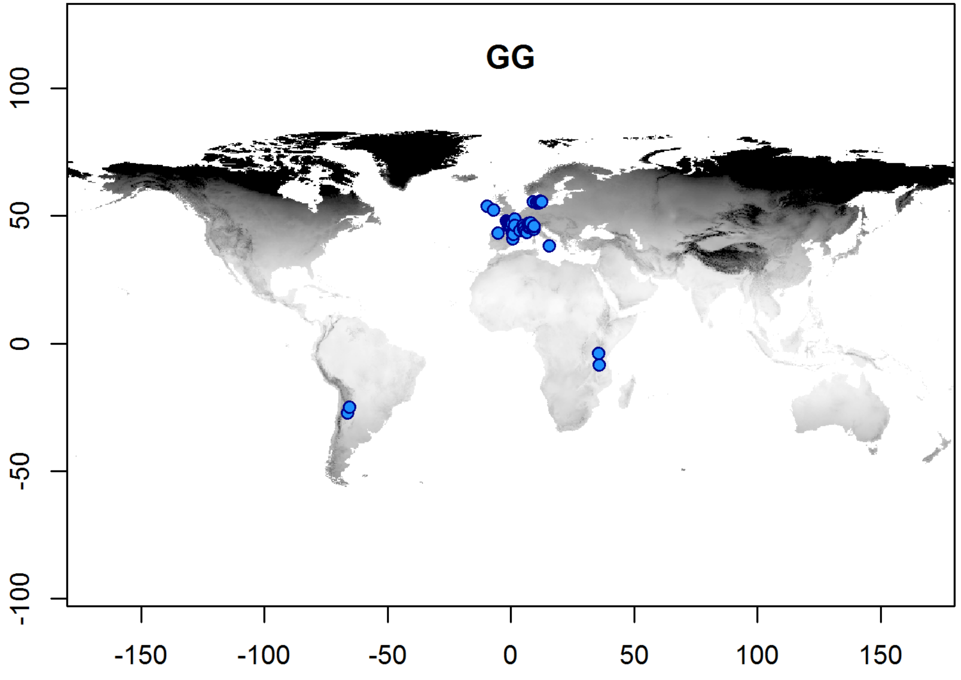

snp38502-scaffold4864-10739

Chromosome: 25 : 41460436

Best association

Environmental variable = bio1

G score = 697.45

Beta 1 = 1.69

AIC = 1689.17

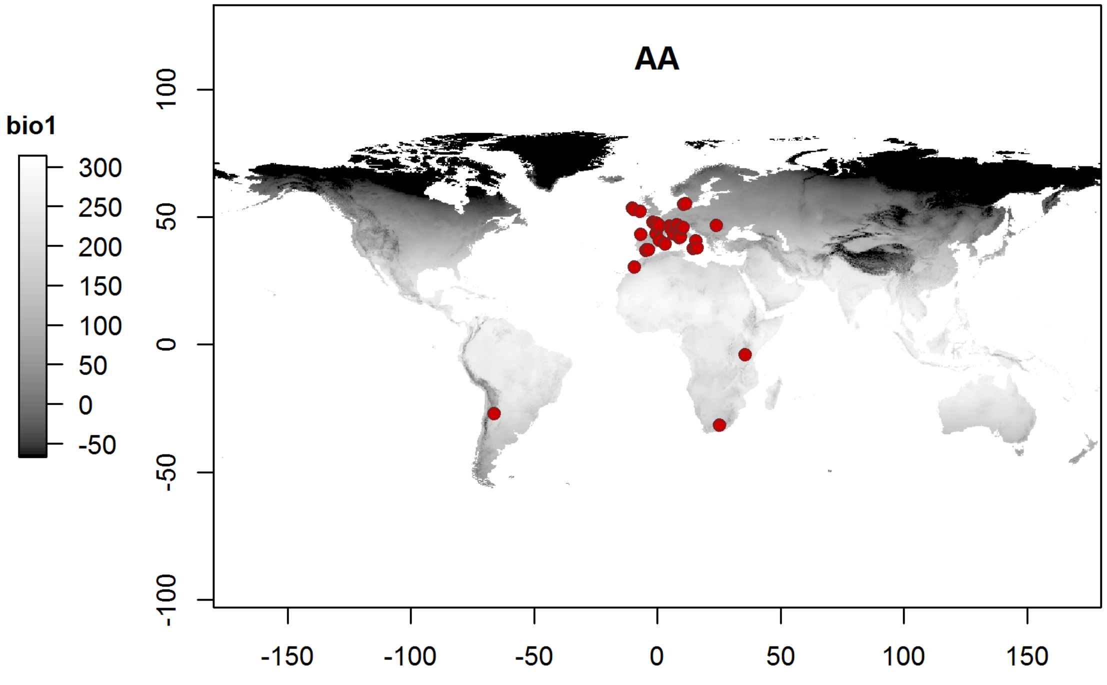

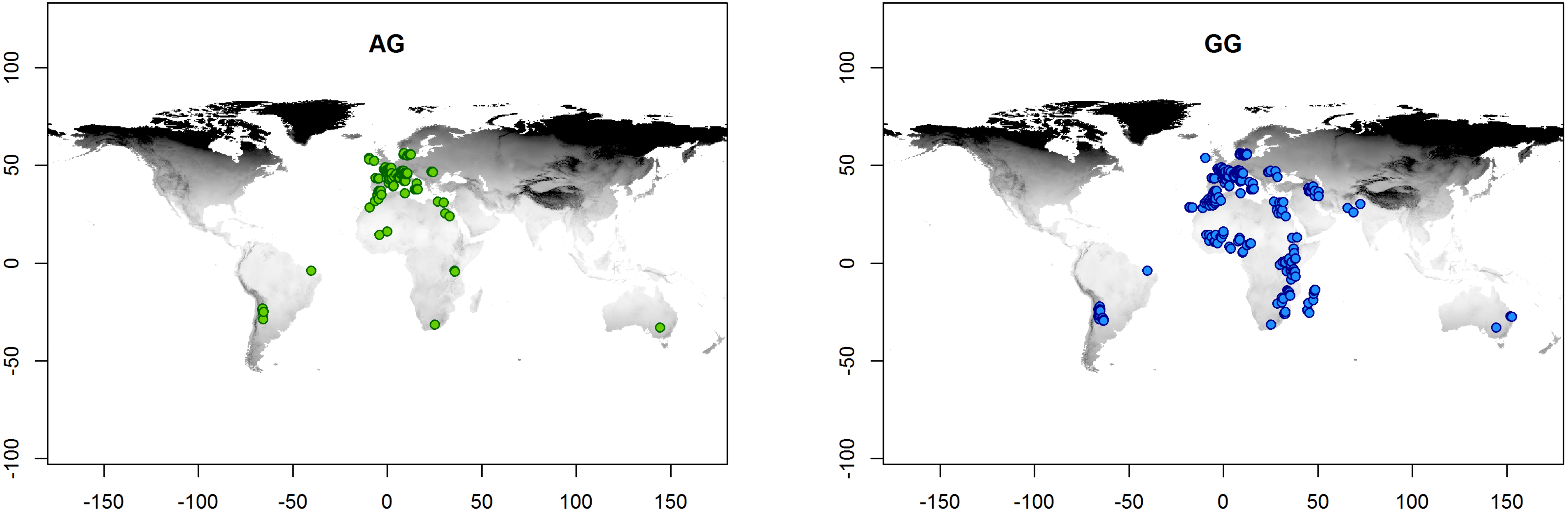

snp38099-scaffold475-377998

Chromosome: 10 : 38273854

Best association

Environmental variable = bio1

G score = 613.87

Beta 1 = 1.58

AIC = 1689.98

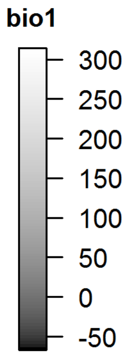

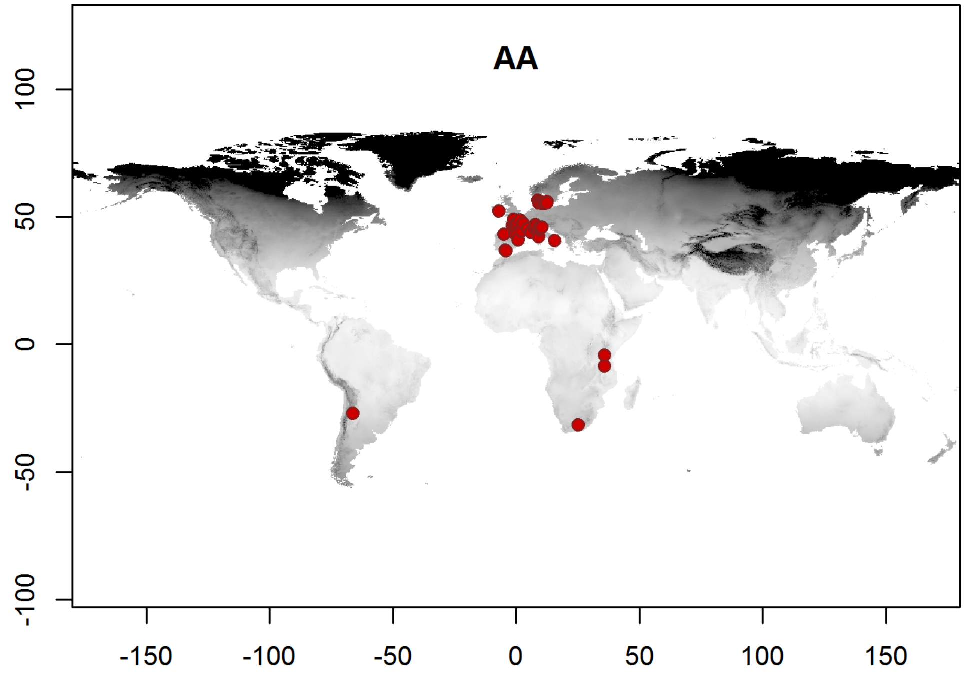

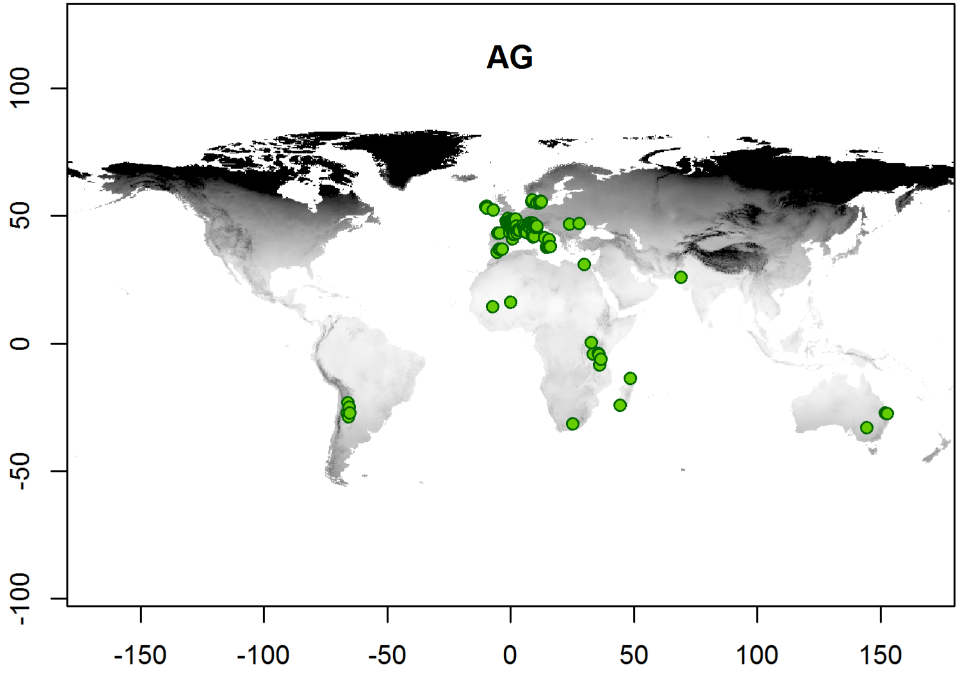

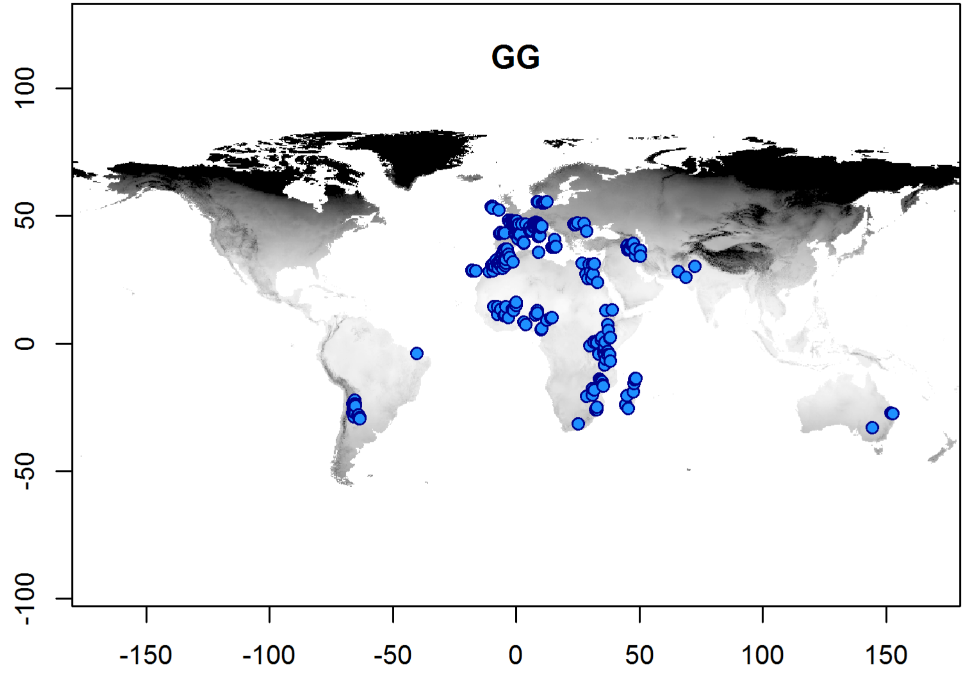

snp34929-scaffold416-2711266

Chromosome: 14 : 36927531

Best association

Environmental variable = bio1

G score = 793.05

Beta 1 = 1.73

AIC = 1771.89

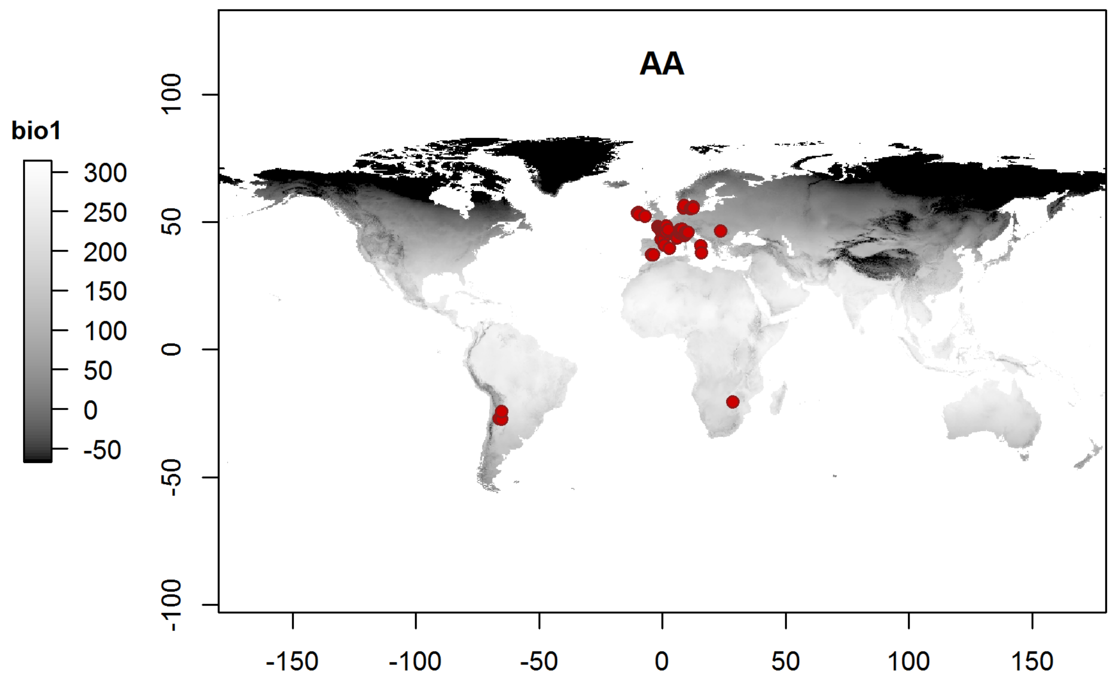

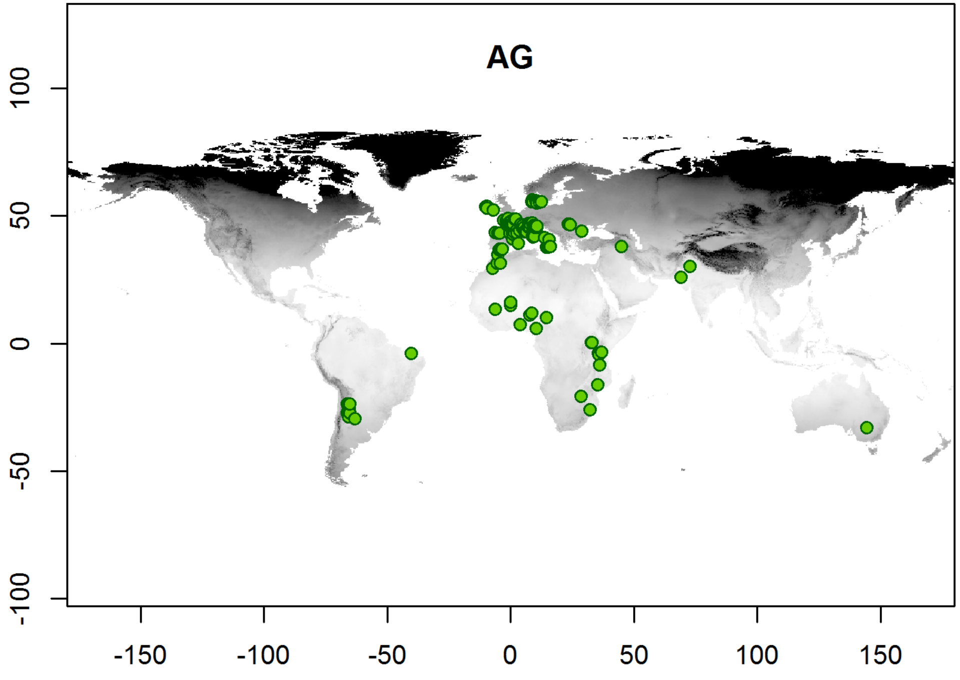

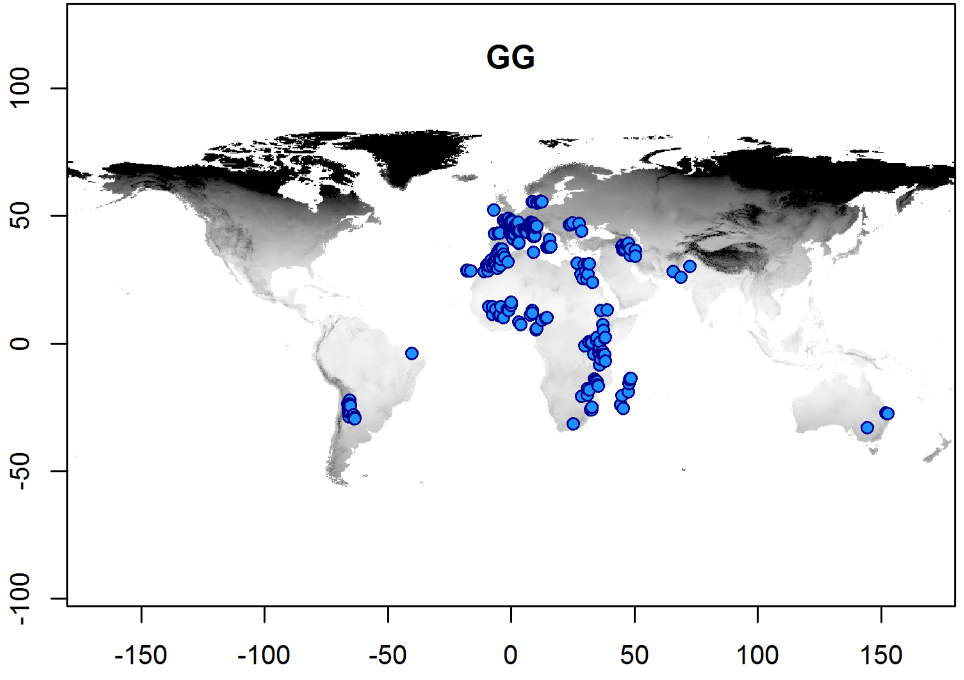

Supplement: Supplementary file 5 — Additional file 5. Genotype distribution of the SNPs detected in the landscape genomics analysis based on the GPS coordinates. Each page represent a SNP, with its coordinates reported on the upper right part of each sheet, with environmental variable associated and statistics (G score, Beta 1 and AIC). The three possible genotypes are labelled with three different colors: AA = red; AB = green; BB = blue [file 12711_2018_421_MOESM5_ESM.pdf]
